# Supplementary material for: A conserved phage phosphoesterase enables evasion of bacterial antiviral immunity
Source: EMBO Rep. 2025 May 29;26(14):3594–613. doi: 10.1038/s44319-025-00488-4 (PMC12287305; doi:10.1038/s44319-025-00488-4)
Supplement: Supplementary file 1 — Appendix [file 44319_2025_488_MOESM1_ESM.pdf]

Appendix

A conserved phage phosphoesterase enables evasion of bacterial antiviral immunity

Junlong Li, Yihao Song, Xiao Guo, Zheng-Guo He

Table of Contents

Appendix Figure S1. Quantification of plaque-forming units (PFU) following BRED-mediated individual gene knockout in phage A10ZJ24 (pages 2-4).
Appendix Figure S2. Assays for the phage gene importance for the infection of A10ZJ24 to different host (page 5).
Appendix Figure S3. Comparative analysis of the genome abundance between wild-type and mutant phage A10ZJ24 following infection of *M. smegmatis* strain (page 6).
Appendix Figure S4. Comparative analysis of the plaque-forming efficiency between A10ZJ24 and its *gp48*-deletion mutant (page 7).
Appendix Figure S5. A10ZJ24 *gp48* expression is toxic to mycobacteria and disrupts the genomic integrity of *M. smegmatis* strain (pages 8-9).
Appendix Figure S6. Assays for the defense activity of anti-phage genes and their transcriptional dynamics post-infection with A10ZJ24 (pages 10-11).
Appendix Figure S7. Assays for defense activity of several anti-phage genes in *M. tuberculosis* H37Ra (pages 12-13).

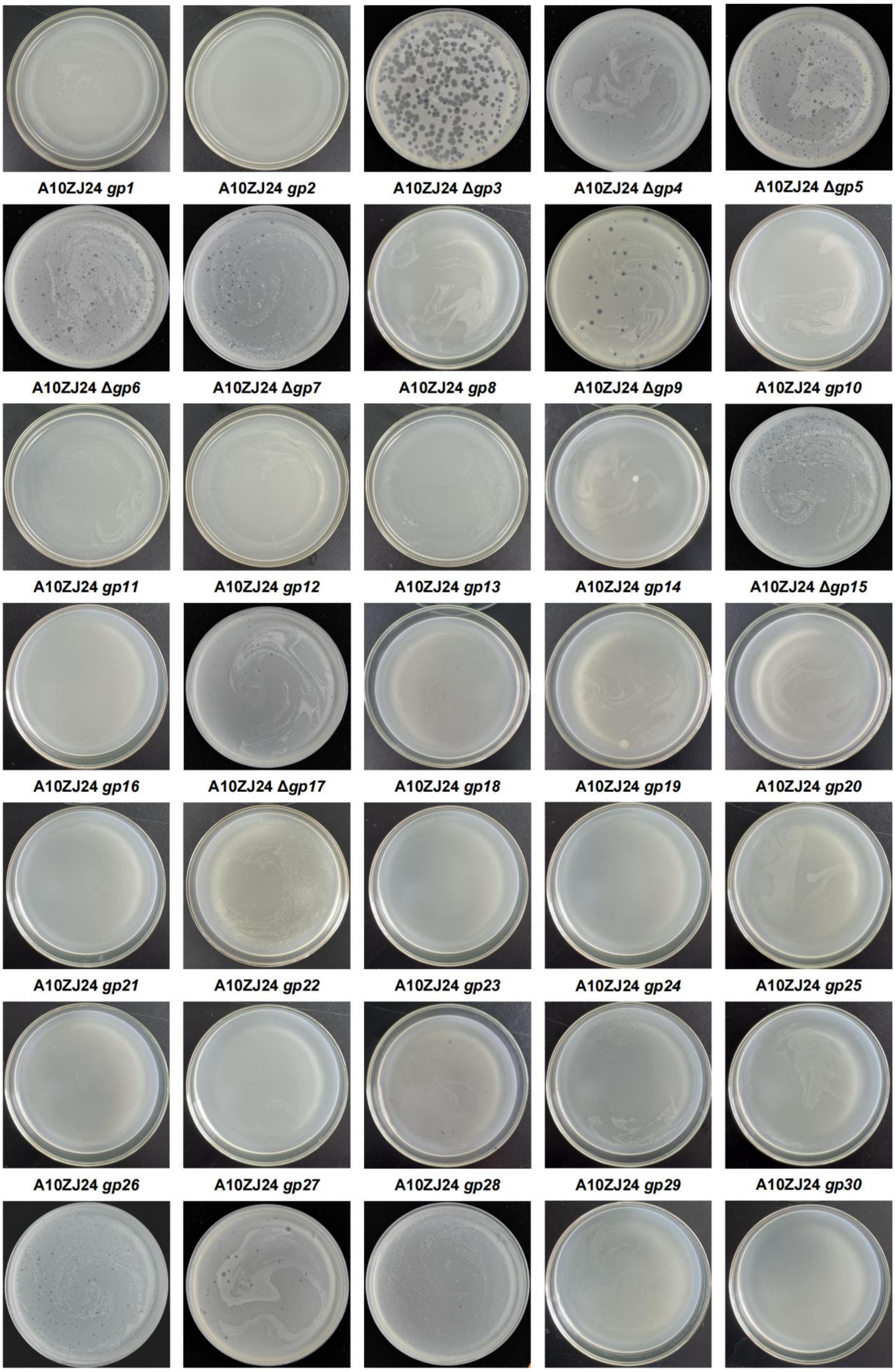
A10ZJ24 *gp1*
A10ZJ24 *gp2*
A10ZJ24 Δ*gp3*
A10ZJ24 Δ*gp4*
A10ZJ24 Δ*gp5*
A10ZJ24 Δ*gp6*
A10ZJ24 Δ*gp7*
A10ZJ24 *gp8*
A10ZJ24 Δ*gp9*
A10ZJ24 *gp10*
A10ZJ24 *gp11*
A10ZJ24 *gp12*
A10ZJ24 *gp13*
A10ZJ24 *gp14*
A10ZJ24 Δ*gp15*
A10ZJ24 *gp16*
A10ZJ24 Δ*gp17*
A10ZJ24 *gp18*
A10ZJ24 *gp19*
A10ZJ24 *gp20*
A10ZJ24 *gp21*
A10ZJ24 *gp22*
A10ZJ24 *gp23*
A10ZJ24 *gp24*
A10ZJ24 *gp25*
A10ZJ24 *gp26*
A10ZJ24 *gp27*
A10ZJ24 *gp28*
A10ZJ24 *gp29*
A10ZJ24 *gp30*
A10ZJ24 Δ*gp31*
A10ZJ24 Δ*gp32*
A10ZJ24 Δ*gp33*
A10ZJ24 *gp34*
A10ZJ24 *gp35*

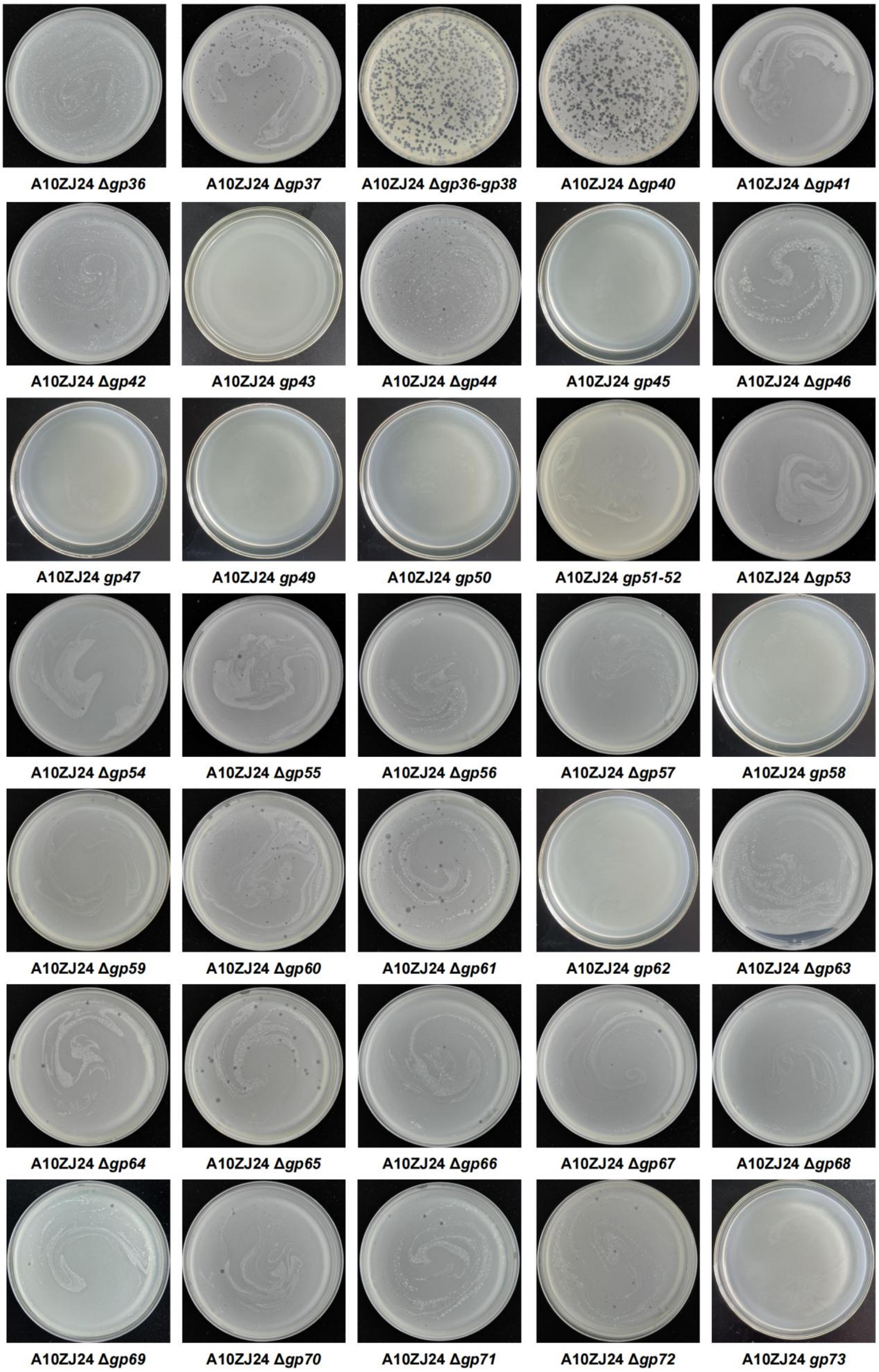
A10ZJ24 Δ*gp36*
A10ZJ24 Δ*gp37*
A10ZJ24 Δ*gp36-gp38*
A10ZJ24 Δ*gp40*
A10ZJ24 Δ*gp41*
A10ZJ24 Δ*gp42*
A10ZJ24 *gp43*
A10ZJ24 Δ*gp44*
A10ZJ24 *gp45*
A10ZJ24 Δ*gp46*
A10ZJ24 *gp47*
A10ZJ24 *gp49*
A10ZJ24 *gp50*
A10ZJ24 *gp51-52*
A10ZJ24 Δ*gp53*
A10ZJ24 Δ*gp54*
A10ZJ24 Δ*gp55*
A10ZJ24 Δ*gp56*
A10ZJ24 Δ*gp57*
A10ZJ24 *gp58*
A10ZJ24 Δ*gp59*
A10ZJ24 Δ*gp60*
A10ZJ24 Δ*gp61*
A10ZJ24 *gp62*
A10ZJ24 Δ*gp63*
A10ZJ24 Δ*gp64*
A10ZJ24 Δ*gp65*
A10ZJ24 Δ*gp66*
A10ZJ24 Δ*gp67*
A10ZJ24 Δ*gp68*
A10ZJ24 Δ*gp69*
A10ZJ24 Δ*gp70*
A10ZJ24 Δ*gp71*
A10ZJ24 Δ*gp72*
A10ZJ24 *gp73*

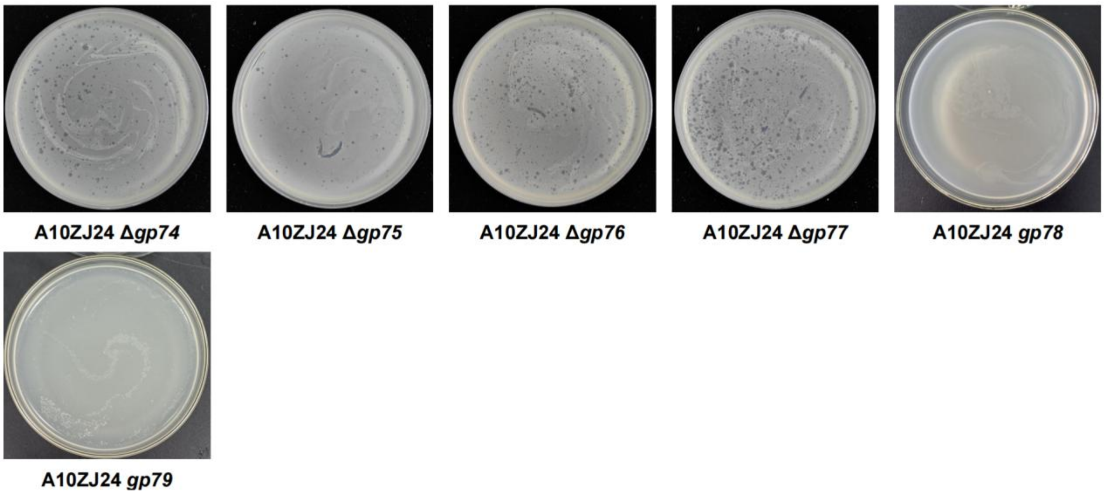

Appendix Figure S1, related to Figure 1. Quantification of plaque-forming units (PFU) following BRED-mediated individual gene knockout in phage A10ZJ24.

Abundant plaque formation by removable phage genes on counter-selection bacterial lawns, in contrast to severely attenuated infectivity of undeletable genes.

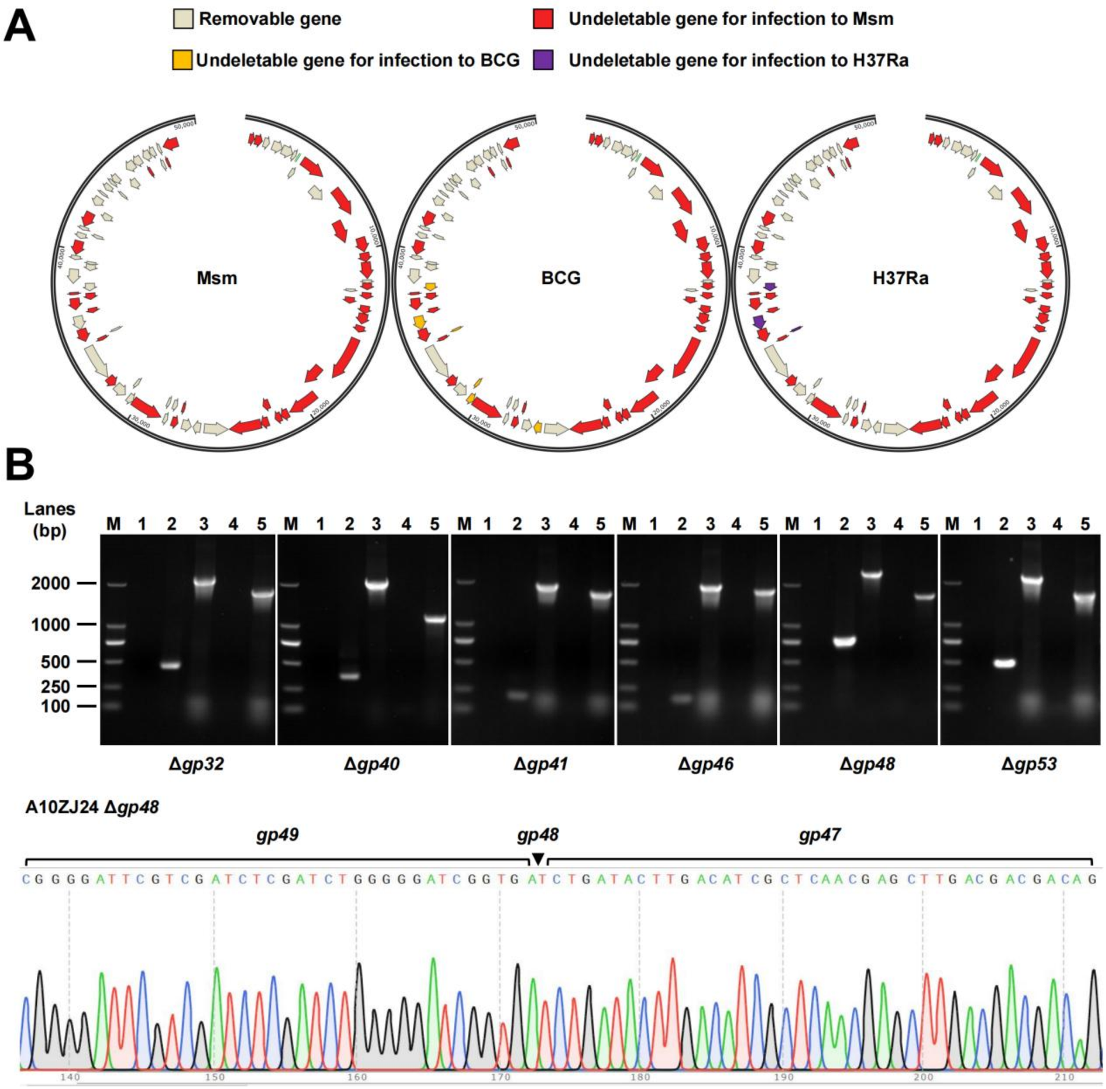

Appendix Figure S2, related to Figure 2. Assays for the phage gene importance for the infection of A10ZJ24 to different host.

A. A map illustrating the key genetic determinants of phage A10ZJ24 infection efficiency in three hosts. Msm represents *M. smegmatis* mc^2 155 strain. BCG represents *M. bovis* BCG strain. H37Ra represents *M. tuberculosis* H37Ra strain.

B. PCR assays for confirming the gene knockout in the genome of phage A10ZJ24.

Top panel: Identification of the deletion of phage A10ZJ24 by PCR. Lane 1 represents the amplified product of target gene using ddH$_2$O as a negative control. Lane 2 represents the amplified product of target gene with the wild-type phage genome as a positive control. Lane 3 represents the amplified product of the flanking regions from wild-type genome. Lane 4 represents the amplified product of target gene with gene-deletion phage genome. Lane 5 represents the amplified product of the flanking regions from deletion mutant.

Bottom panel: Sequencing chromatogram of adjacent *gp48* region in A10ZJ24Δ*gp48* mutant.

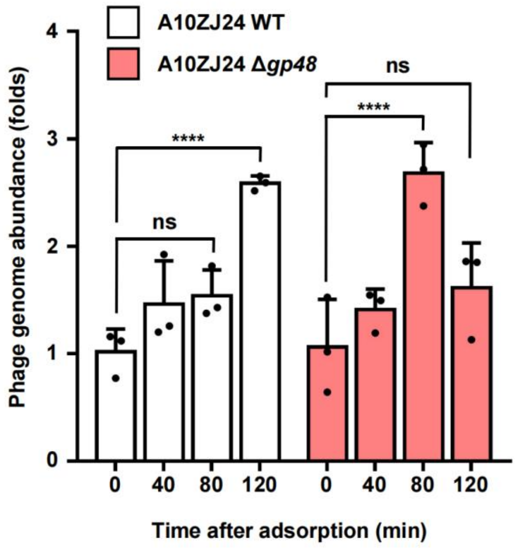

Appendix Figure S3, related to Figure 3. Comparative analysis of the genome abundance between wild-type and mutant phage A10ZJ24 following infection of *M. smegmatis* strain.

All data are presented as the mean ± SD (n = 3, biological replicates) . The P values were calculated by two-way ANOVA using GraphPad Prism v7.0. Asterisks denote significant differences (ns = non-significant, **** $P < 0.0001$) between two groups. Genome abundance of A10ZJ24 WT at 40 mins: $p = 0.2226$, at 80 mins: $p = 0.1295$, at 120 mins: $p < 0.0001$; Genome abundance of A10ZJ24 Δ*gp48* at 40 mins: $p =$ 0.3958, at 80 mins: $p < 0.0001$, at 120 mins: $p = 0.1074$.

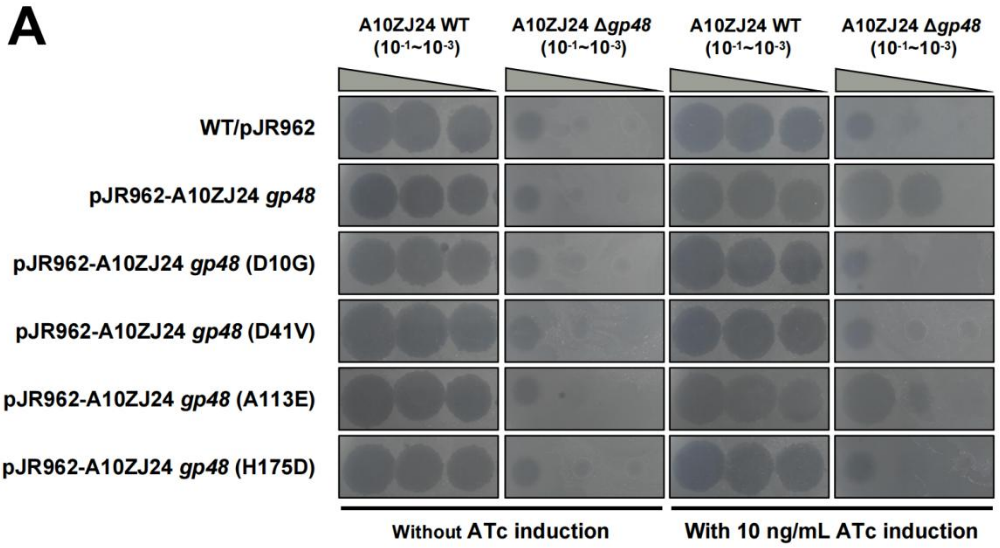

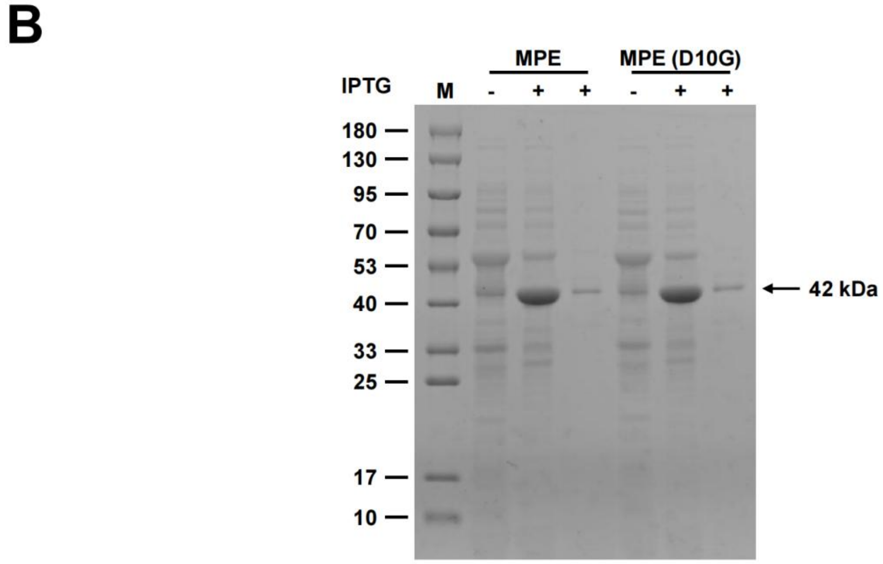

Appendix Figure S4, related to Figure 4. Comparative analysis of the plaque-forming efficiency between A10ZJ24 and its *gp48*-deletion mutant.

A. Comparative analysis of the plaque-forming efficiency of A10ZJ24 Δ*gp48* on the lawns of *M. tuberculosis* H37Ra expressing *gp48* single-point mutants ((D10G/ D41G/A113E/H175D) with induction of 0.1 mg/mL ATc. WT/pJR962 represents the wild-type strain harboring an empty vector pJR962 as a negative control.

B. SDS-PAGE analysis of A10ZJ24 MPE and its mutant protein. Both proteins were expressed as His-SUMO-tagged fusions and induced with 0.5 M IPTG.

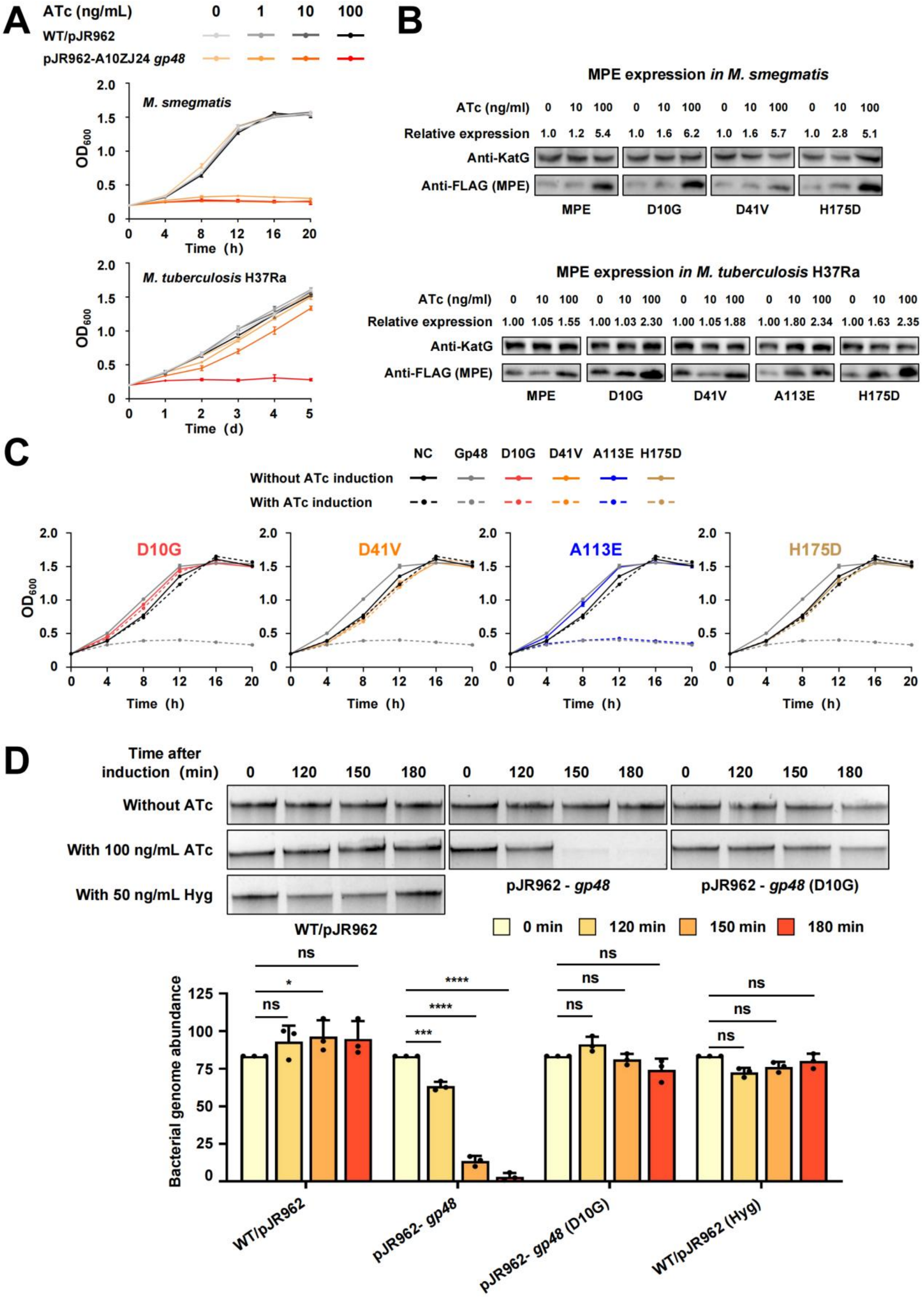
A
ATc (ng/mL) 0 1 10 100
WT/pJR962
pJR962-A10ZJ24 gp48
M. smegmatis
OD600
Time (h)
M. tuberculosis H37Ra
Time (d)
B
MPE expression in M. smegmatis
ATc (ng/ml) 0 10 100 0 10 100 0 10 100 0 10 100
Relative expression 1.0 1.2 5.4 1.0 1.6 6.2 1.0 1.6 5.7 1.0 2.8 5.1
Anti-KatG
Anti-FLAG (MPE)
MPE D10G D41V H175D
MPE expression in M. tuberculosis H37Ra
ATc (ng/ml) 0 10 100 0 10 100 0 10 100 0 10 100 0 10 100
Relative expression 1.00 1.05 1.55 1.00 1.03 2.30 1.00 1.05 1.88 1.00 1.80 2.34 1.00 1.63 2.35
Anti-KatG
Anti-FLAG (MPE)
MPE D10G D41V A113E H175D
C
NC Gp48 D10G D41V A113E H175D
Without ATc induction
With ATc induction
D10G D41V A113E H175D
OD600
Time (h)
D
Time after induction (min) 0 120 150 180 0 120 150 180 0 120 150 180
Without ATc
With 100 ng/mL ATc
With 50 ng/mL Hyg
WT/pJR962
pJR962 - gp48
pJR962 - gp48 (D10G)
0 min 120 min 150 min 180 min
ns * **** *** ns
Bacterial genome abundance
WT/pJR962
pJR962- gp48
pJR962- gp48 (D10G)
WT/pJR962 (Hyg)

Appendix Figure S5, related to Figure 5. A10ZJ24 *gp48* expression is toxic to mycobacteria and disrupts the genomic integrity of *M. smegmatis* strain.

A. Impact of A10ZJ24 phage *gp48* expression on the growth of *M. smegmatis* mc^2 155 and *M. tuberculosis* H37Ra. WT/pJR962 represents the wild-type strain harboring empty vector pJR962. pJR962-A10ZJ24 *gp48* represents the ATc-induced strain for *gp48* expression. All data are presented as the mean ± SD (*n* = 3, biological replicates) .

B. Western blot analysis of MPE and KatG in *M. smegmatis* mc^2 155 and *M. tuberculosis* H37Ra. FLAG-tagged MPE (anti-FLAG) and endogenous KatG (anti-KatG, loading control) expression profiles in *M. smegmatis* mc^2 155 and *M. tuberculosis* H37Ra were evaluated under 0, 0.01, and 0.1 mg/mL ATc induction. MPE, D10G, D41V, A113E, H175D represent pJR962-*gp48-flag*, pJR962-*gp48* (D10G) -*flag*, pJR962-*gp48* (D41V) -*flag*, pJR962-*gp48* (A113E) -*flag*, pJR962-*gp48* (H175D) -*flag* strains, respectively. In each panel, the normalized value of the first sample was set to 1, and the values of other samples were represented by the folds change of their normalized values relative to the first sample.

C. Effect of expressions of various mutant *gp48* genes on the growth of *M. smegmatis* mc^2 155. NC represents the strain harboring empty vector pJR962. Gp48, D10G, D41V, A113E, and H175D represent the strains harboring pJR962-*gp48*, pJR962-*gp48* (D10G), pJR962-*gp48* (D41G), pJR962-*gp48* (A113E), and pJR962-*gp48* (H175D) expression vector, respectively. All data are presented as the mean ± SD (*n* = 3, biological replicates) .

D. Genomic DNA integrity assessment in *gp48*-expressing *M. smegmatis* strains. Total DNA (200 ng) from ATc-induced *M. smegmatis* expressing Gp48 or D10G mutant was electrophoresed (upper panel). Complete genome abundance was quantified by band intensity analysis (Image Lab software, lower panel). All data are presented as the mean ± SD (*n* = 3, biological replicates) . The *P* values were calculated by two-way ANOVA using GraphPad Prism v7.0. Asterisks denote significant differences (ns = non-significant, * P <0.05, *** P <0.001, **** P < 0.0001) between two groups. Genome abundance of WT/pJR962 at 120 mins: p = 0.1197, at 150 mins: p = 0.0256, at 180 mins: p = 0.0573; Genome abundance of pJR962-*gp48* at 120 mins: p = 0.0006, at 150 mins: p < 0.0001, at 180 mins: p < 0.0001; Genome abundance of pJR962-*gp48* (D10G) at 120 mins: p = 0.2549, at 150 mins: p = 0.9362, at 180 mins: p = 0.1504; Genome abundance of WT/pJR962 (Hyg) at 120 mins: p = 0.0739, at 150 mins: p = 0.3092, at 180 mins: p = 0.8231.

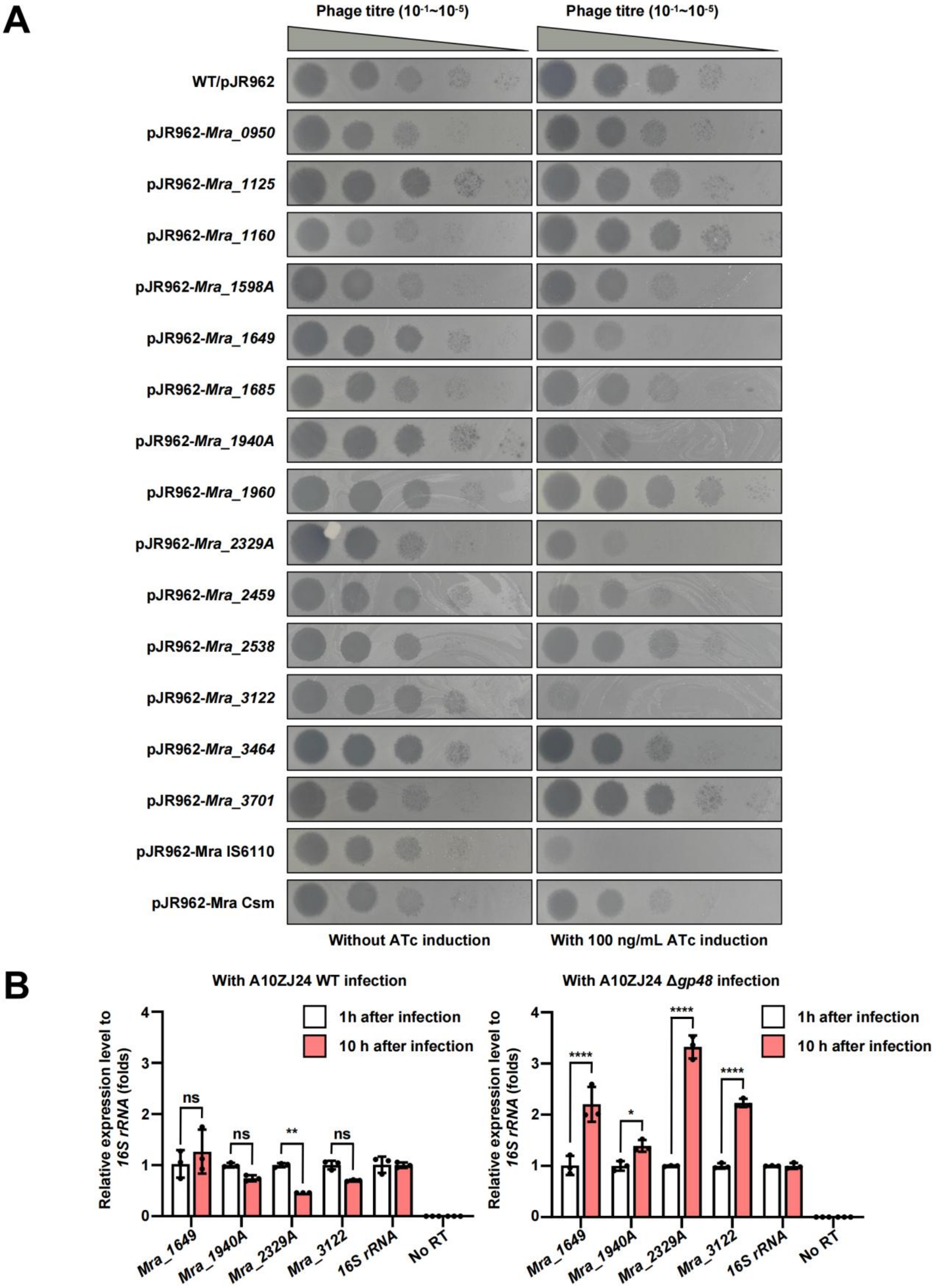
A
Phage titre (10-1~10-5)
Phage titre (10-1~10-5)
WT/pJR962
pJR962-Mra_0950
pJR962-Mra_1125
pJR962-Mra_1160
pJR962-Mra_1598A
pJR962-Mra_1649
pJR962-Mra_1685
pJR962-Mra_1940A
pJR962-Mra_1960
pJR962-Mra_2329A
pJR962-Mra_2459
pJR962-Mra_2538
pJR962-Mra_3122
pJR962-Mra_3464
pJR962-Mra_3701
pJR962-Mra IS6110
pJR962-Mra Csm
Without ATc induction
With 100 ng/mL ATc induction
B
With A10ZJ24 WT infection
With A10ZJ24 Δgp48 infection
Relative expression level to 16S rRNA (folds)
1h after infection
10 h after infection
ns
**
****
*
Mra_1649
Mra_1940A
Mra_2329A
Mra_3122
16S rRNA
No RT
0
1
2
3
4

Appendix Figure S6, related to Figure 6. Assays for the defense activity of anti-phage genes and their transcriptional dynamics post-infection with A10ZJ24.

A. Effect of expressions of anti-phage genes on the plaques-forming efficiency of phage A10ZJ24 on the lawns of *M. smegmatis* mc^2 155. WT/pJR962 represents the wild-type strain harboring empty vector (negative control). pJR962-Mra IS6110 represents ATc-induced strain expressing *Mra_3420*. pJR962-Mra Csm represents co-expression strain of *Mra_2840, Mra_2841,* and *Mra_2842*.

B. Quantitative real-time PCR assays of several anti-phage genes in *M. tuberculosis* H37Ra at 1 and 10 hours post-infection (hpi). Left panel: Wild-type A10ZJ24 infection. Right panel: A10ZJ24 *Δgp48* mutant phage infection. Data normalized to endogenous *16S rRNA* (ΔΔCt method). All data are presented as the mean ± SD (*n* = 3, biological replicates) . The *P* values were calculated by two-way ANOVA using GraphPad Prism v7.0. Asterisks denote significant differences (ns = non-significant, ***P* <0.01, **** *P* < 0.0001) between two groups. The expression level of *Mra_1649* 10 h after infection with A10ZJ24 WT: p = 0.3678, *Mra_1940A*: p = 0.2993, *Mra_2329A*: p = 0.0019, *Mra_3122*: p = 0.1573; the expression level of *Mra_1649* 10 h after infection with A10ZJ24 Δ*gp48*: p < 0.0001, *Mra_1940A*: p = 0.0138, *Mra_2329A*: p < 0.0001, *Mra_3122*: p < 0.0001.

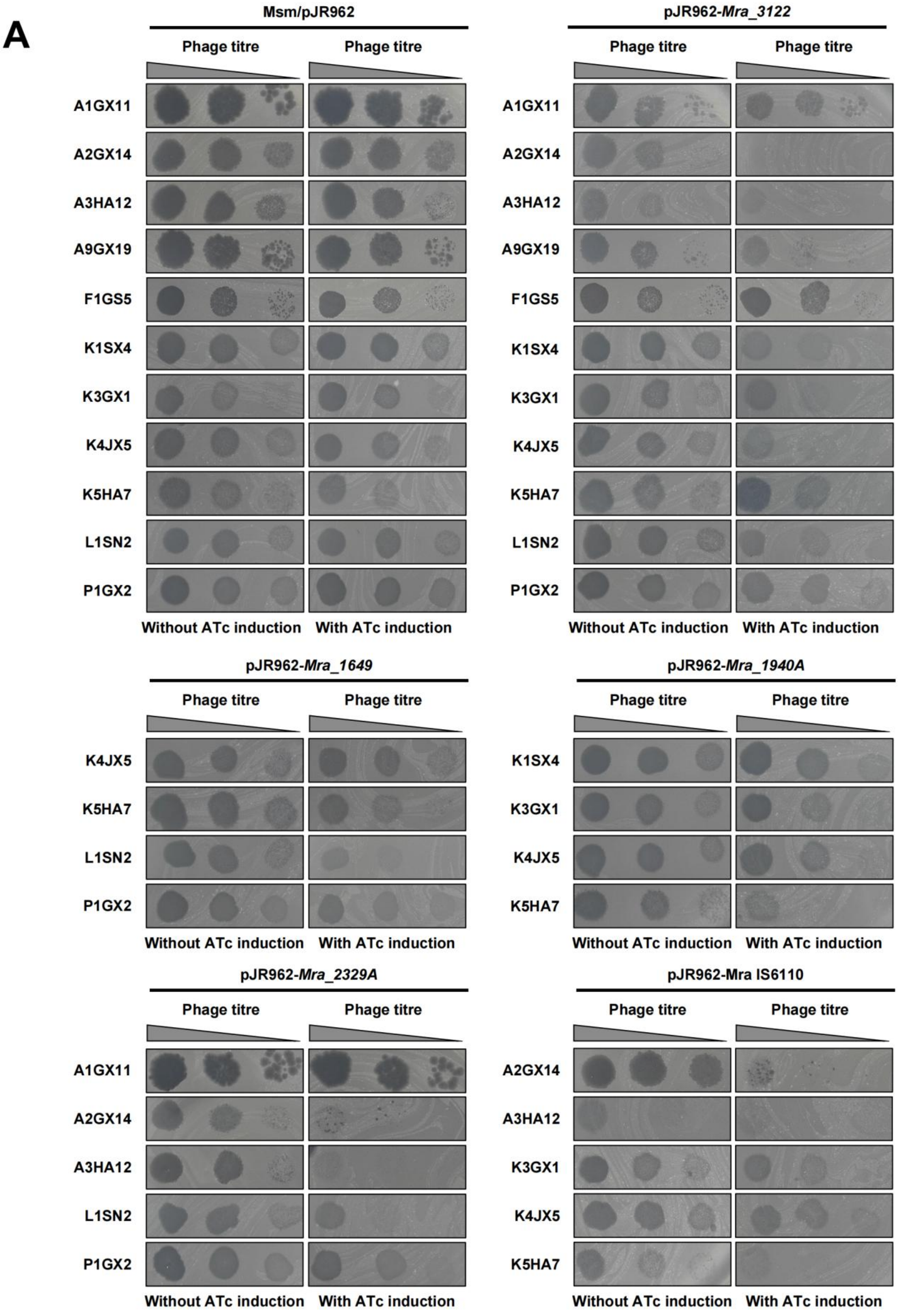
A
Msm/pJR962
pJR962-Mra_3122
pJR962-Mra_1649
pJR962-Mra_1940A
pJR962-Mra_2329A
pJR962-Mra IS6110
Phage titre
A1GX11
A2GX14
A3HA12
A9GX19
F1GS5
K1SX4
K3GX1
K4JX5
K5HA7
L1SN2
P1GX2
Without ATc induction
With ATc induction

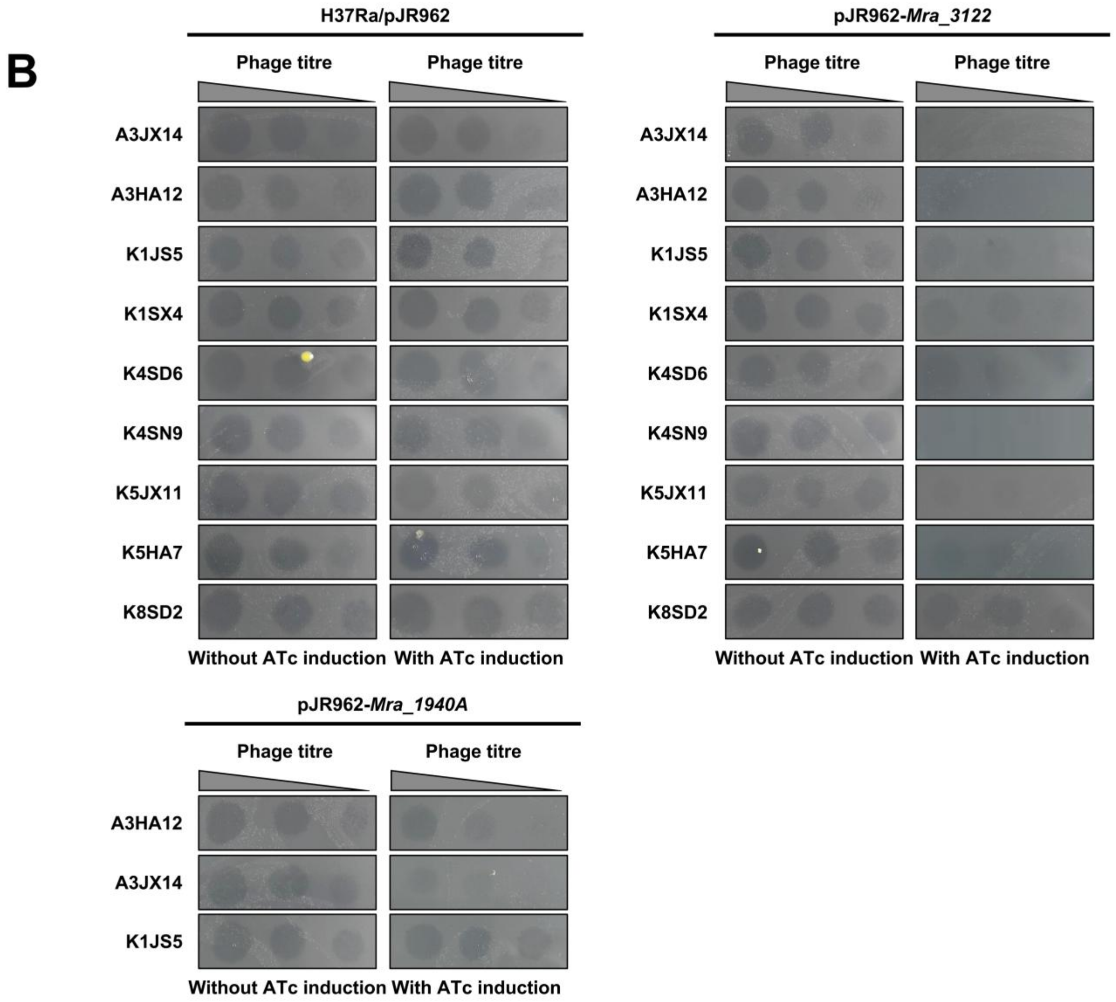

Appendix Figure S7, related to Figure 7. Assays for defense activity of several anti-phage genes in *M. tuberculosis* H37Ra.

A. Comparative assays for the plaques formation efficiency of phages from different clusters on the lawns of *M. smegmatis*. Phages were spotted onto the lawns of the *M. smegmatis* mc^2 155 strains containing different anti-phage gene expressed vector. Msm/pJR962 represents the wild type *M. smegmatis* mc^2 155 strain containing empty vector pJR962.

B. Comparative assays for plaque formation efficiency of phages from different clusters on the lawns of *M. tuberculosis*. Mra/pJR962 represents the wild-type *M. tuberculosis* H37Ra strain harboring empty vector pJR962.
