# Supplementary figures and images for: A conserved phage phosphoesterase enables evasion of bacterial antiviral immunity (part 1 of 2)
Source: EMBO Rep. 2025 May 29;26(14):3594–613. doi: 10.1038/s44319-025-00488-4 (PMC12287305; doi:10.1038/s44319-025-00488-4)

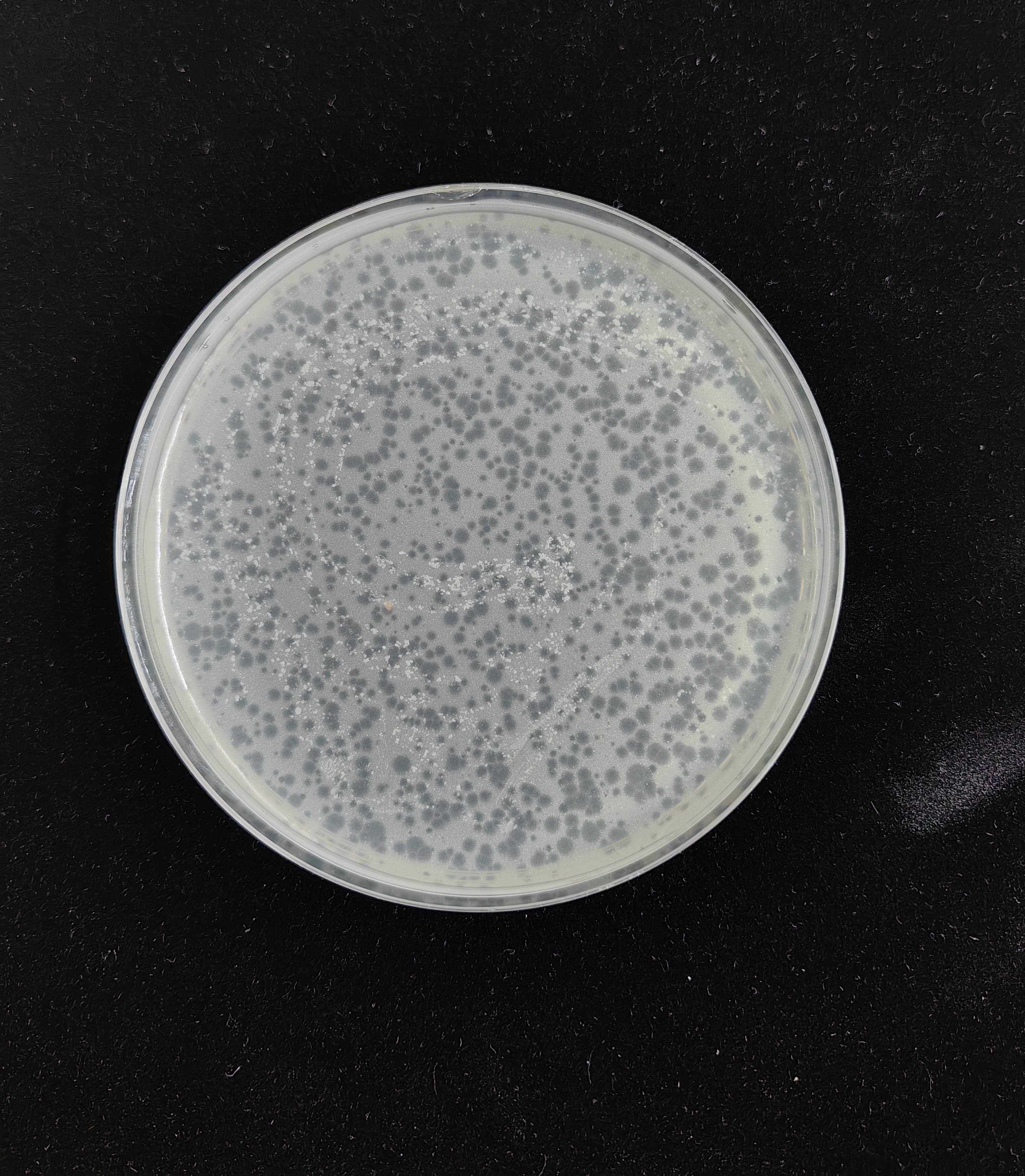

Supplement: Supplementary file 3 — Source data Fig. 1 [file 44319_2025_488_MOESM3_ESM.zip › Figure 1/1B/gp48.tiff]

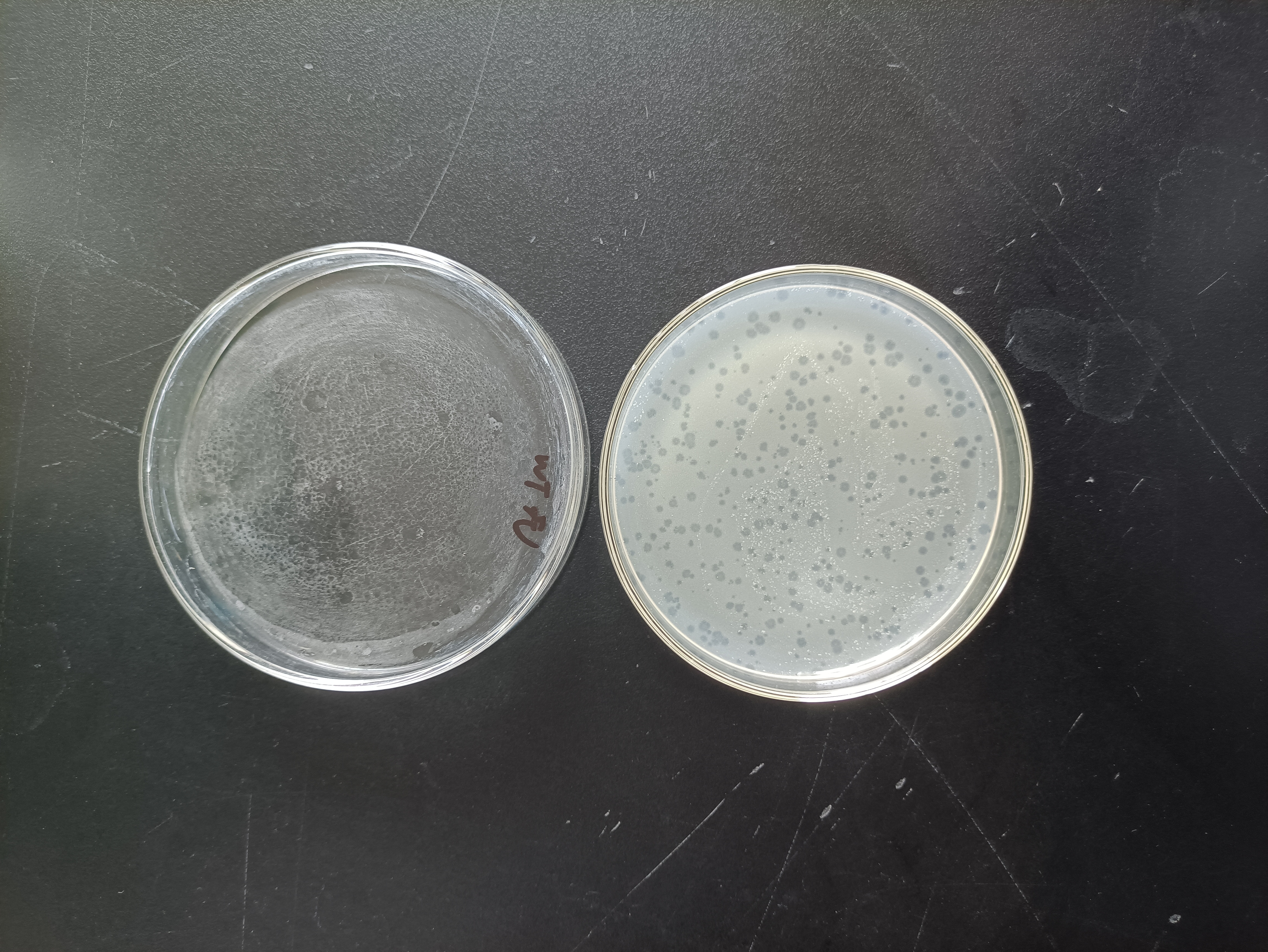

Supplement: Supplementary file 3 — Source data Fig. 1 [file 44319_2025_488_MOESM3_ESM.zip › Figure 1/1B/negative control.tiff]

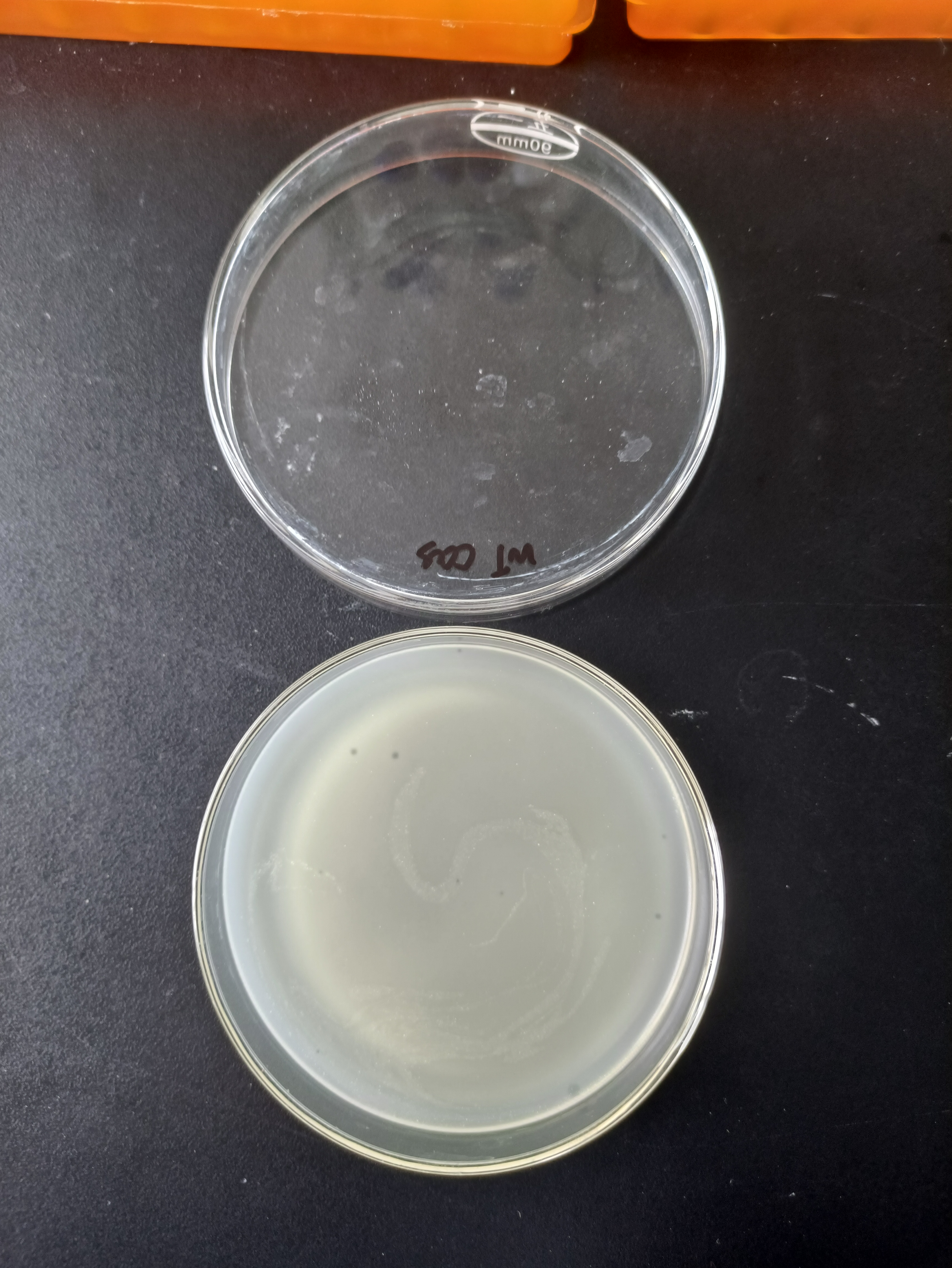

Supplement: Supplementary file 3 — Source data Fig. 1 [file 44319_2025_488_MOESM3_ESM.zip › Figure 1/1B/positive control.tiff]

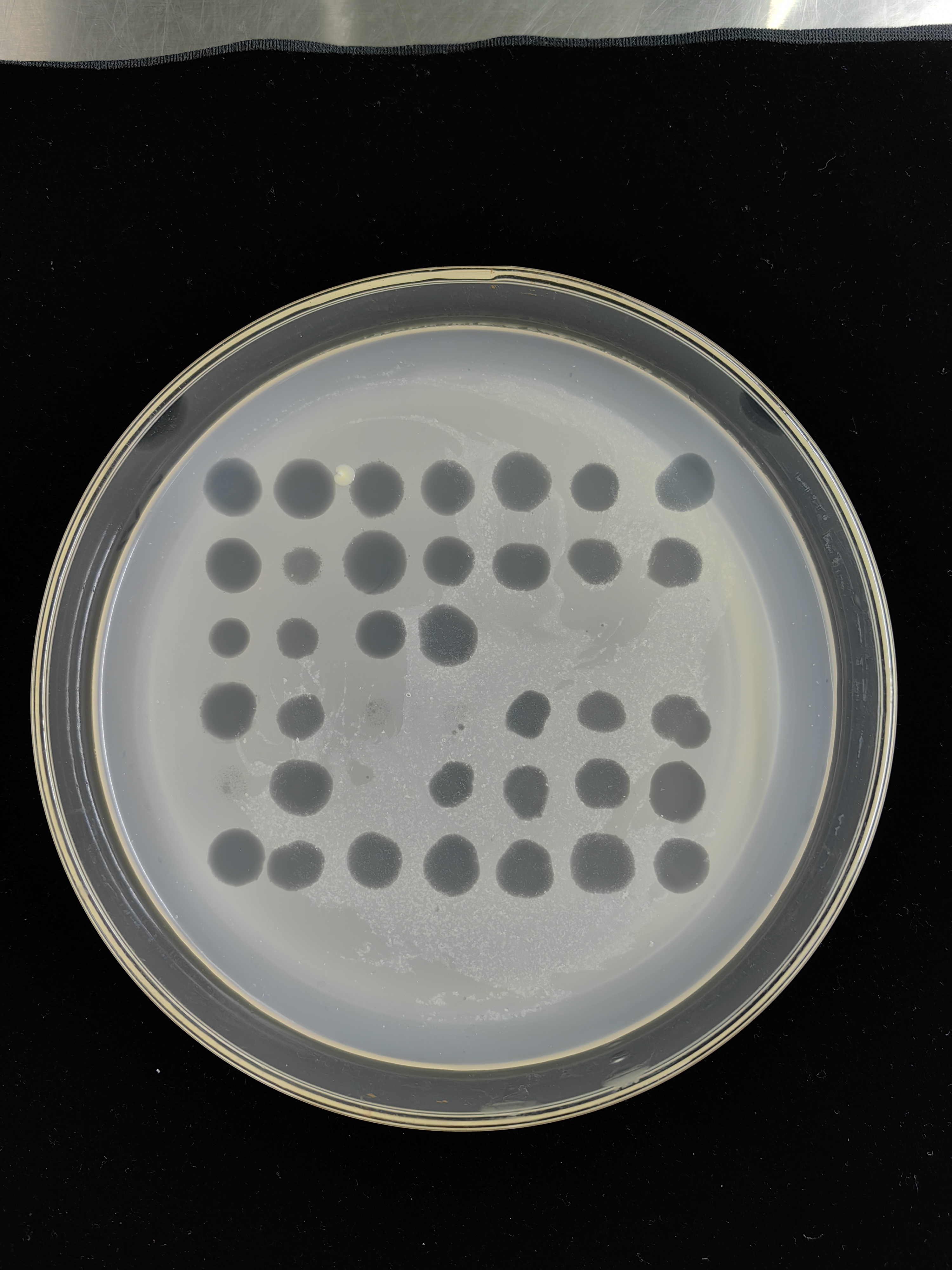

Supplement: Supplementary file 4 — Source data Fig. 2 [file 44319_2025_488_MOESM4_ESM.zip › Figure 2/2A/M. bovis BCG.tiff]

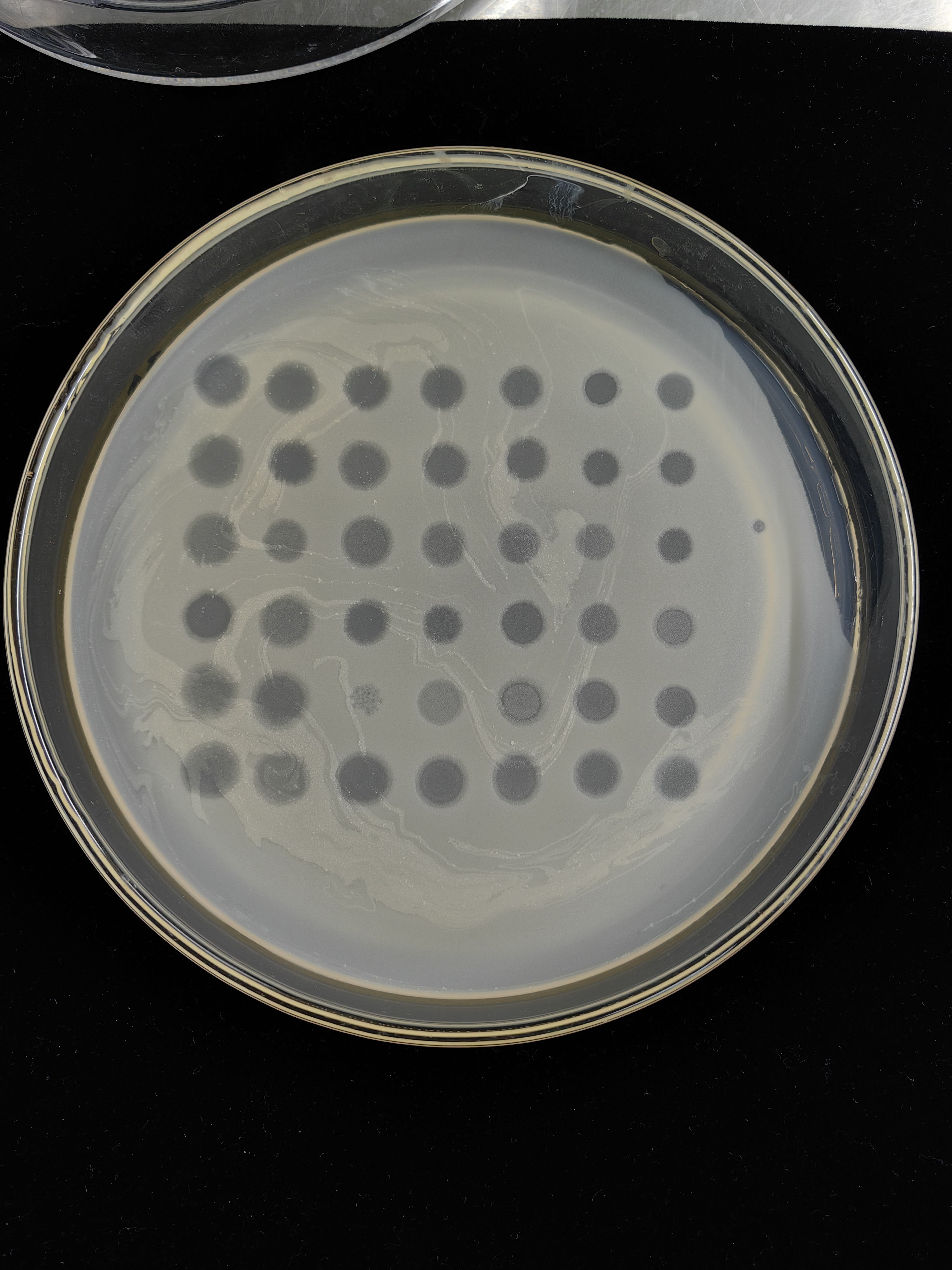

Supplement: Supplementary file 4 — Source data Fig. 2 [file 44319_2025_488_MOESM4_ESM.zip › Figure 2/2A/M. smegmatis.tiff]

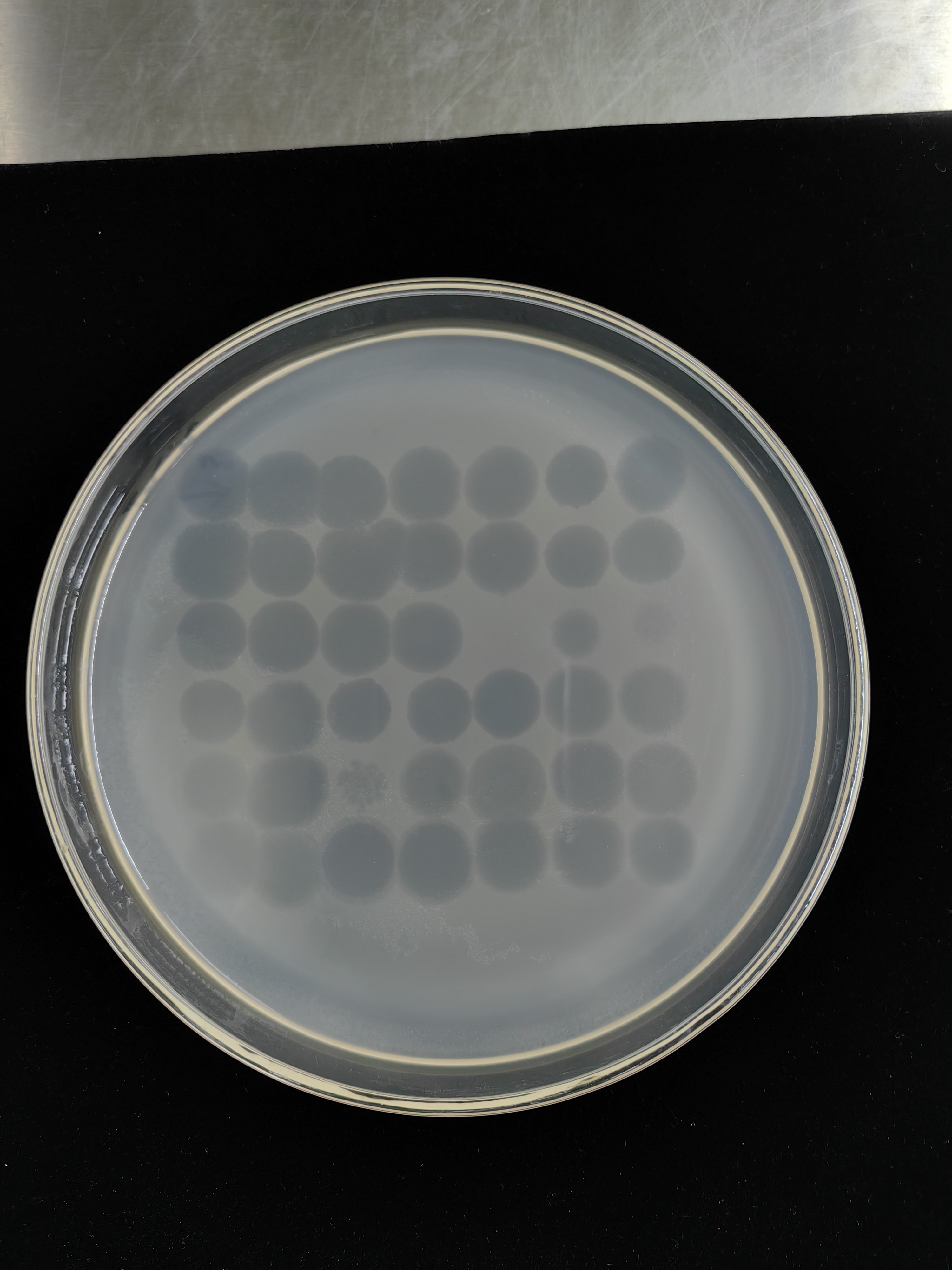

Supplement: Supplementary file 4 — Source data Fig. 2 [file 44319_2025_488_MOESM4_ESM.zip › Figure 2/2A/M. tuberculosis H37Ra.tiff]

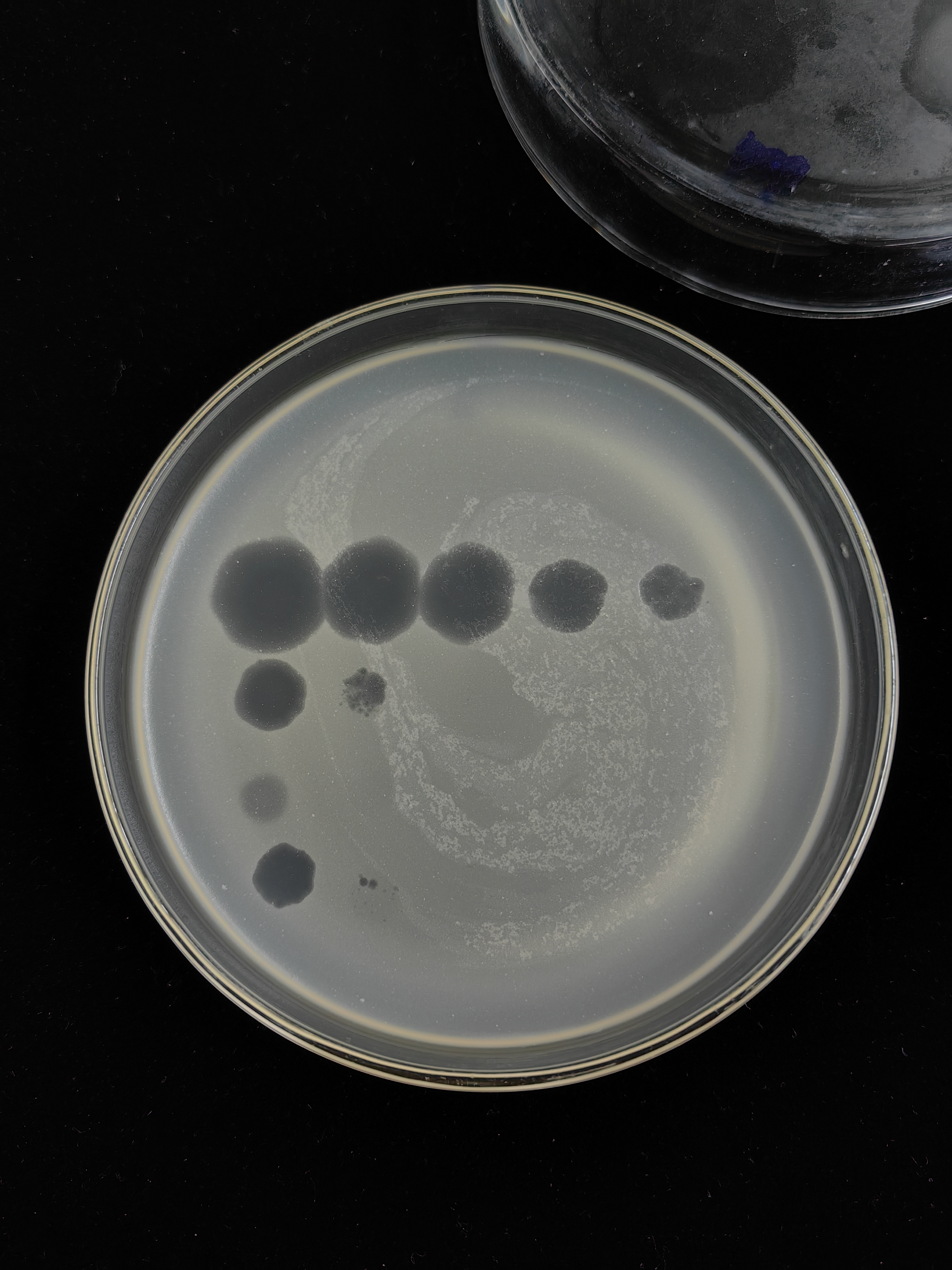

Supplement: Supplementary file 4 — Source data Fig. 2 [file 44319_2025_488_MOESM4_ESM.zip › Figure 2/2B/M. bovis BCG -2.tiff]

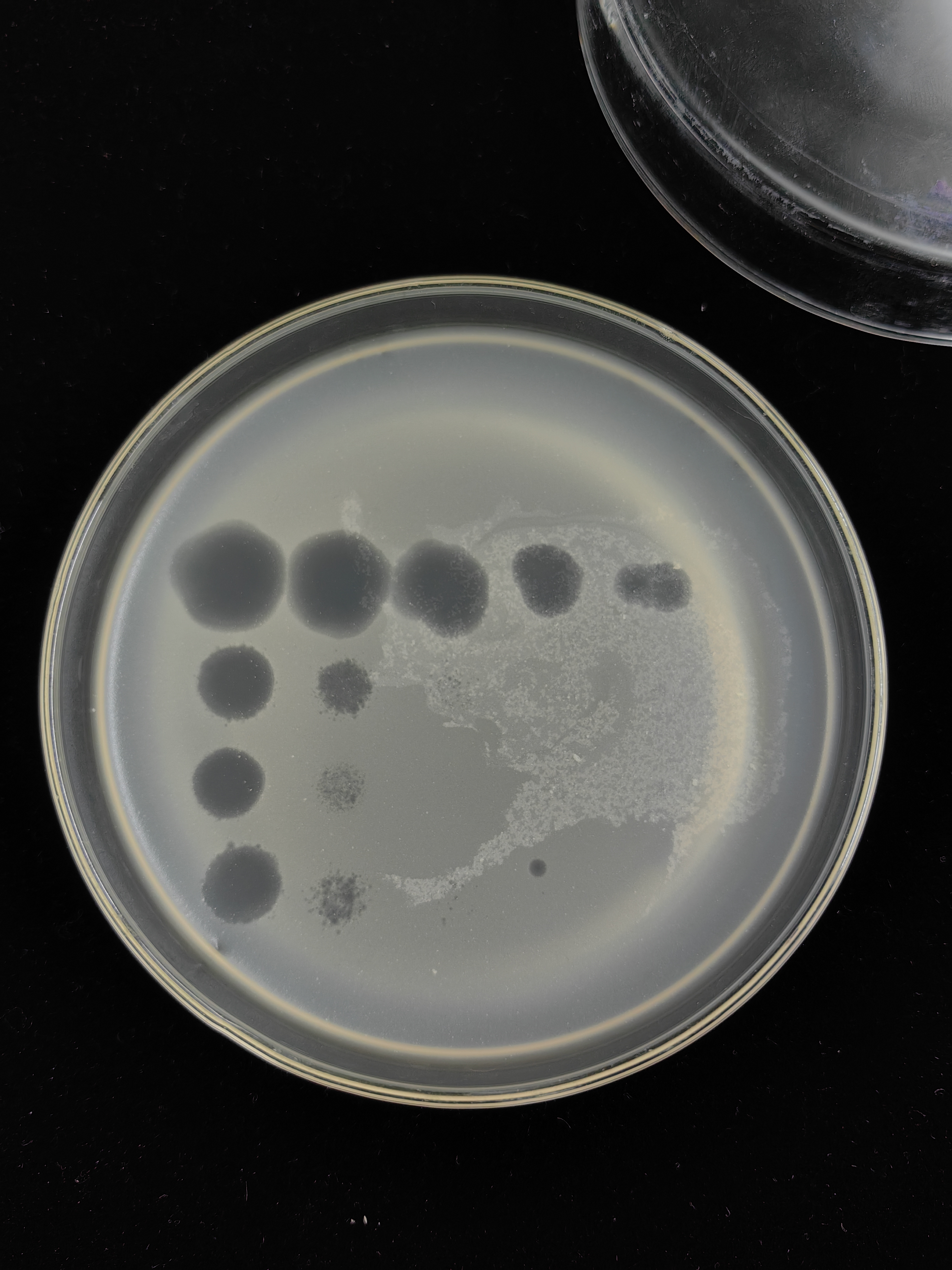

Supplement: Supplementary file 4 — Source data Fig. 2 [file 44319_2025_488_MOESM4_ESM.zip › Figure 2/2B/M. bovis BCG-1.tiff]

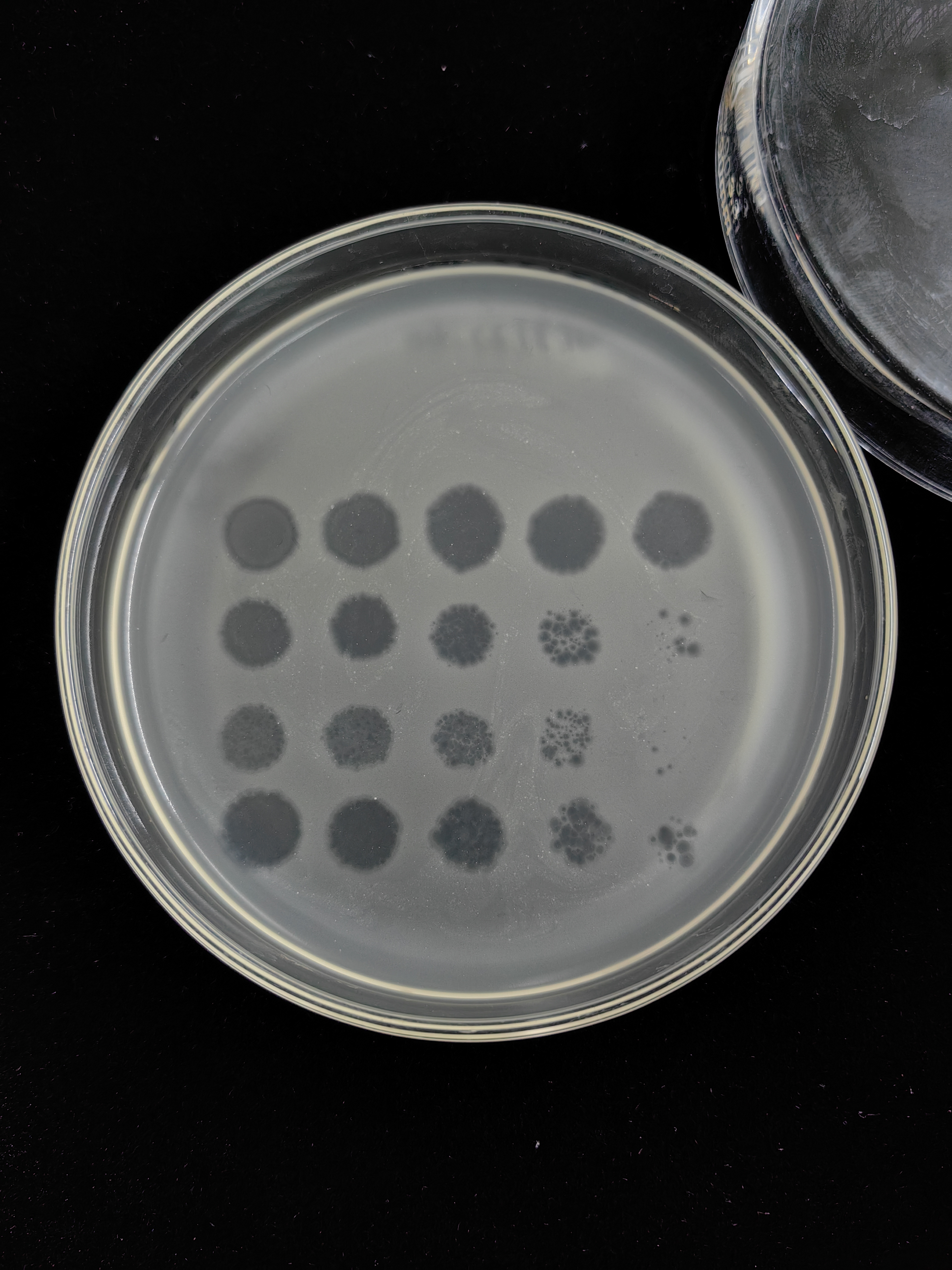

Supplement: Supplementary file 4 — Source data Fig. 2 [file 44319_2025_488_MOESM4_ESM.zip › Figure 2/2B/M. smegmatis -1.tiff]

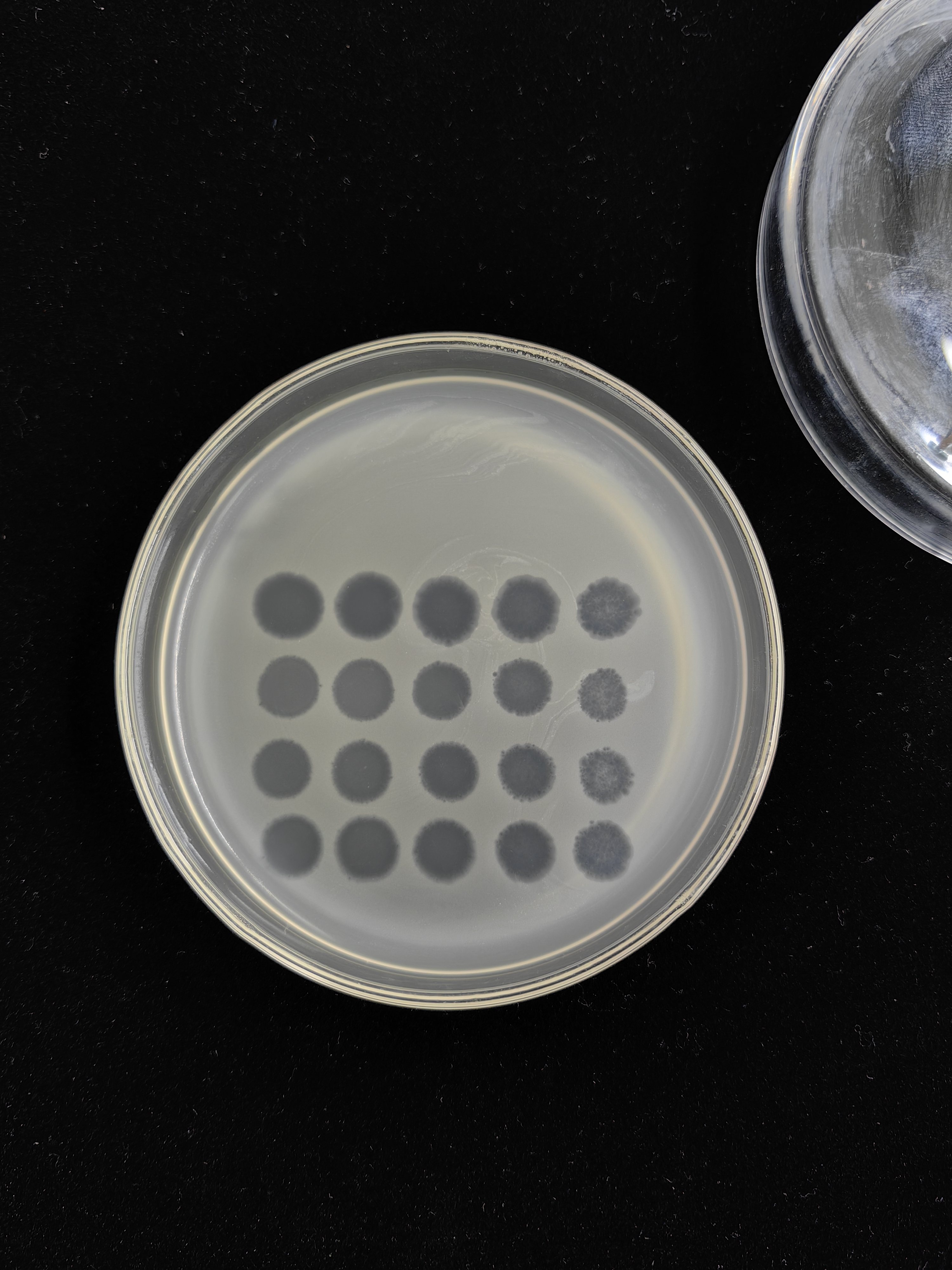

Supplement: Supplementary file 4 — Source data Fig. 2 [file 44319_2025_488_MOESM4_ESM.zip › Figure 2/2B/M. smegmatis -2.tiff]

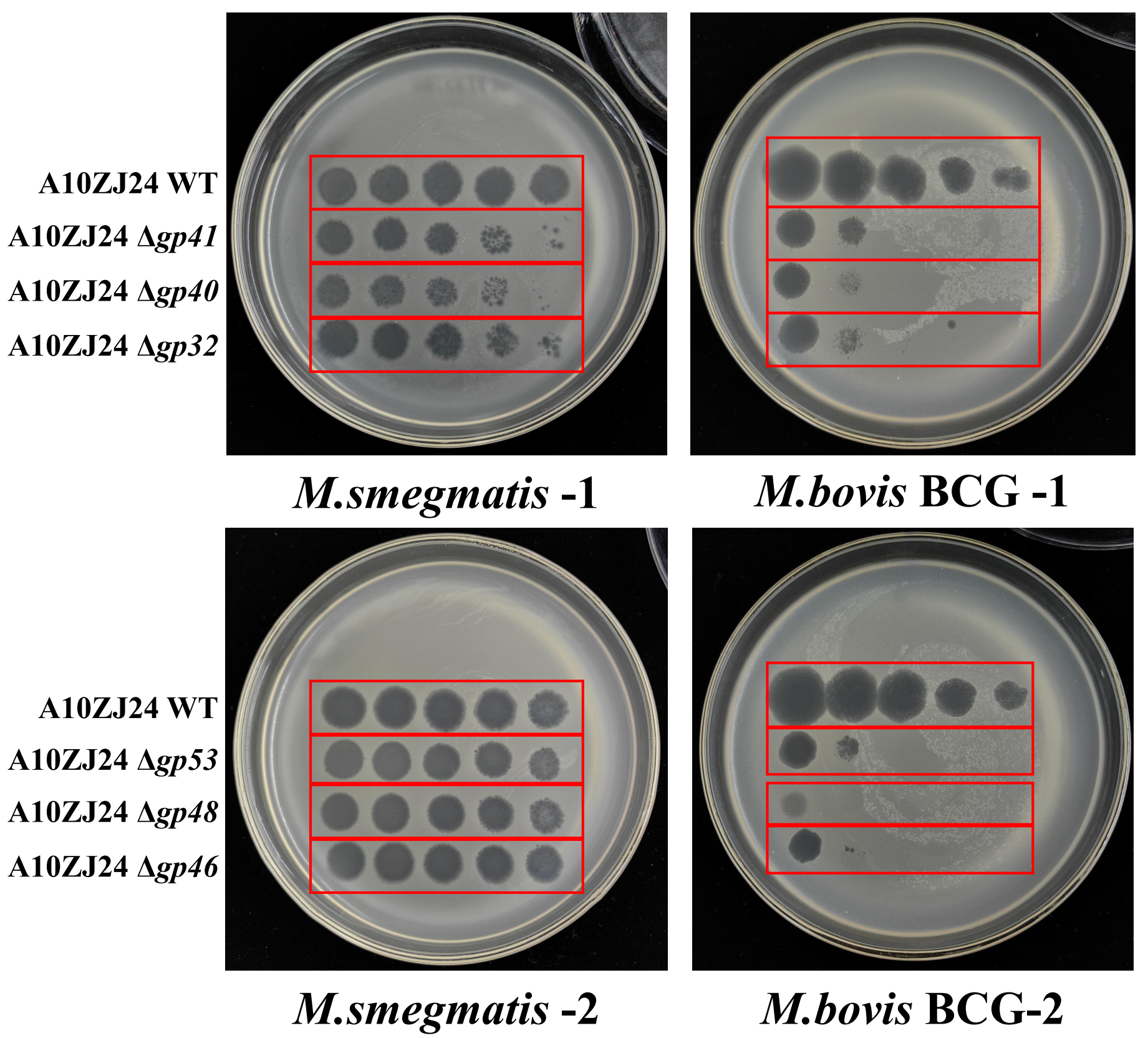

Supplement: Supplementary file 4 — Source data Fig. 2 [file 44319_2025_488_MOESM4_ESM.zip › Figure 2/2B/README.tif]

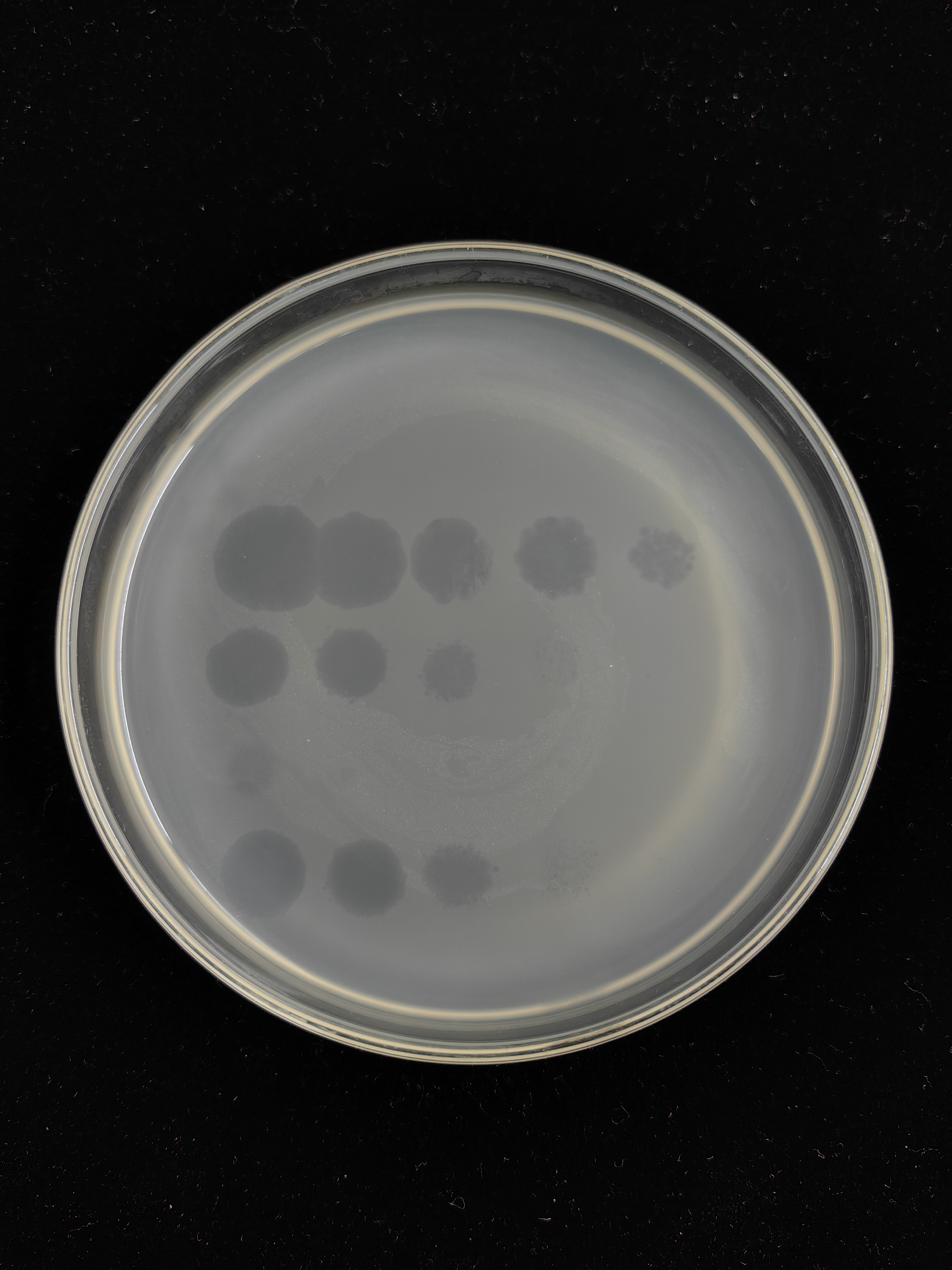

Supplement: Supplementary file 4 — Source data Fig. 2 [file 44319_2025_488_MOESM4_ESM.zip › Figure 2/2C/M. tuberculosis H37Ra 2days.tiff]

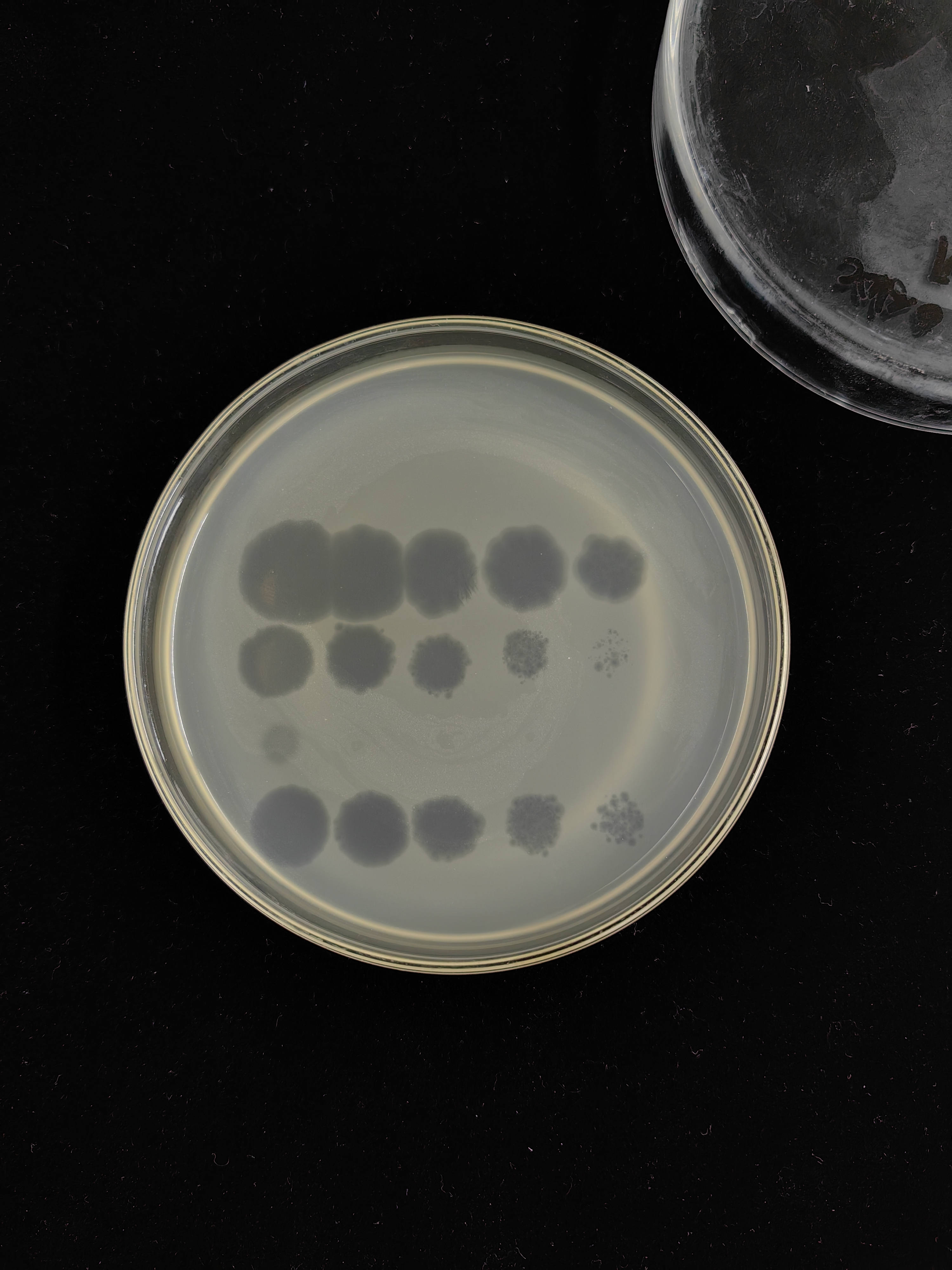

Supplement: Supplementary file 4 — Source data Fig. 2 [file 44319_2025_488_MOESM4_ESM.zip › Figure 2/2C/M. tuberculosis H37Ra 4days.tiff]

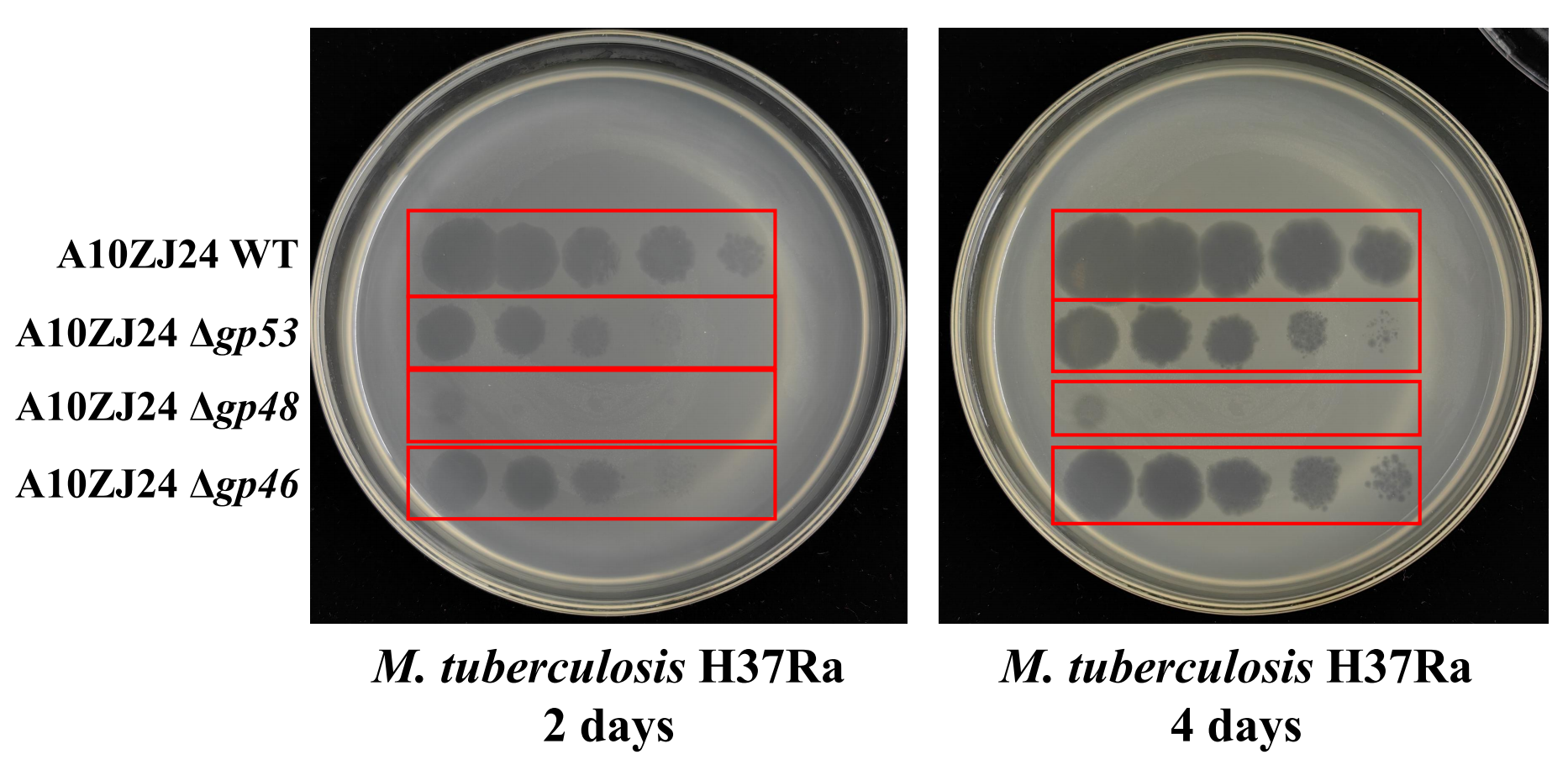

Supplement: Supplementary file 4 — Source data Fig. 2 [file 44319_2025_488_MOESM4_ESM.zip › Figure 2/2C/README.tif]

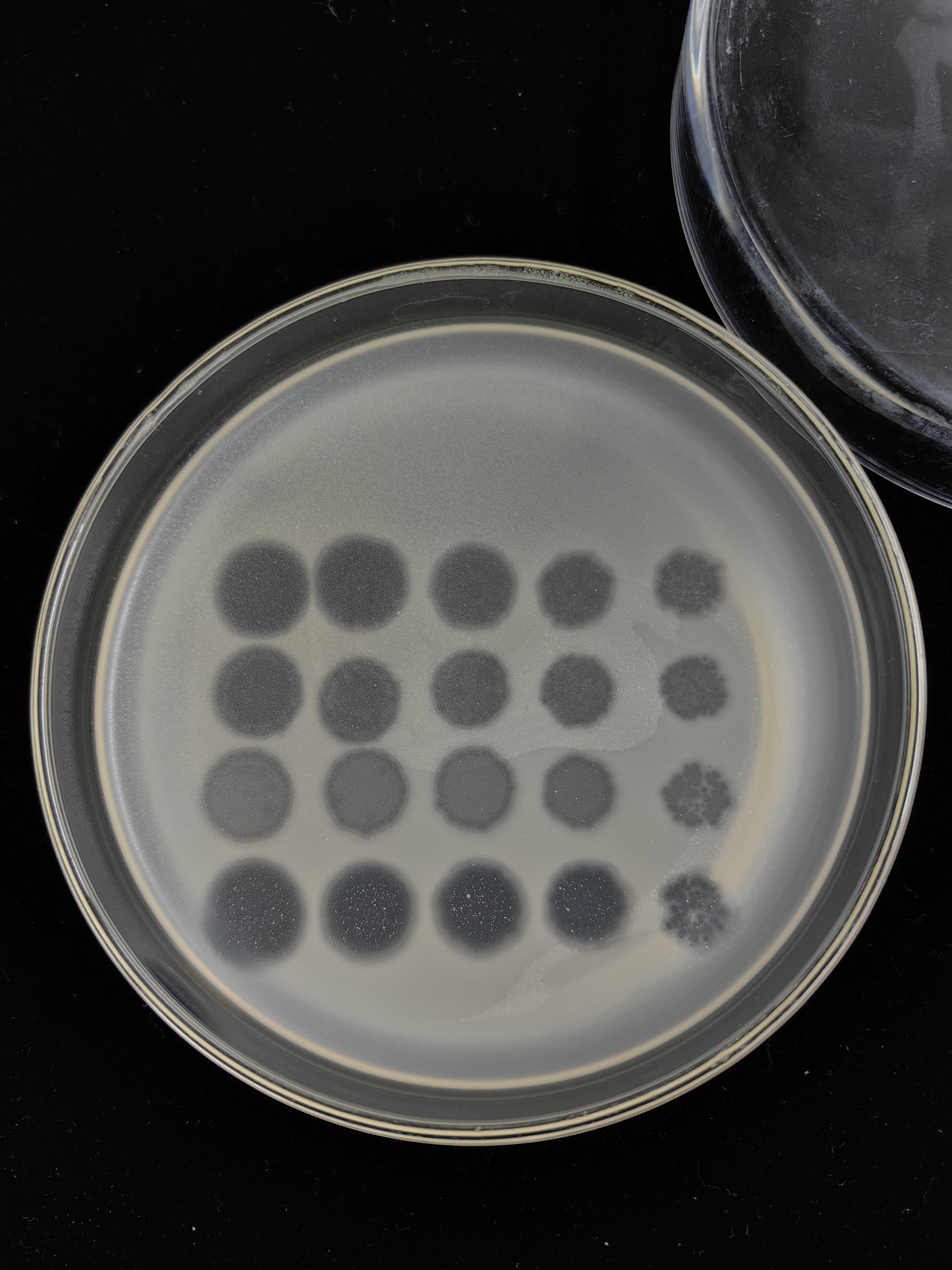

Supplement: Supplementary file 6 — Source data Fig. 4 [file 44319_2025_488_MOESM6_ESM.zip › Figure 4/4B/M. smegmatis.tiff]

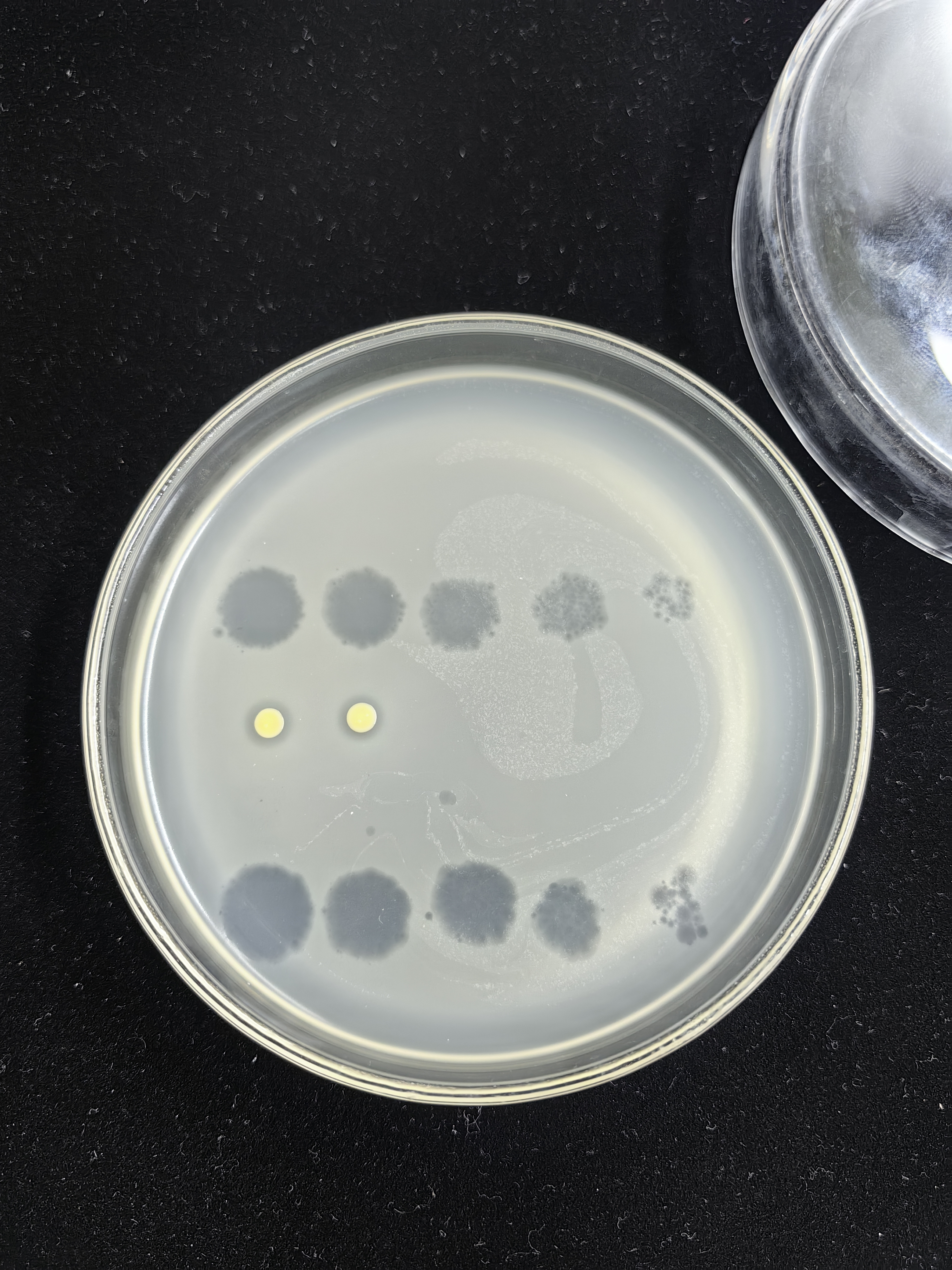

Supplement: Supplementary file 6 — Source data Fig. 4 [file 44319_2025_488_MOESM6_ESM.zip › Figure 4/4B/M. tuberculosis H37Ra-1.tiff]

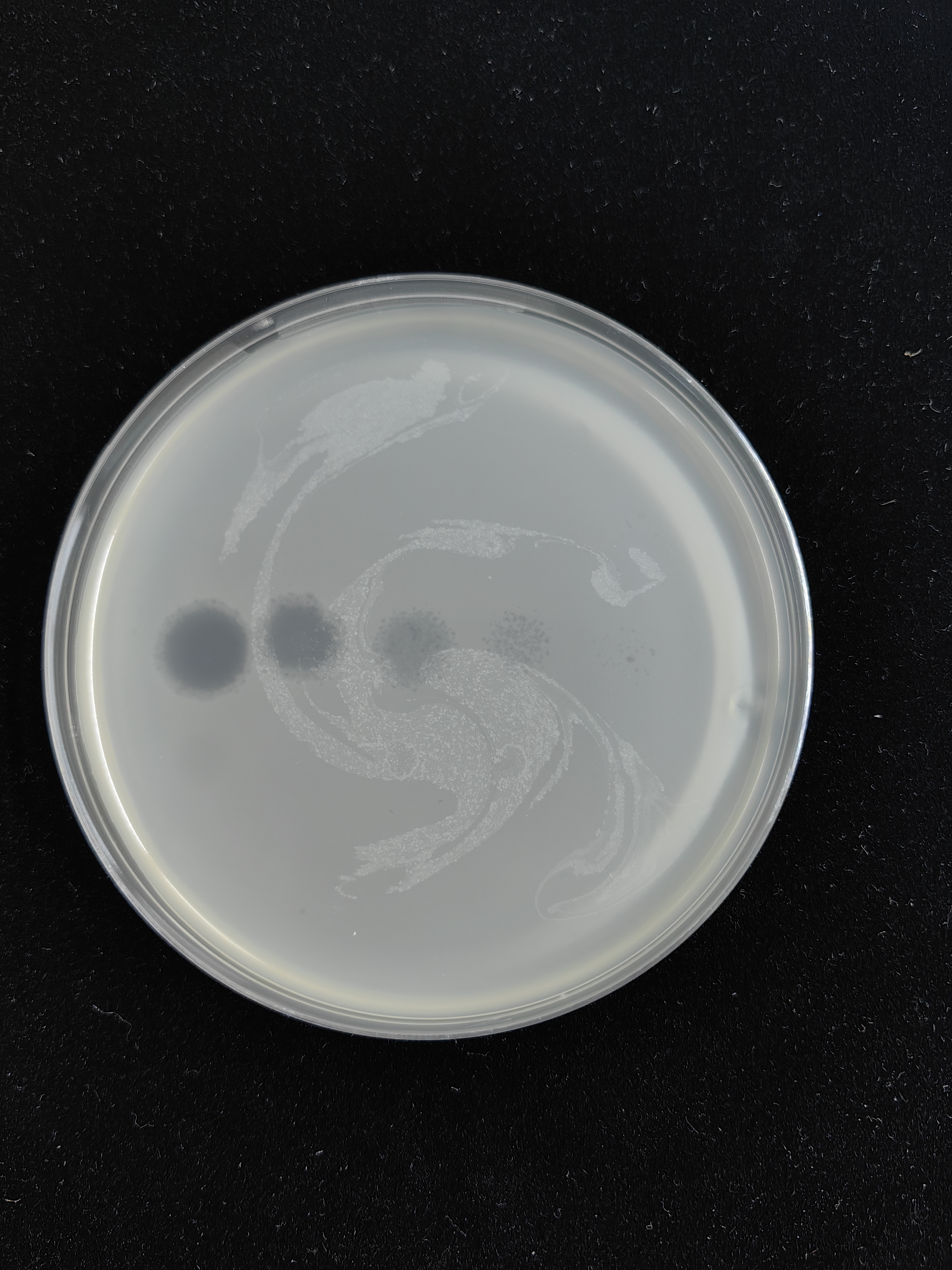

Supplement: Supplementary file 6 — Source data Fig. 4 [file 44319_2025_488_MOESM6_ESM.zip › Figure 4/4B/M. tuberculosis H37Ra-2.tiff]

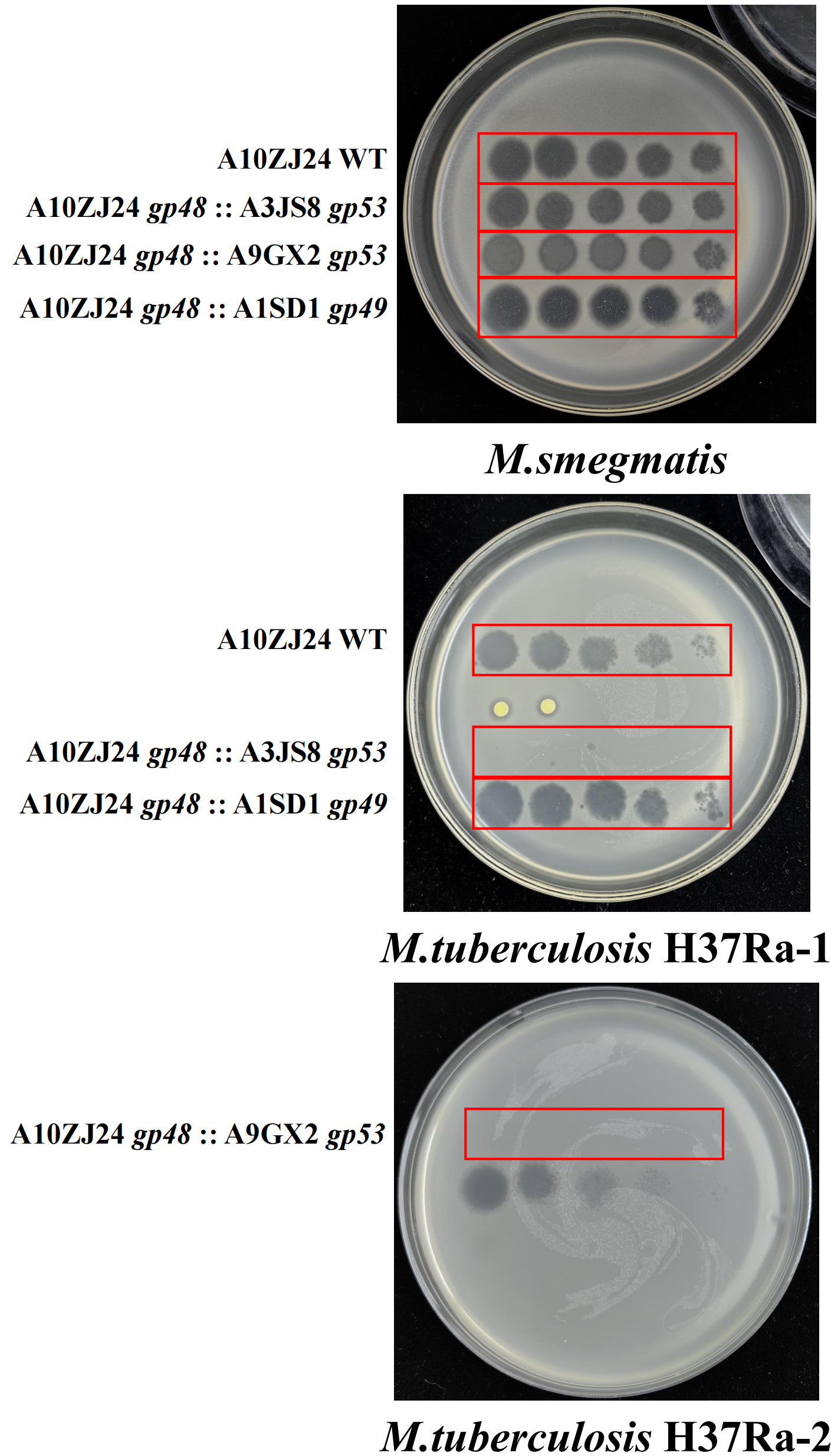

Supplement: Supplementary file 6 — Source data Fig. 4 [file 44319_2025_488_MOESM6_ESM.zip › Figure 4/4B/README.tif]

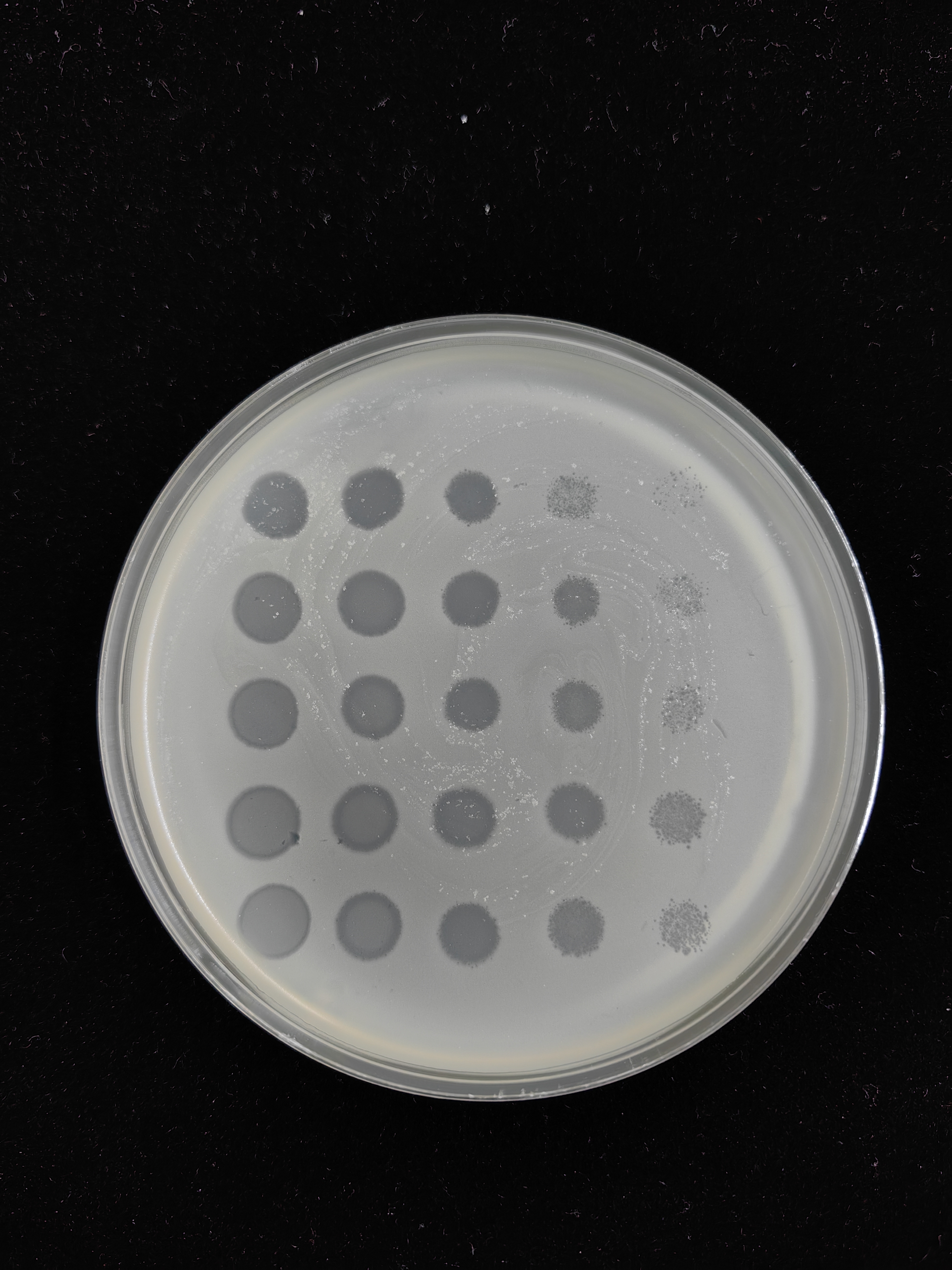

Supplement: Supplementary file 6 — Source data Fig. 4 [file 44319_2025_488_MOESM6_ESM.zip › Figure 4/4C/M. smegmatis.tiff]

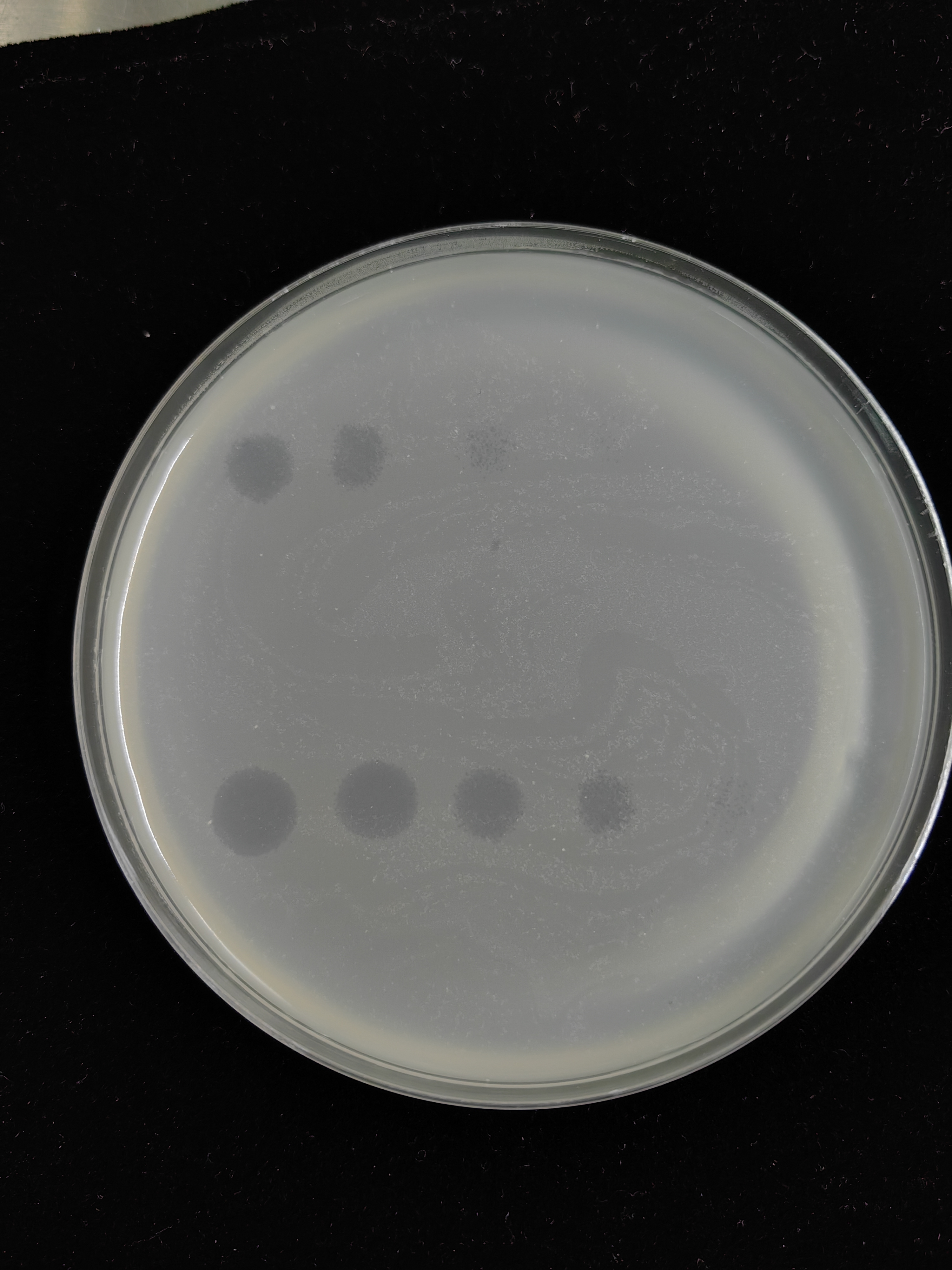

Supplement: Supplementary file 6 — Source data Fig. 4 [file 44319_2025_488_MOESM6_ESM.zip › Figure 4/4C/M. tuberculosis H37Ra-1.tiff]

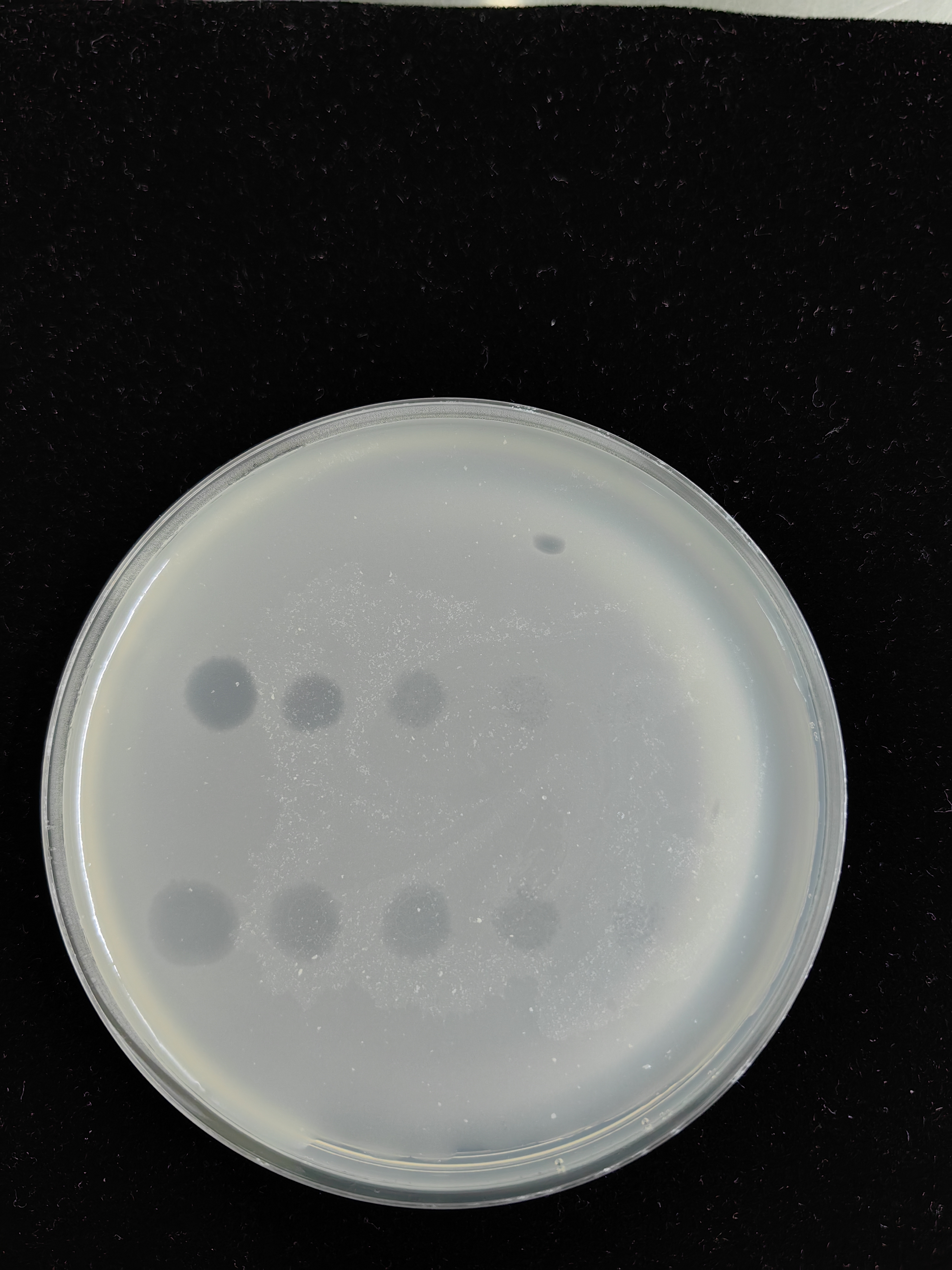

Supplement: Supplementary file 6 — Source data Fig. 4 [file 44319_2025_488_MOESM6_ESM.zip › Figure 4/4C/M. tuberculosis H37Ra-2.tiff]

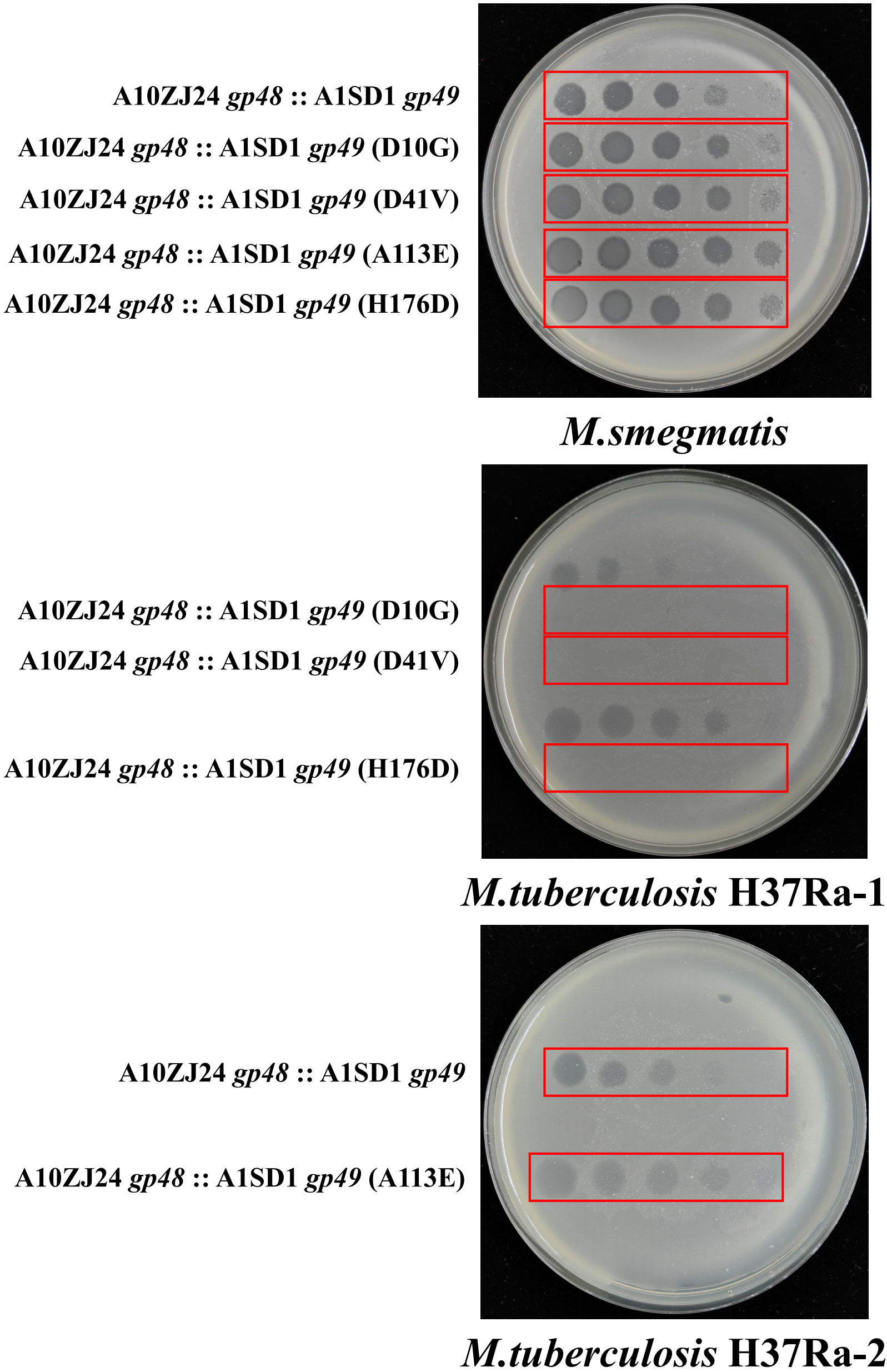

Supplement: Supplementary file 6 — Source data Fig. 4 [file 44319_2025_488_MOESM6_ESM.zip › Figure 4/4C/README.tif]

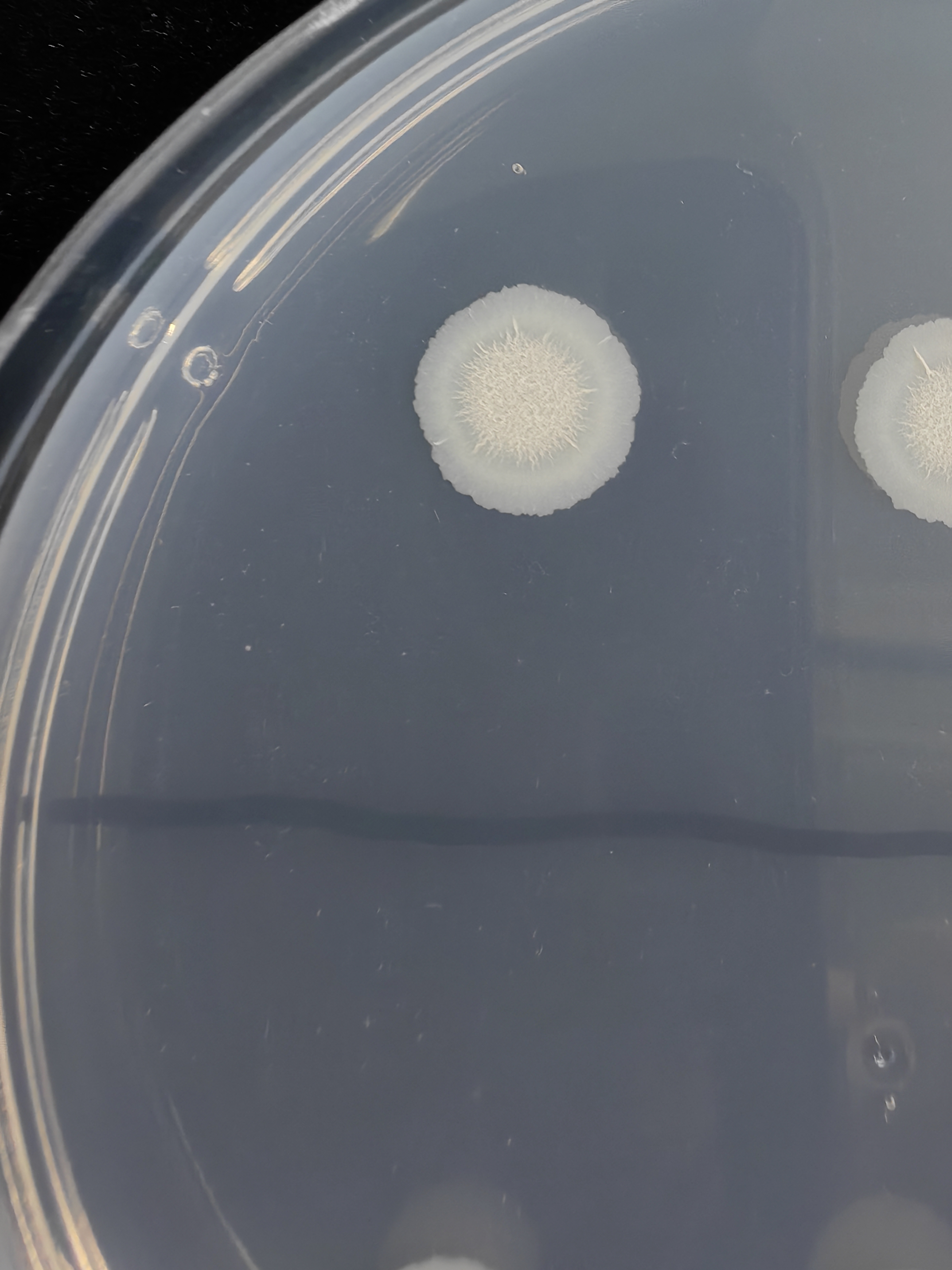

Supplement: Supplementary file 7 — Source data Fig. 5 [file 44319_2025_488_MOESM7_ESM.zip › Figure 5/5A/Mra-pJR962 with ATc induction.tiff]

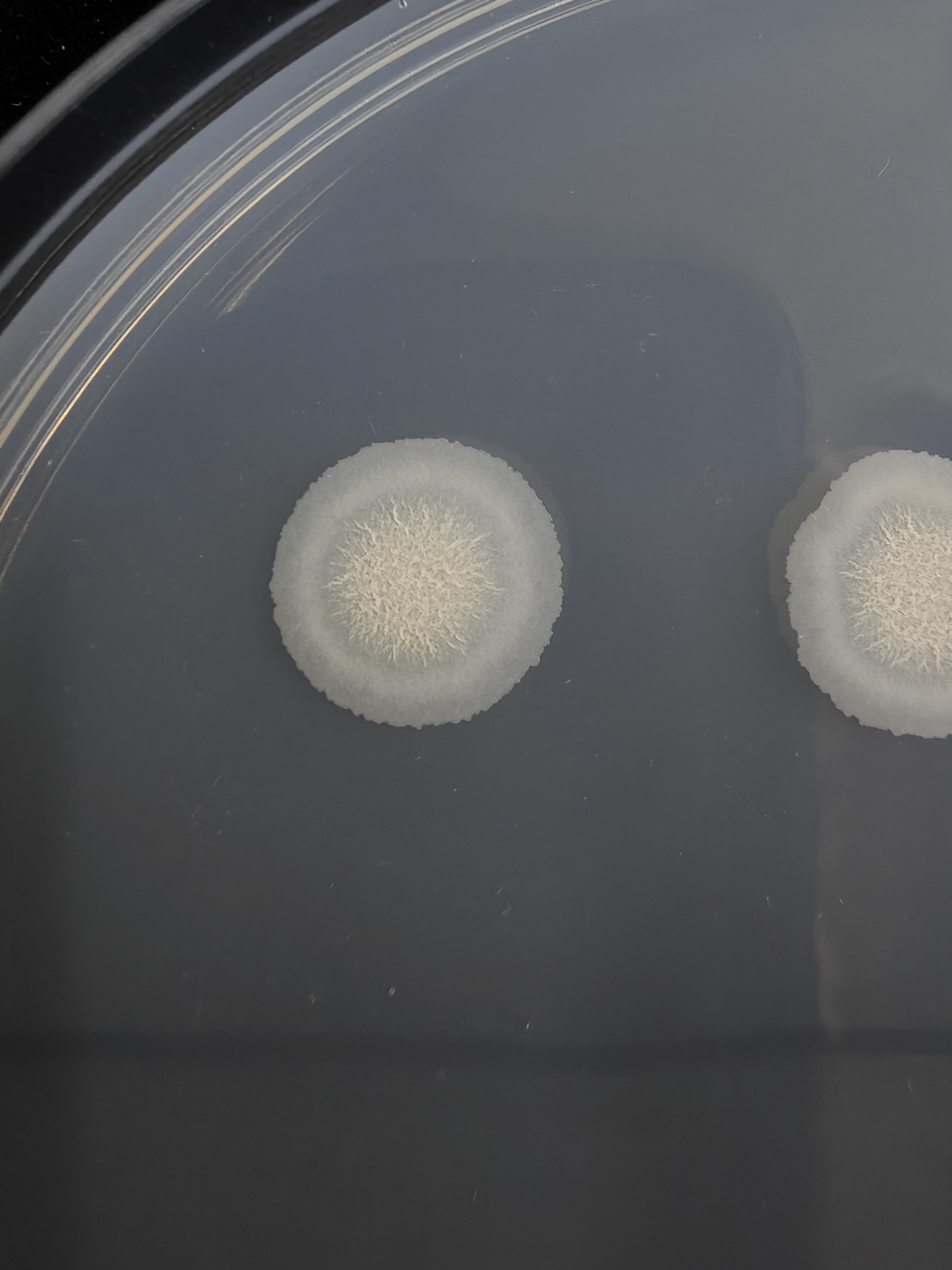

Supplement: Supplementary file 7 — Source data Fig. 5 [file 44319_2025_488_MOESM7_ESM.zip › Figure 5/5A/Mra-pJR962 without ATc induction.tiff]

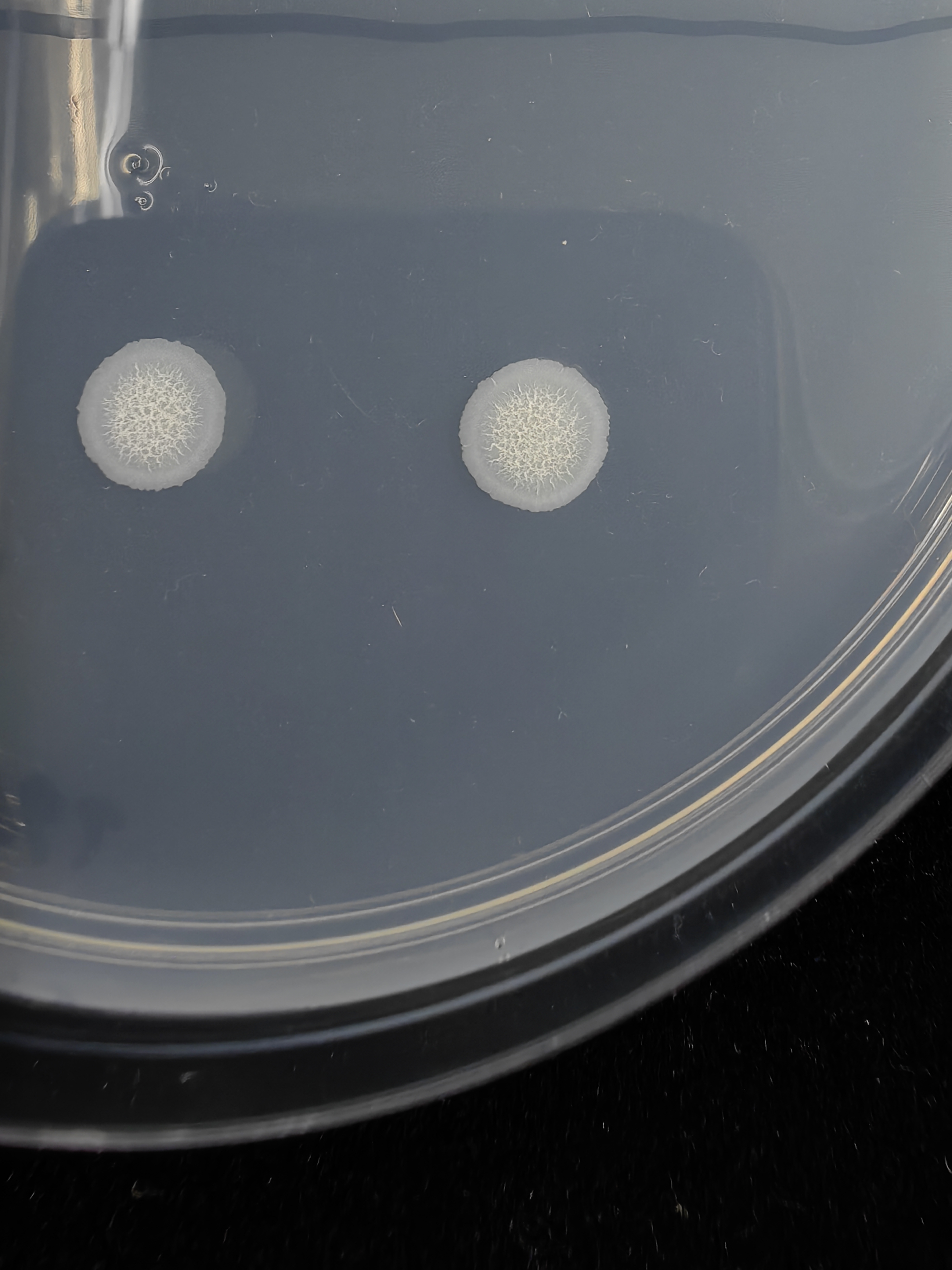

Supplement: Supplementary file 7 — Source data Fig. 5 [file 44319_2025_488_MOESM7_ESM.zip › Figure 5/5A/Mra-pJR962-gp48 with ATc induction.tiff]

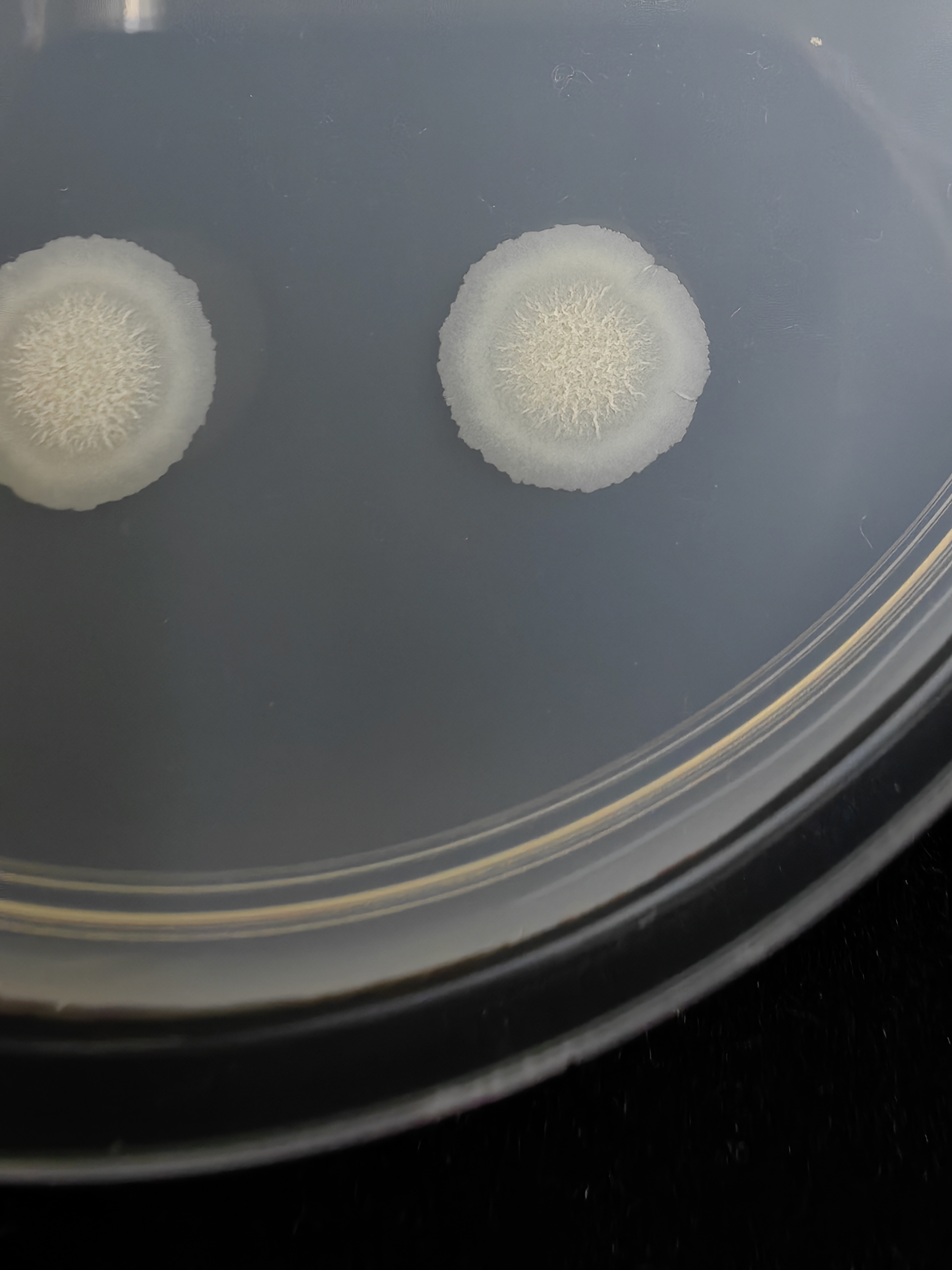

Supplement: Supplementary file 7 — Source data Fig. 5 [file 44319_2025_488_MOESM7_ESM.zip › Figure 5/5A/Mra-pJR962-gp48 without ATc induction.tiff]

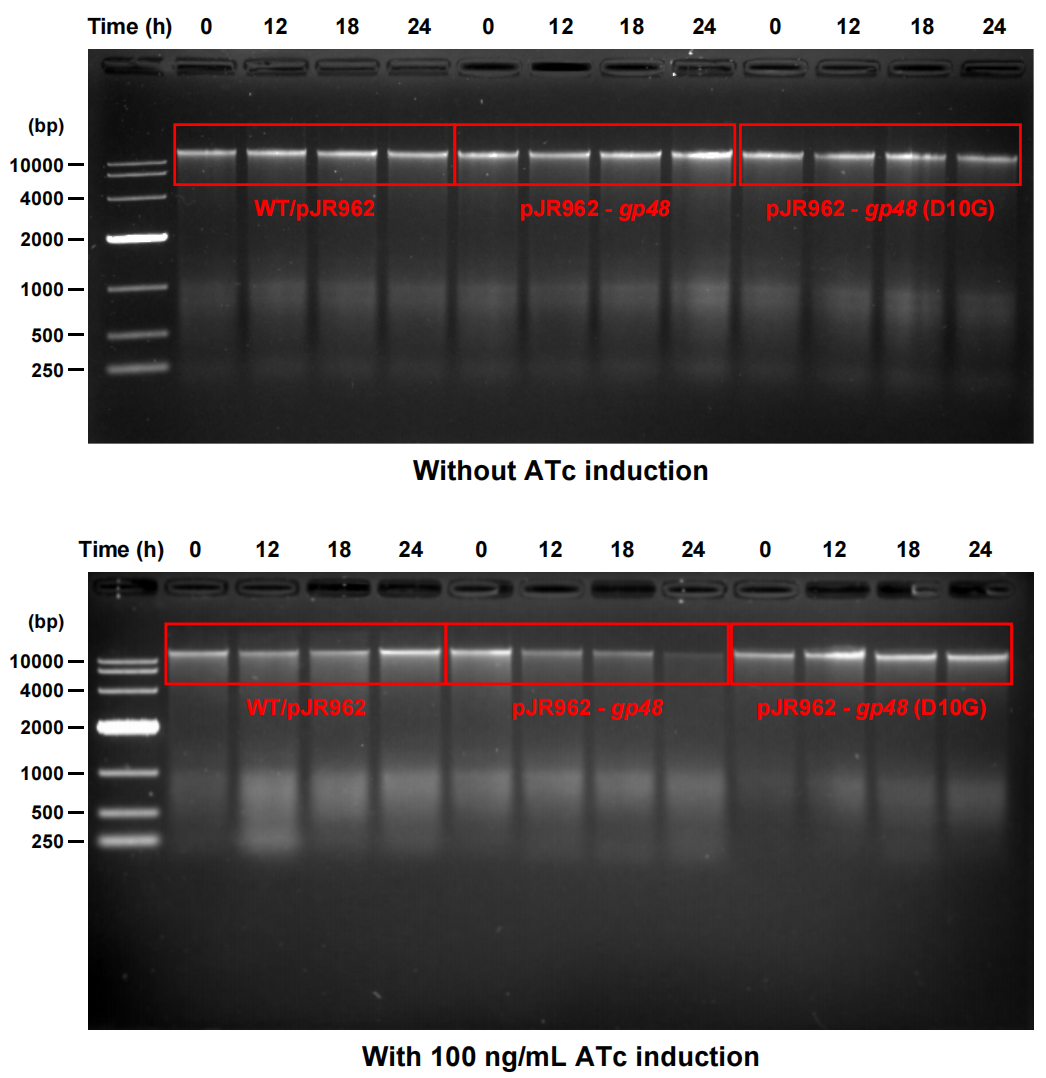

Supplement: Supplementary file 7 — Source data Fig. 5 [file 44319_2025_488_MOESM7_ESM.zip › Figure 5/5C/README.tif]

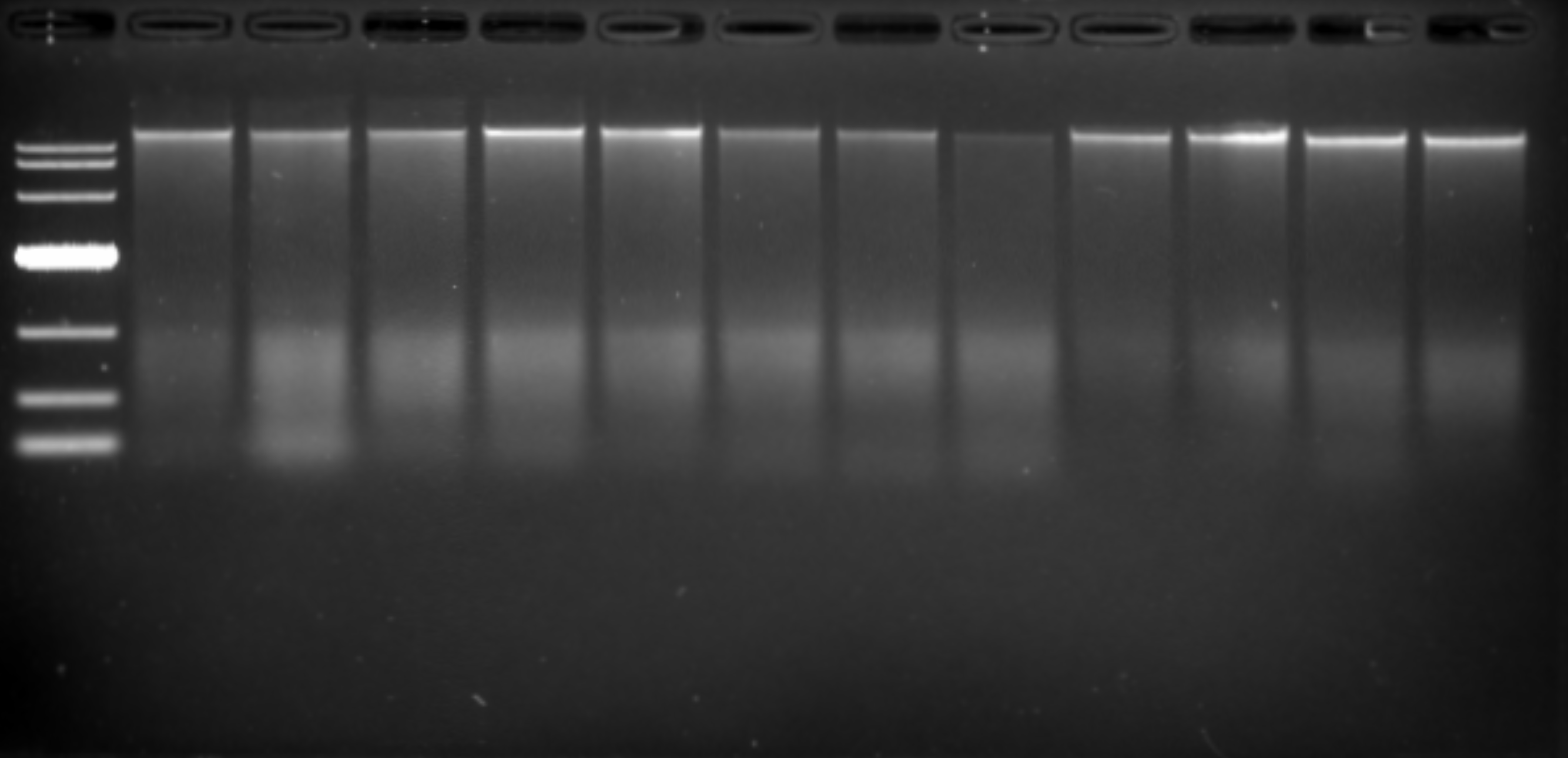

Supplement: Supplementary file 7 — Source data Fig. 5 [file 44319_2025_488_MOESM7_ESM.zip › Figure 5/5C/With ATc induction.tif]

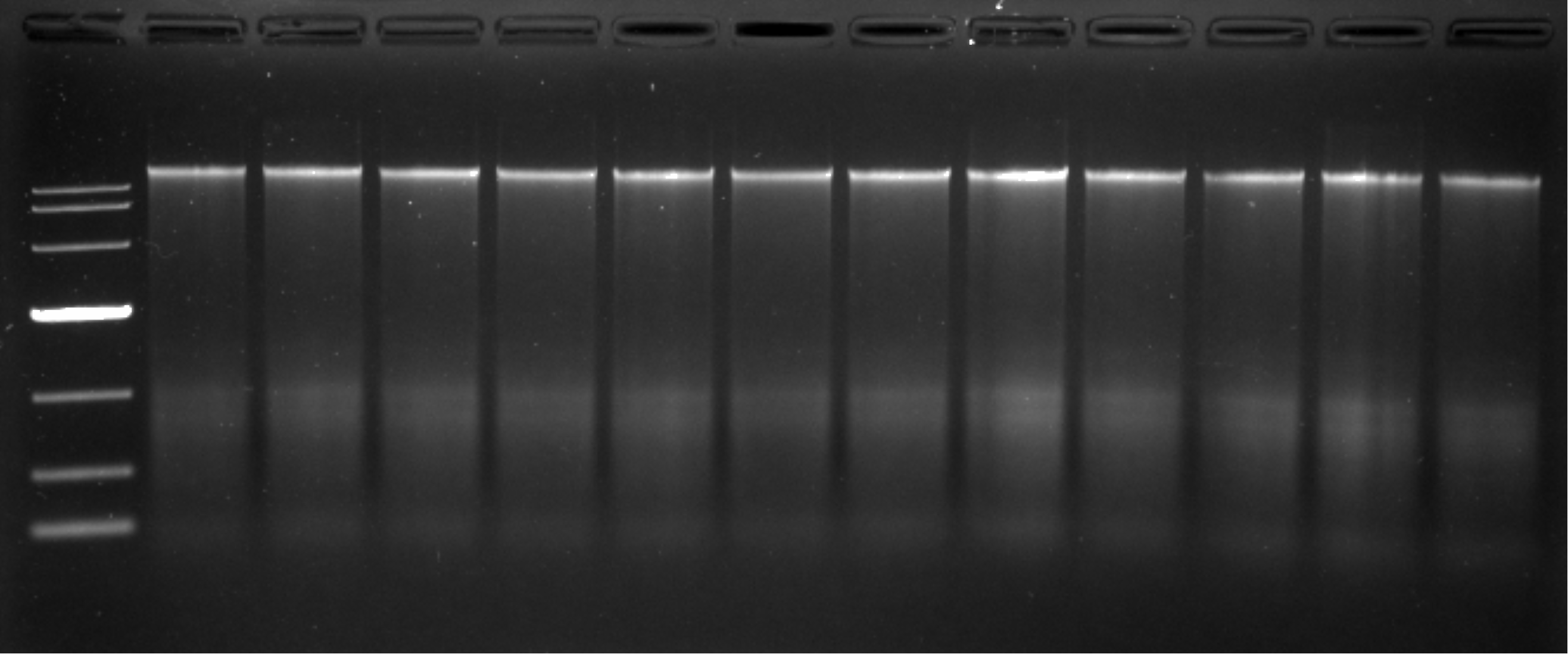

Supplement: Supplementary file 7 — Source data Fig. 5 [file 44319_2025_488_MOESM7_ESM.zip › Figure 5/5C/Without ATc induction.tif]

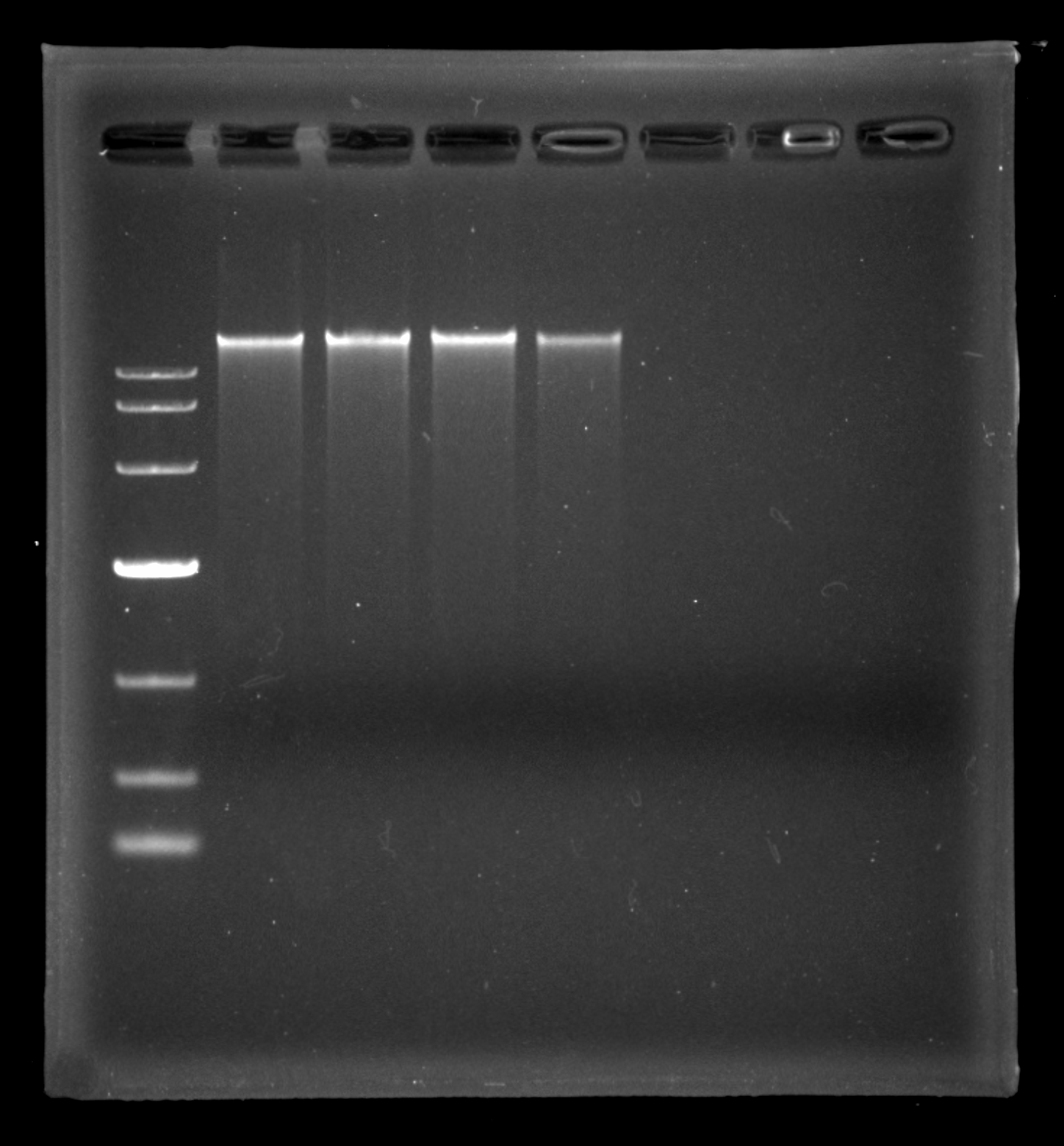

Supplement: Supplementary file 7 — Source data Fig. 5 [file 44319_2025_488_MOESM7_ESM.zip › Figure 5/5D/Bacterial genome.tif]

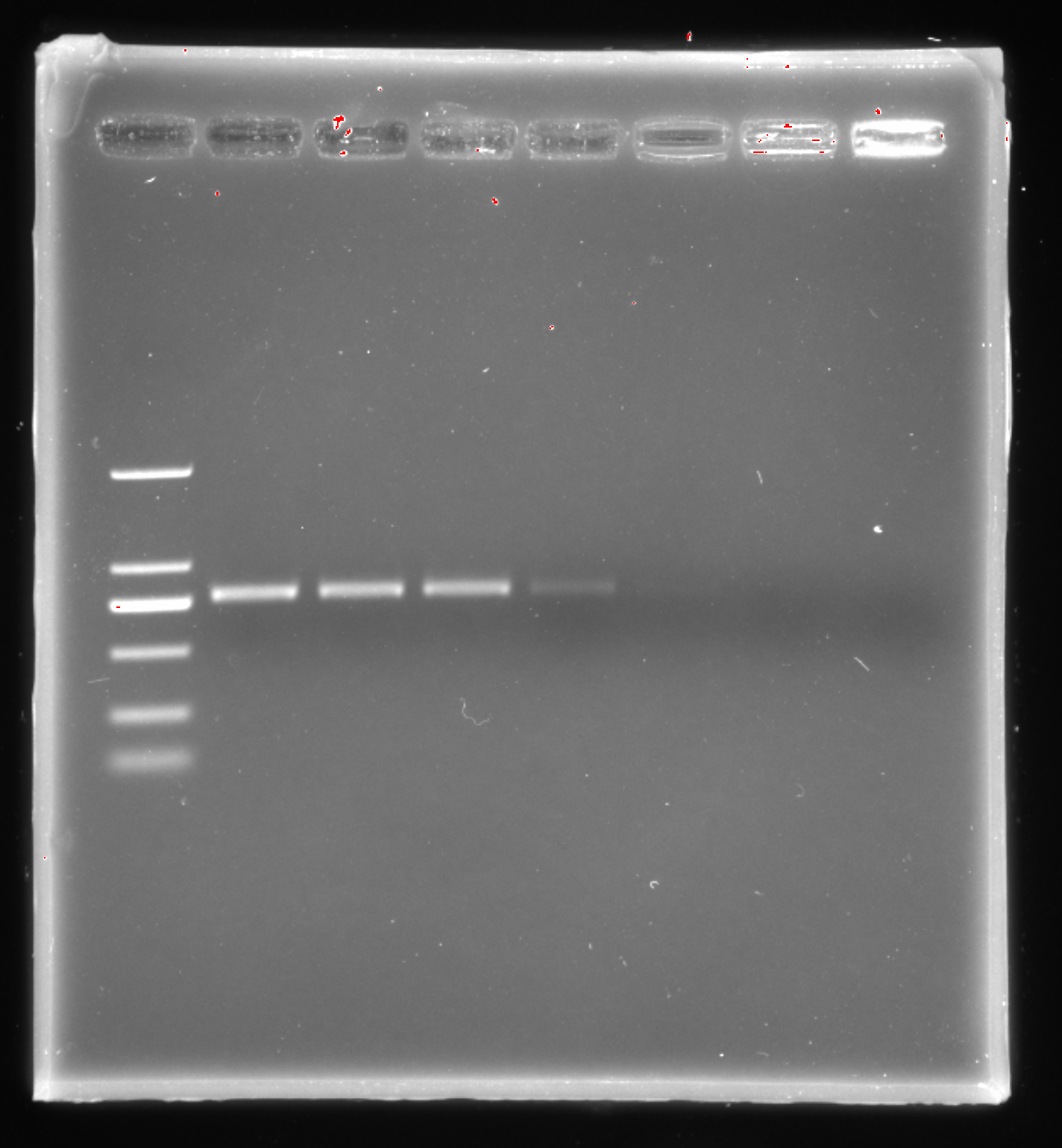

Supplement: Supplementary file 7 — Source data Fig. 5 [file 44319_2025_488_MOESM7_ESM.zip › Figure 5/5D/Linear DNA.tif]

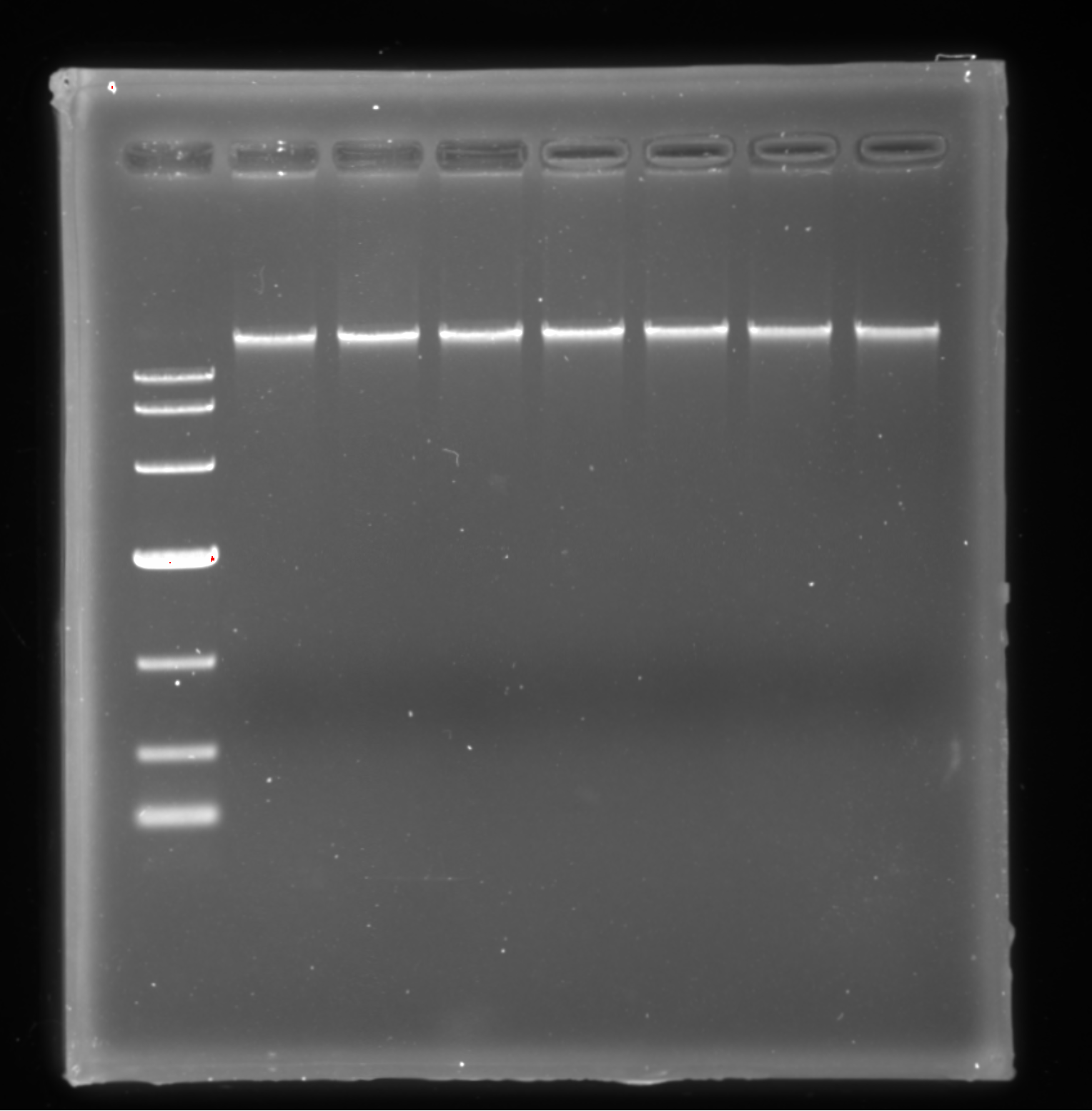

Supplement: Supplementary file 7 — Source data Fig. 5 [file 44319_2025_488_MOESM7_ESM.zip › Figure 5/5D/Phage genome.tif]

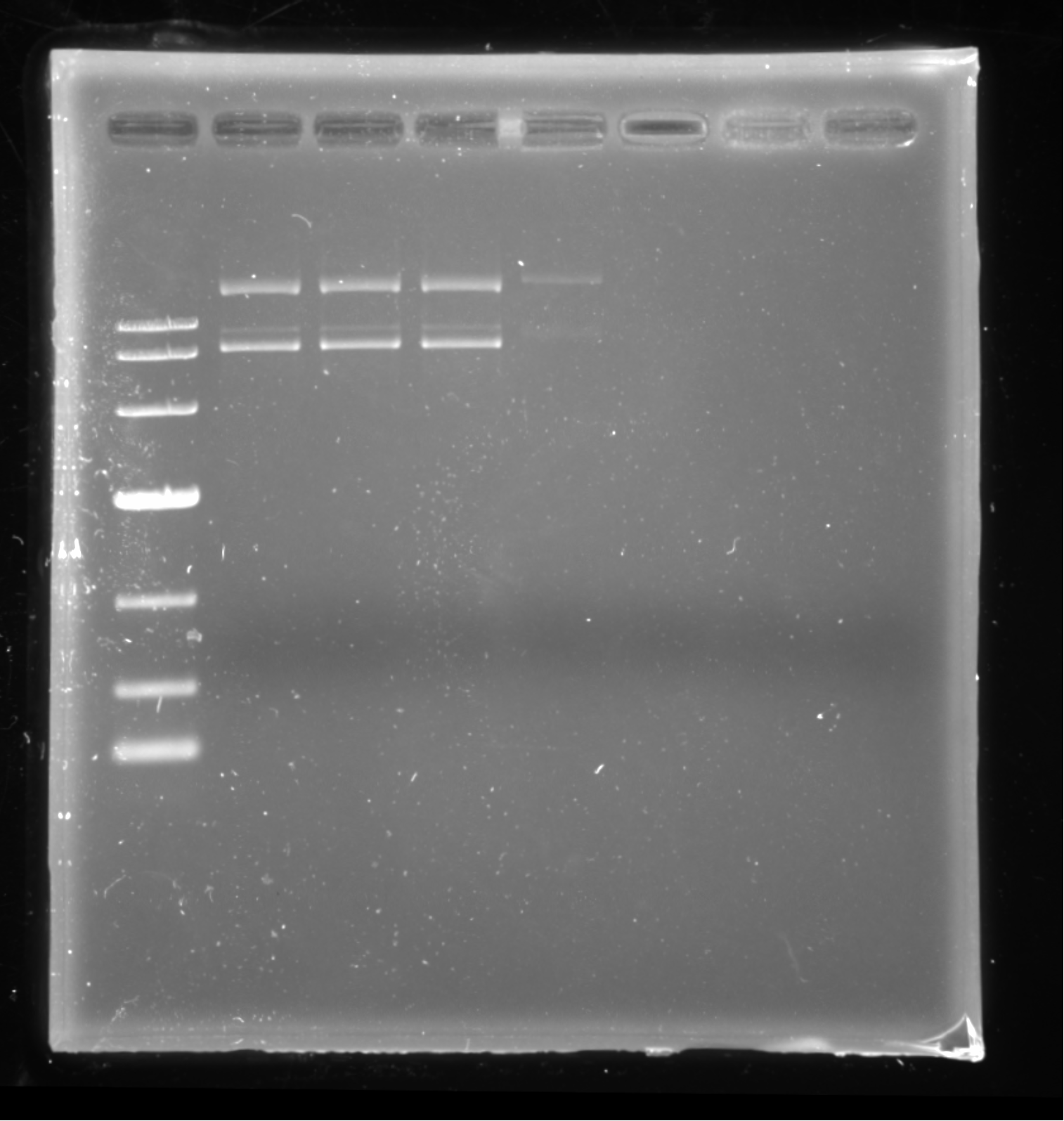

Supplement: Supplementary file 7 — Source data Fig. 5 [file 44319_2025_488_MOESM7_ESM.zip › Figure 5/5D/Plasmid.tif]

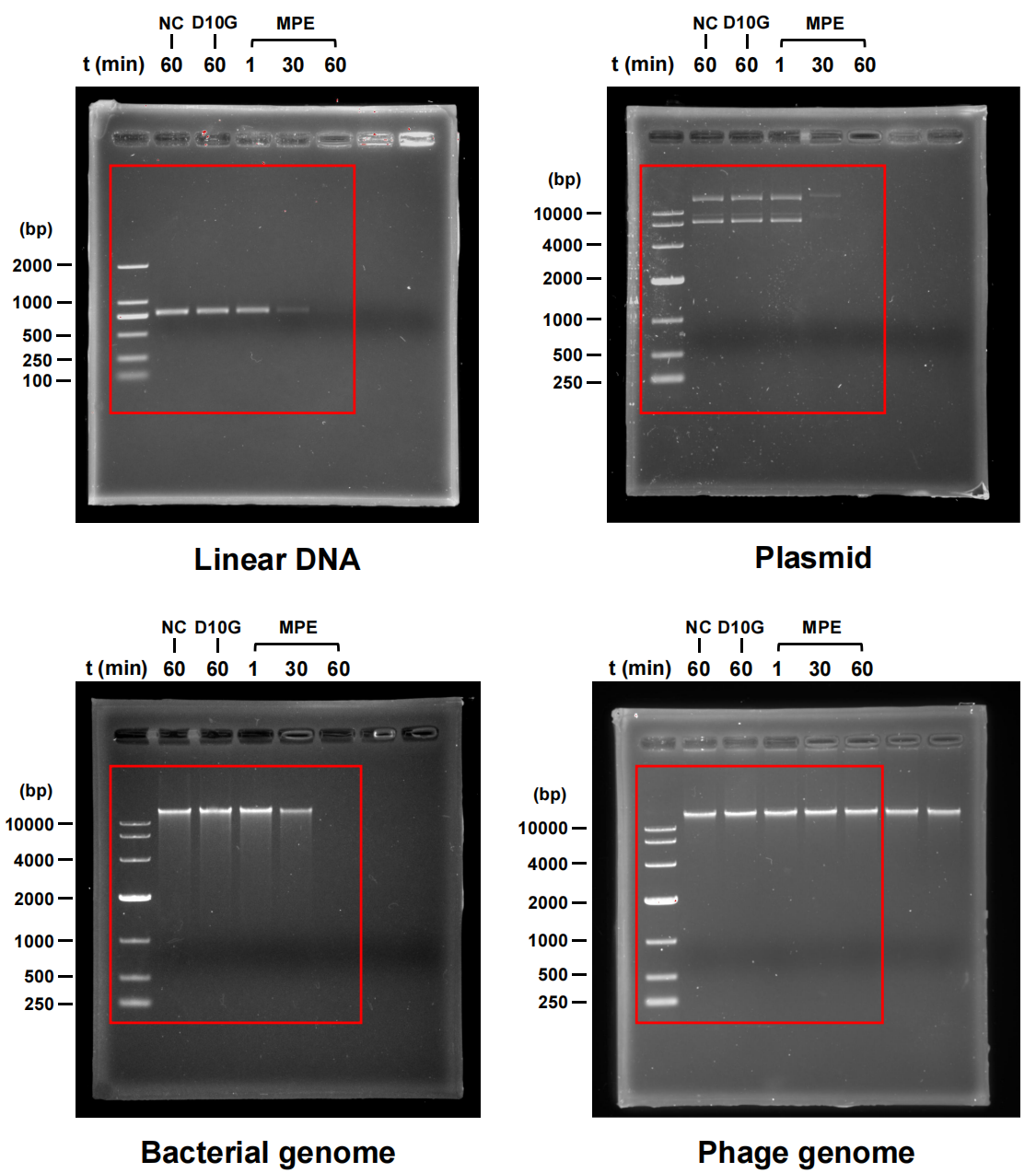

Supplement: Supplementary file 7 — Source data Fig. 5 [file 44319_2025_488_MOESM7_ESM.zip › Figure 5/5D/README.tif]

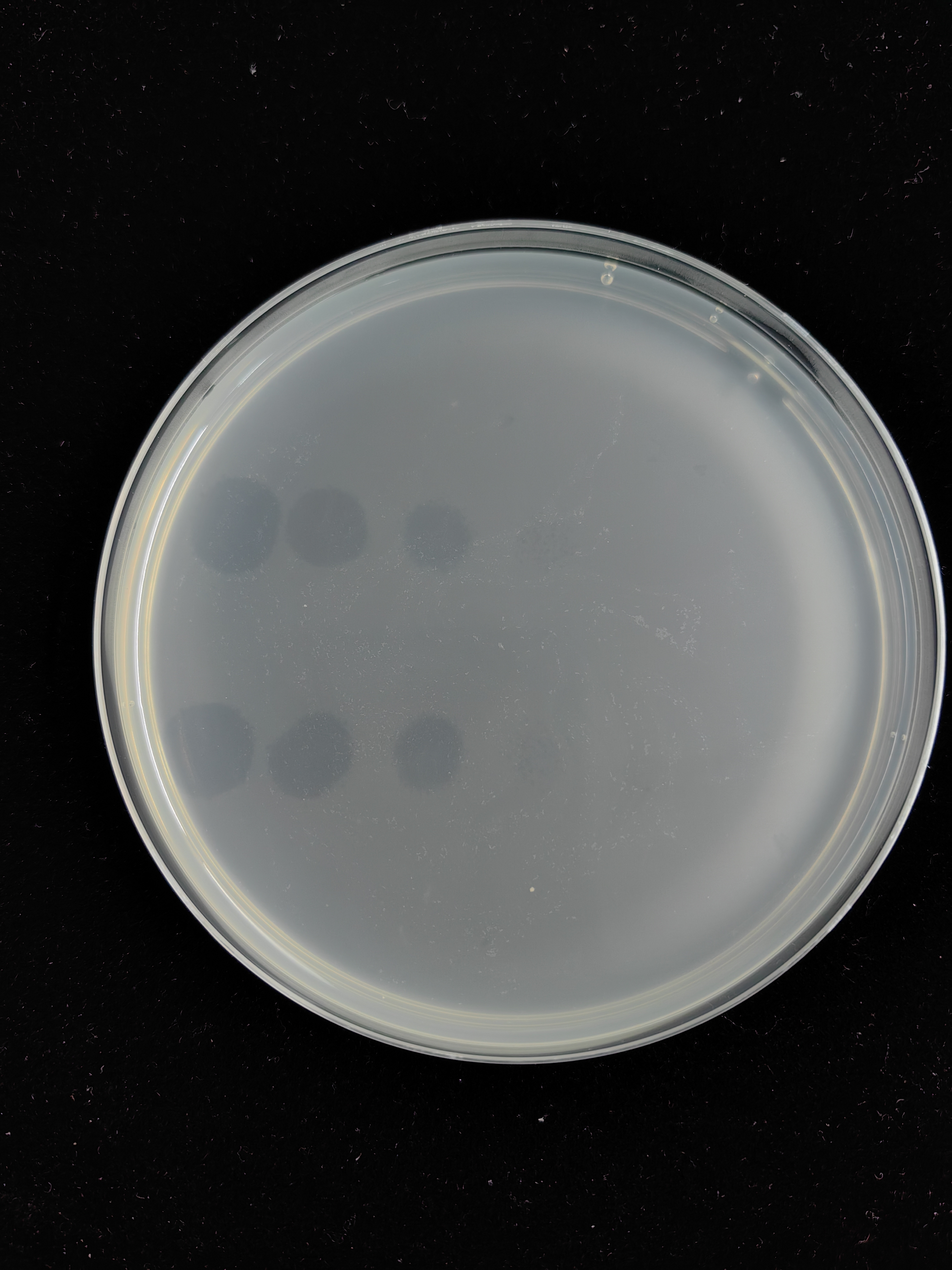

Supplement: Supplementary file 8 — Source data Fig. 6 [file 44319_2025_488_MOESM8_ESM.zip › Figure 6/6C/pJR962 with ATc induction.tiff]

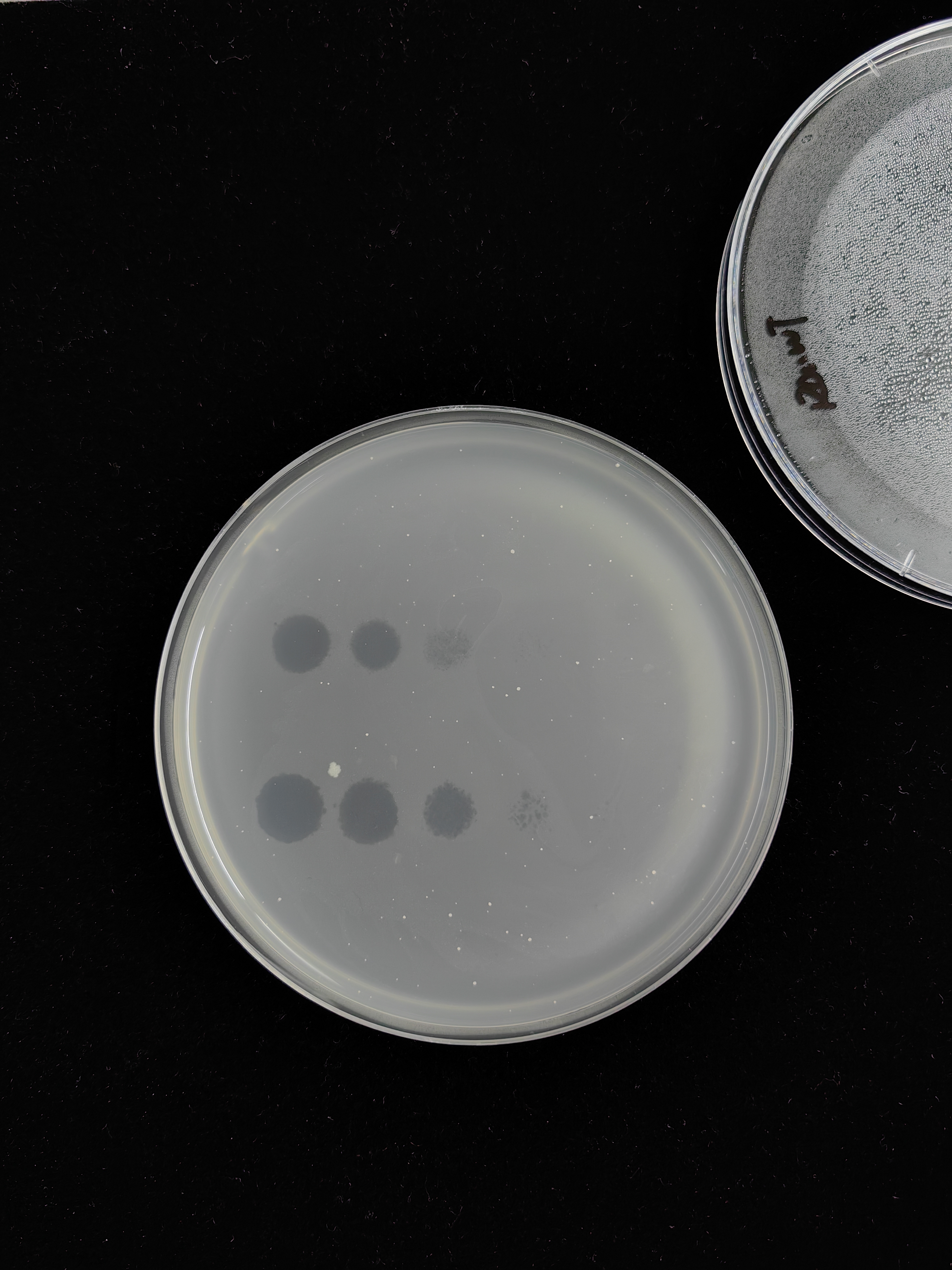

Supplement: Supplementary file 8 — Source data Fig. 6 [file 44319_2025_488_MOESM8_ESM.zip › Figure 6/6C/pJR962 without ATc induction.tiff]

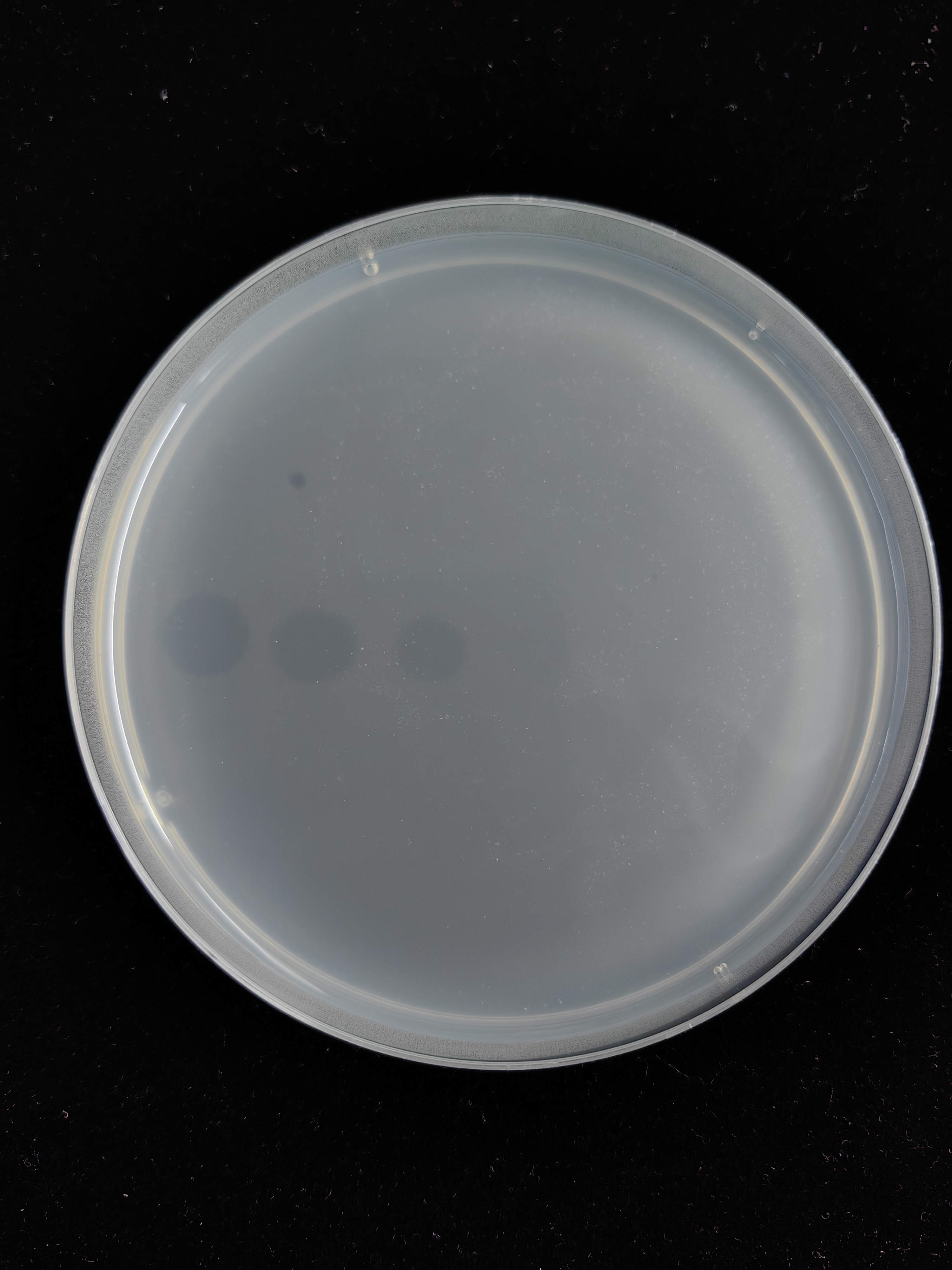

Supplement: Supplementary file 8 — Source data Fig. 6 [file 44319_2025_488_MOESM8_ESM.zip › Figure 6/6C/pJR962-Mra Csm with ATc induction.tiff]

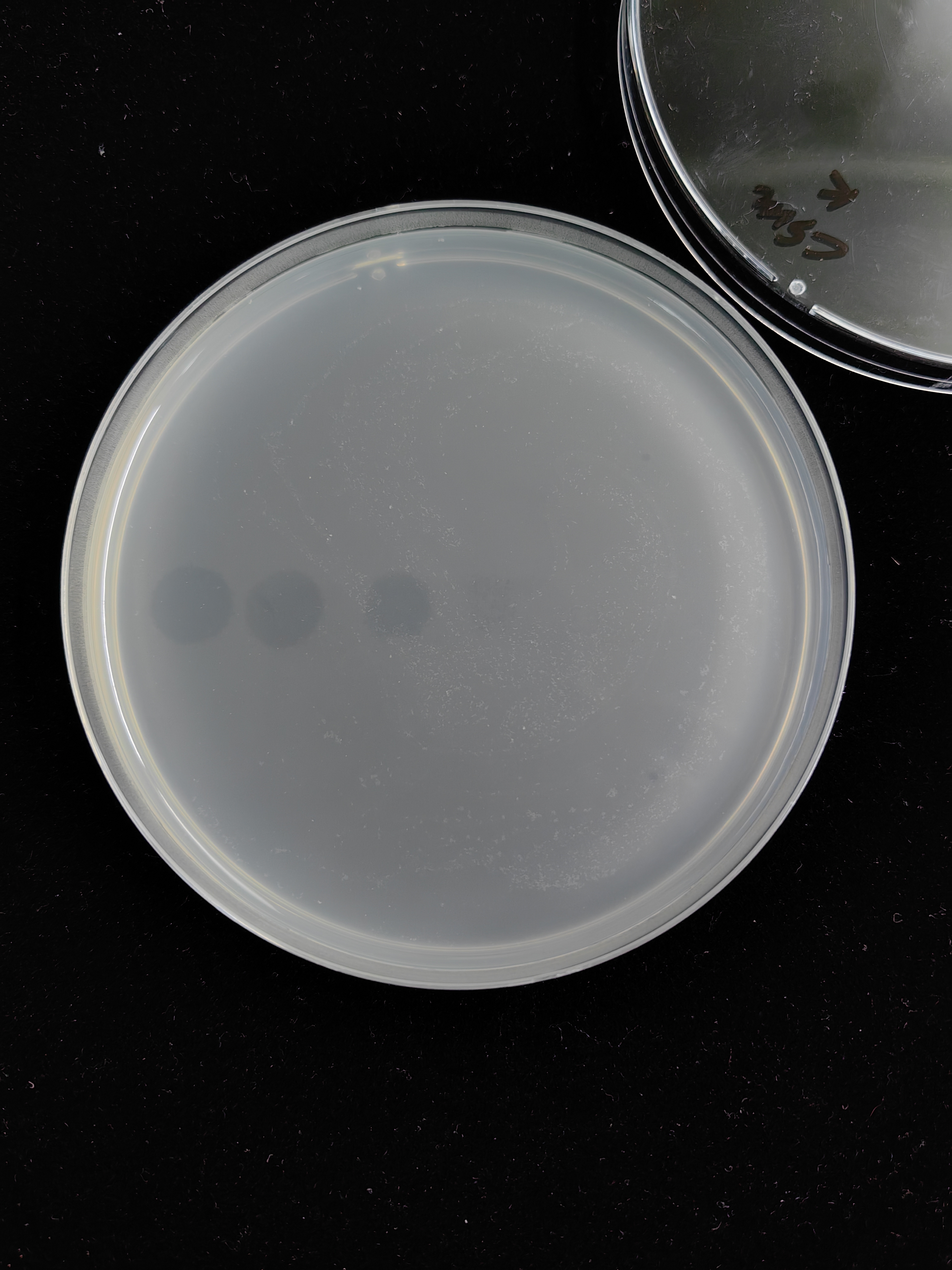

Supplement: Supplementary file 8 — Source data Fig. 6 [file 44319_2025_488_MOESM8_ESM.zip › Figure 6/6C/pJR962-Mra Csm without ATc induction.tiff]

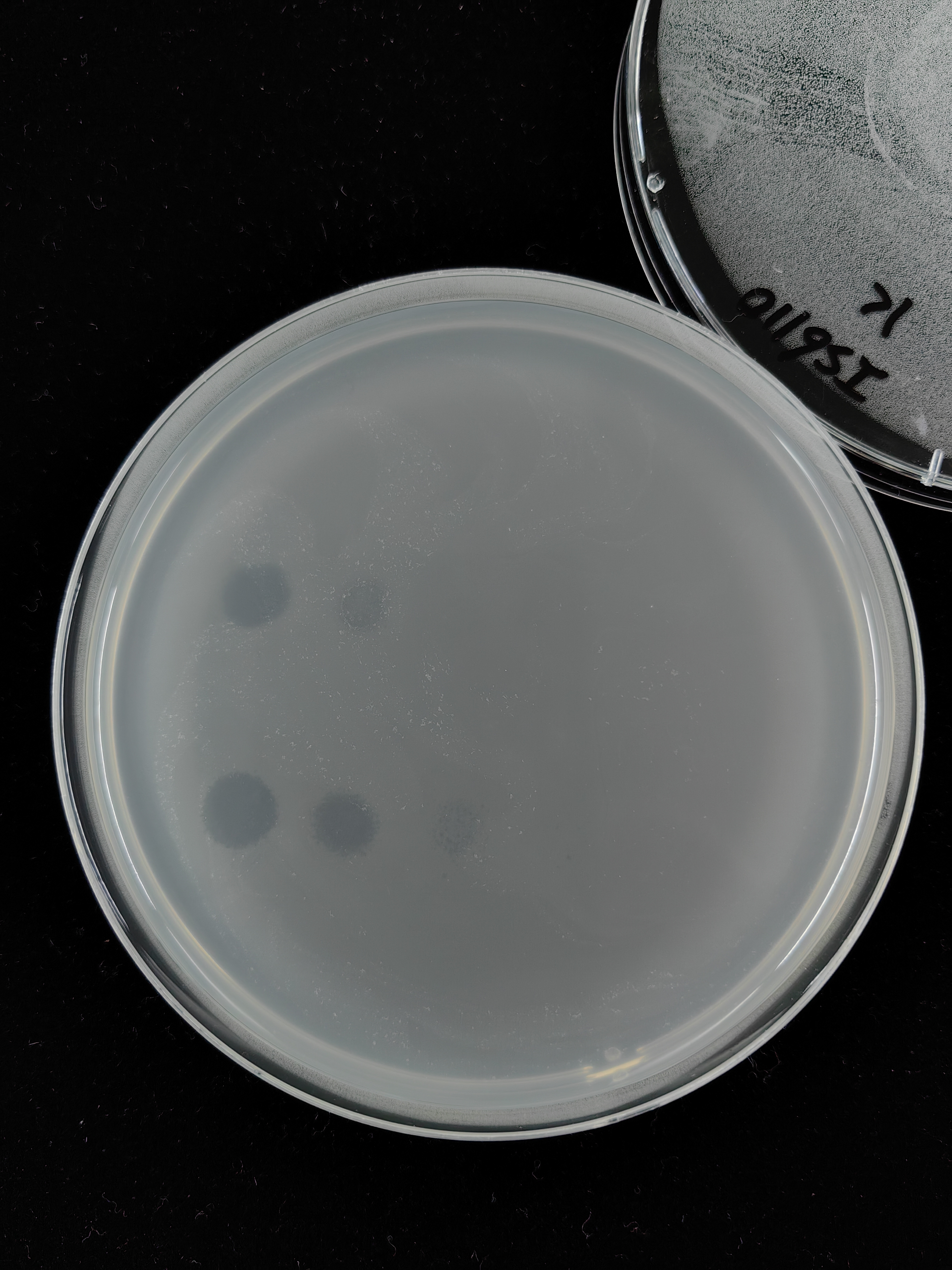

Supplement: Supplementary file 8 — Source data Fig. 6 [file 44319_2025_488_MOESM8_ESM.zip › Figure 6/6C/pJR962-Mra IS6110 with ATc induction.tiff]

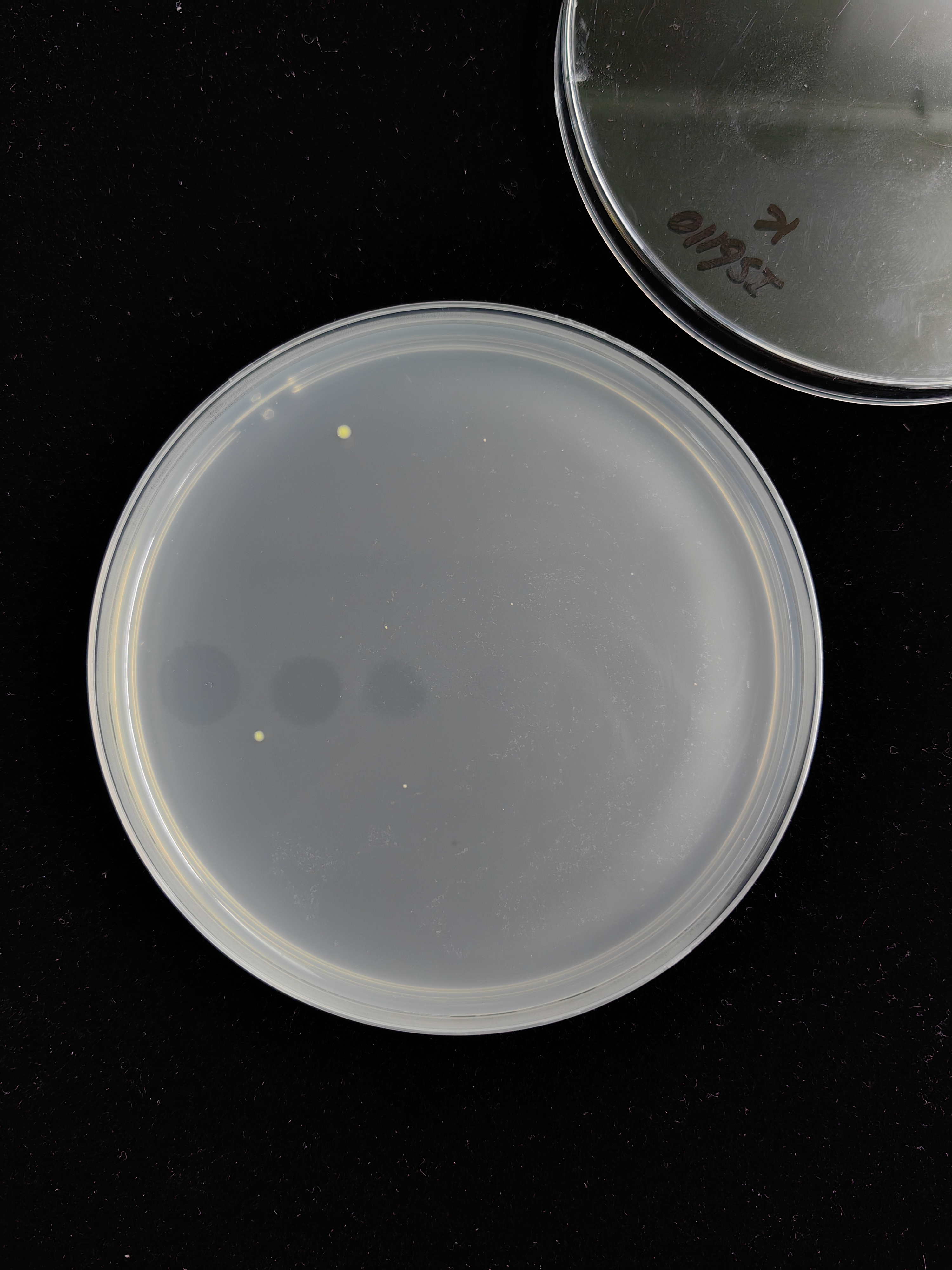

Supplement: Supplementary file 8 — Source data Fig. 6 [file 44319_2025_488_MOESM8_ESM.zip › Figure 6/6C/pJR962-Mra IS6110 without ATc induction.tiff]

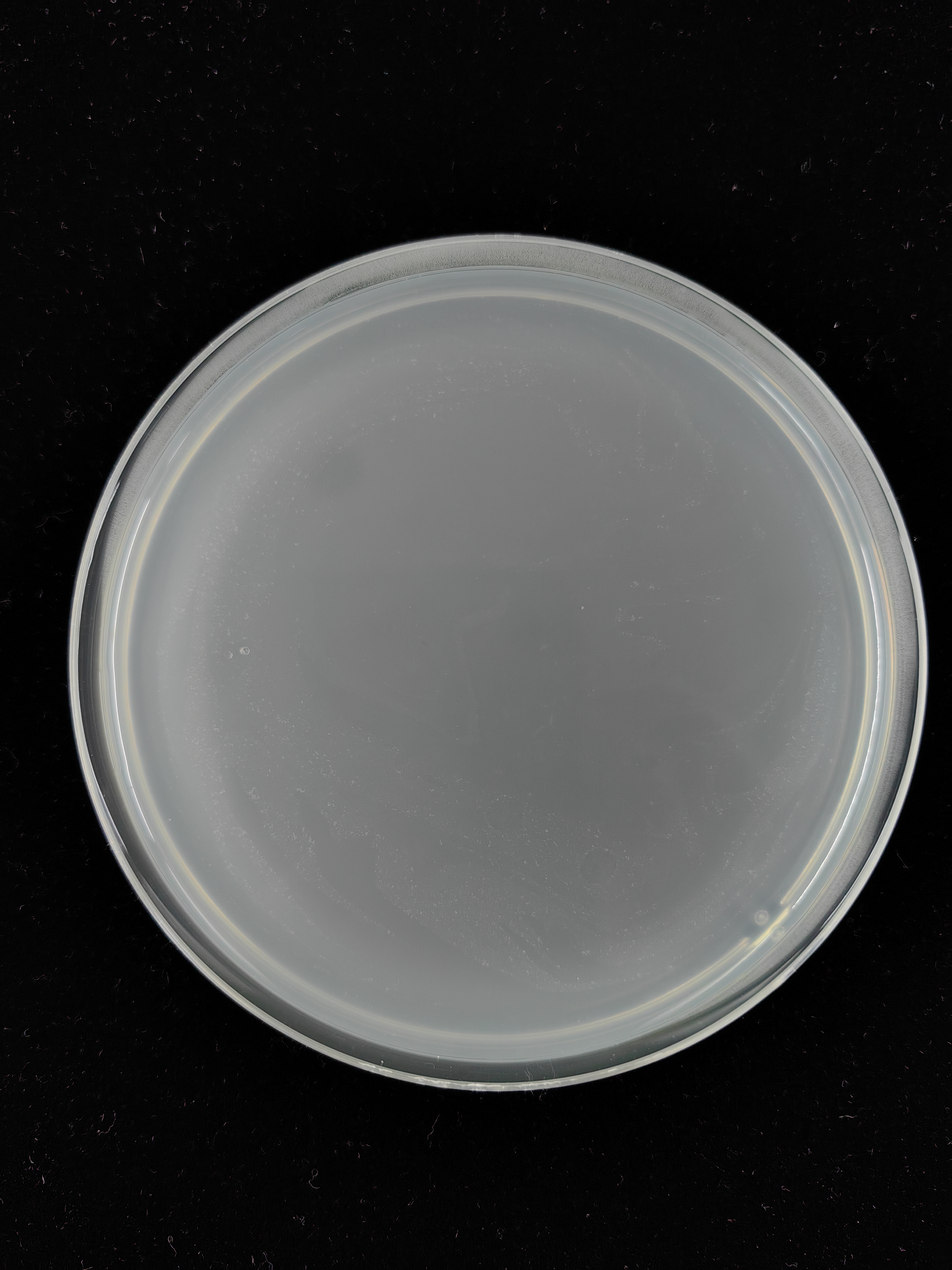

Supplement: Supplementary file 8 — Source data Fig. 6 [file 44319_2025_488_MOESM8_ESM.zip › Figure 6/6C/pJR962-Mra1649 with ATc induction.tiff]

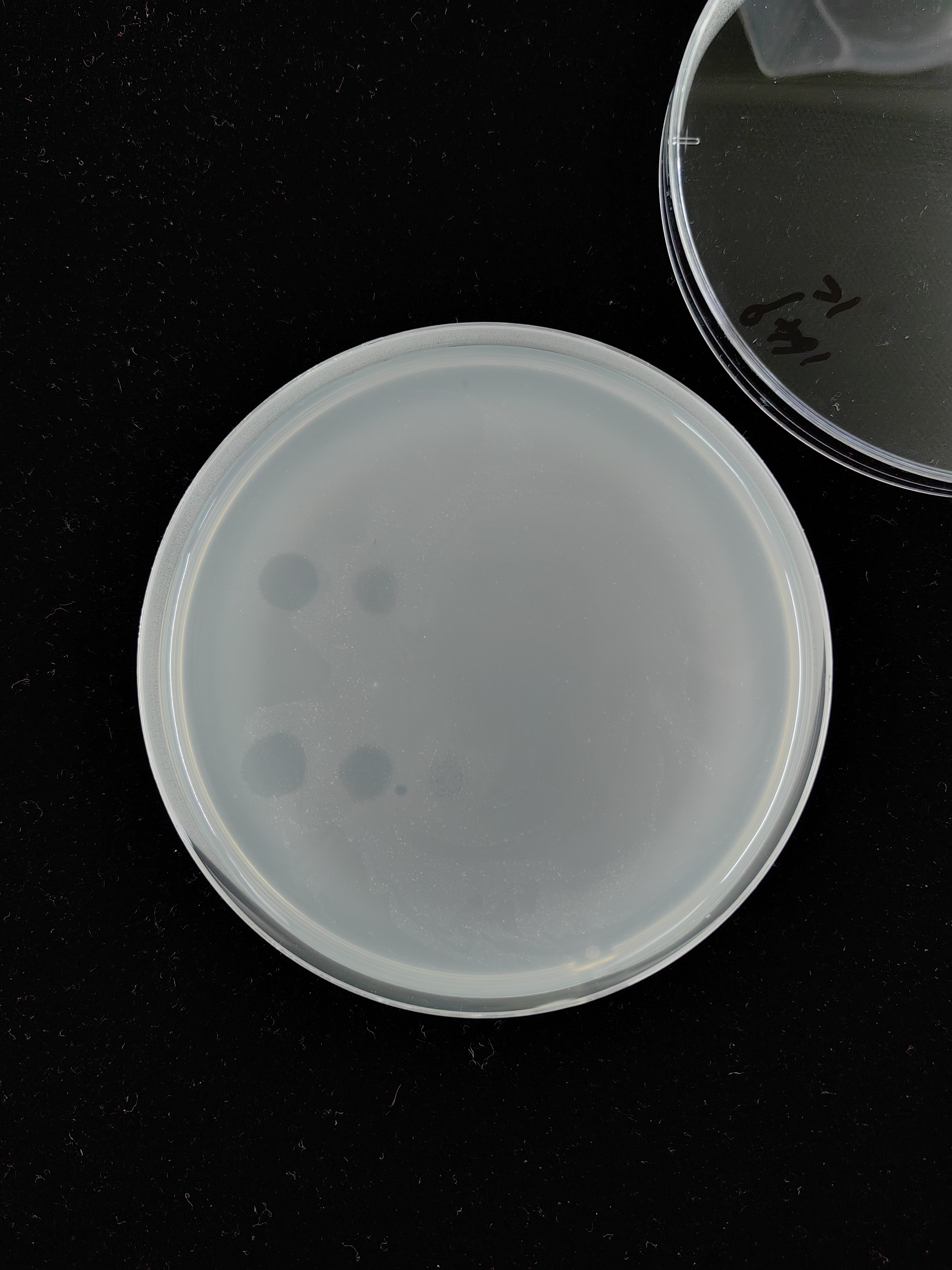

Supplement: Supplementary file 8 — Source data Fig. 6 [file 44319_2025_488_MOESM8_ESM.zip › Figure 6/6C/pJR962-Mra1649 without ATc induction.tiff]

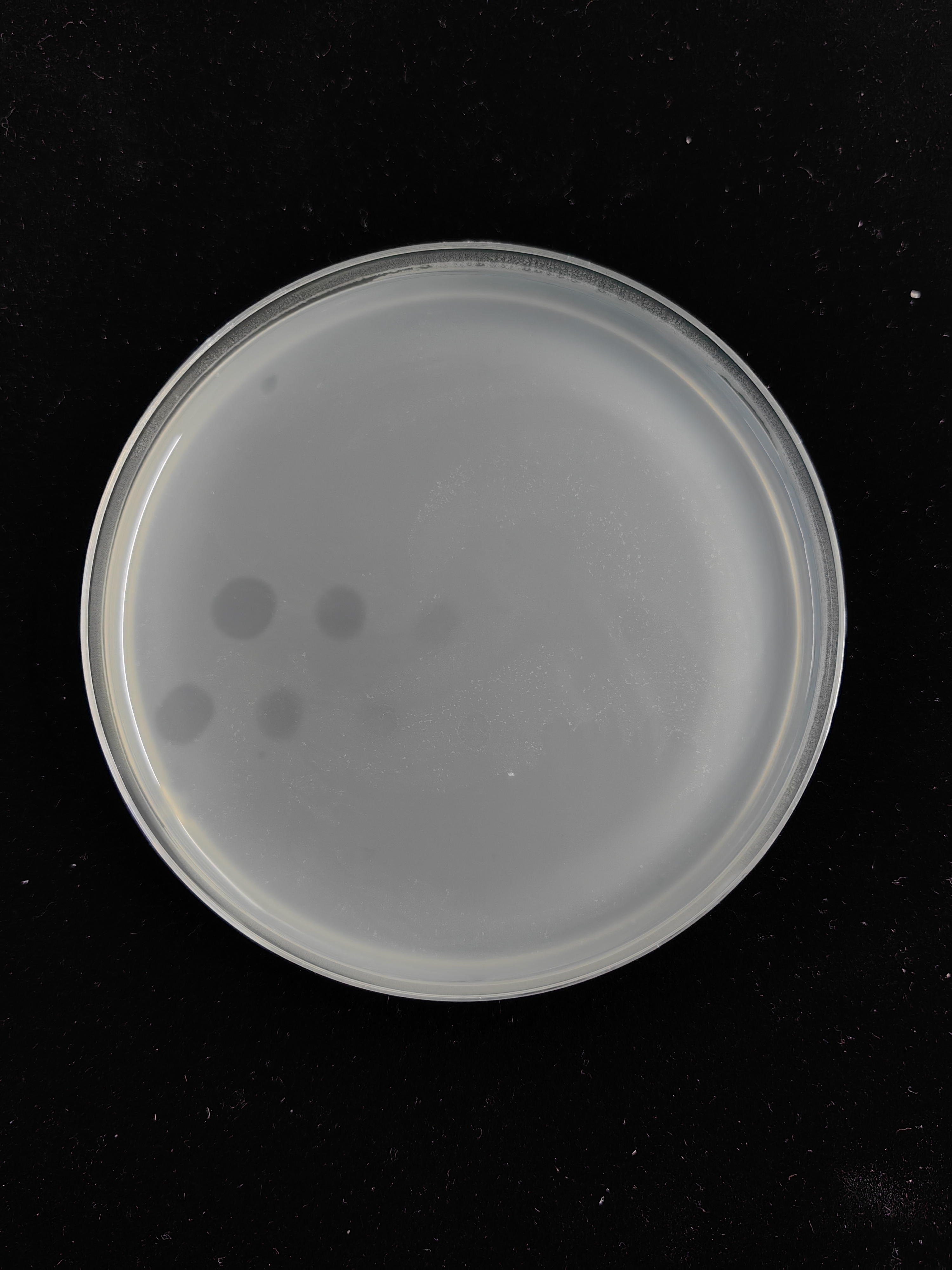

Supplement: Supplementary file 8 — Source data Fig. 6 [file 44319_2025_488_MOESM8_ESM.zip › Figure 6/6C/pJR962-Mra1940A with ATc induction.tiff]

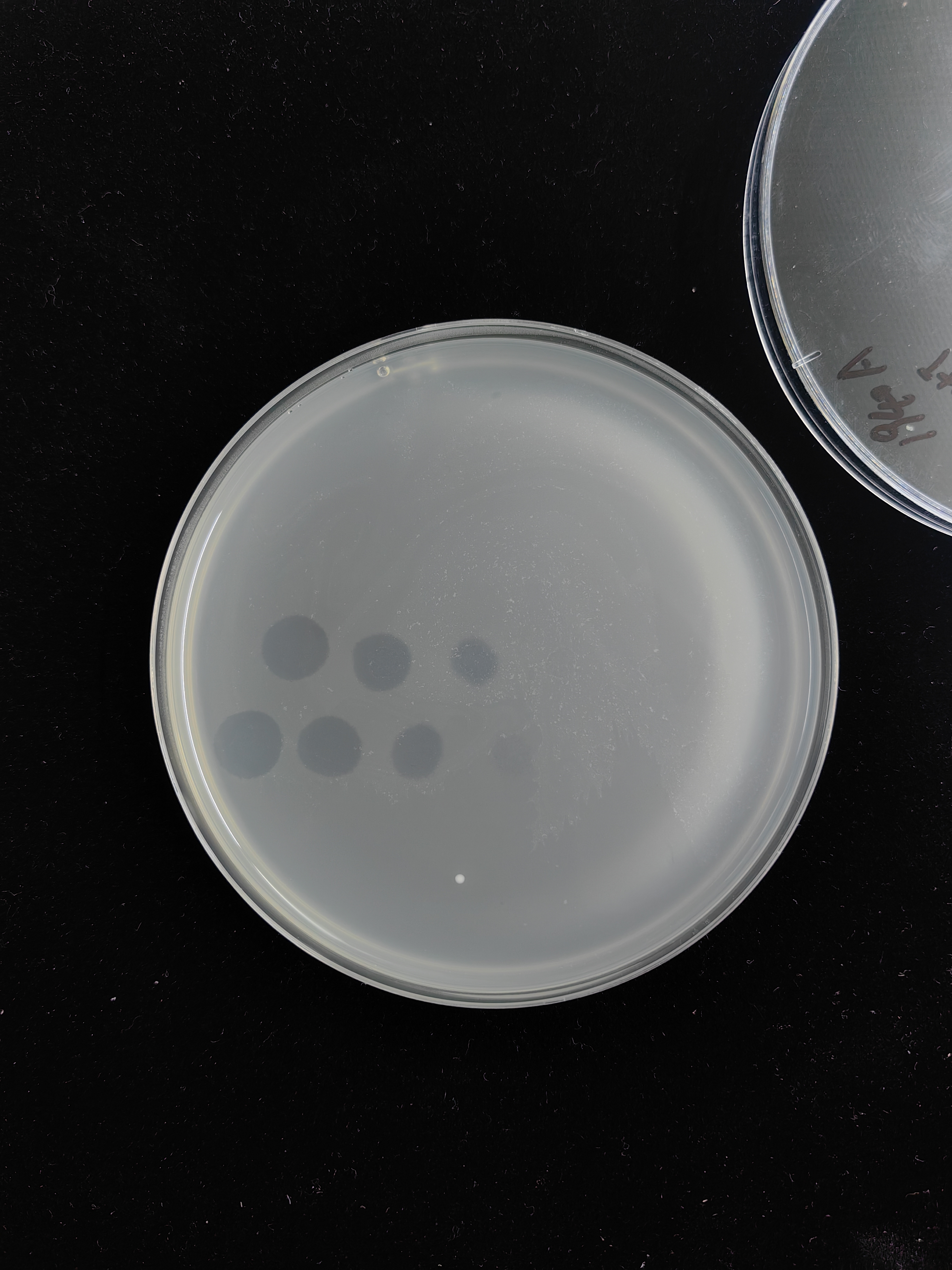

Supplement: Supplementary file 8 — Source data Fig. 6 [file 44319_2025_488_MOESM8_ESM.zip › Figure 6/6C/pJR962-Mra1940A without ATc induction.tiff]

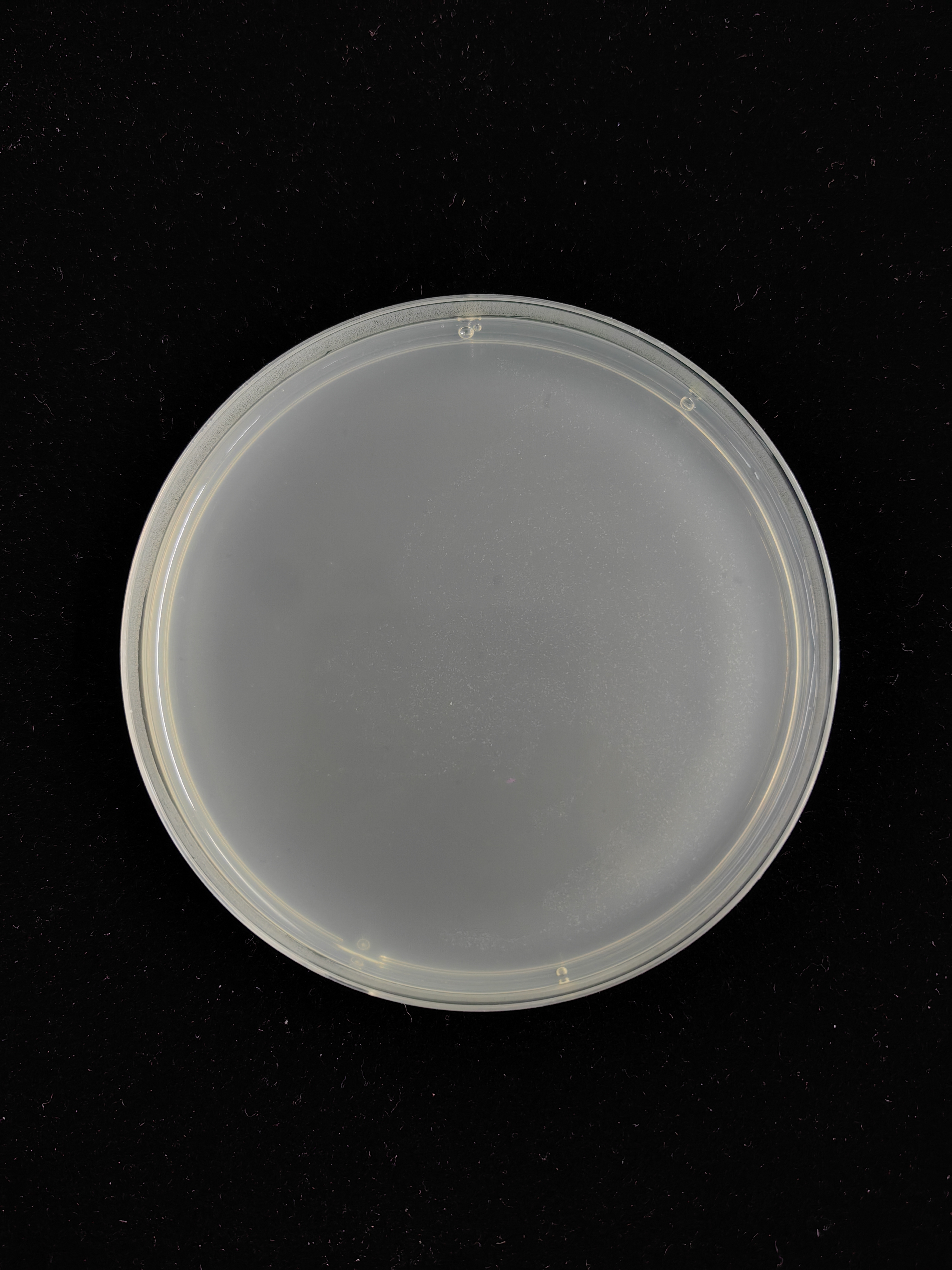

Supplement: Supplementary file 8 — Source data Fig. 6 [file 44319_2025_488_MOESM8_ESM.zip › Figure 6/6C/pJR962-Mra3122 with ATc induction.tiff]

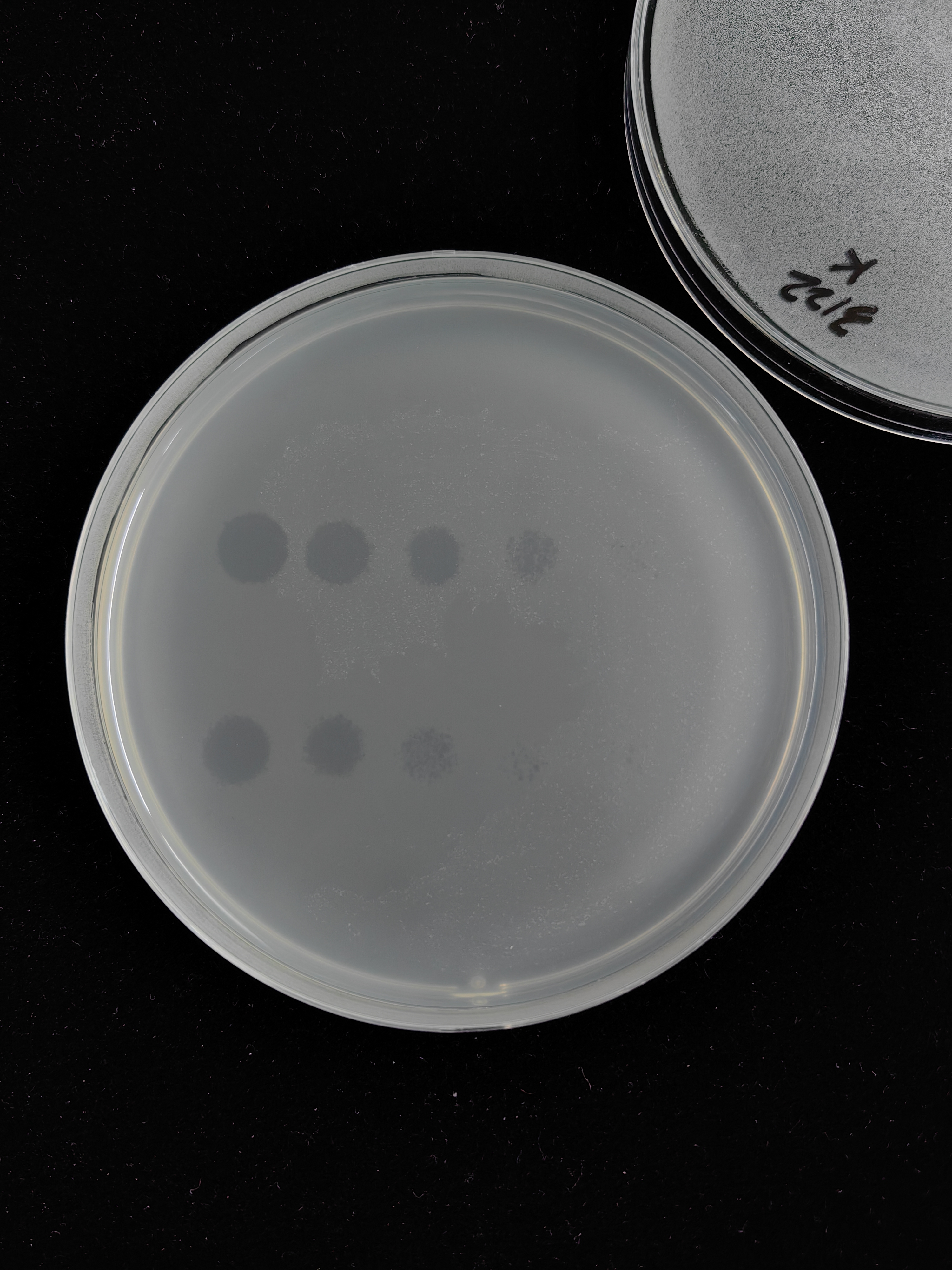

Supplement: Supplementary file 8 — Source data Fig. 6 [file 44319_2025_488_MOESM8_ESM.zip › Figure 6/6C/pJR962-Mra3122 without ATc induction.tiff]

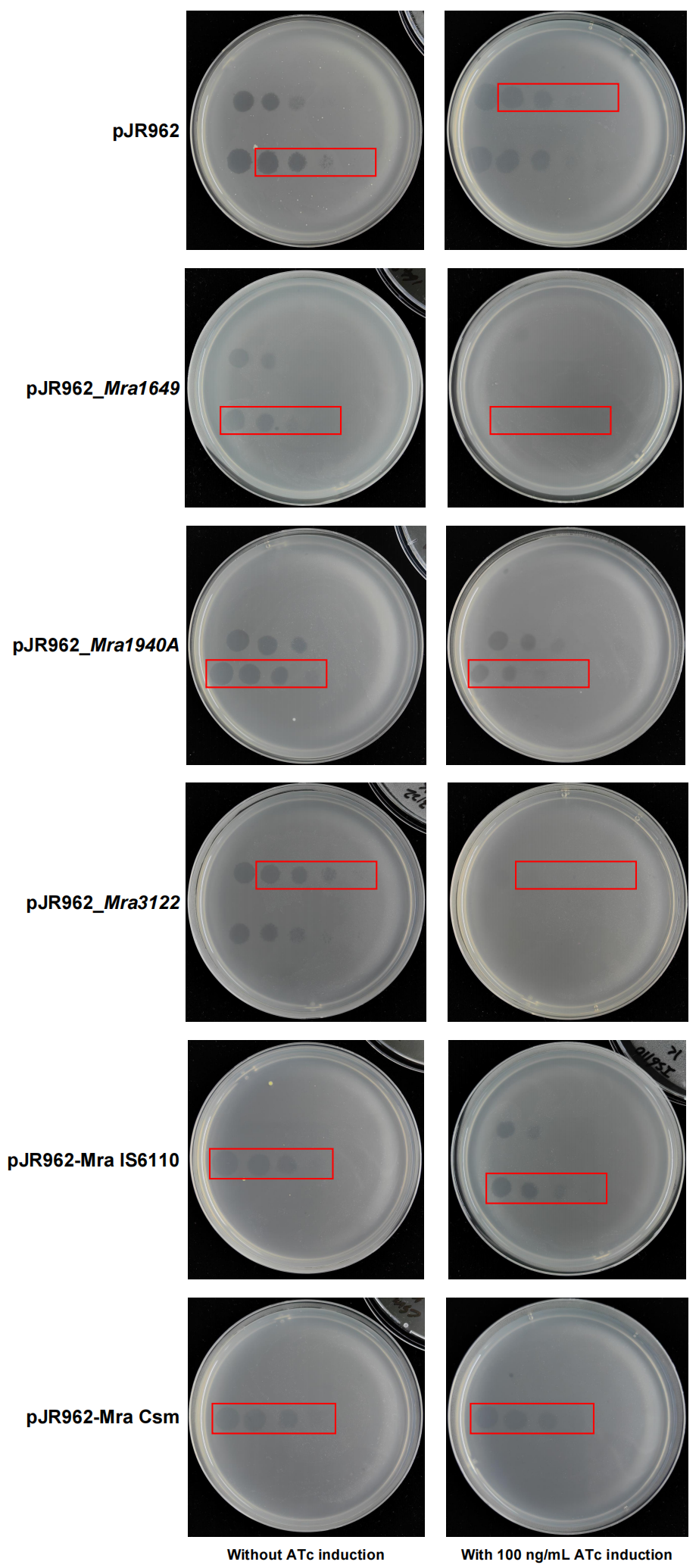

Supplement: Supplementary file 8 — Source data Fig. 6 [file 44319_2025_488_MOESM8_ESM.zip › Figure 6/6C/README.tif]

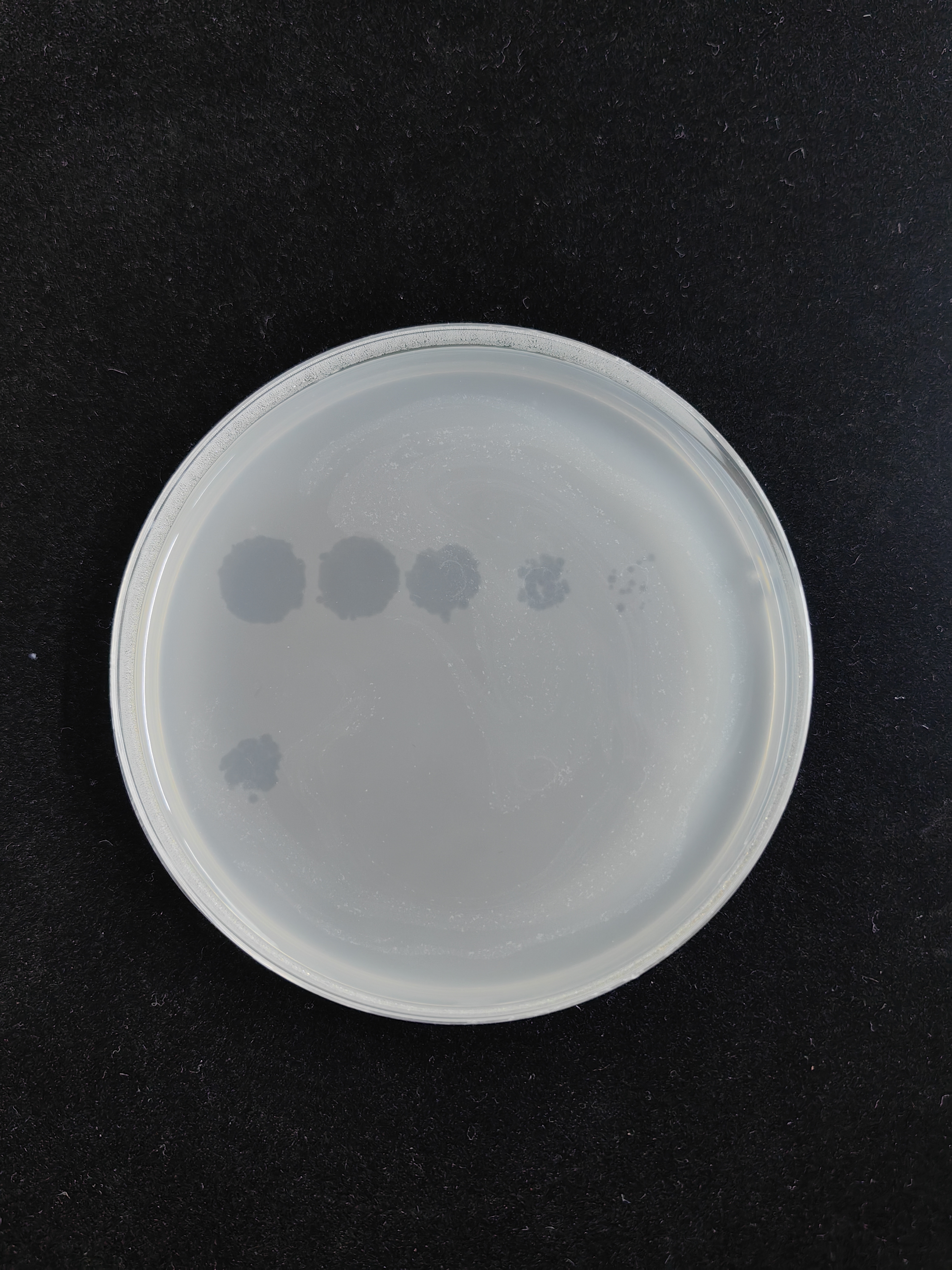

Supplement: Supplementary file 8 — Source data Fig. 6 [file 44319_2025_488_MOESM8_ESM.zip › Figure 6/6D/pLJR965-Mra_1649 with induction.tiff]

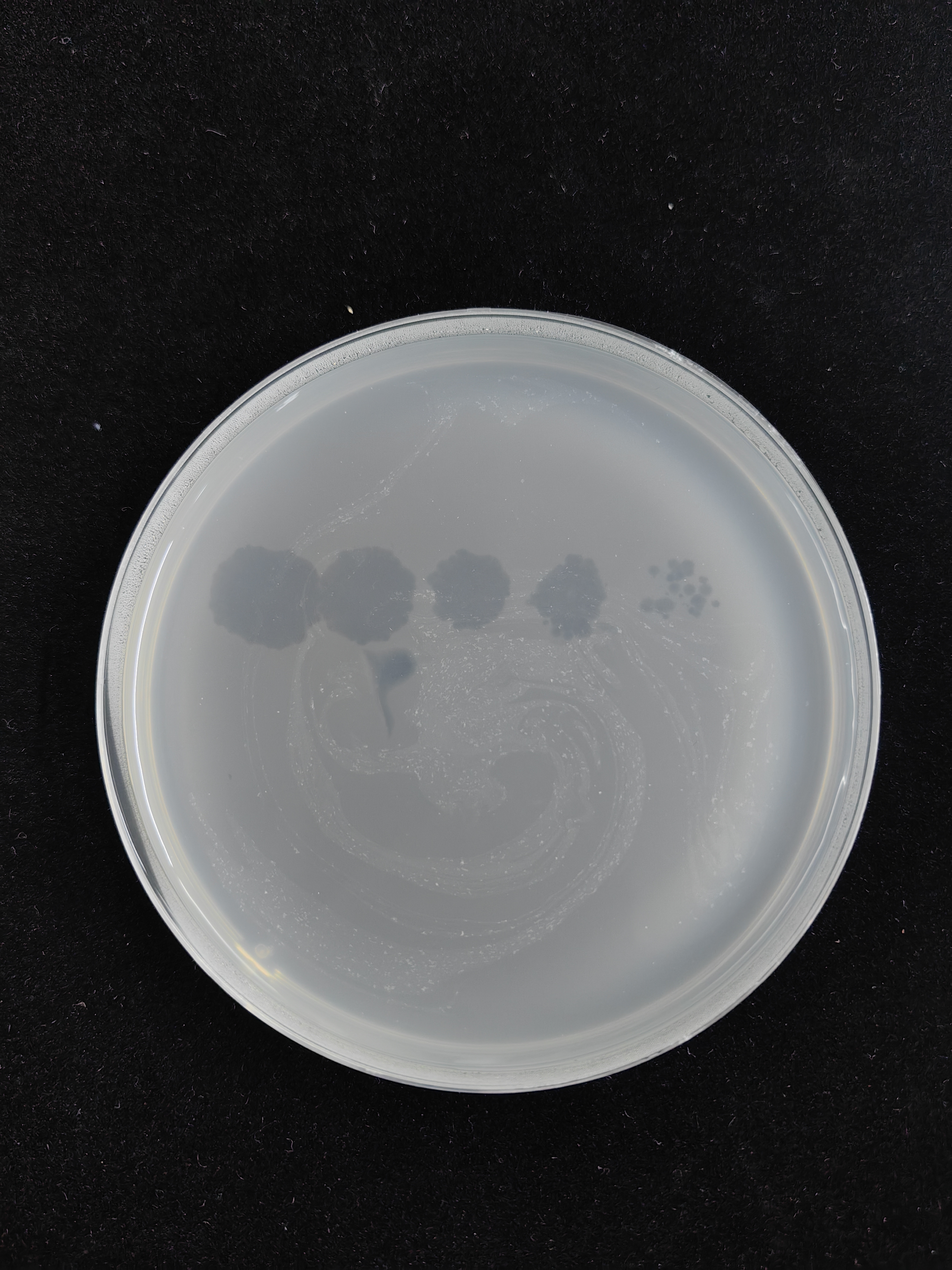

Supplement: Supplementary file 8 — Source data Fig. 6 [file 44319_2025_488_MOESM8_ESM.zip › Figure 6/6D/pLJR965-Mra_1649 without induction.tiff]

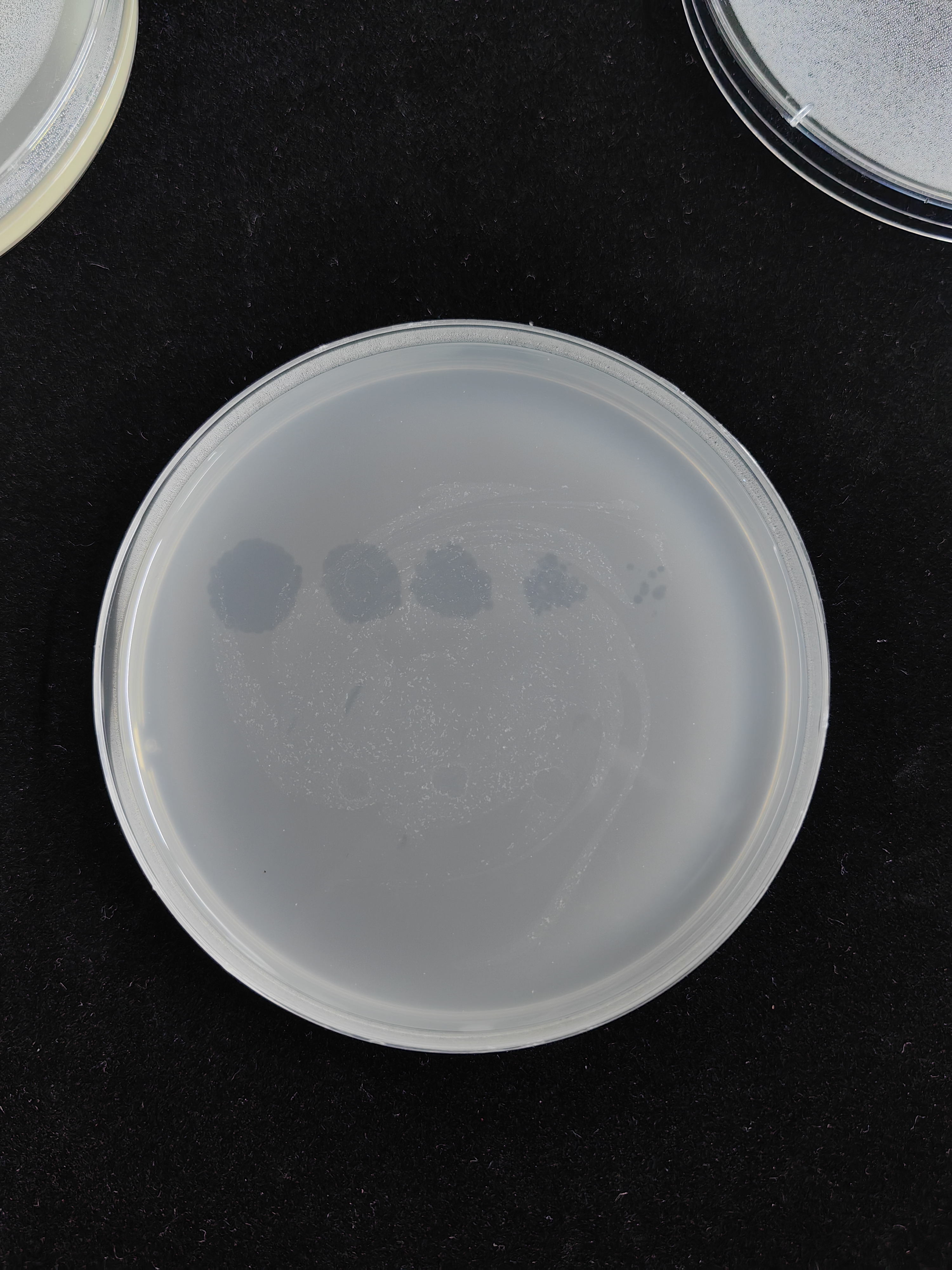

Supplement: Supplementary file 8 — Source data Fig. 6 [file 44319_2025_488_MOESM8_ESM.zip › Figure 6/6D/pLJR965-Mra_1940A with induction.tiff]

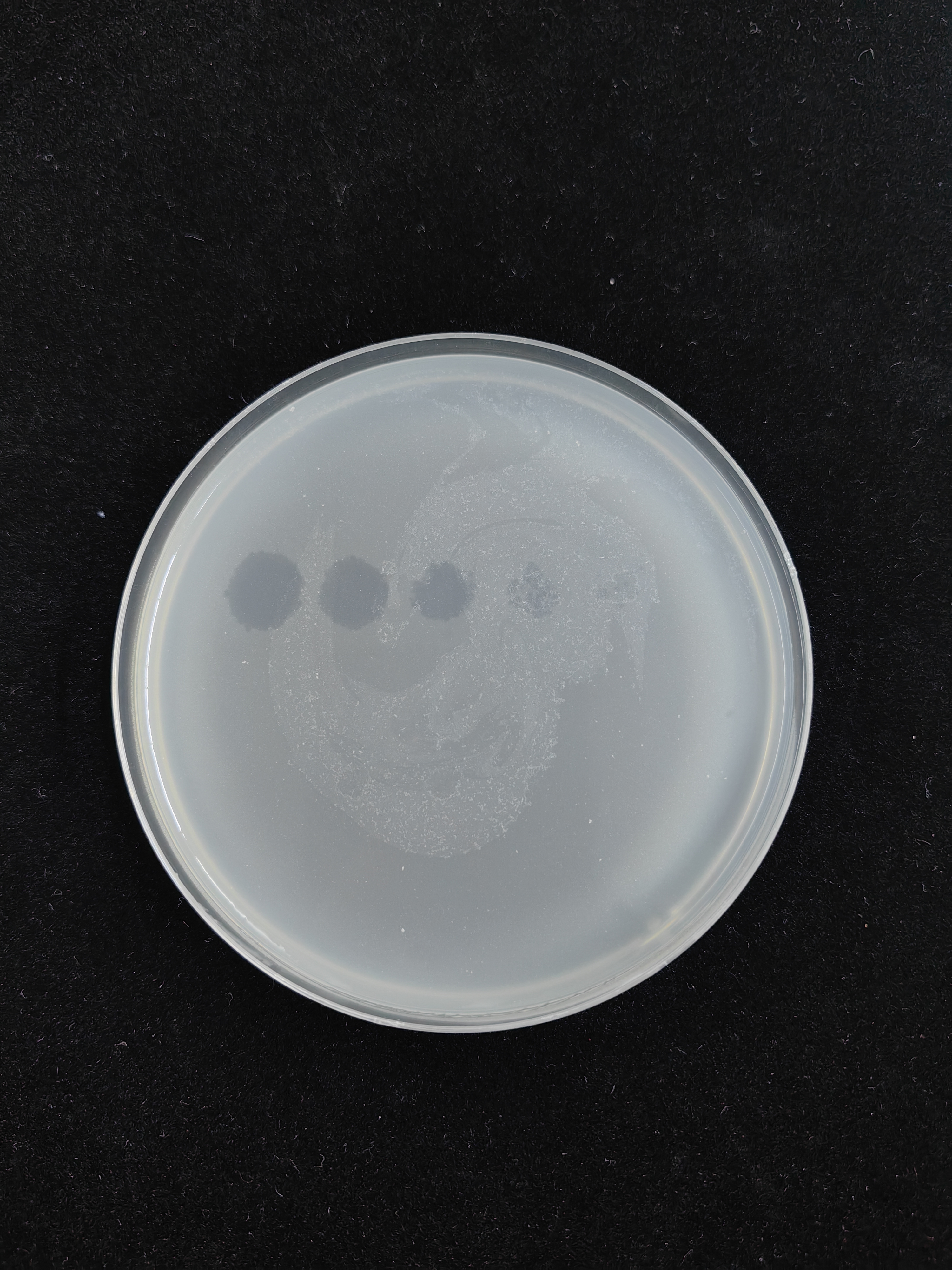

Supplement: Supplementary file 8 — Source data Fig. 6 [file 44319_2025_488_MOESM8_ESM.zip › Figure 6/6D/pLJR965-Mra_1940A without induction.tiff]

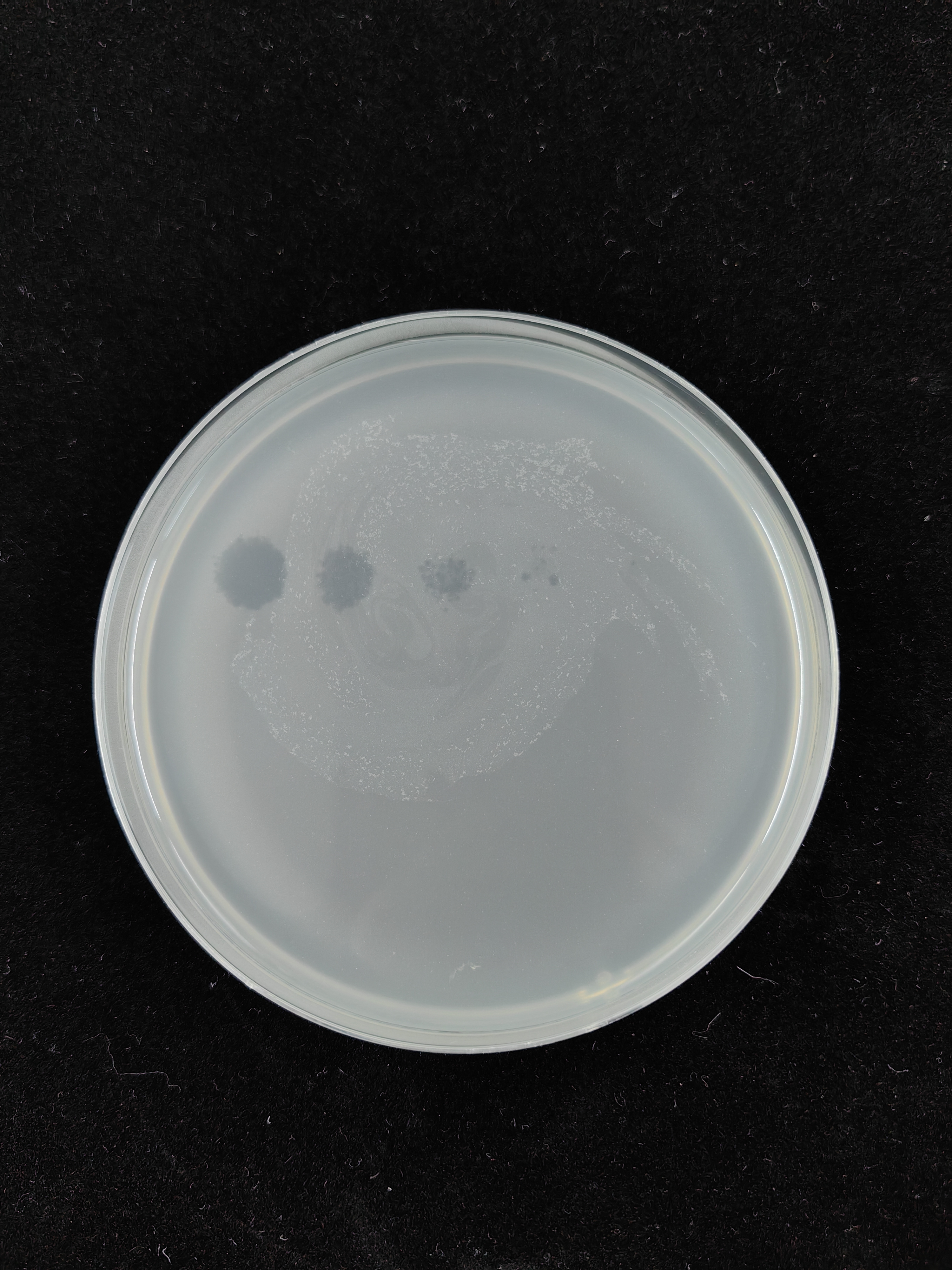

Supplement: Supplementary file 8 — Source data Fig. 6 [file 44319_2025_488_MOESM8_ESM.zip › Figure 6/6D/pLJR965-Mra_2329A with induction.tiff]

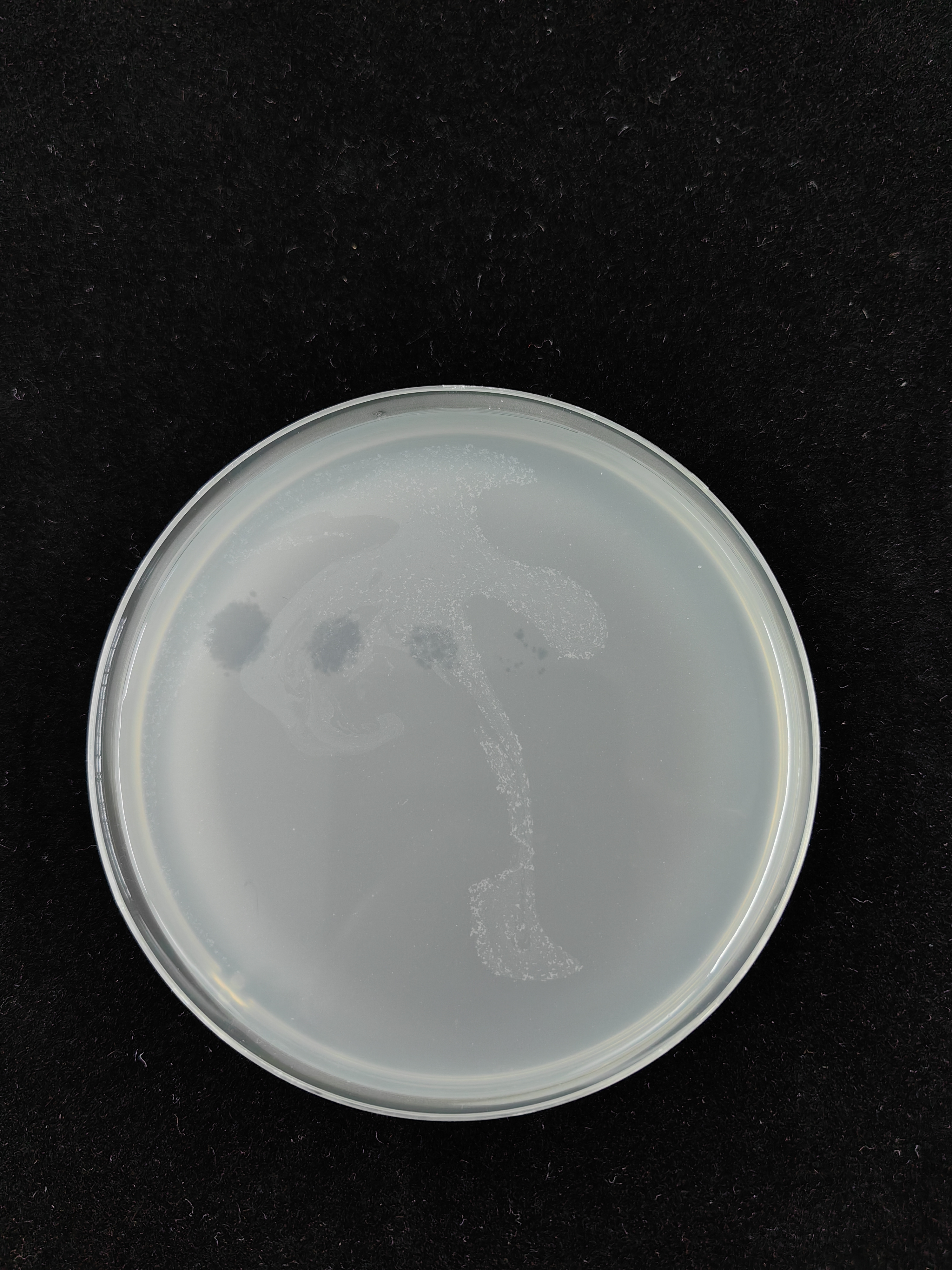

Supplement: Supplementary file 8 — Source data Fig. 6 [file 44319_2025_488_MOESM8_ESM.zip › Figure 6/6D/pLJR965-Mra_2329A without induction.tiff]

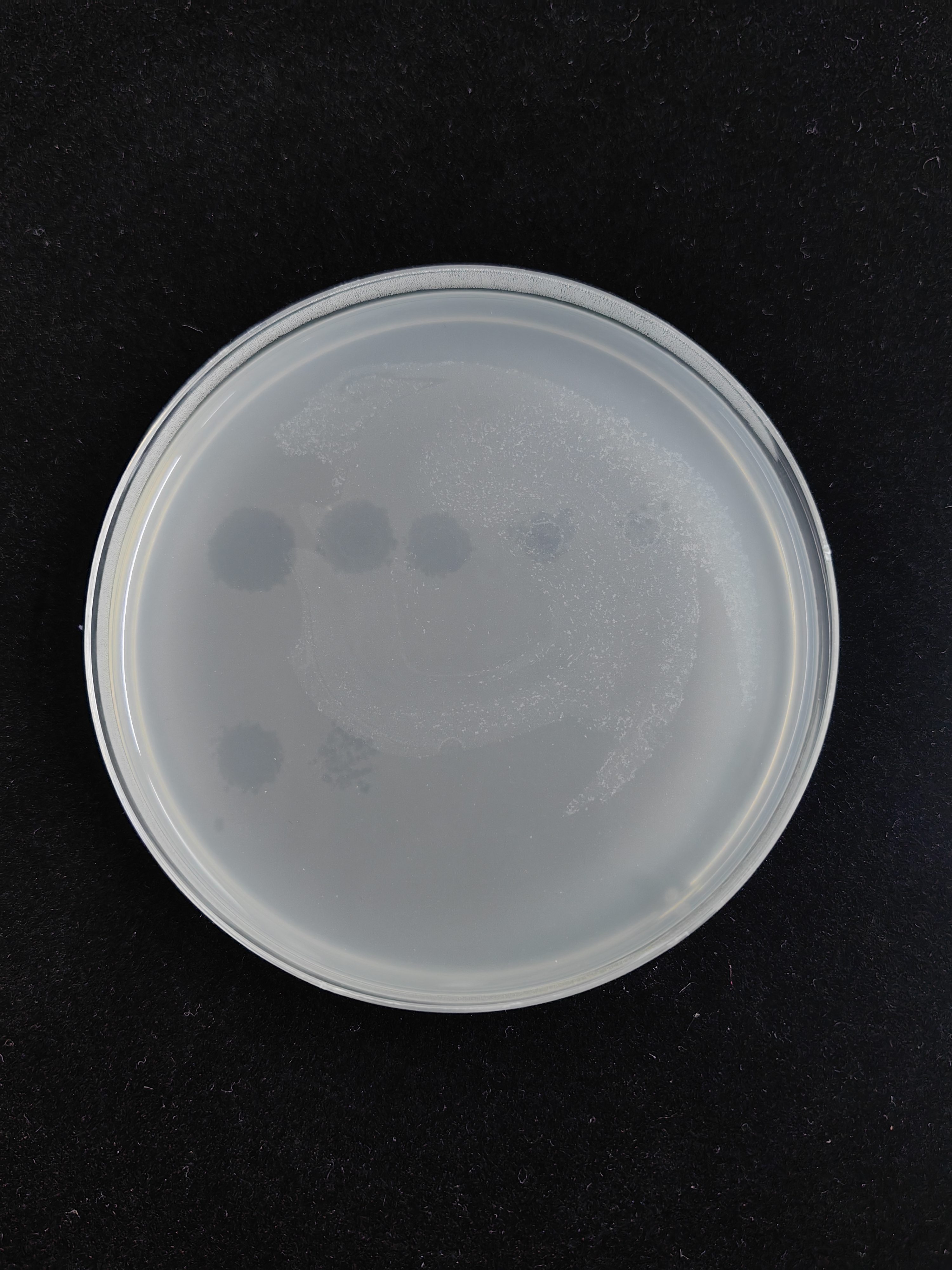

Supplement: Supplementary file 8 — Source data Fig. 6 [file 44319_2025_488_MOESM8_ESM.zip › Figure 6/6D/pLJR965-Mra_3122 with induction.tiff]

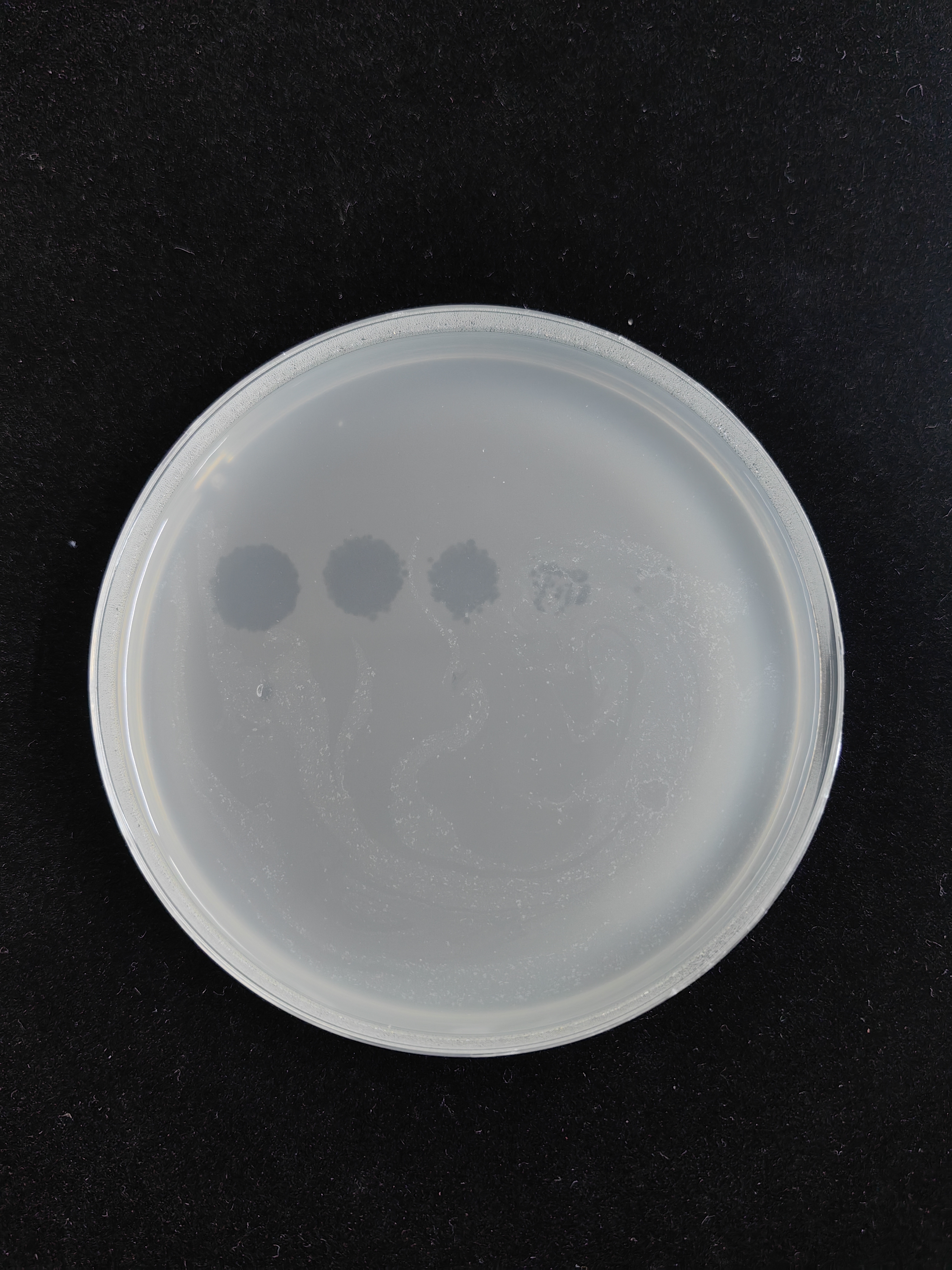

Supplement: Supplementary file 8 — Source data Fig. 6 [file 44319_2025_488_MOESM8_ESM.zip › Figure 6/6D/pLJR965-Mra_3122 without induction.tiff]

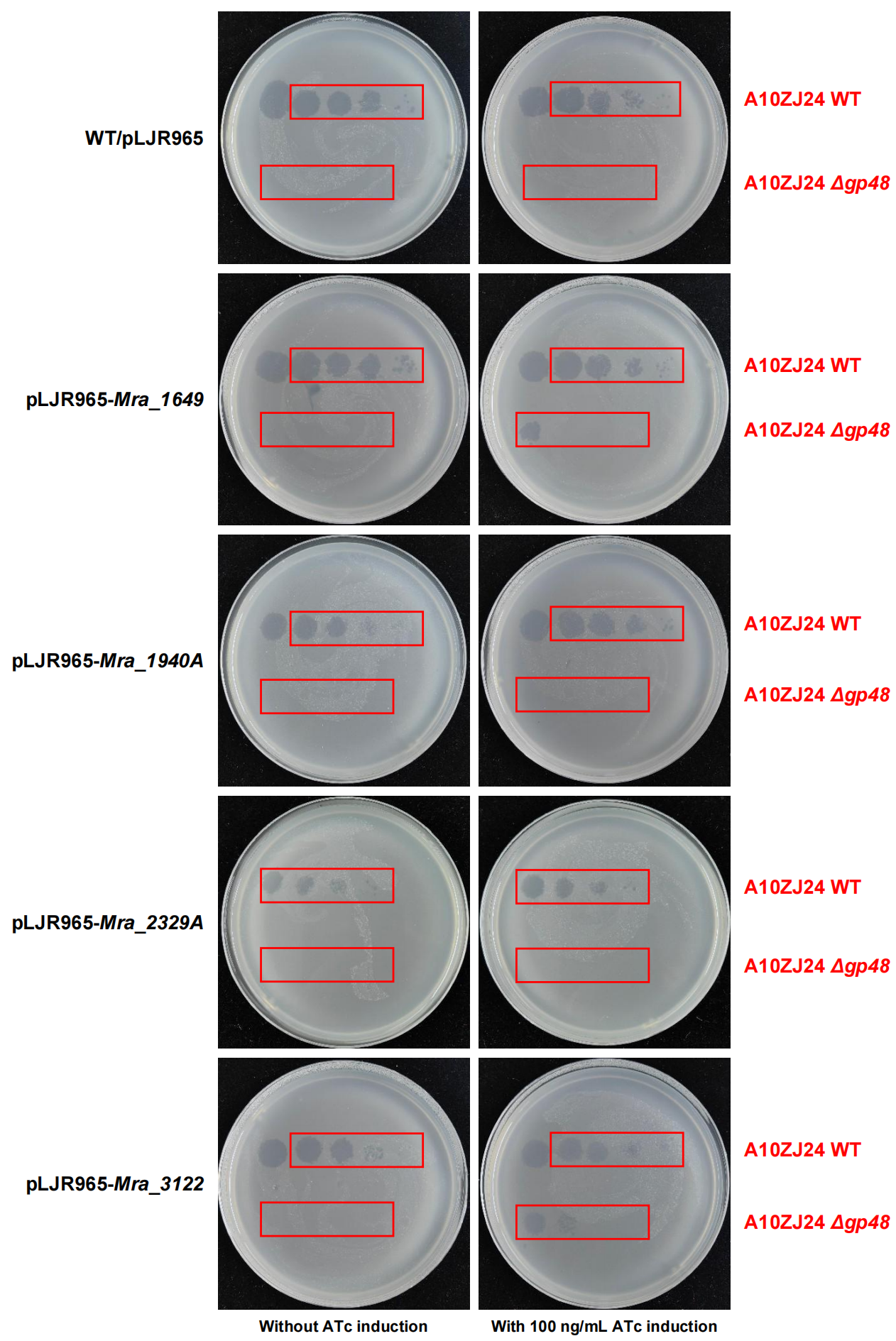

Supplement: Supplementary file 8 — Source data Fig. 6 [file 44319_2025_488_MOESM8_ESM.zip › Figure 6/6D/README.tif]

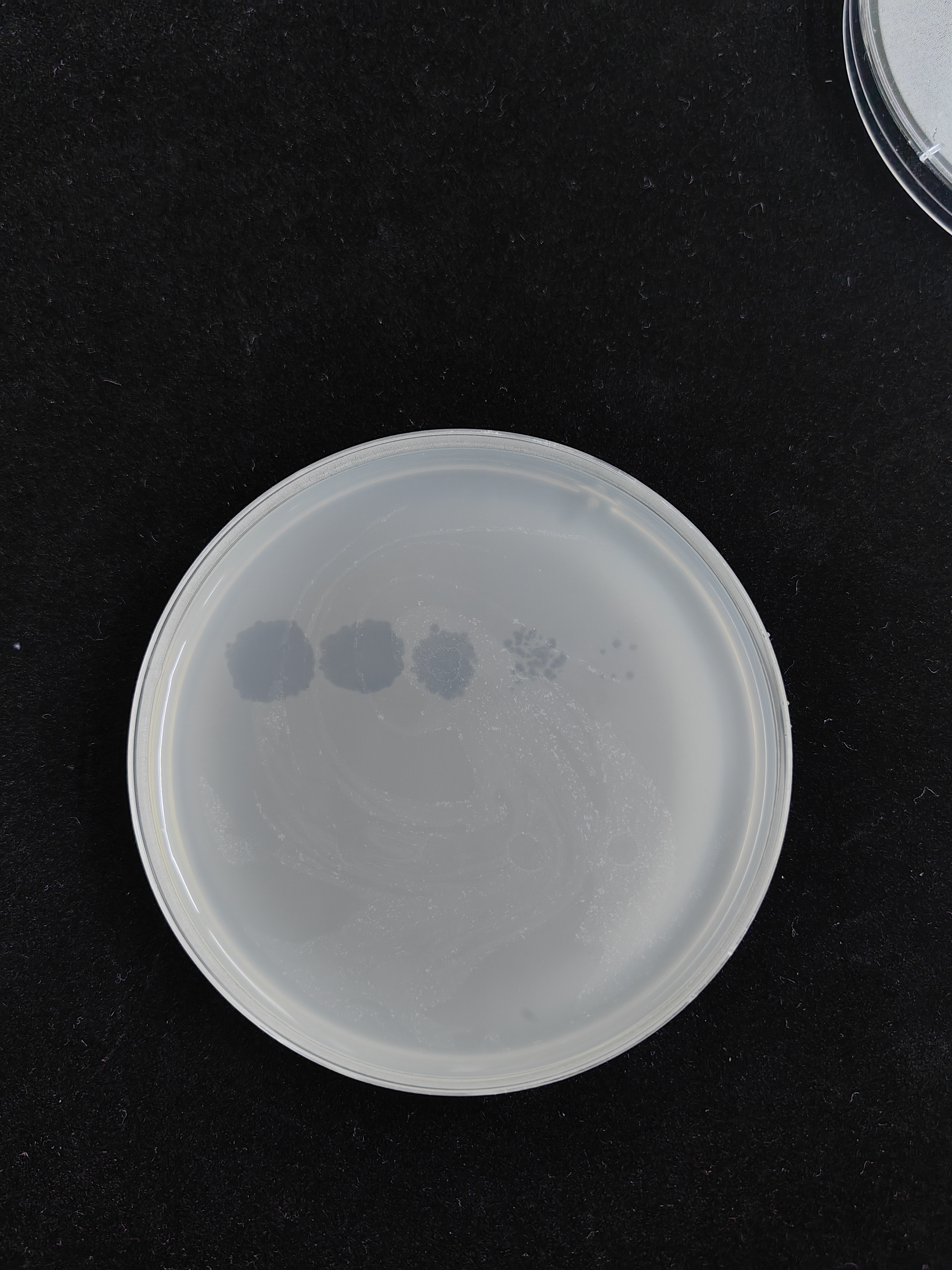

Supplement: Supplementary file 8 — Source data Fig. 6 [file 44319_2025_488_MOESM8_ESM.zip › Figure 6/6D/WT-pLJR965 with induction.tiff]

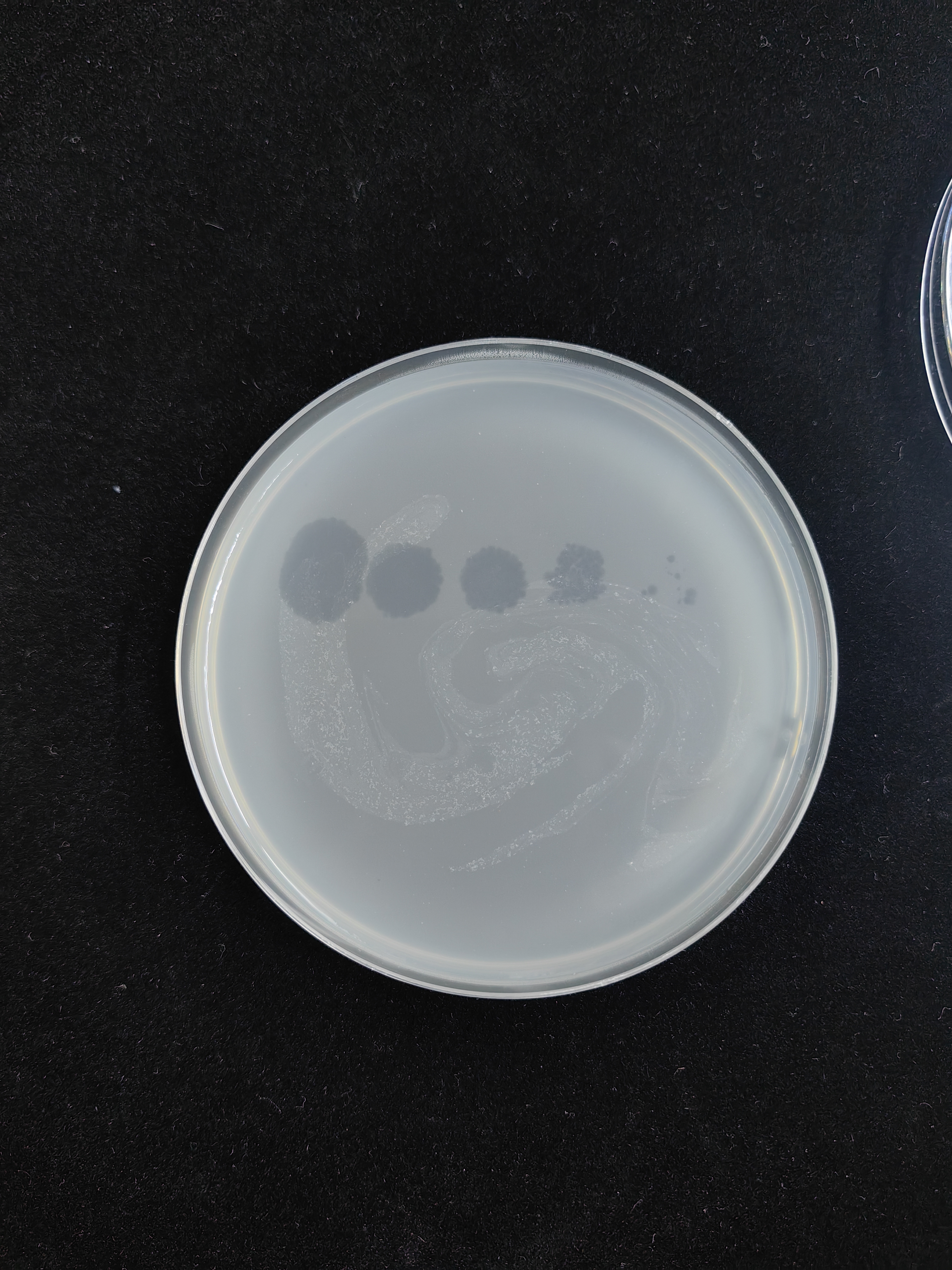

Supplement: Supplementary file 8 — Source data Fig. 6 [file 44319_2025_488_MOESM8_ESM.zip › Figure 6/6D/WT-pLJR965 without induction.tiff]

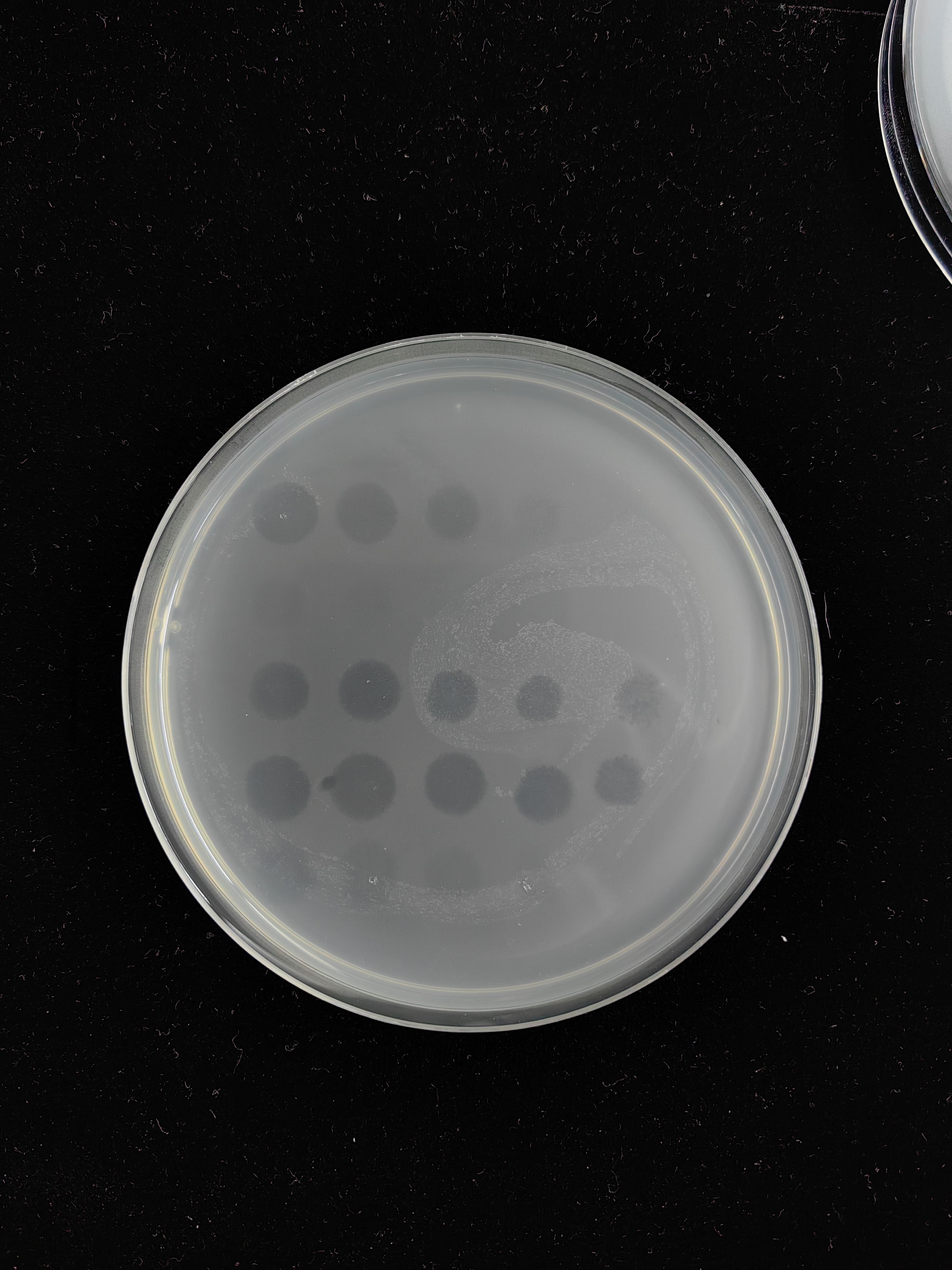

Supplement: Supplementary file 9 — Source data Fig. 7 [file 44319_2025_488_MOESM9_ESM.zip › Figure 7/7B/pJR962-1.tiff]

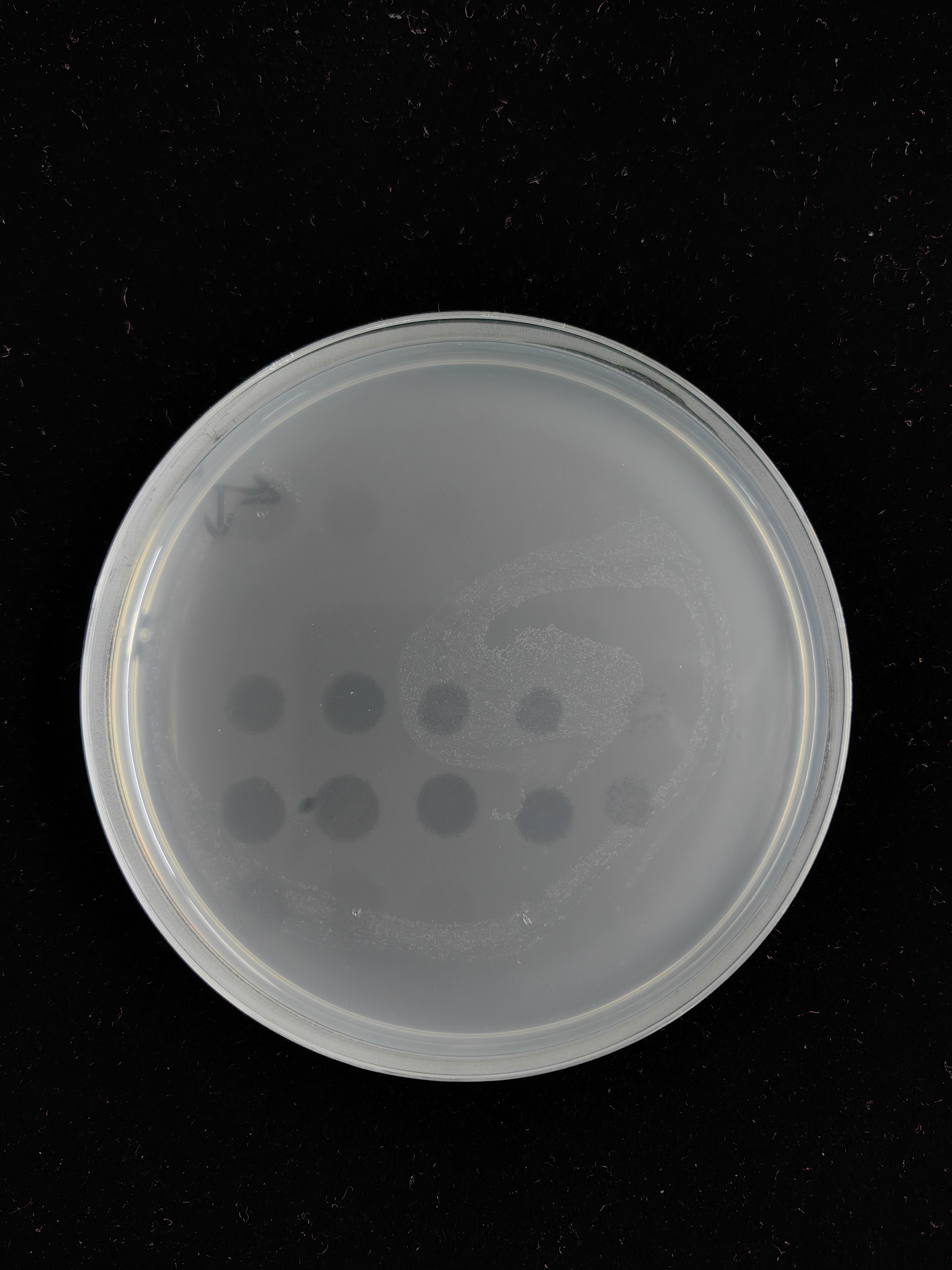

Supplement: Supplementary file 9 — Source data Fig. 7 [file 44319_2025_488_MOESM9_ESM.zip › Figure 7/7B/pJR962-2.tiff]

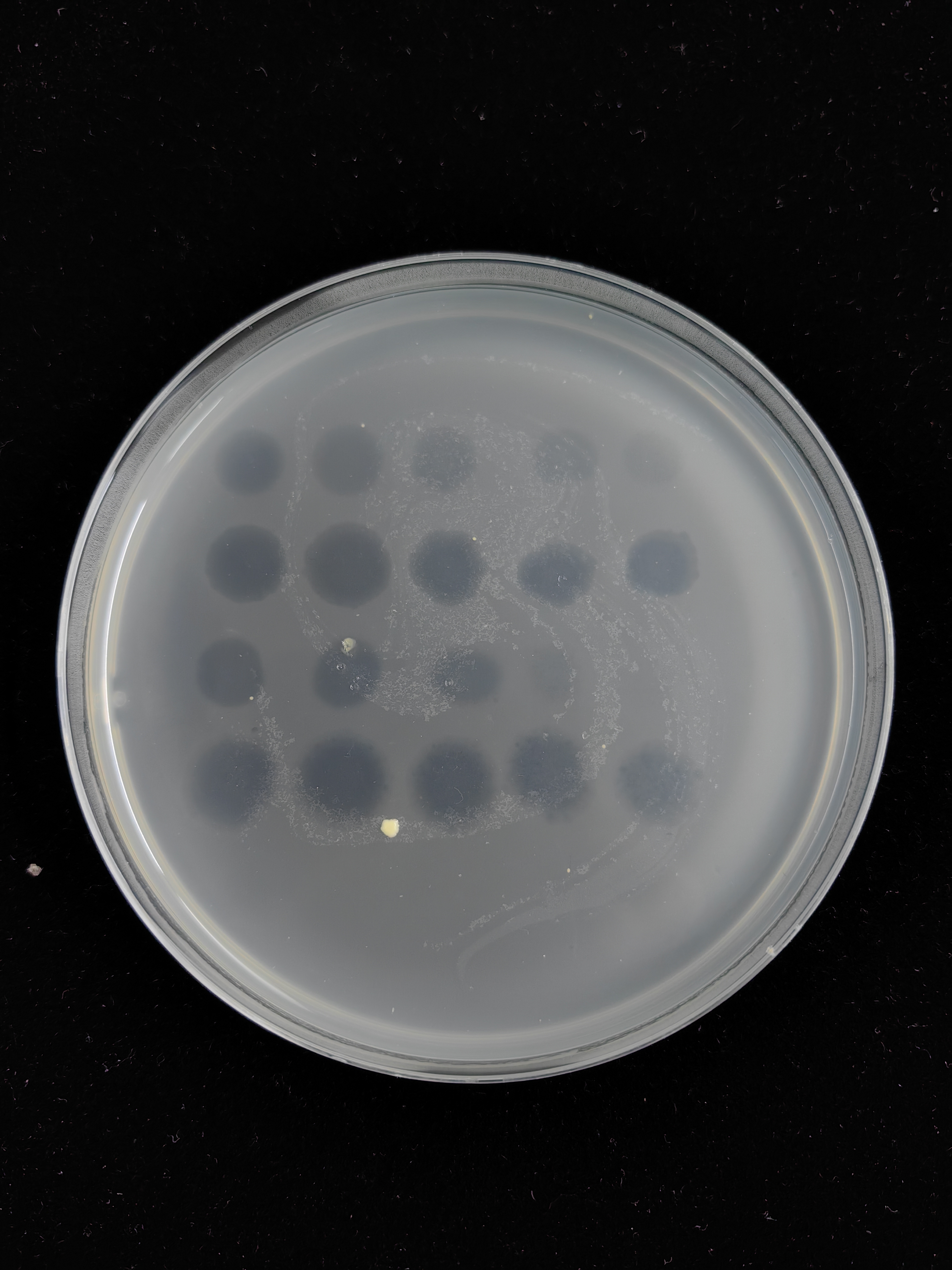

Supplement: Supplementary file 9 — Source data Fig. 7 [file 44319_2025_488_MOESM9_ESM.zip › Figure 7/7B/pJR962-3.tiff]

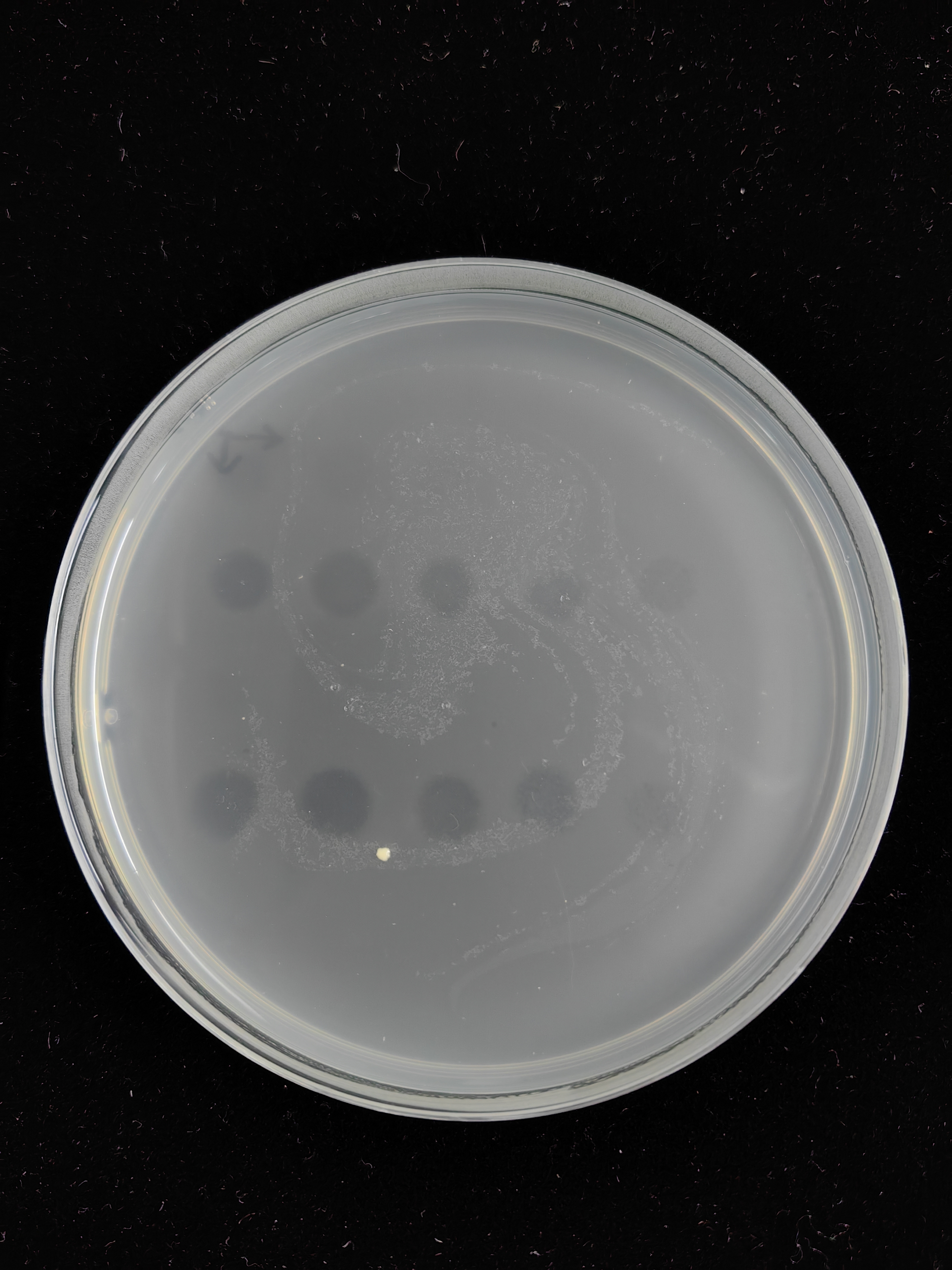

Supplement: Supplementary file 9 — Source data Fig. 7 [file 44319_2025_488_MOESM9_ESM.zip › Figure 7/7B/pJR962-4.tiff]

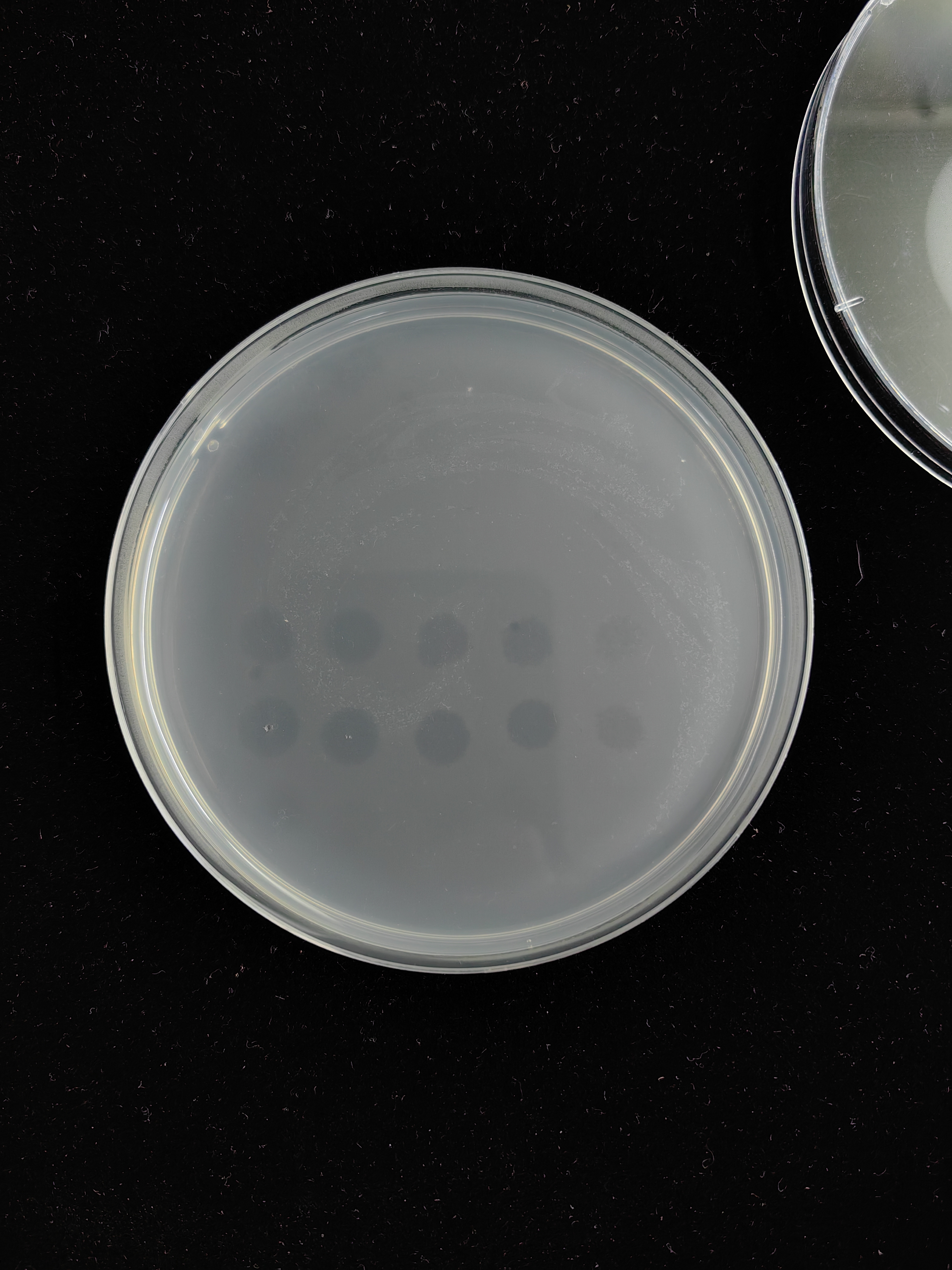

Supplement: Supplementary file 9 — Source data Fig. 7 [file 44319_2025_488_MOESM9_ESM.zip › Figure 7/7B/pJR962-Mra3122-1.tiff]

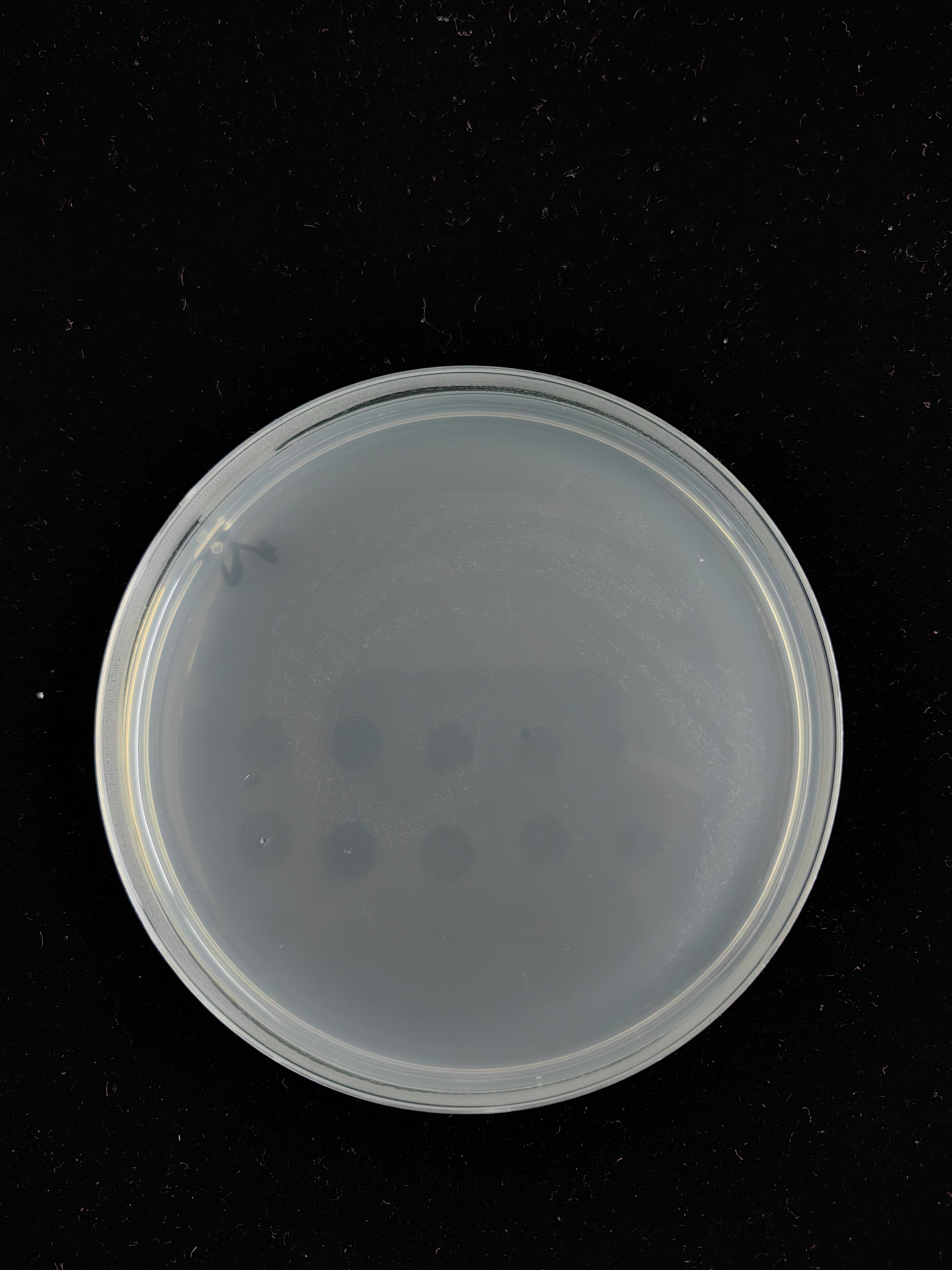

Supplement: Supplementary file 9 — Source data Fig. 7 [file 44319_2025_488_MOESM9_ESM.zip › Figure 7/7B/pJR962-Mra3122-2.tiff]

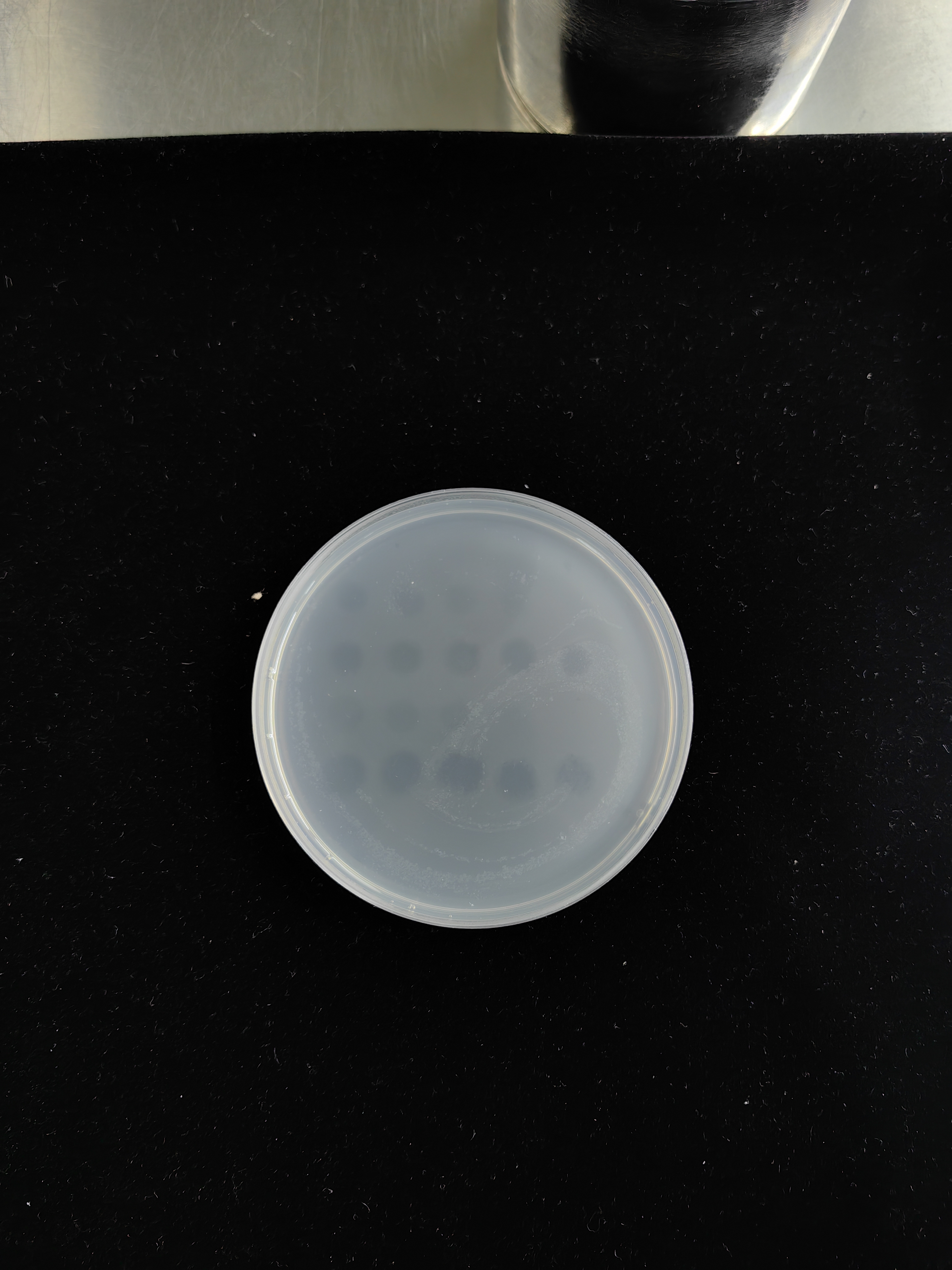

Supplement: Supplementary file 9 — Source data Fig. 7 [file 44319_2025_488_MOESM9_ESM.zip › Figure 7/7B/pJR962-Mra3122-3.tiff]

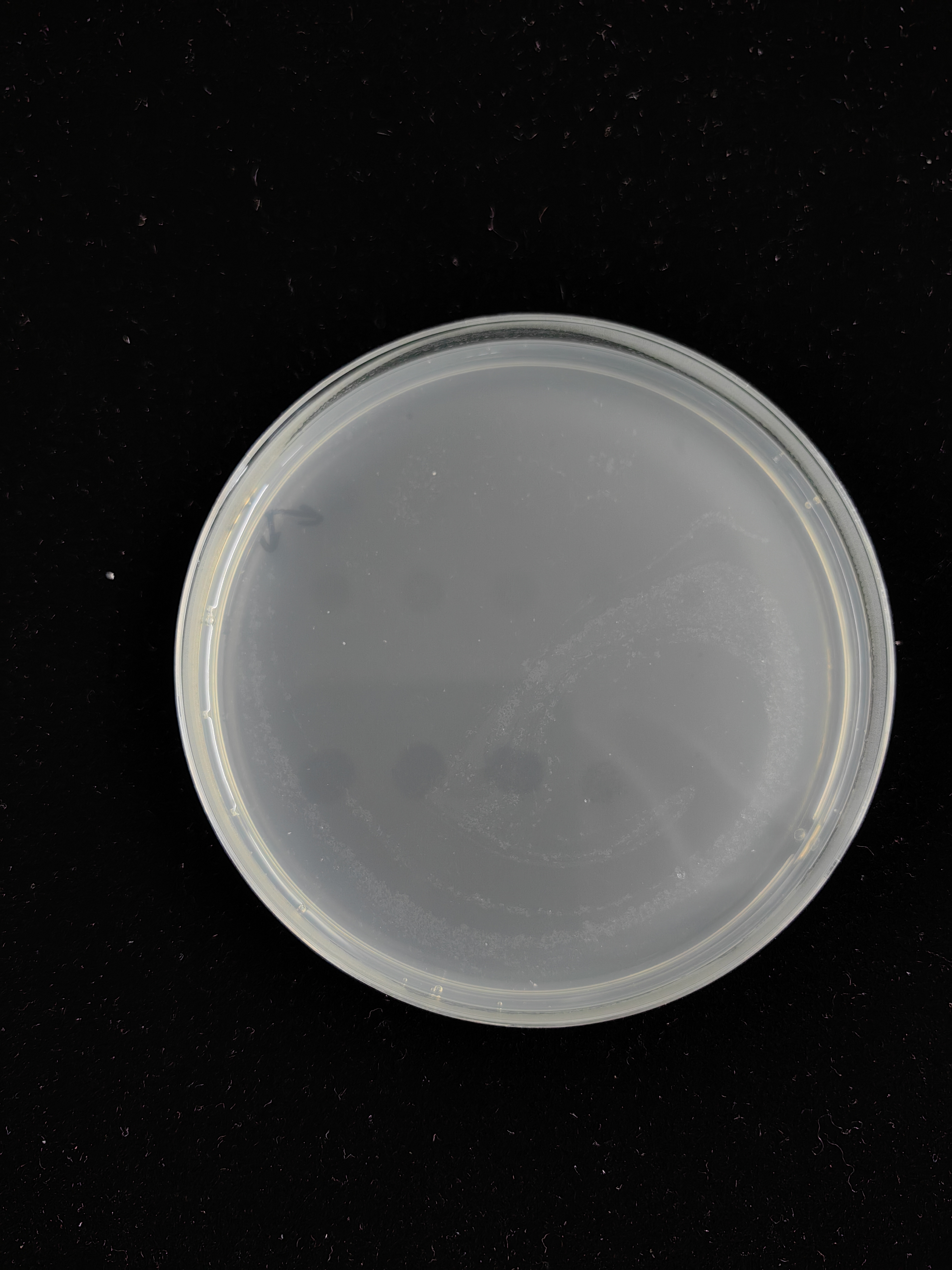

Supplement: Supplementary file 9 — Source data Fig. 7 [file 44319_2025_488_MOESM9_ESM.zip › Figure 7/7B/pJR962-Mra3122-4.tiff]

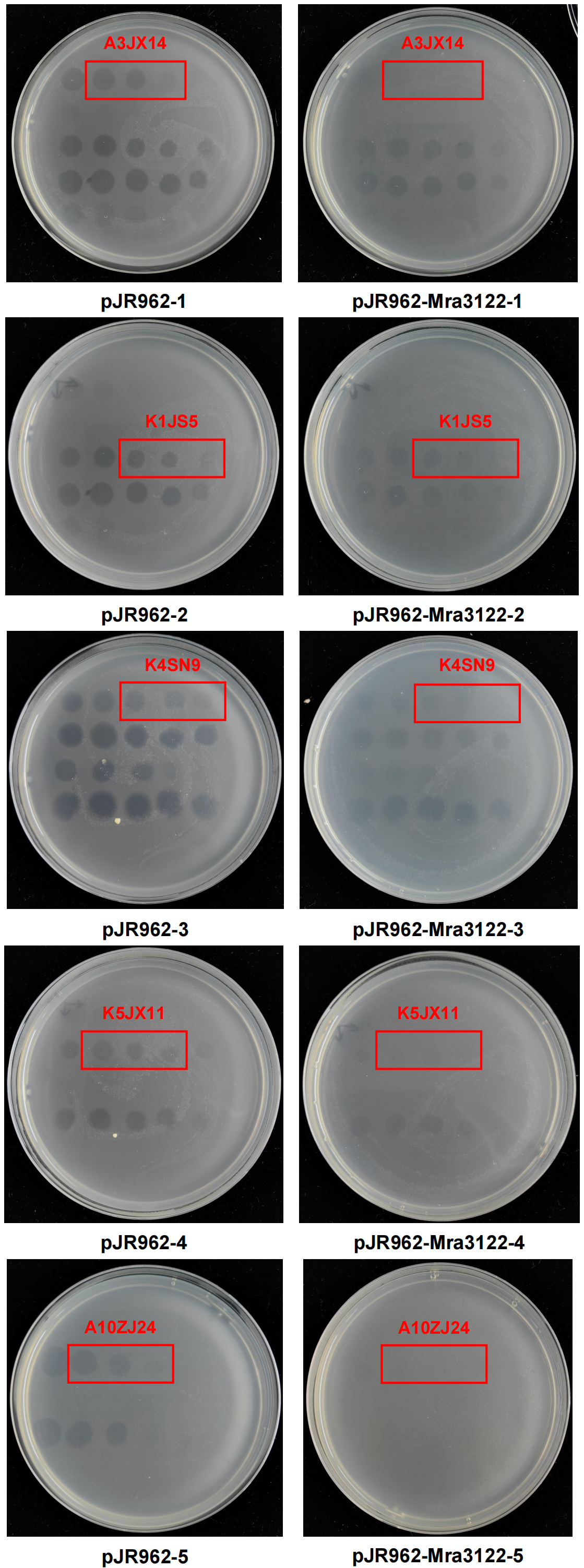

Supplement: Supplementary file 9 — Source data Fig. 7 [file 44319_2025_488_MOESM9_ESM.zip › Figure 7/7B/README.tif]

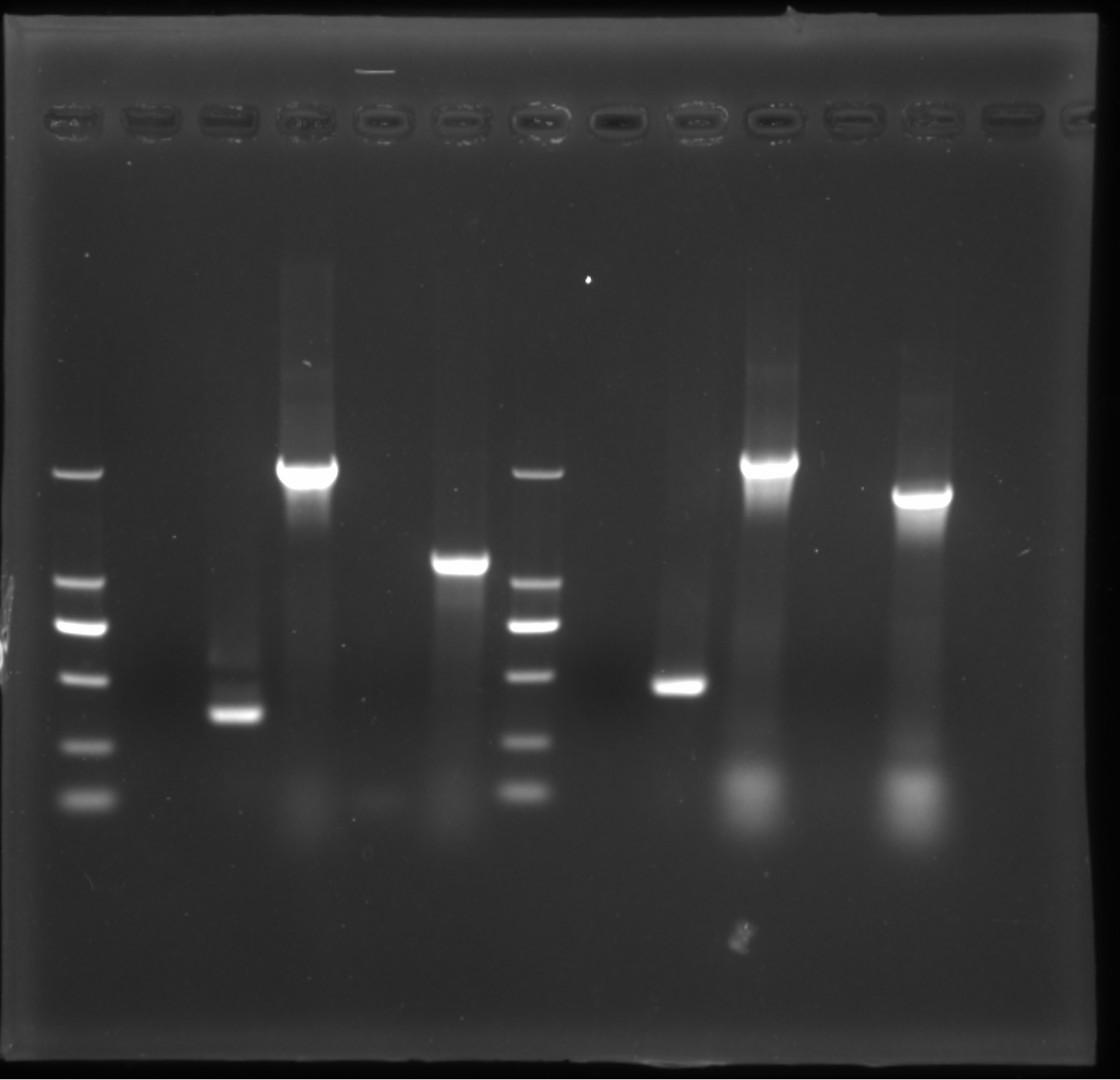

Supplement: Supplementary file 10 — Figure S2-5 Source Data [file 44319_2025_488_MOESM10_ESM.zip › Figure S2-S5_Source Data/Appendix Figure S2/S2B/A10ZJ24 Δgp32, Δgp40.tif]

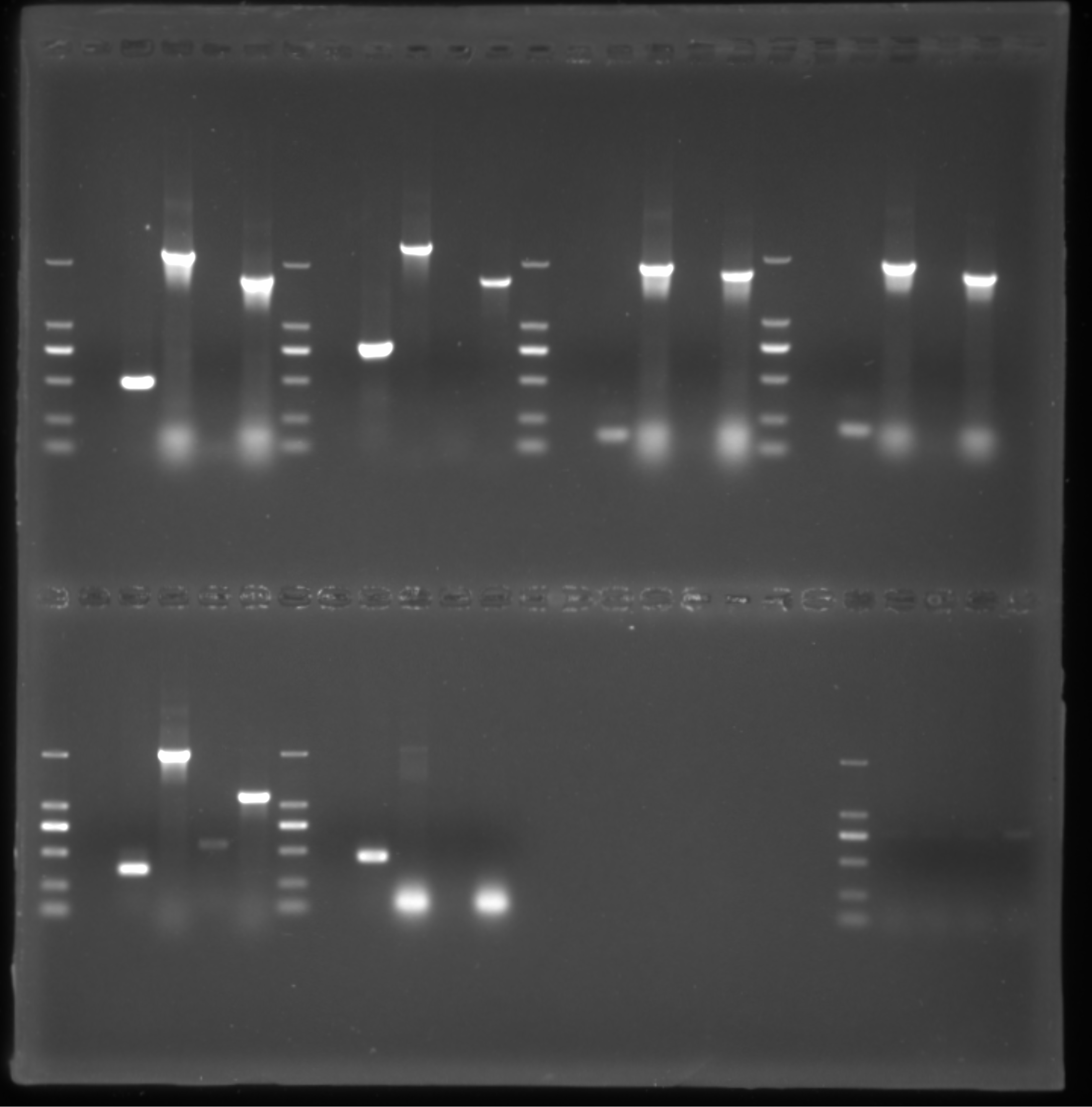

Supplement: Supplementary file 10 — Figure S2-5 Source Data [file 44319_2025_488_MOESM10_ESM.zip › Figure S2-S5_Source Data/Appendix Figure S2/S2B/A10ZJ24 Δgp41, Δgp46, Δgp48, Δgp53.tif]

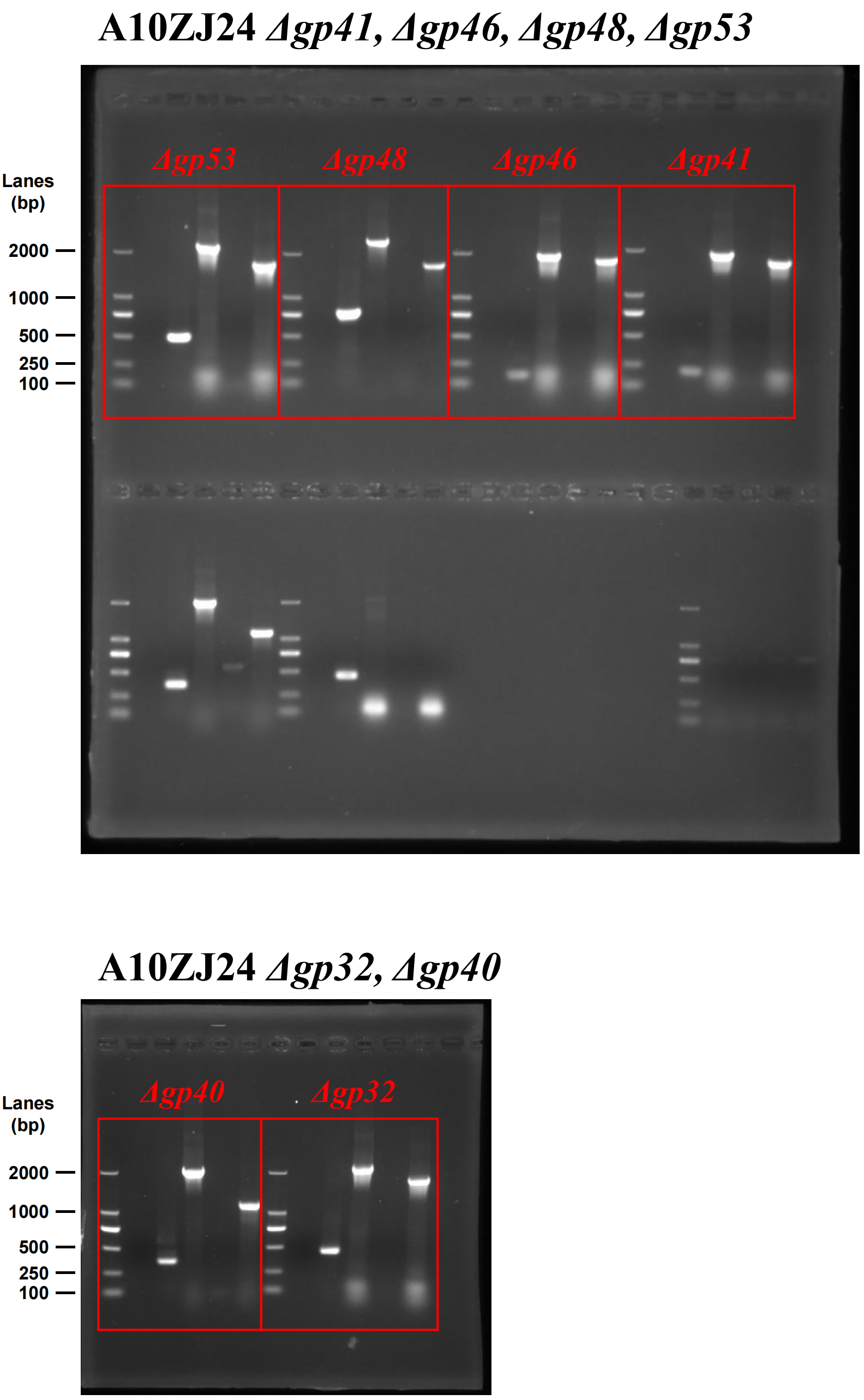

Supplement: Supplementary file 10 — Figure S2-5 Source Data [file 44319_2025_488_MOESM10_ESM.zip › Figure S2-S5_Source Data/Appendix Figure S2/S2B/README.tif]

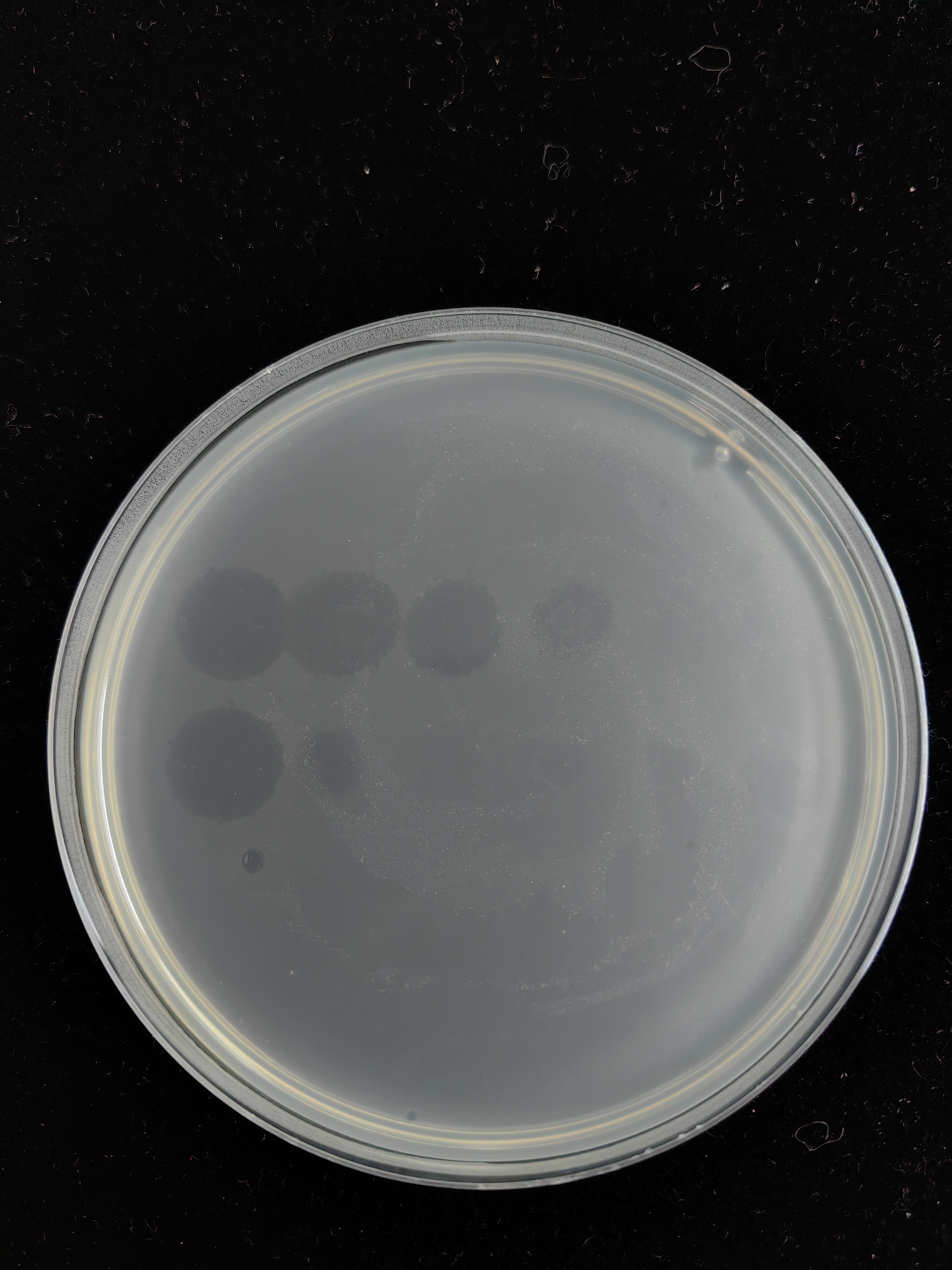

Supplement: Supplementary file 10 — Figure S2-5 Source Data [file 44319_2025_488_MOESM10_ESM.zip › Figure S2-S5_Source Data/Appendix Figure S4/S4A/pJR962-A10ZJ24 gp48 (A113E) with ATc induction.tiff]

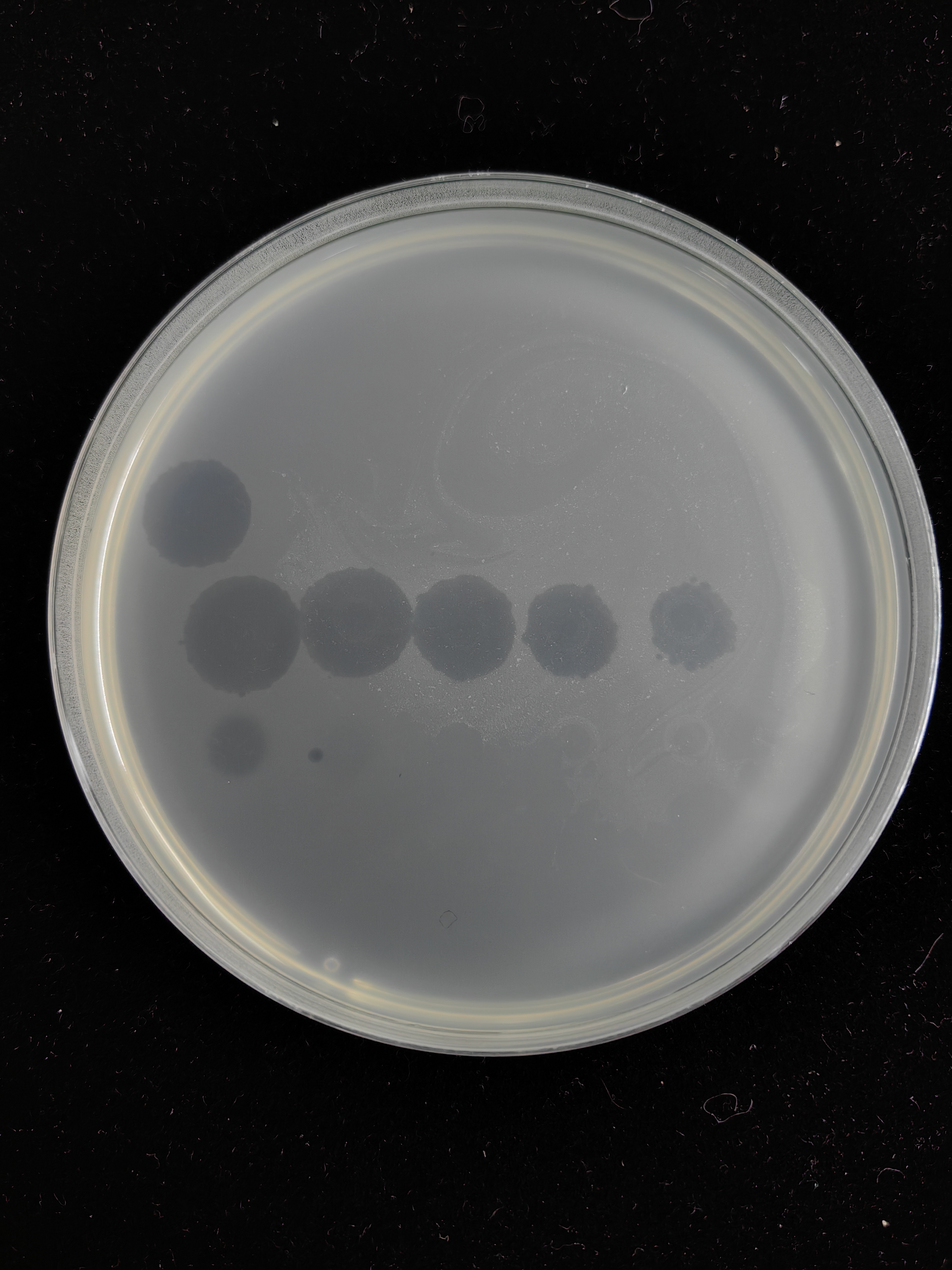

Supplement: Supplementary file 10 — Figure S2-5 Source Data [file 44319_2025_488_MOESM10_ESM.zip › Figure S2-S5_Source Data/Appendix Figure S4/S4A/pJR962-A10ZJ24 gp48 (A113E) without ATc induction.tiff]

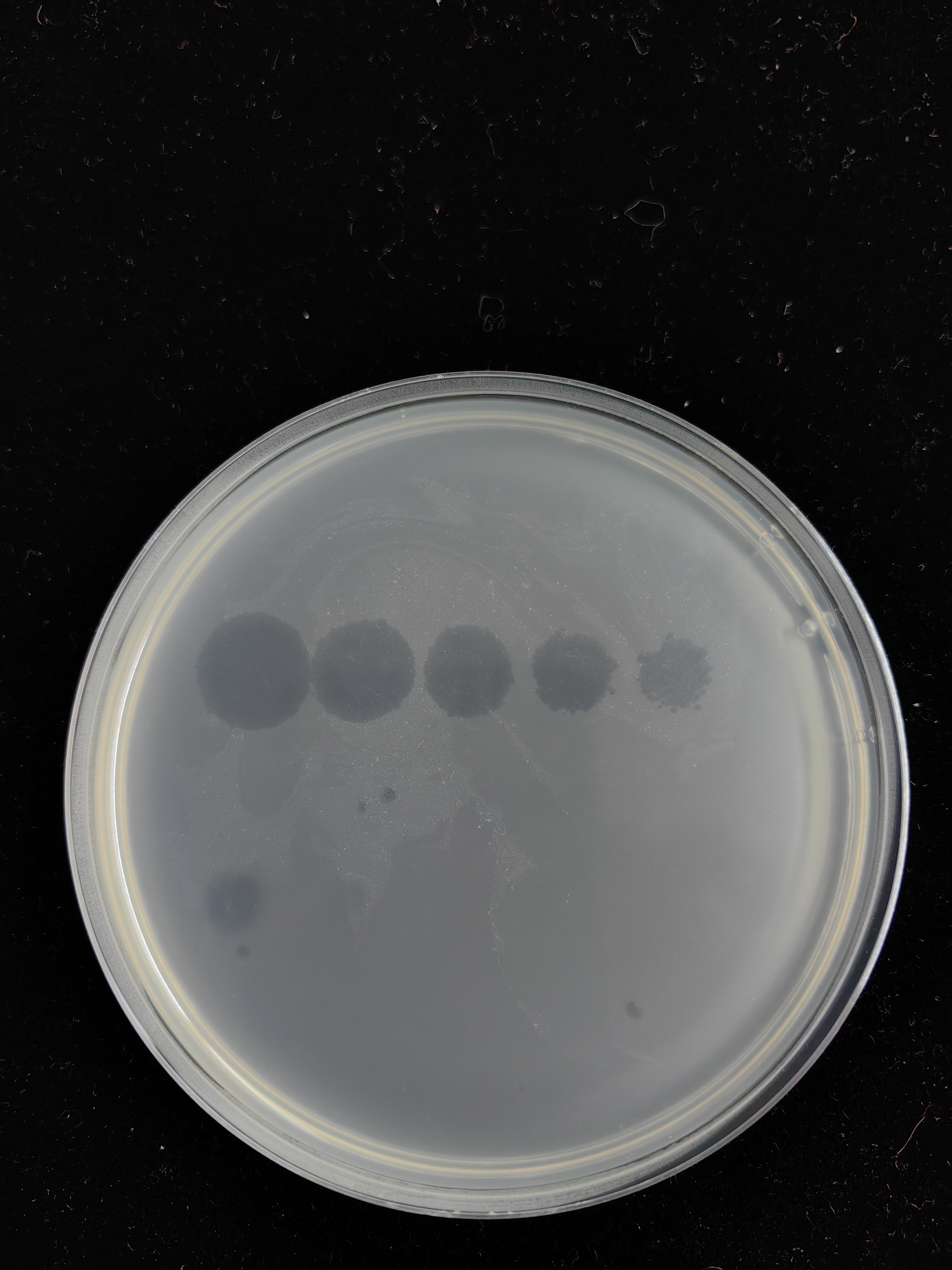

Supplement: Supplementary file 10 — Figure S2-5 Source Data [file 44319_2025_488_MOESM10_ESM.zip › Figure S2-S5_Source Data/Appendix Figure S4/S4A/pJR962-A10ZJ24 gp48 (D10G) with ATc induction.tiff]

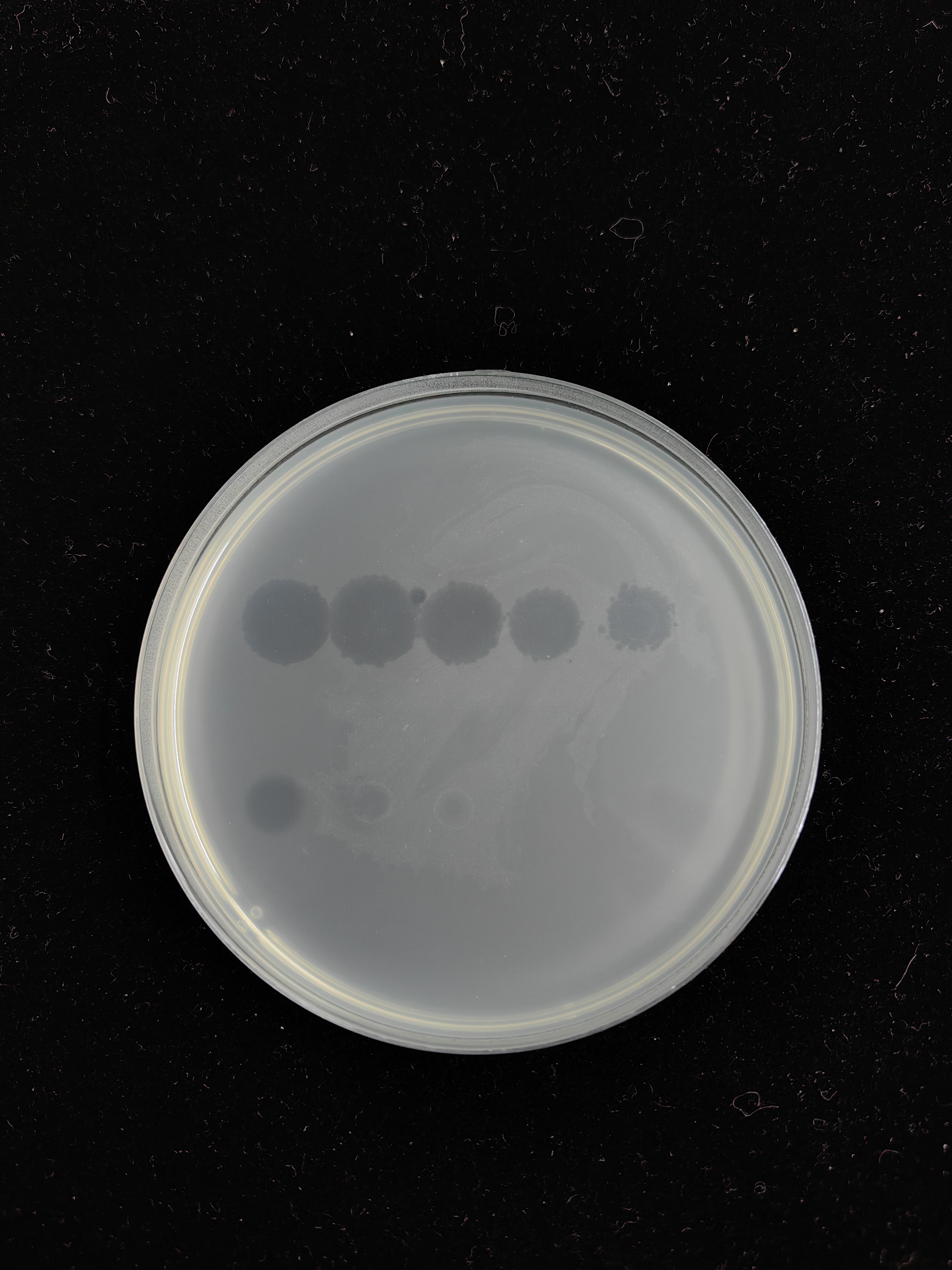

Supplement: Supplementary file 10 — Figure S2-5 Source Data [file 44319_2025_488_MOESM10_ESM.zip › Figure S2-S5_Source Data/Appendix Figure S4/S4A/pJR962-A10ZJ24 gp48 (D10G) without ATc induction.tiff]

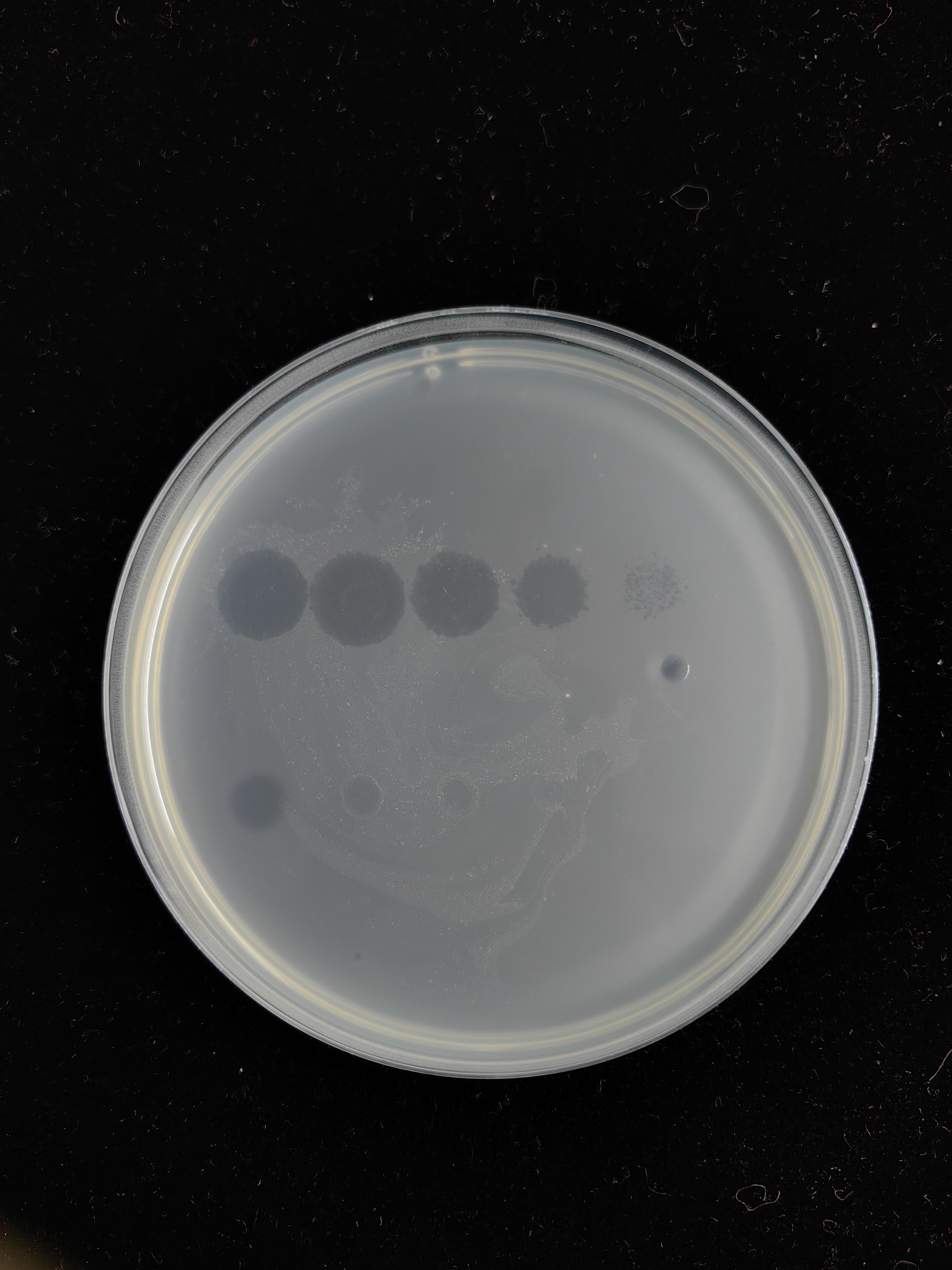

Supplement: Supplementary file 10 — Figure S2-5 Source Data [file 44319_2025_488_MOESM10_ESM.zip › Figure S2-S5_Source Data/Appendix Figure S4/S4A/pJR962-A10ZJ24 gp48 (D41V) with ATc induction.tiff]

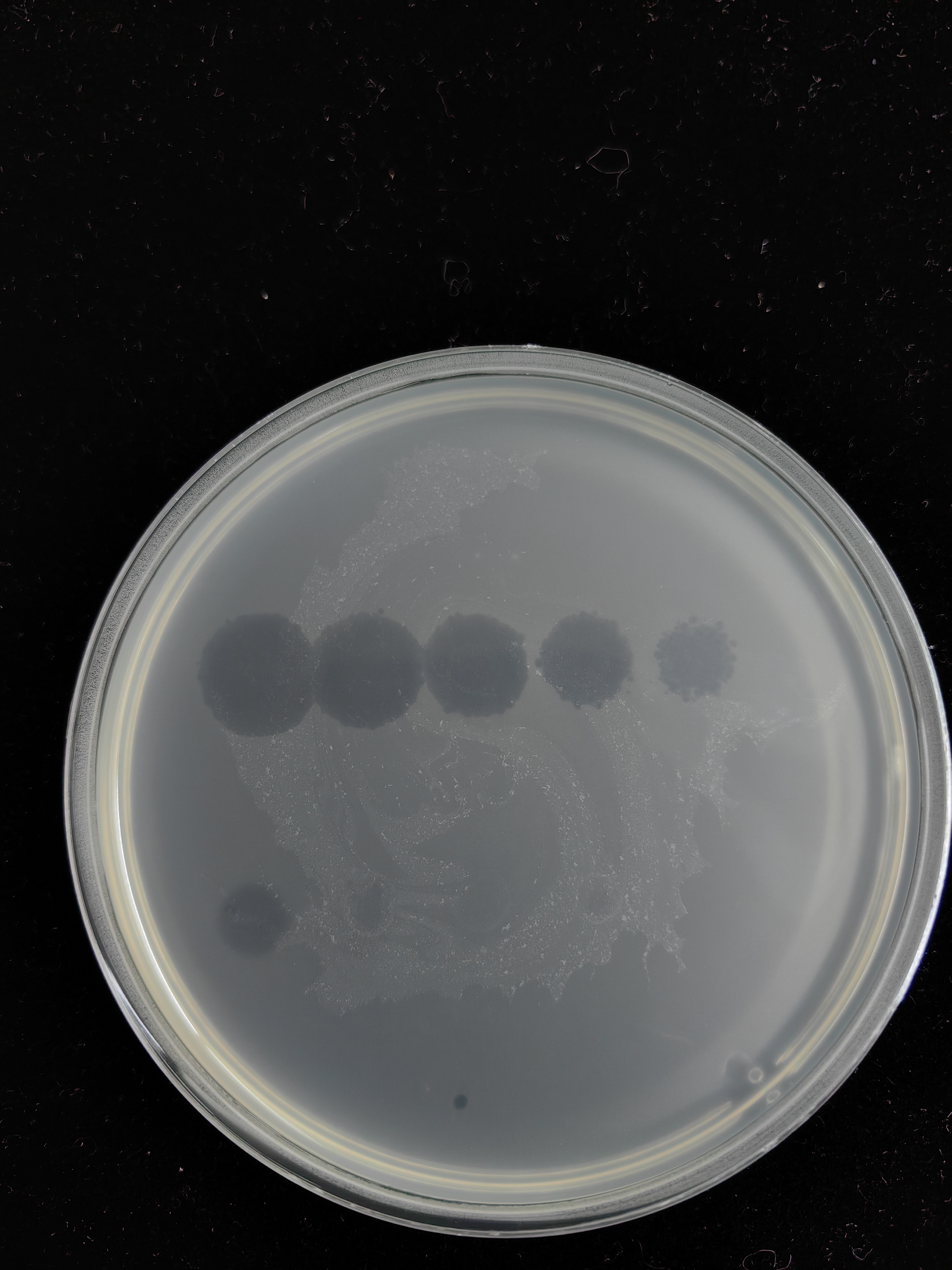

Supplement: Supplementary file 10 — Figure S2-5 Source Data [file 44319_2025_488_MOESM10_ESM.zip › Figure S2-S5_Source Data/Appendix Figure S4/S4A/pJR962-A10ZJ24 gp48 (D41V) without ATc induction.tiff]

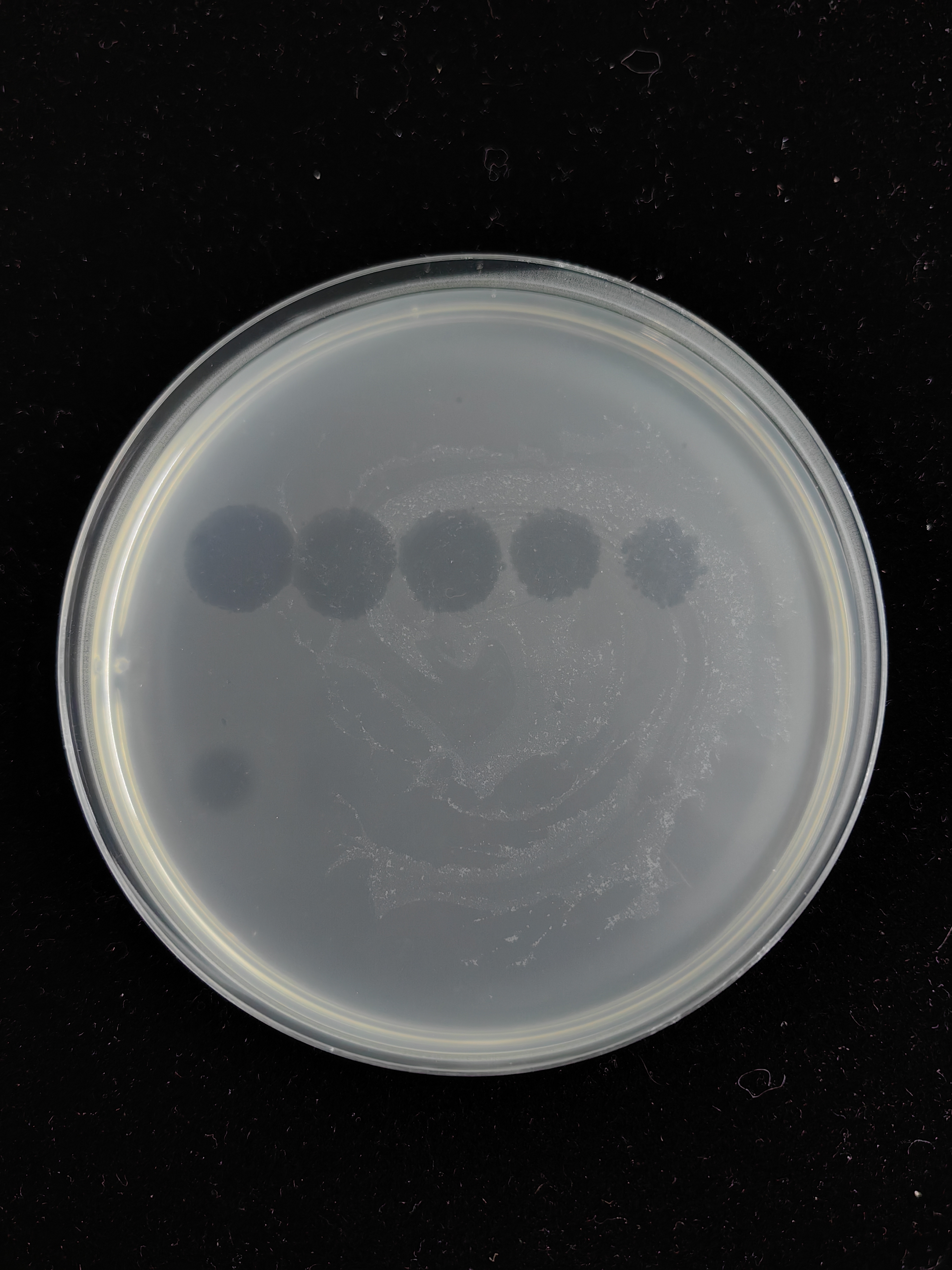

Supplement: Supplementary file 10 — Figure S2-5 Source Data [file 44319_2025_488_MOESM10_ESM.zip › Figure S2-S5_Source Data/Appendix Figure S4/S4A/pJR962-A10ZJ24 gp48 (H175D) with ATc induction.tiff]

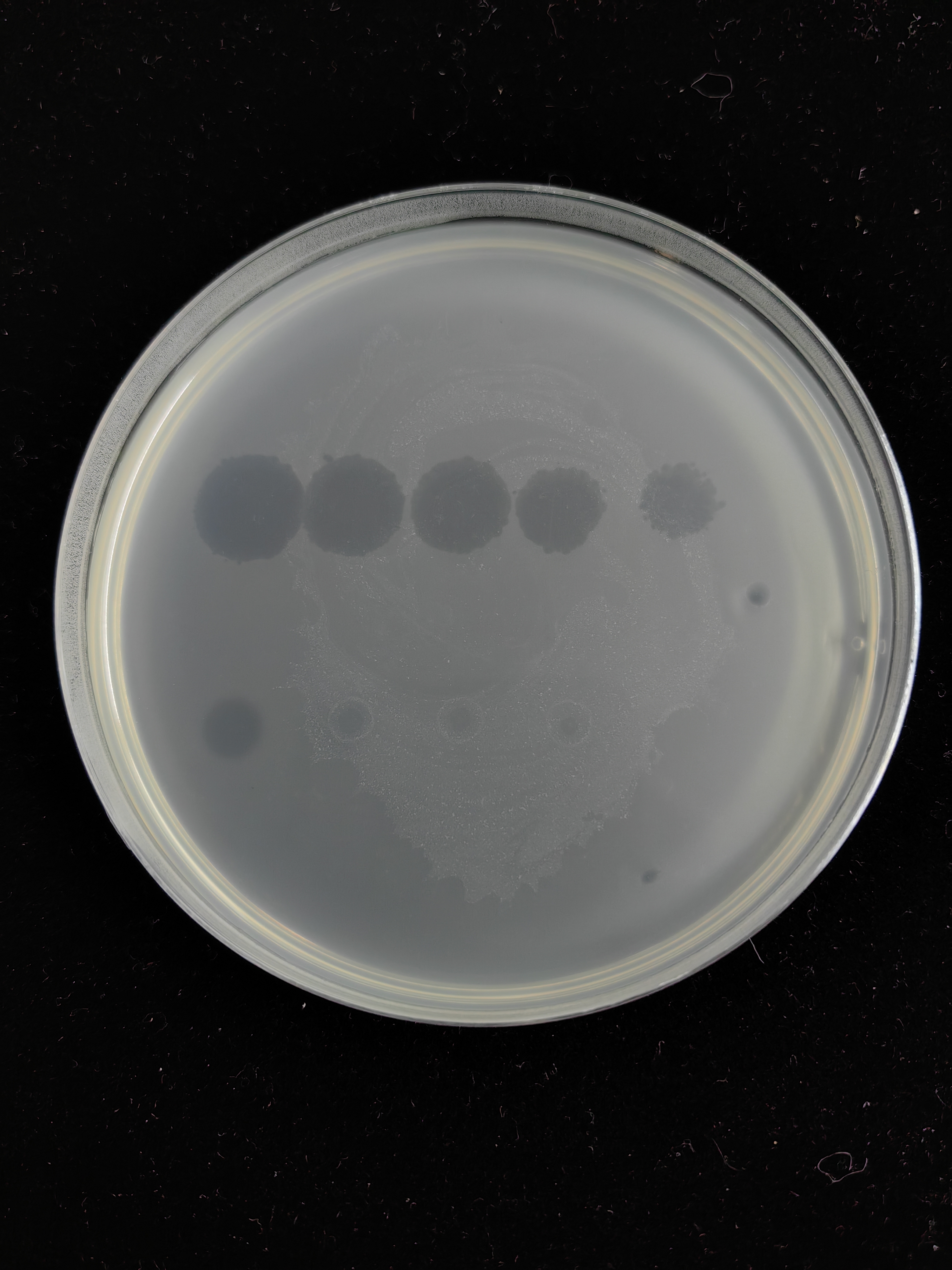

Supplement: Supplementary file 10 — Figure S2-5 Source Data [file 44319_2025_488_MOESM10_ESM.zip › Figure S2-S5_Source Data/Appendix Figure S4/S4A/pJR962-A10ZJ24 gp48 (H175D) without ATc induction.tiff]

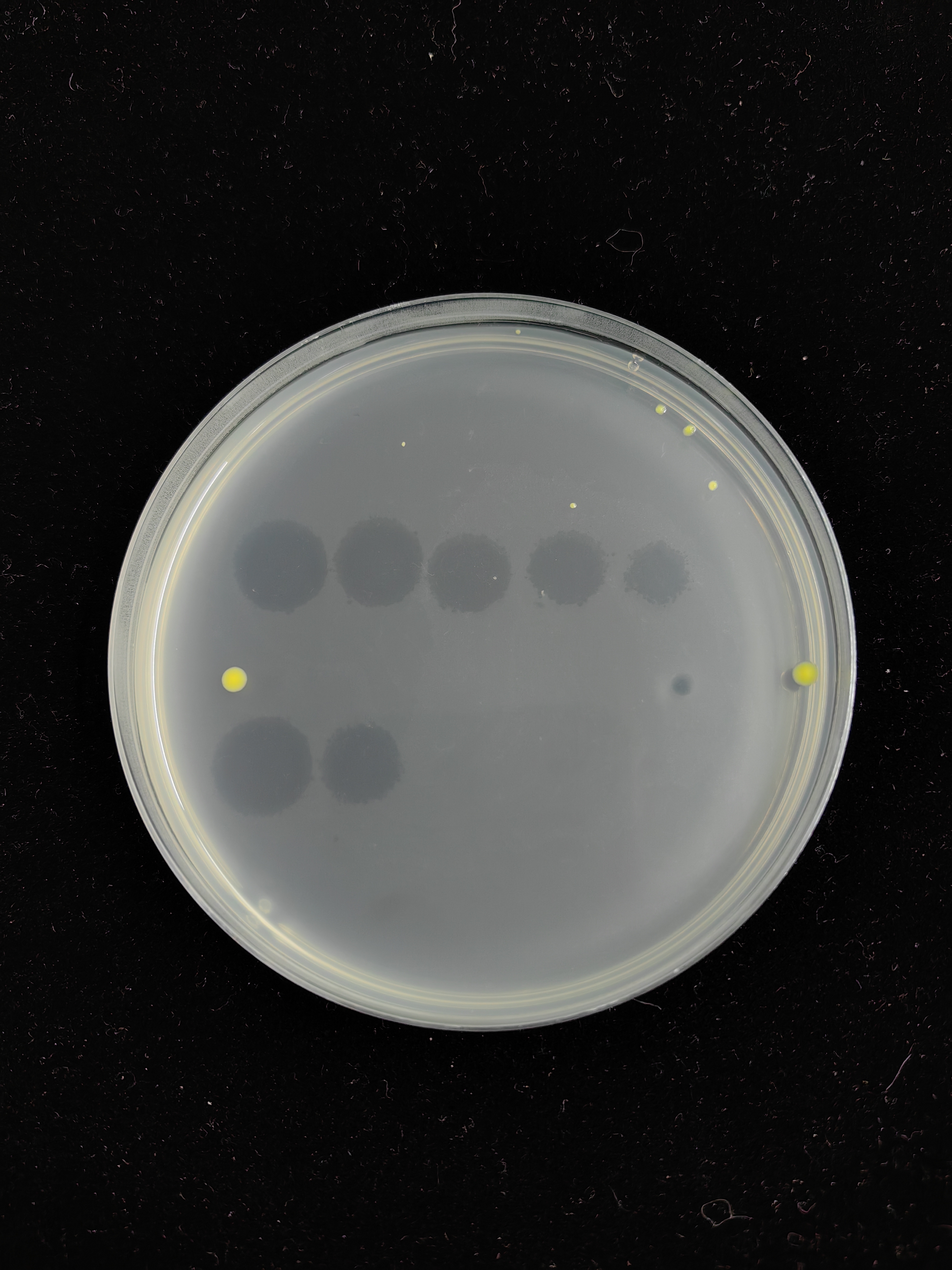

Supplement: Supplementary file 10 — Figure S2-5 Source Data [file 44319_2025_488_MOESM10_ESM.zip › Figure S2-S5_Source Data/Appendix Figure S4/S4A/pJR962-A10ZJ24 gp48 with ATc induction.tiff]

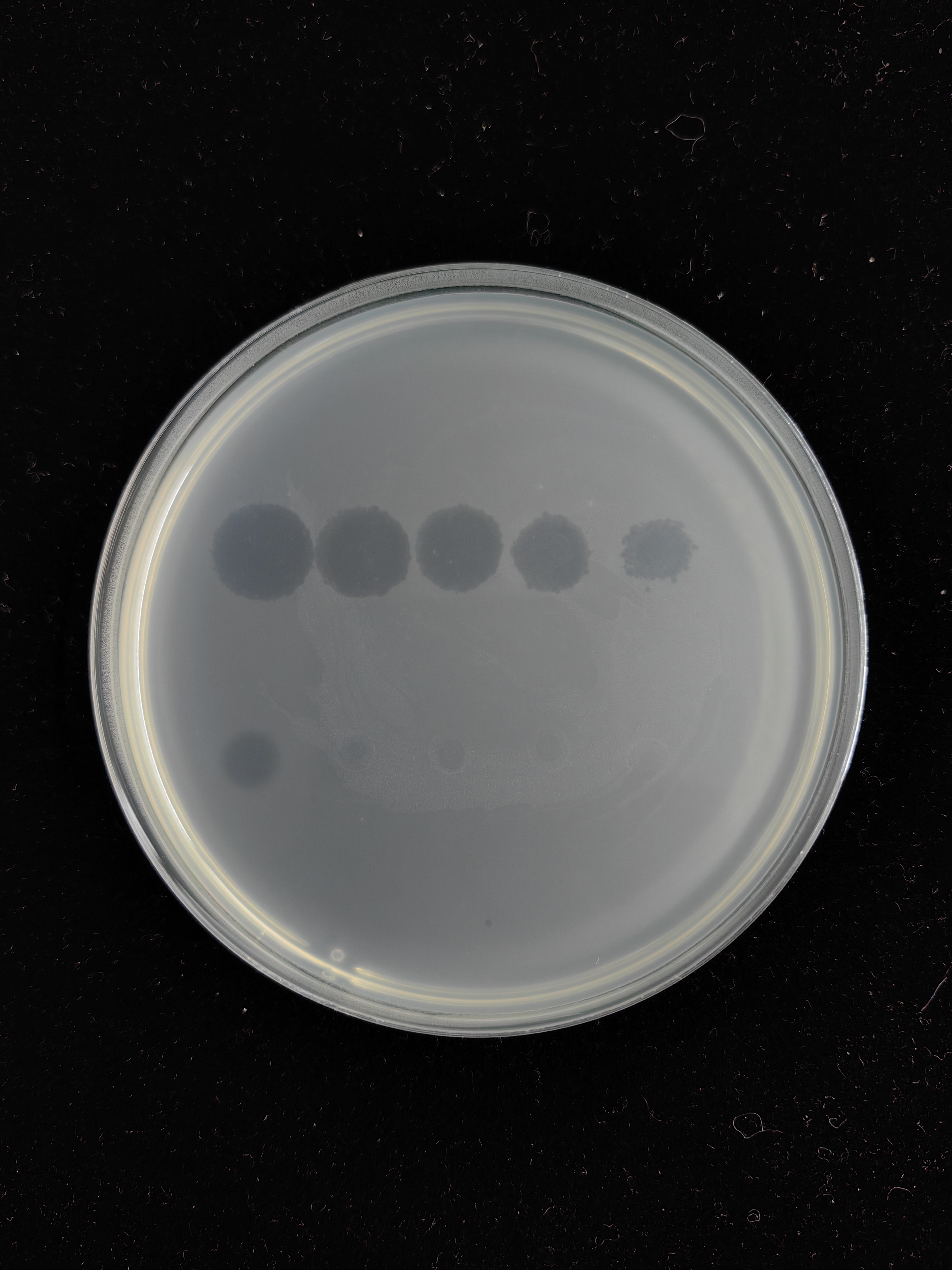

Supplement: Supplementary file 10 — Figure S2-5 Source Data [file 44319_2025_488_MOESM10_ESM.zip › Figure S2-S5_Source Data/Appendix Figure S4/S4A/pJR962-A10ZJ24 gp48 without ATc induction.tiff]

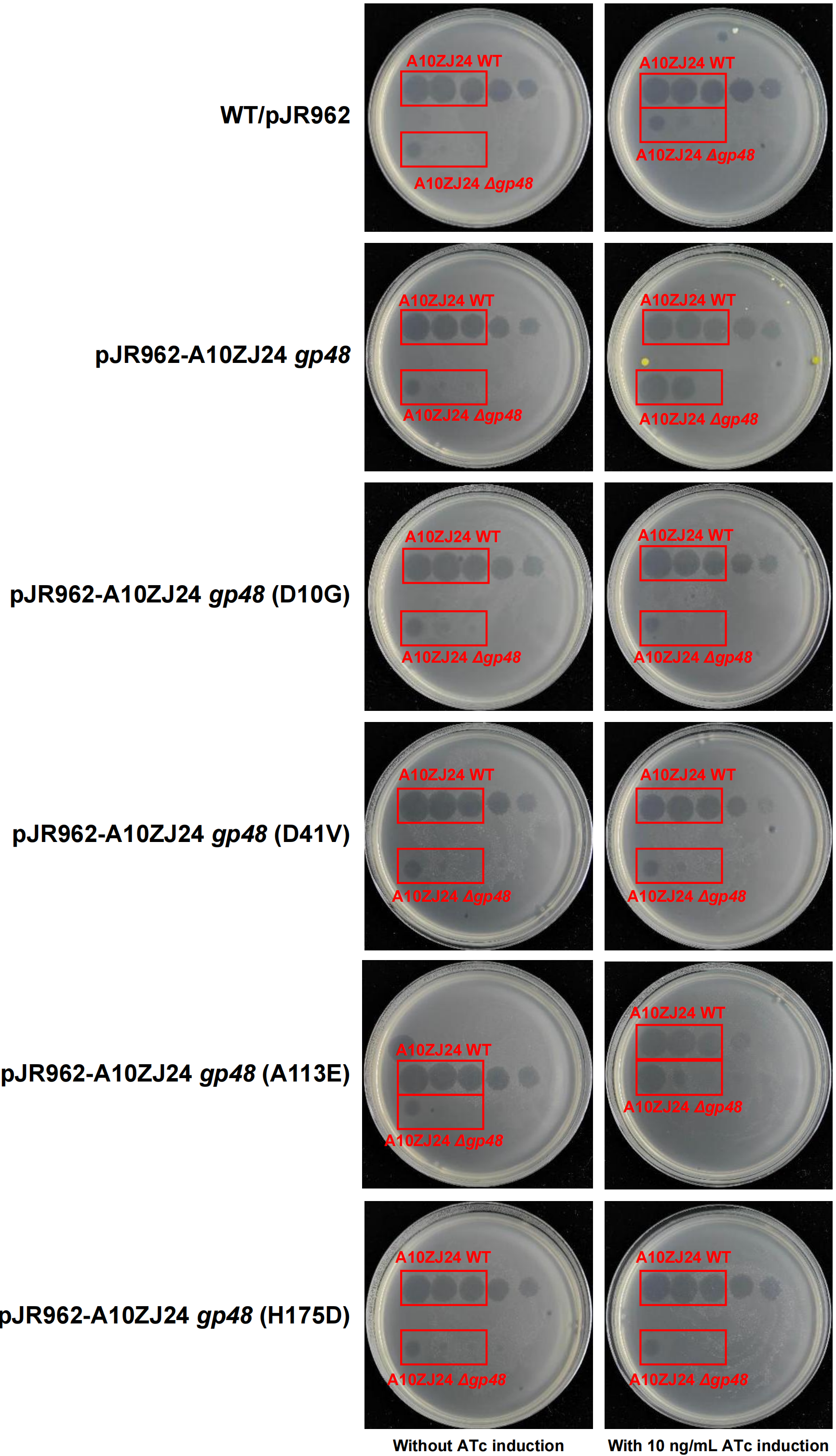

Supplement: Supplementary file 10 — Figure S2-5 Source Data [file 44319_2025_488_MOESM10_ESM.zip › Figure S2-S5_Source Data/Appendix Figure S4/S4A/README.tif]

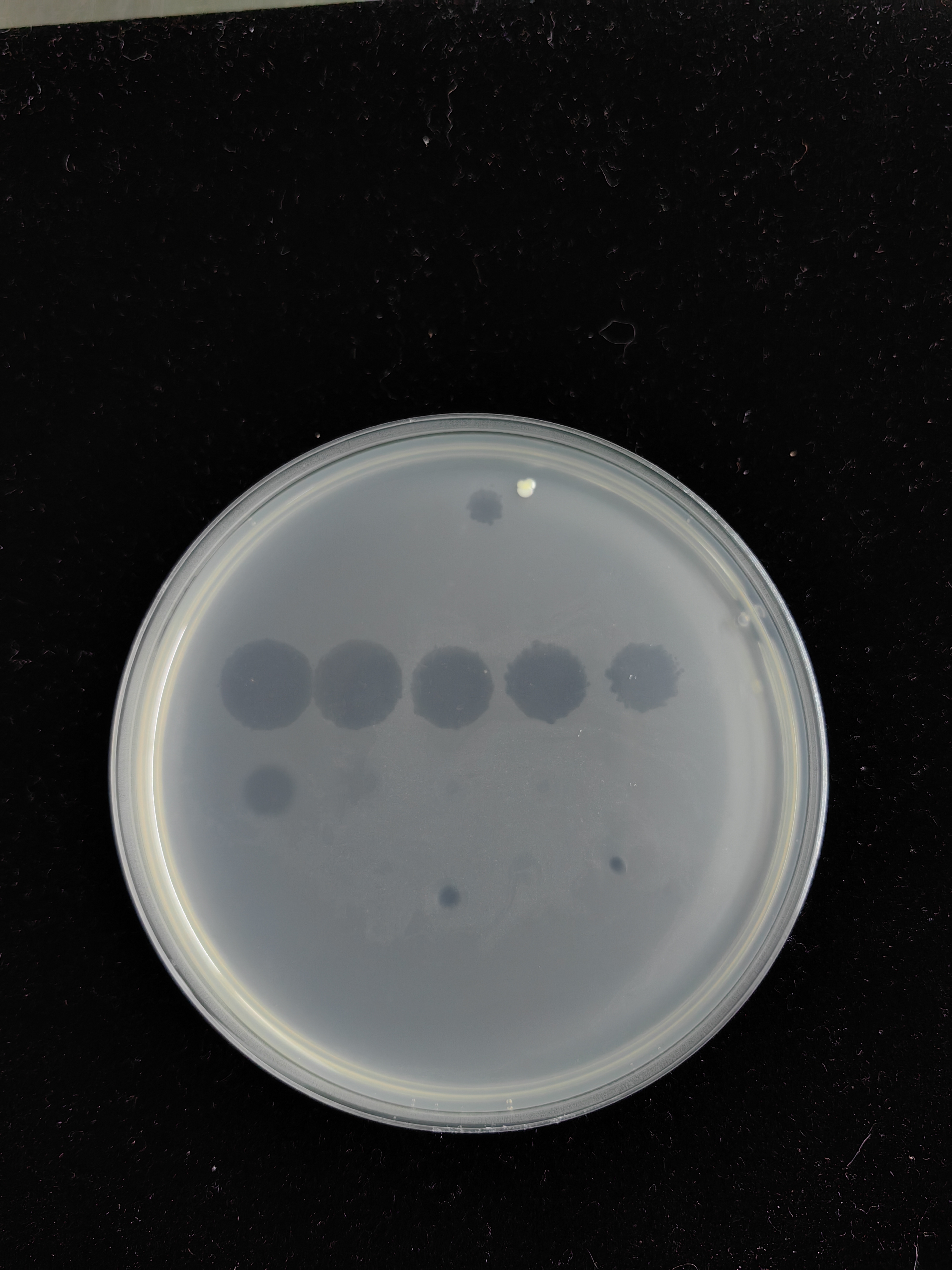

Supplement: Supplementary file 10 — Figure S2-5 Source Data [file 44319_2025_488_MOESM10_ESM.zip › Figure S2-S5_Source Data/Appendix Figure S4/S4A/WT-PJR962 with ATc induction.tiff]

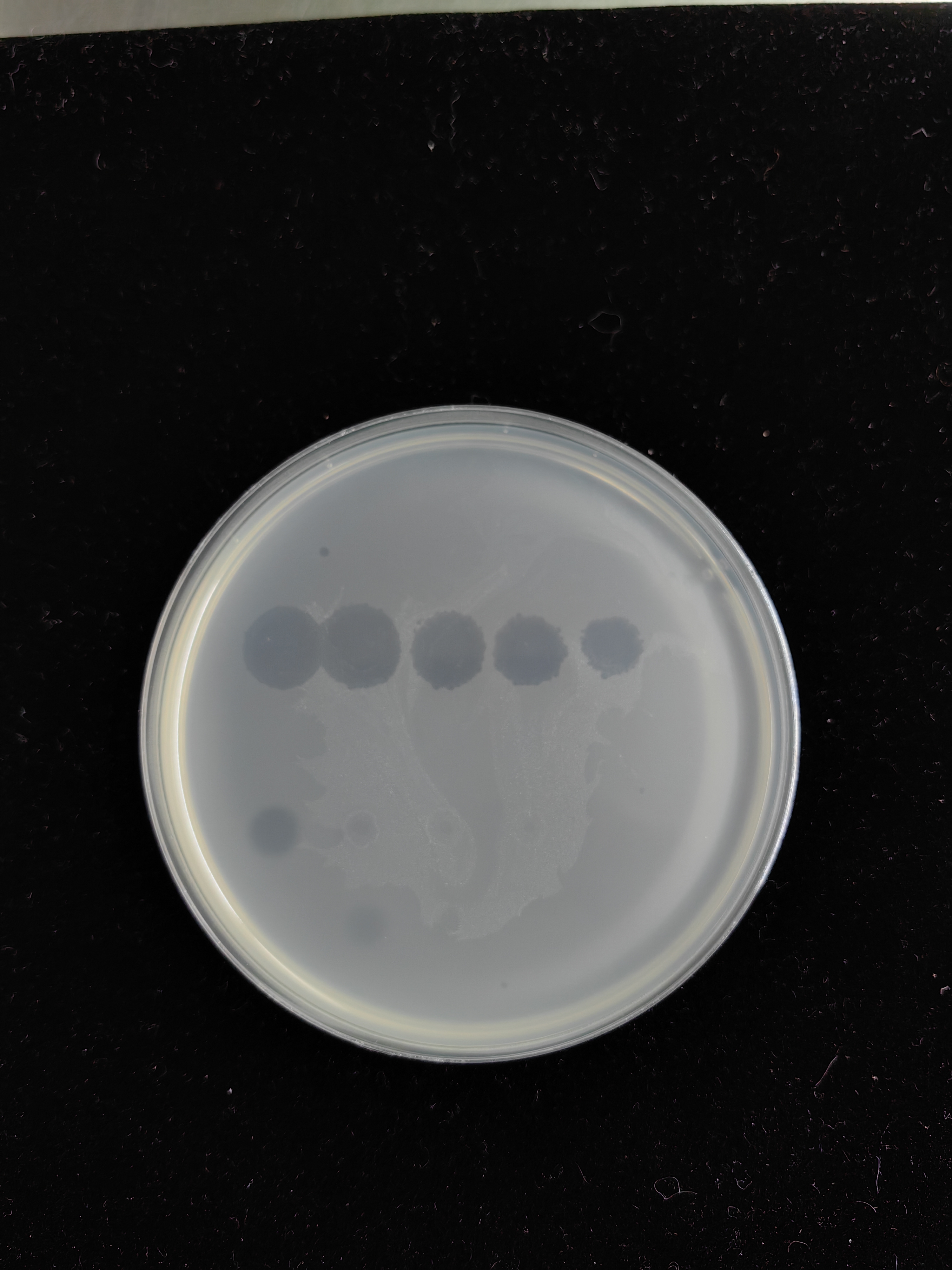

Supplement: Supplementary file 10 — Figure S2-5 Source Data [file 44319_2025_488_MOESM10_ESM.zip › Figure S2-S5_Source Data/Appendix Figure S4/S4A/WT-pJR962 without ATc induction.tiff]

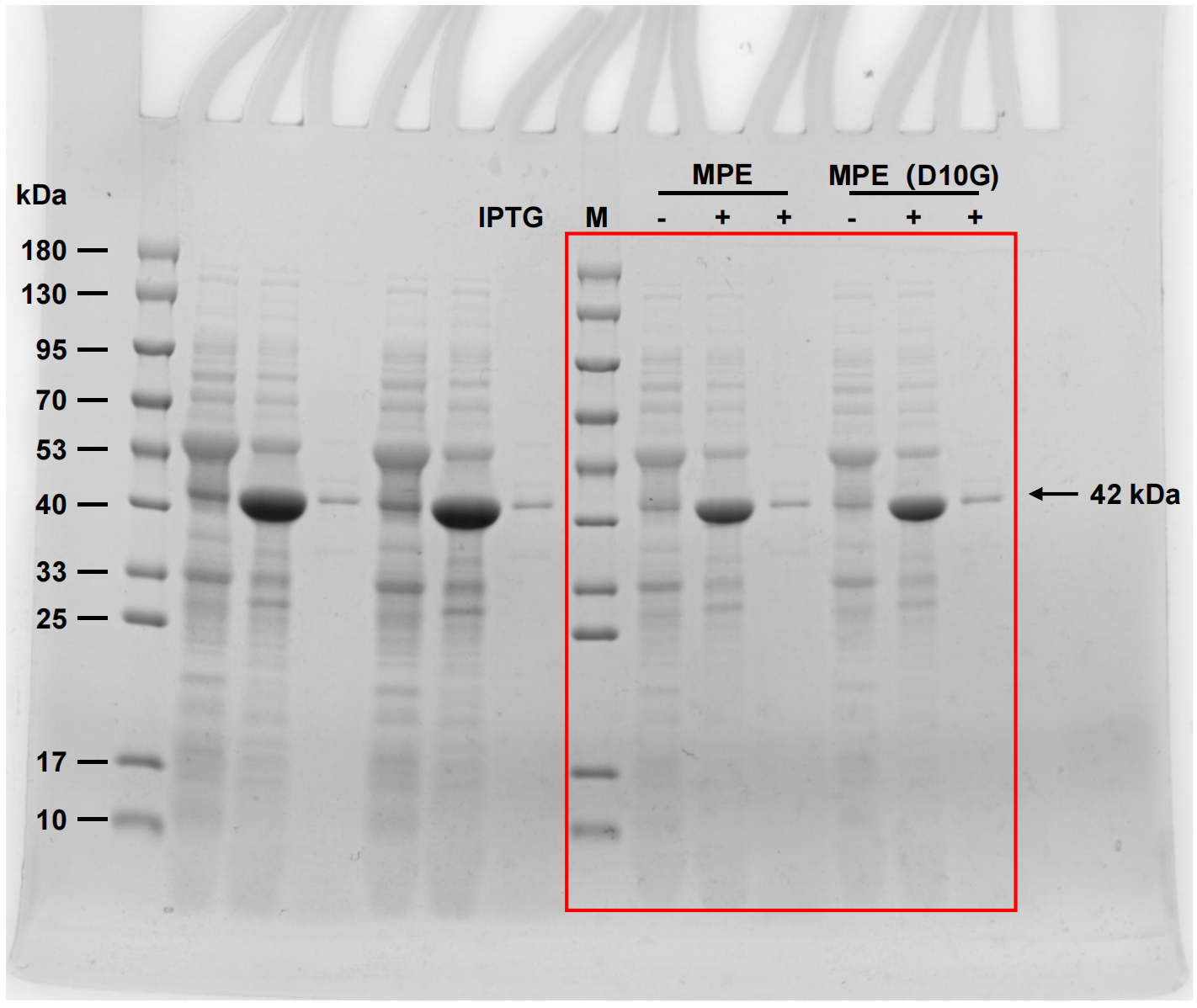

Supplement: Supplementary file 10 — Figure S2-5 Source Data [file 44319_2025_488_MOESM10_ESM.zip › Figure S2-S5_Source Data/Appendix Figure S4/S4B/Expression and purification of MPE.tif]

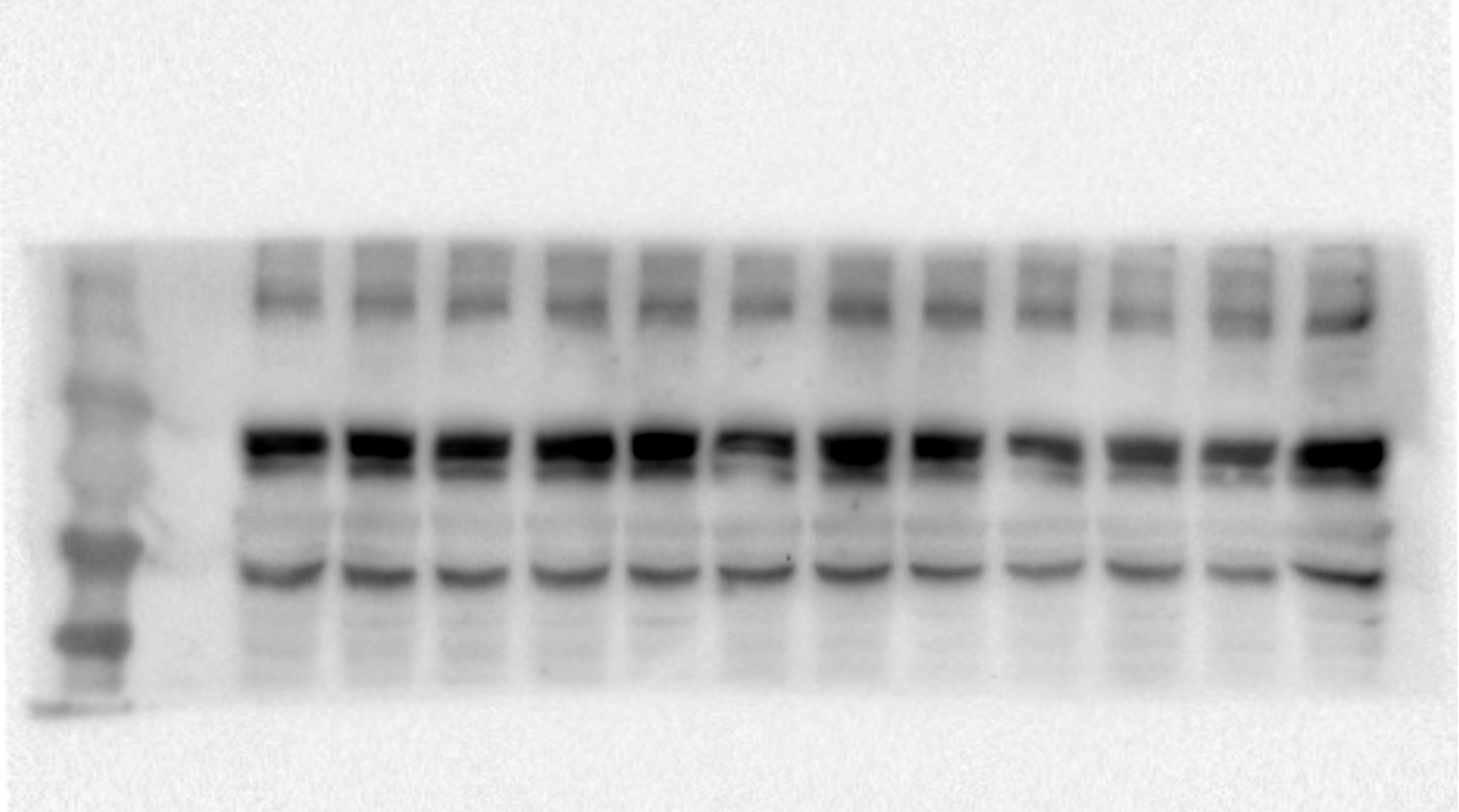

Supplement: Supplementary file 10 — Figure S2-5 Source Data [file 44319_2025_488_MOESM10_ESM.zip › Figure S2-S5_Source Data/Appendix Figure S5/S5B/In M.smegmatis KatG.tif]

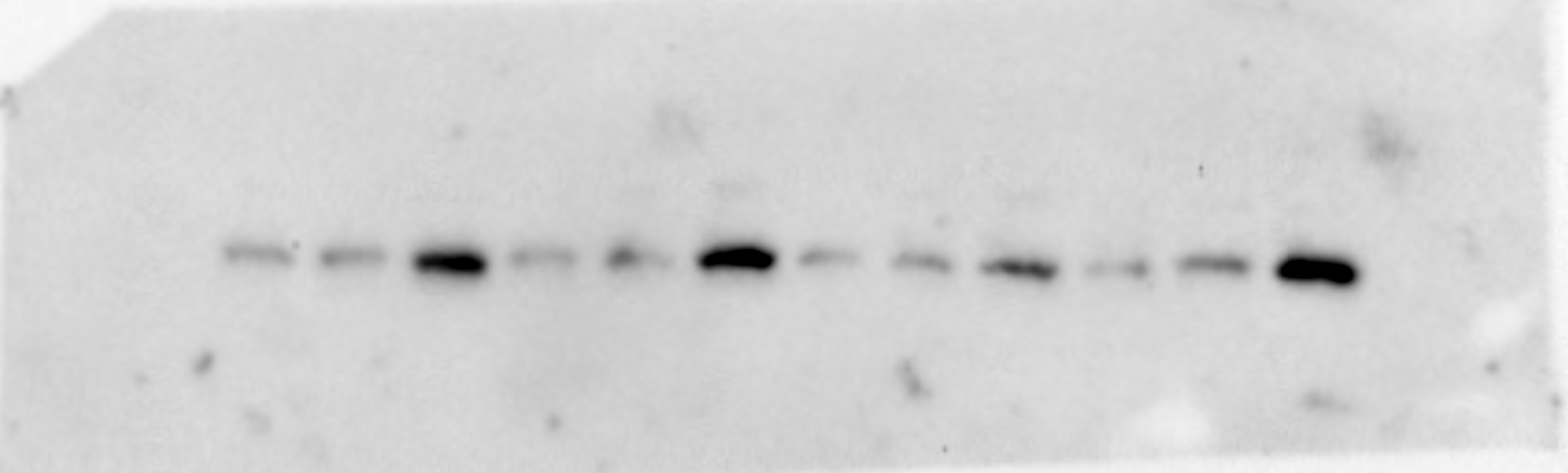

Supplement: Supplementary file 10 — Figure S2-5 Source Data [file 44319_2025_488_MOESM10_ESM.zip › Figure S2-S5_Source Data/Appendix Figure S5/S5B/In M.smegmatis MPE-Flag.tif]

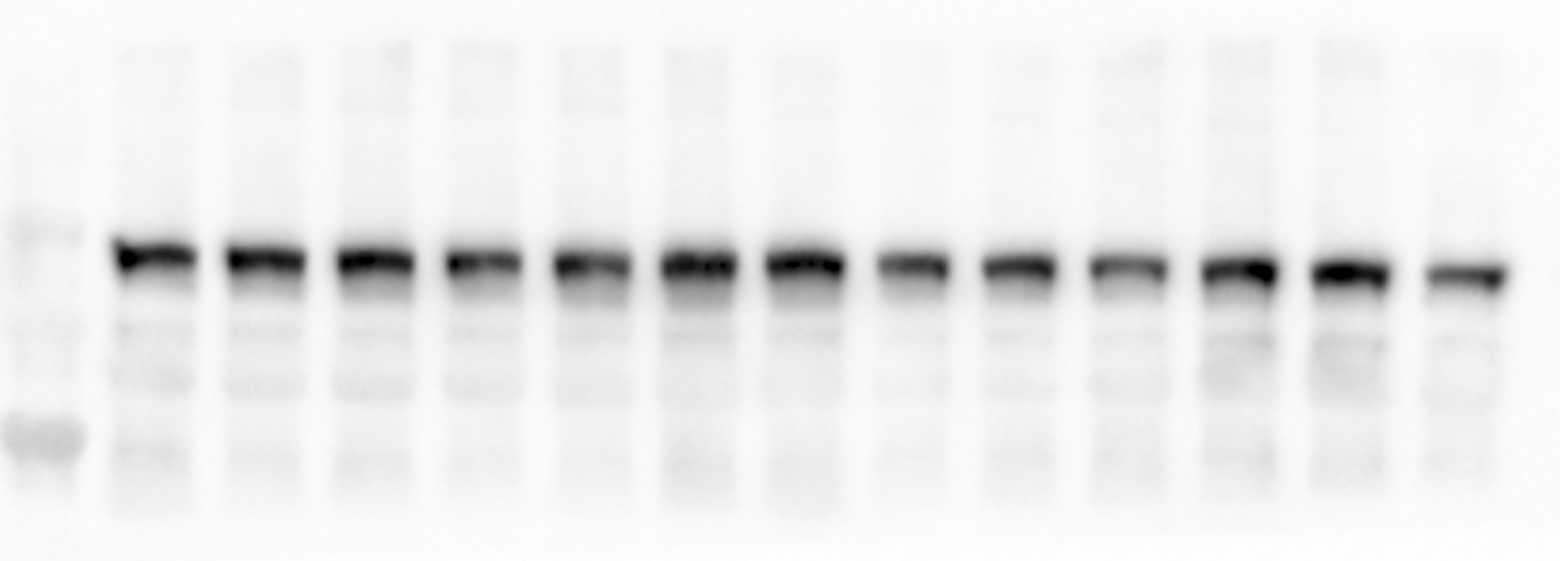

Supplement: Supplementary file 10 — Figure S2-5 Source Data [file 44319_2025_488_MOESM10_ESM.zip › Figure S2-S5_Source Data/Appendix Figure S5/S5B/In M.tuberculosis KatG-1.tif]

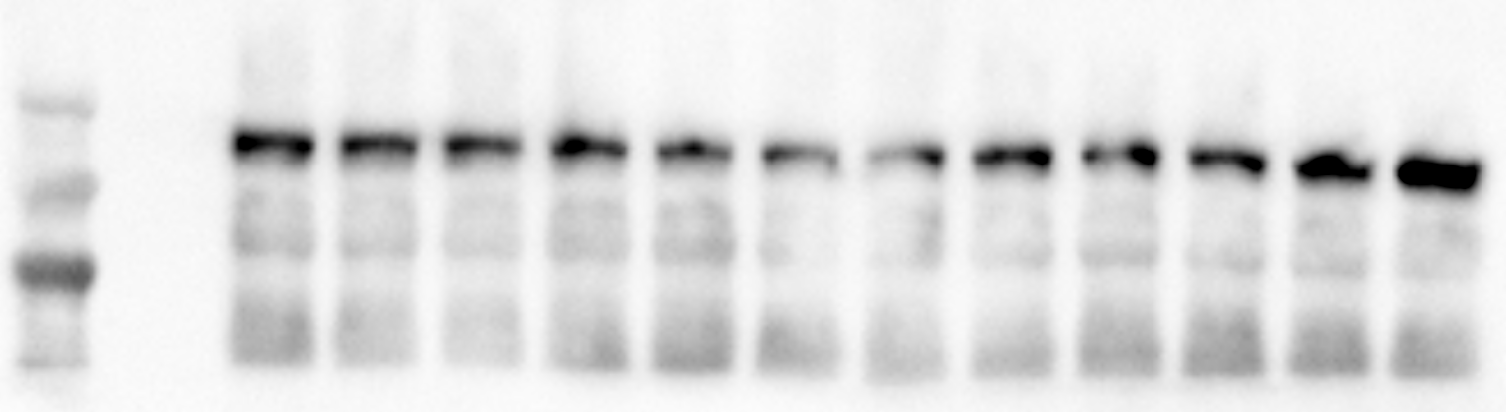

Supplement: Supplementary file 10 — Figure S2-5 Source Data [file 44319_2025_488_MOESM10_ESM.zip › Figure S2-S5_Source Data/Appendix Figure S5/S5B/In M.tuberculosis KatG-2.tif]

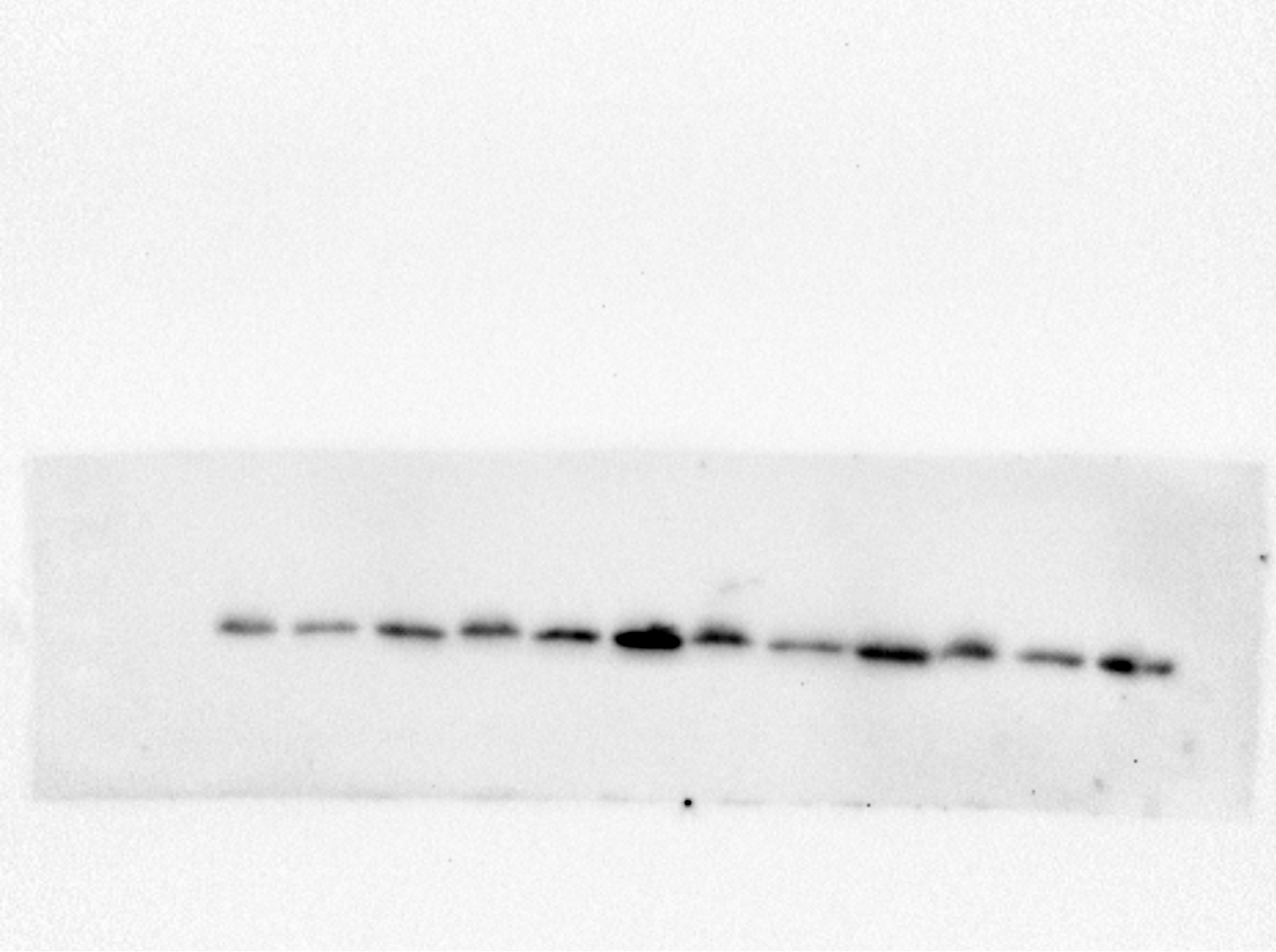

Supplement: Supplementary file 10 — Figure S2-5 Source Data [file 44319_2025_488_MOESM10_ESM.zip › Figure S2-S5_Source Data/Appendix Figure S5/S5B/In M.tuberculosis MPE-Flag-1.tif]

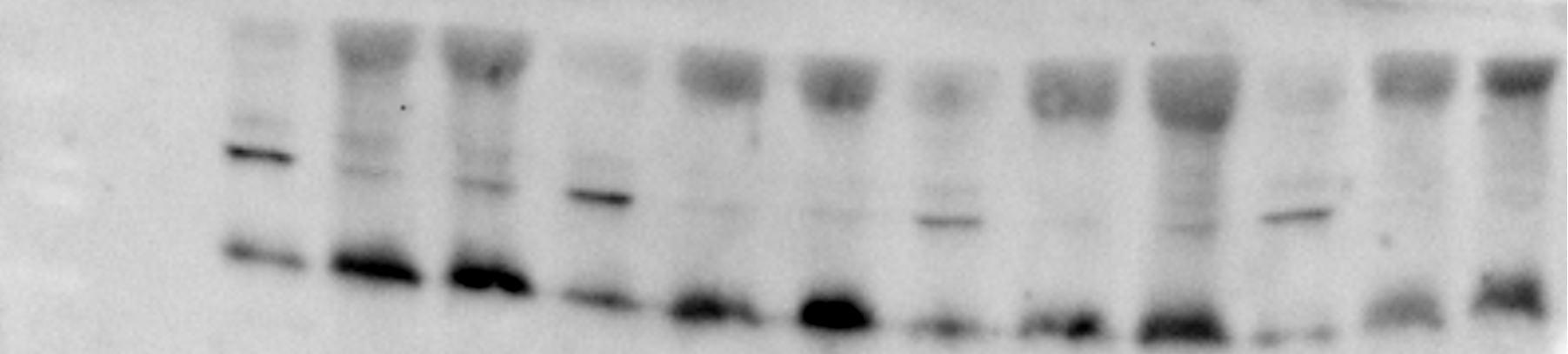

Supplement: Supplementary file 10 — Figure S2-5 Source Data [file 44319_2025_488_MOESM10_ESM.zip › Figure S2-S5_Source Data/Appendix Figure S5/S5B/In M.tuberculosis MPE-Flag-2.tif]

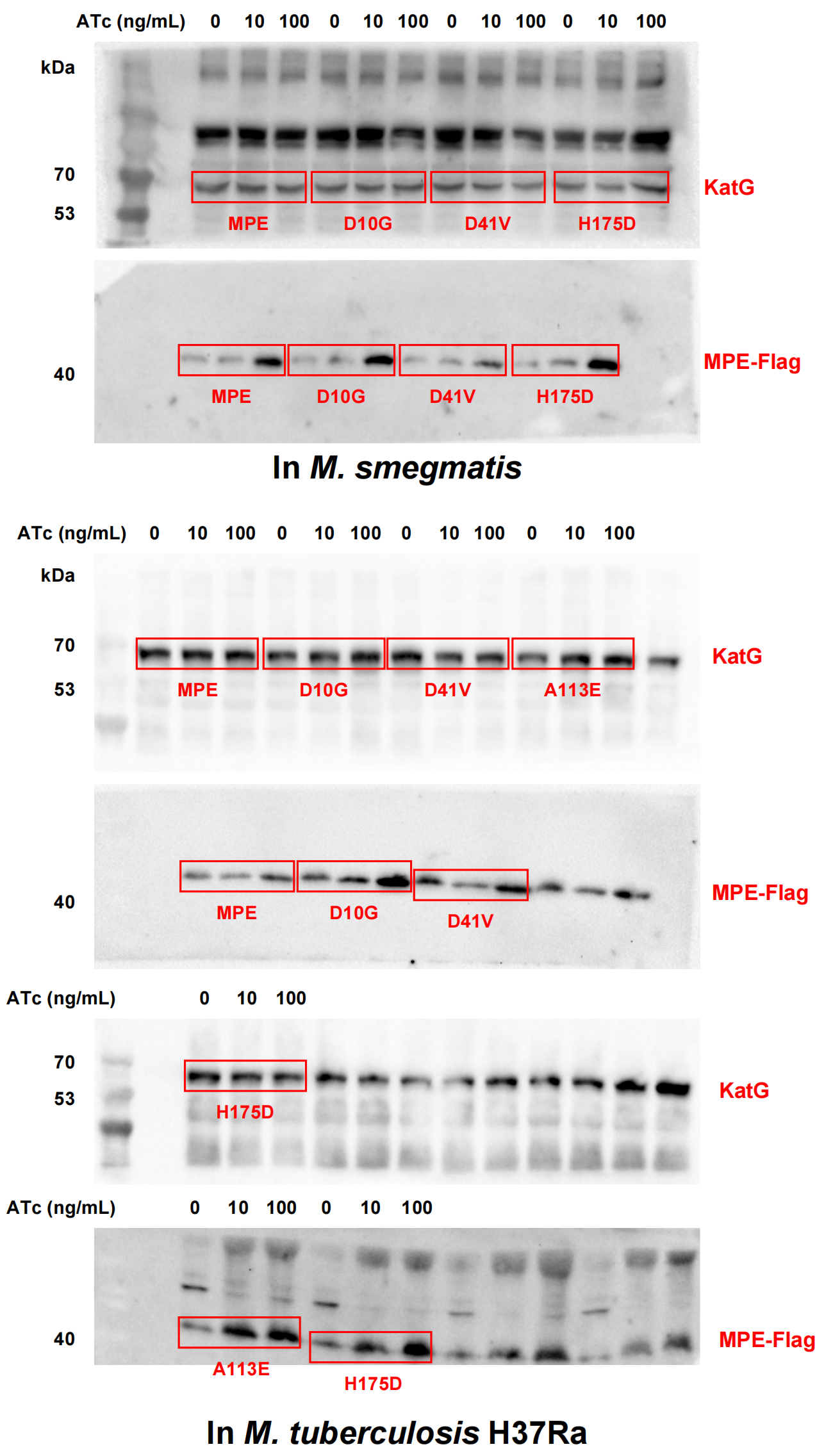

Supplement: Supplementary file 10 — Figure S2-5 Source Data [file 44319_2025_488_MOESM10_ESM.zip › Figure S2-S5_Source Data/Appendix Figure S5/S5B/README.tif]

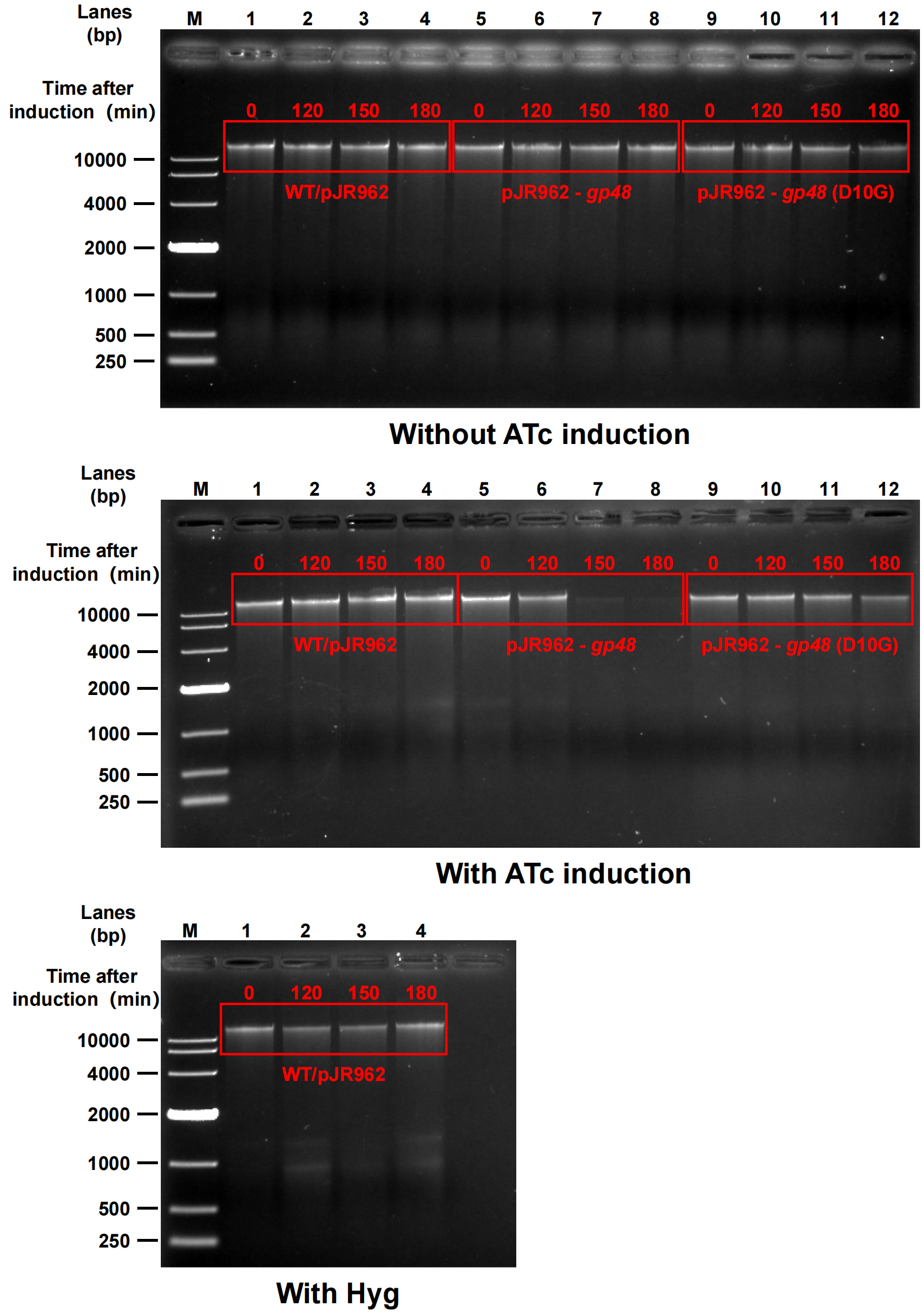

Supplement: Supplementary file 10 — Figure S2-5 Source Data [file 44319_2025_488_MOESM10_ESM.zip › Figure S2-S5_Source Data/Appendix Figure S5/S5D/README.tif]

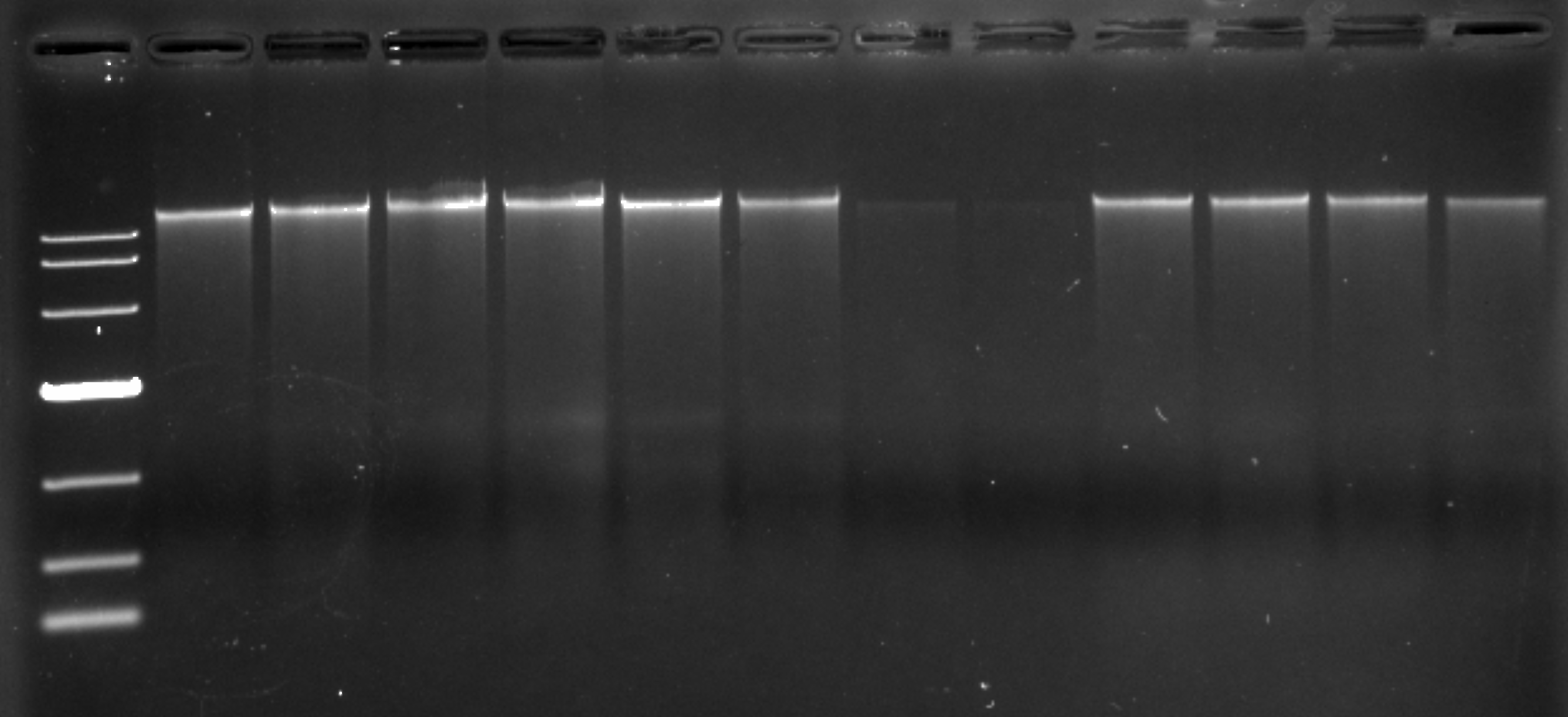

Supplement: Supplementary file 10 — Figure S2-5 Source Data [file 44319_2025_488_MOESM10_ESM.zip › Figure S2-S5_Source Data/Appendix Figure S5/S5D/With ATc induction.tif]

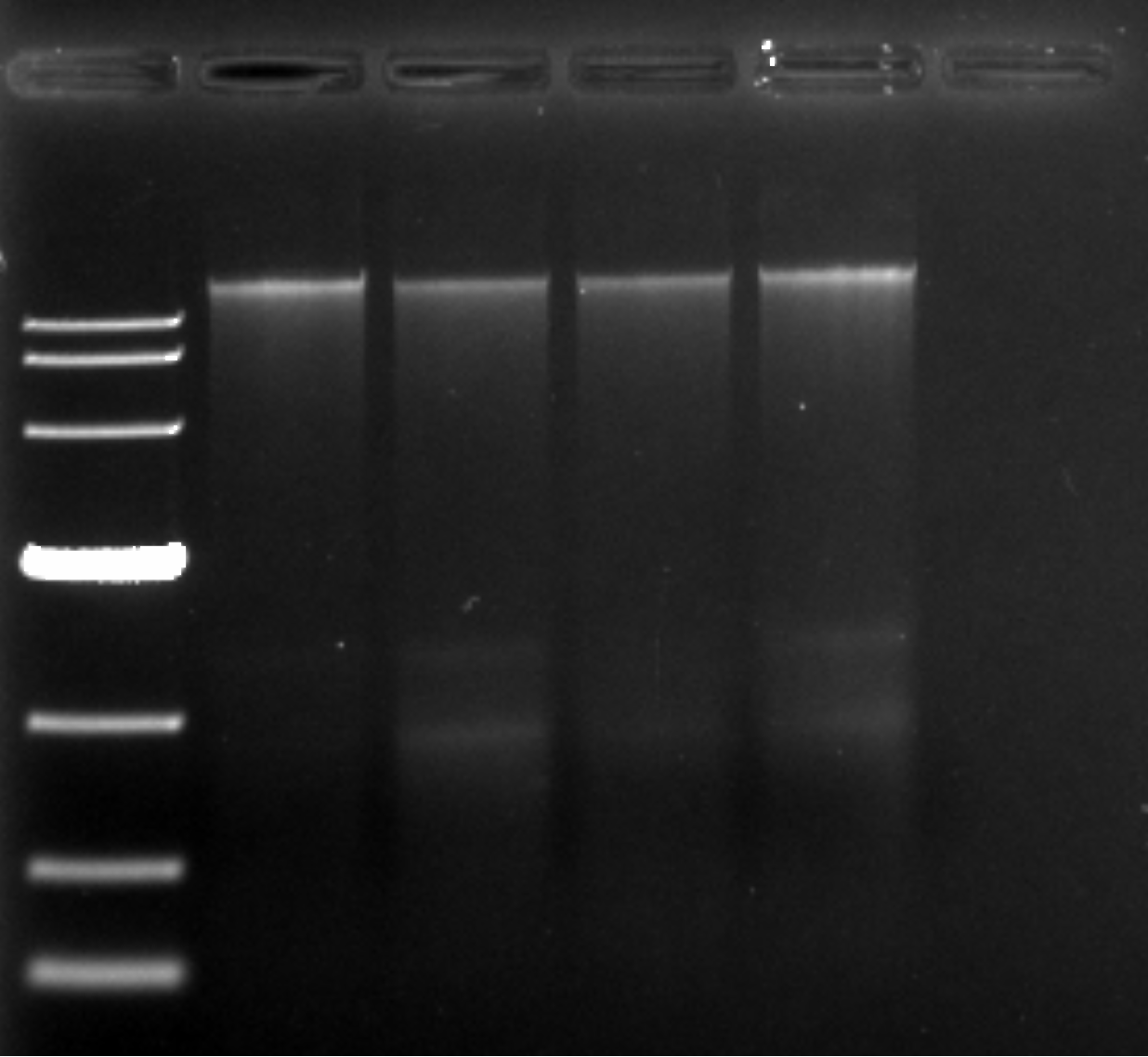

Supplement: Supplementary file 10 — Figure S2-5 Source Data [file 44319_2025_488_MOESM10_ESM.zip › Figure S2-S5_Source Data/Appendix Figure S5/S5D/With Hyg.tif]

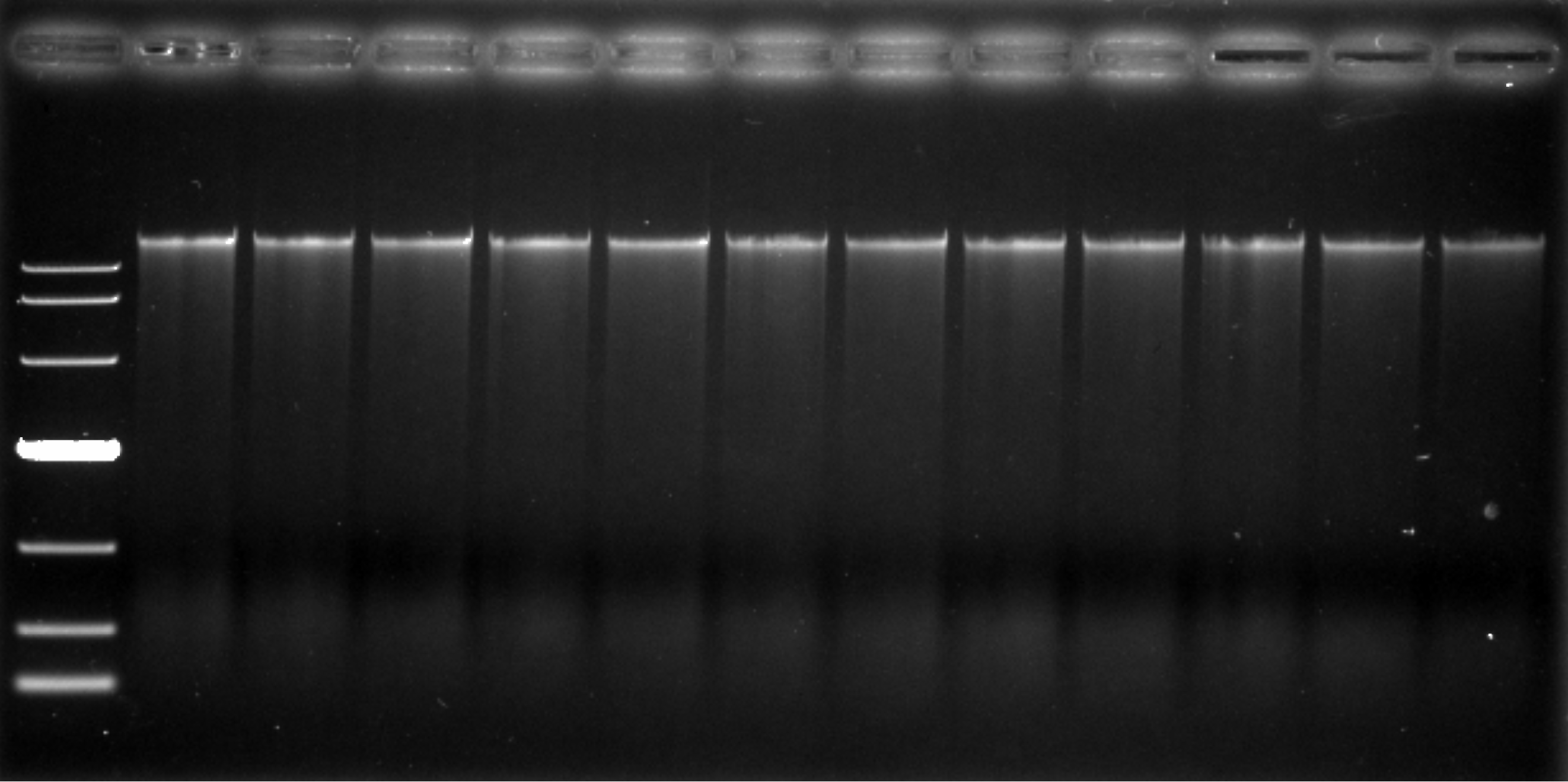

Supplement: Supplementary file 10 — Figure S2-5 Source Data [file 44319_2025_488_MOESM10_ESM.zip › Figure S2-S5_Source Data/Appendix Figure S5/S5D/Without ATc induction.tif]

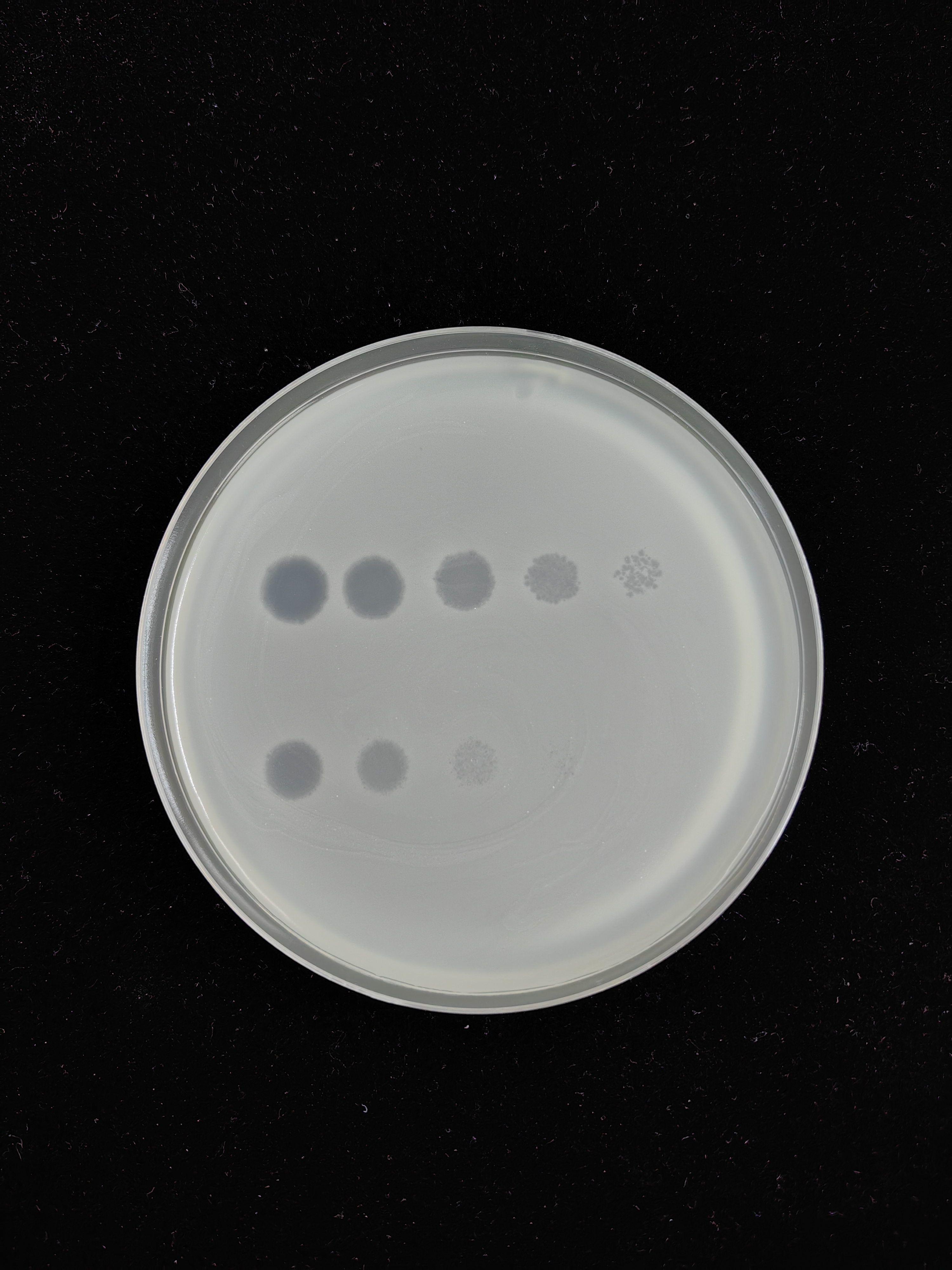

Supplement: Supplementary file 11 — Figure S6 Source Data [file 44319_2025_488_MOESM11_ESM.zip › Appendix Figure S6/S6A/pJR962-Mra Csm with ATc induction.tiff]

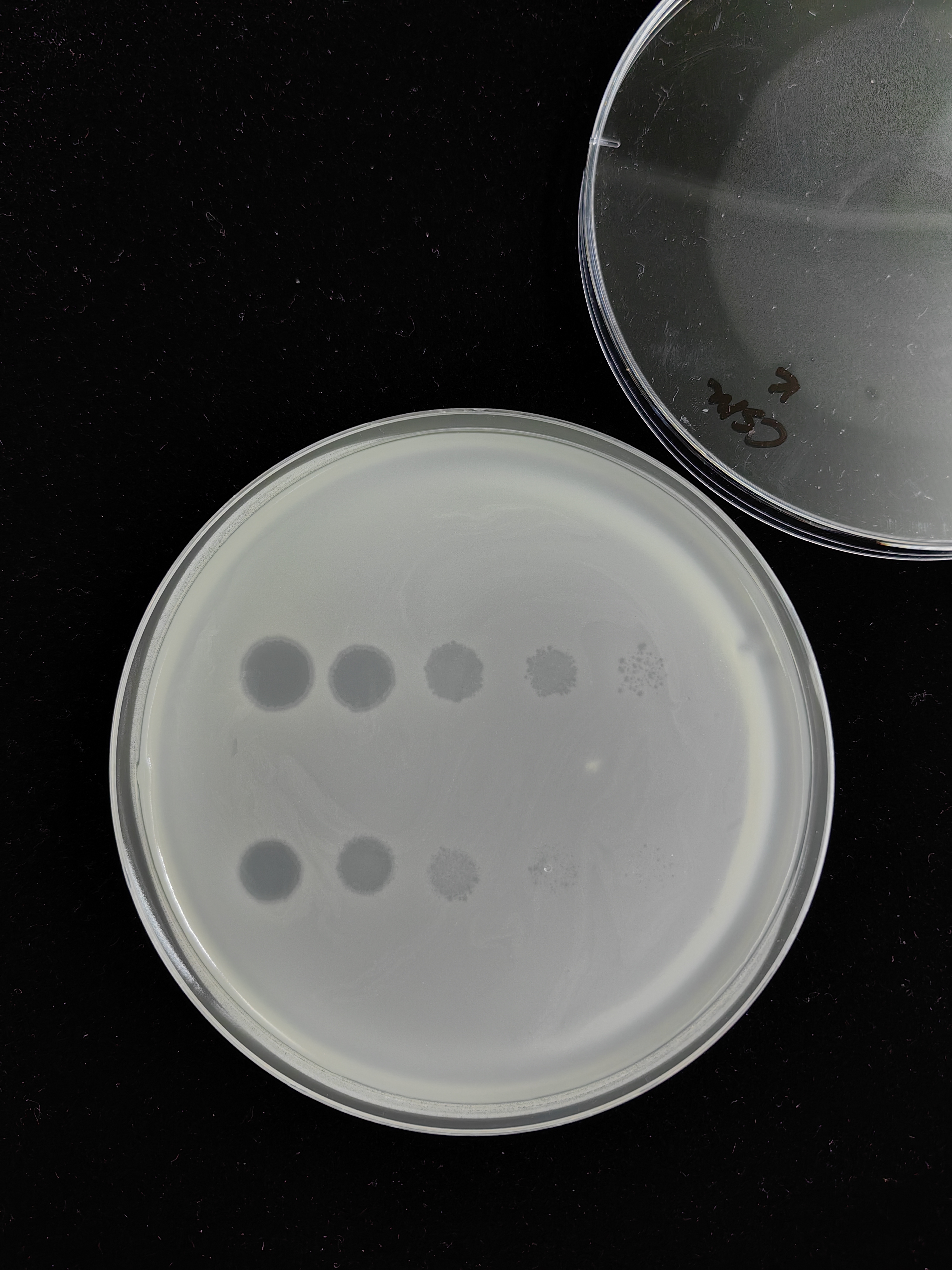

Supplement: Supplementary file 11 — Figure S6 Source Data [file 44319_2025_488_MOESM11_ESM.zip › Appendix Figure S6/S6A/pJR962-Mra Csm without ATc induction.tiff]

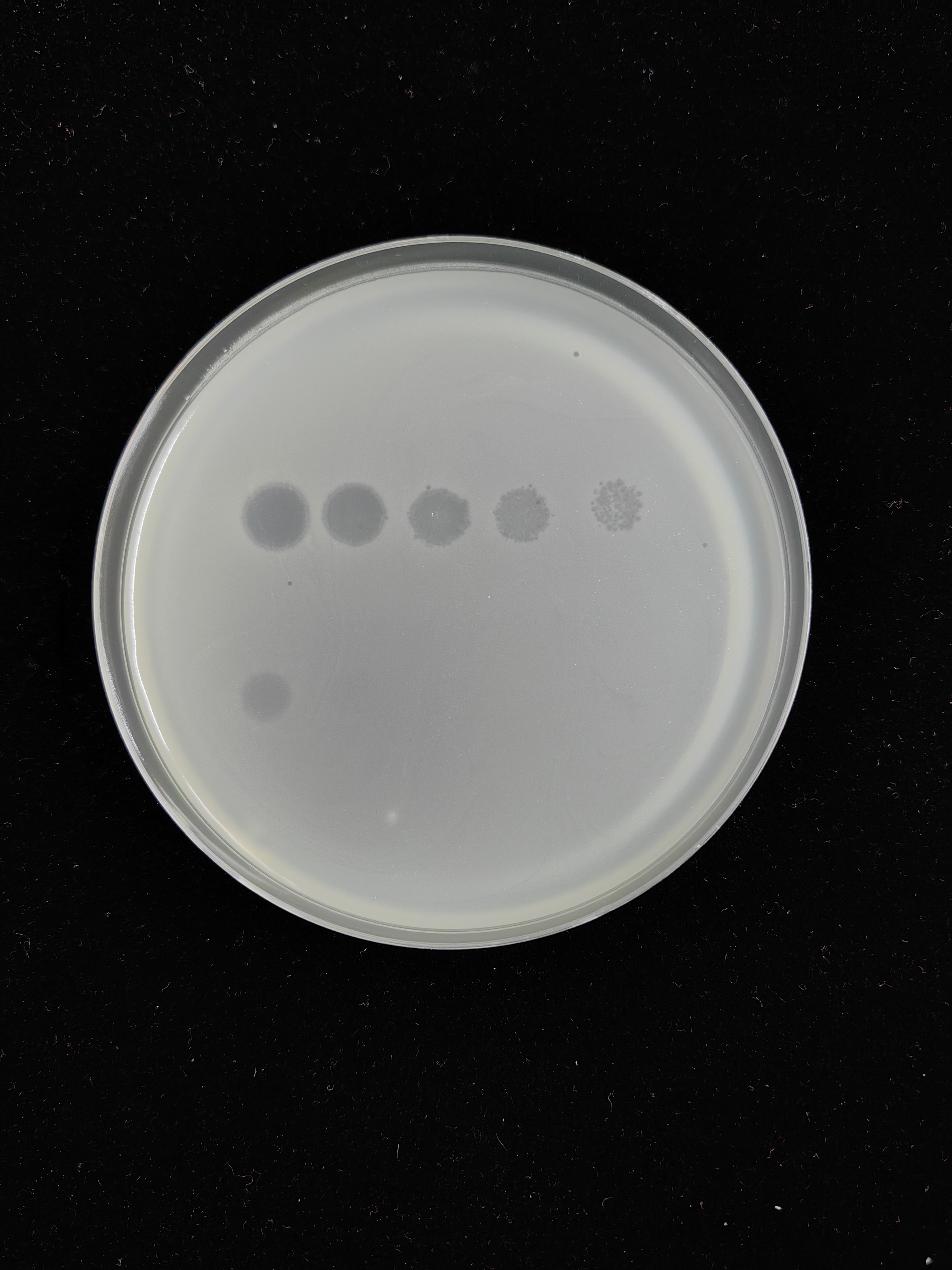

Supplement: Supplementary file 11 — Figure S6 Source Data [file 44319_2025_488_MOESM11_ESM.zip › Appendix Figure S6/S6A/pJR962-Mra IS6110 with ATc induction.tiff]

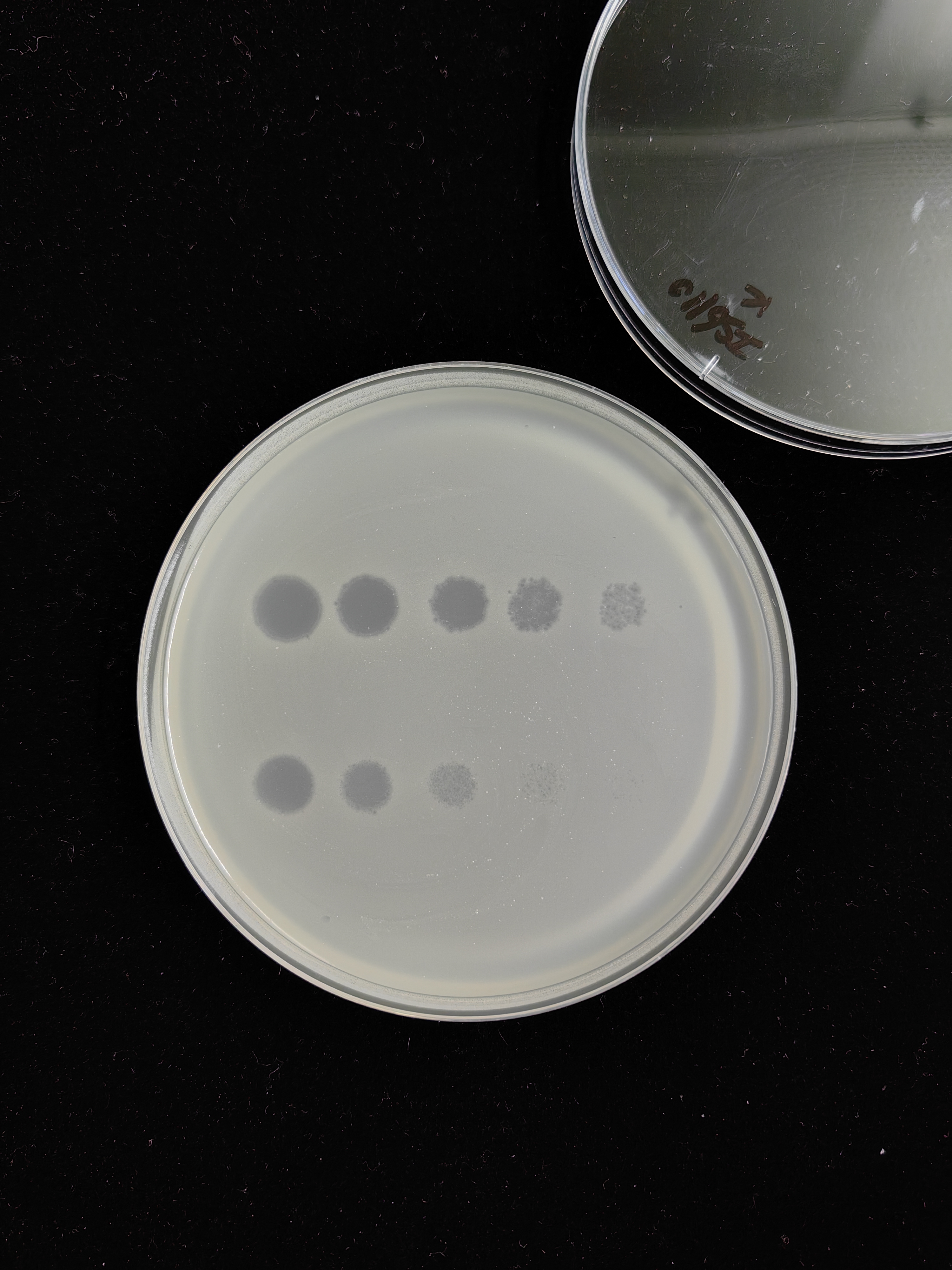

Supplement: Supplementary file 11 — Figure S6 Source Data [file 44319_2025_488_MOESM11_ESM.zip › Appendix Figure S6/S6A/pJR962-Mra IS6110 without ATc induction.tiff]

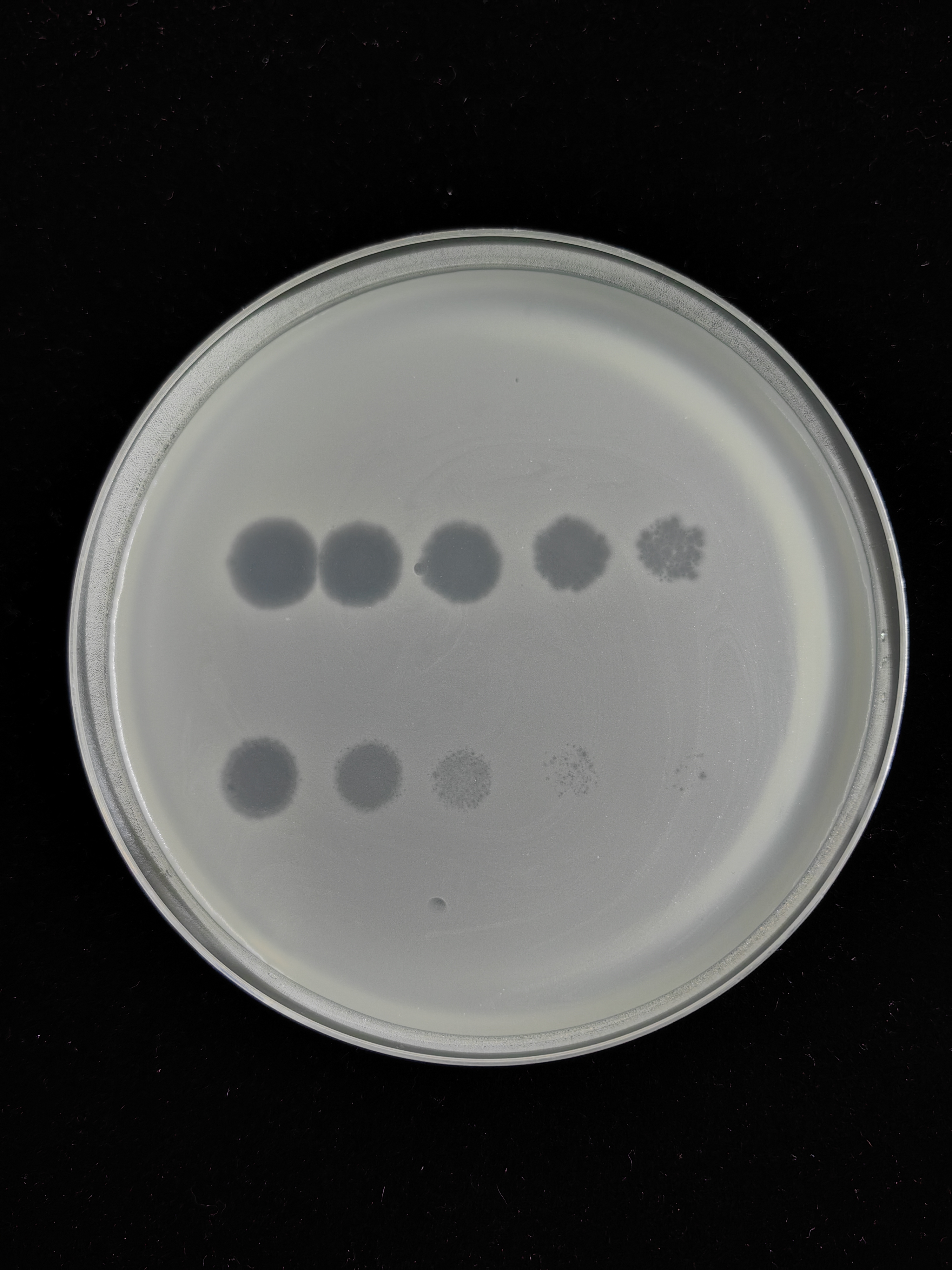

Supplement: Supplementary file 11 — Figure S6 Source Data [file 44319_2025_488_MOESM11_ESM.zip › Appendix Figure S6/S6A/pJR962-Mra_0950 with ATc induction.tiff]
