# Supplementary figures and images for: A conserved phage phosphoesterase enables evasion of bacterial antiviral immunity (part 2 of 2)
Source: EMBO Rep. 2025 May 29;26(14):3594–613. doi: 10.1038/s44319-025-00488-4 (PMC12287305; doi:10.1038/s44319-025-00488-4)

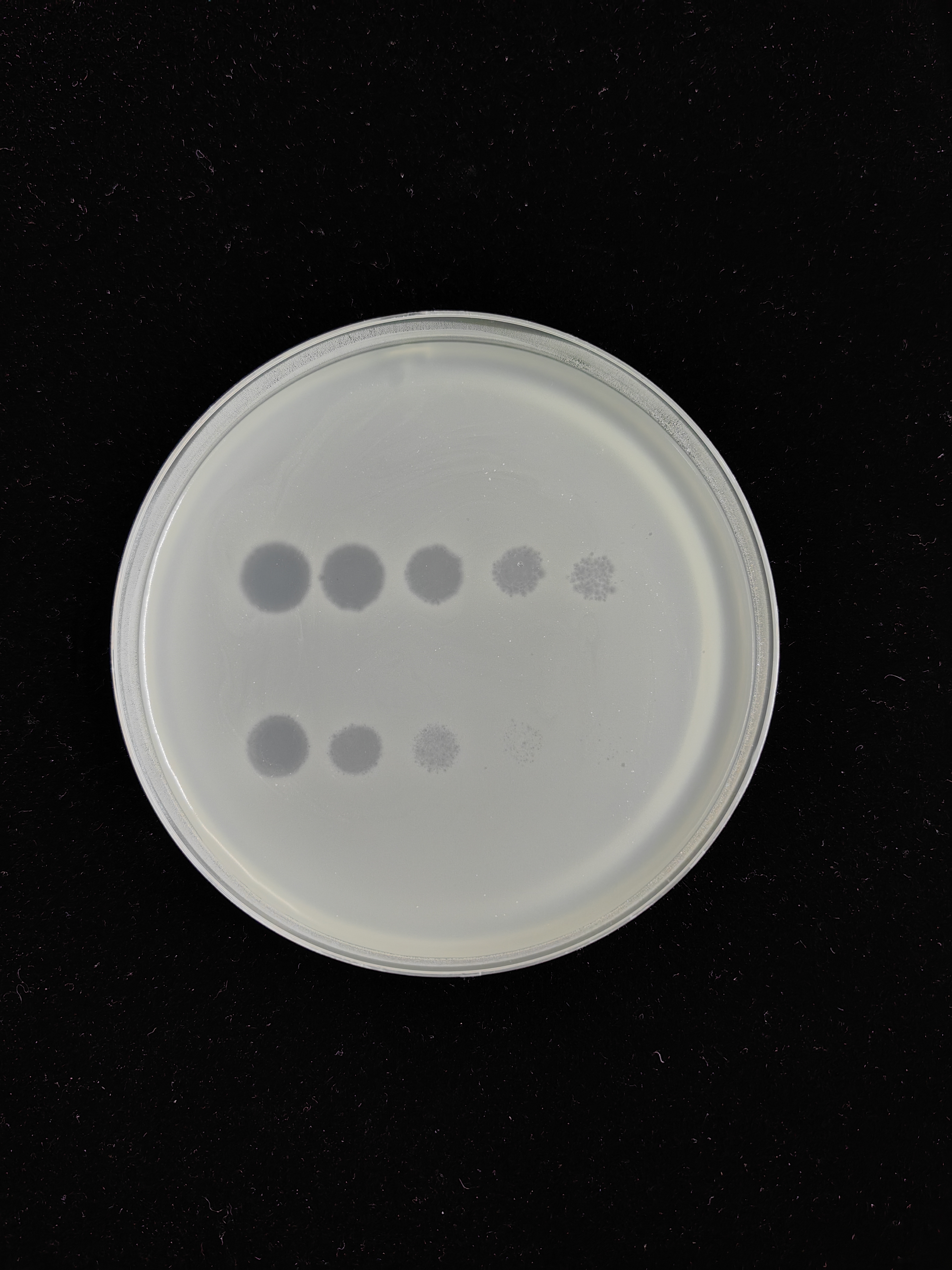

Supplement: Supplementary file 11 — Figure S6 Source Data [file 44319_2025_488_MOESM11_ESM.zip › Appendix Figure S6/S6A/pJR962-Mra_0950 without ATc induction.tiff]

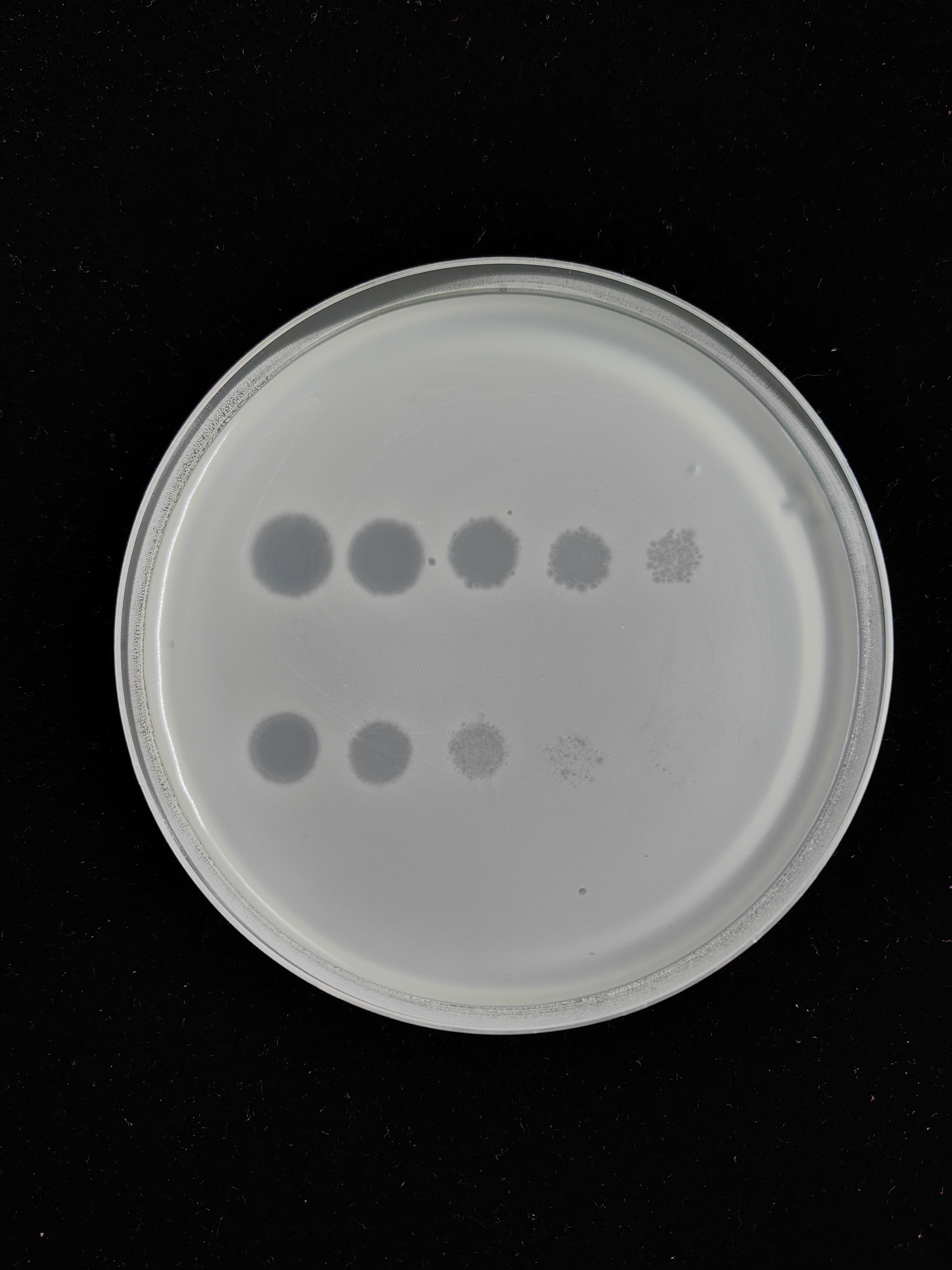

Supplement: Supplementary file 11 — Figure S6 Source Data [file 44319_2025_488_MOESM11_ESM.zip › Appendix Figure S6/S6A/pJR962-Mra_1125 with ATc induction.tiff]

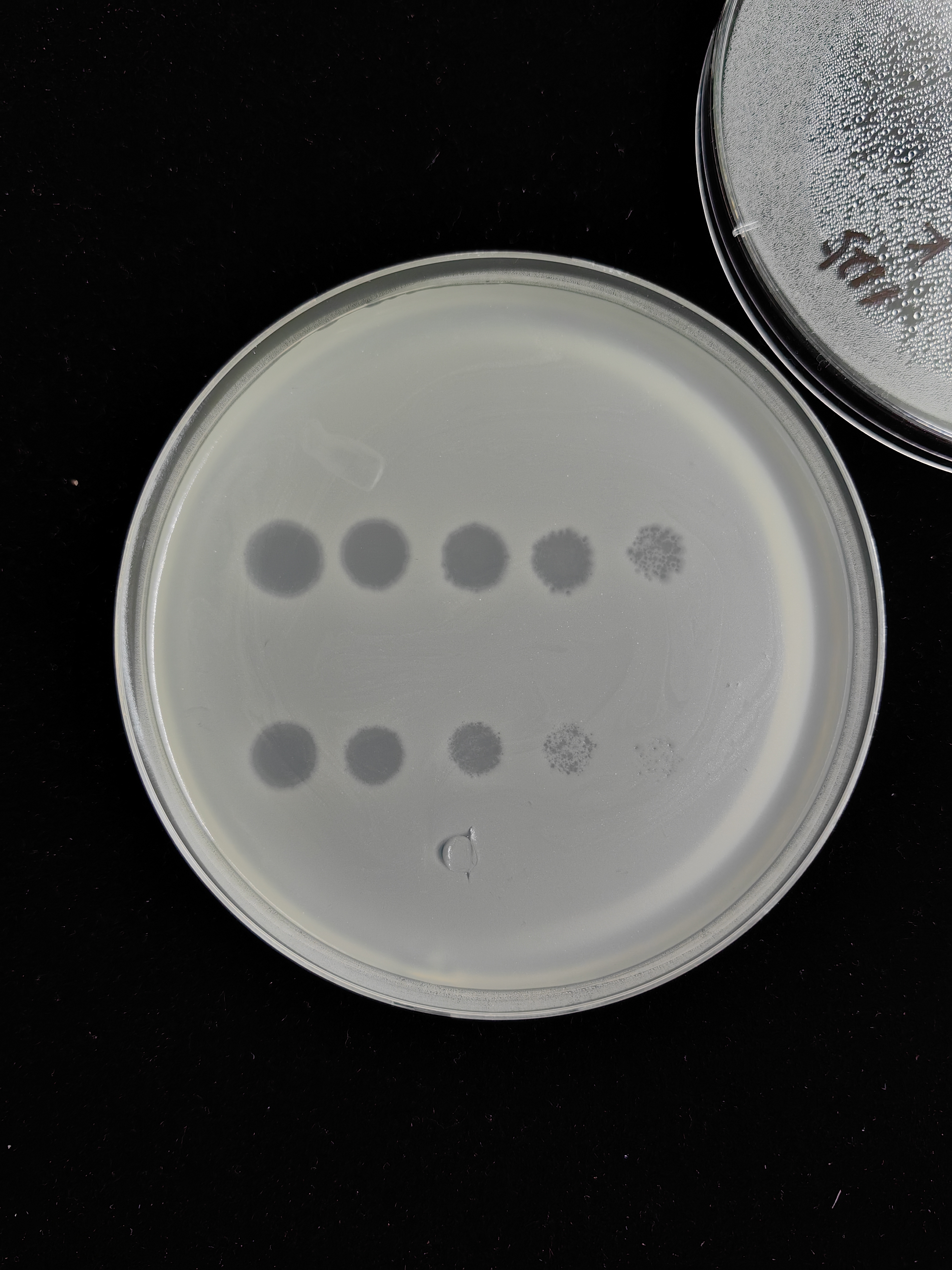

Supplement: Supplementary file 11 — Figure S6 Source Data [file 44319_2025_488_MOESM11_ESM.zip › Appendix Figure S6/S6A/pJR962-Mra_1125 without ATc induction.tiff]

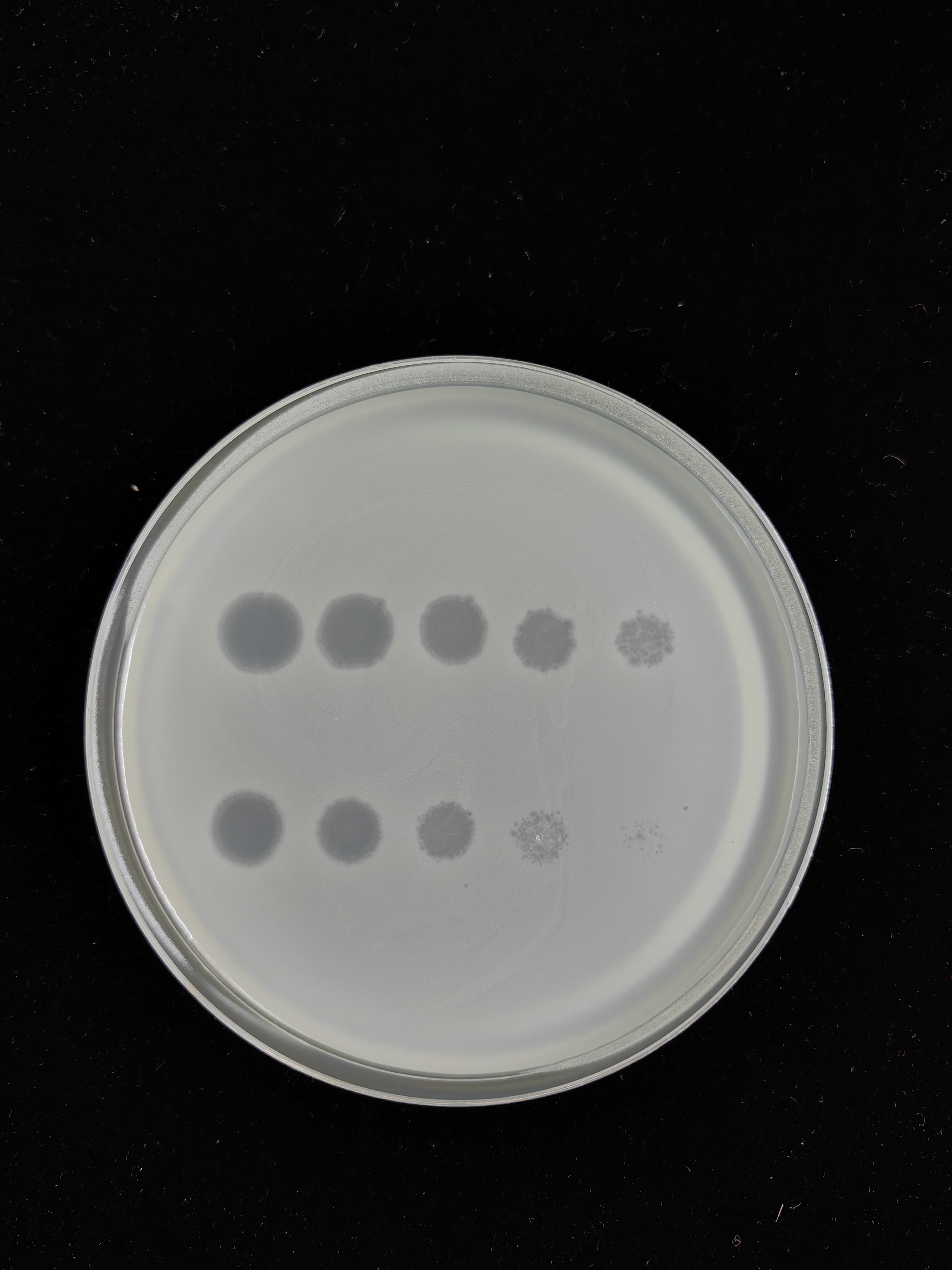

Supplement: Supplementary file 11 — Figure S6 Source Data [file 44319_2025_488_MOESM11_ESM.zip › Appendix Figure S6/S6A/pJR962-Mra_1160 with ATc induction.tiff]

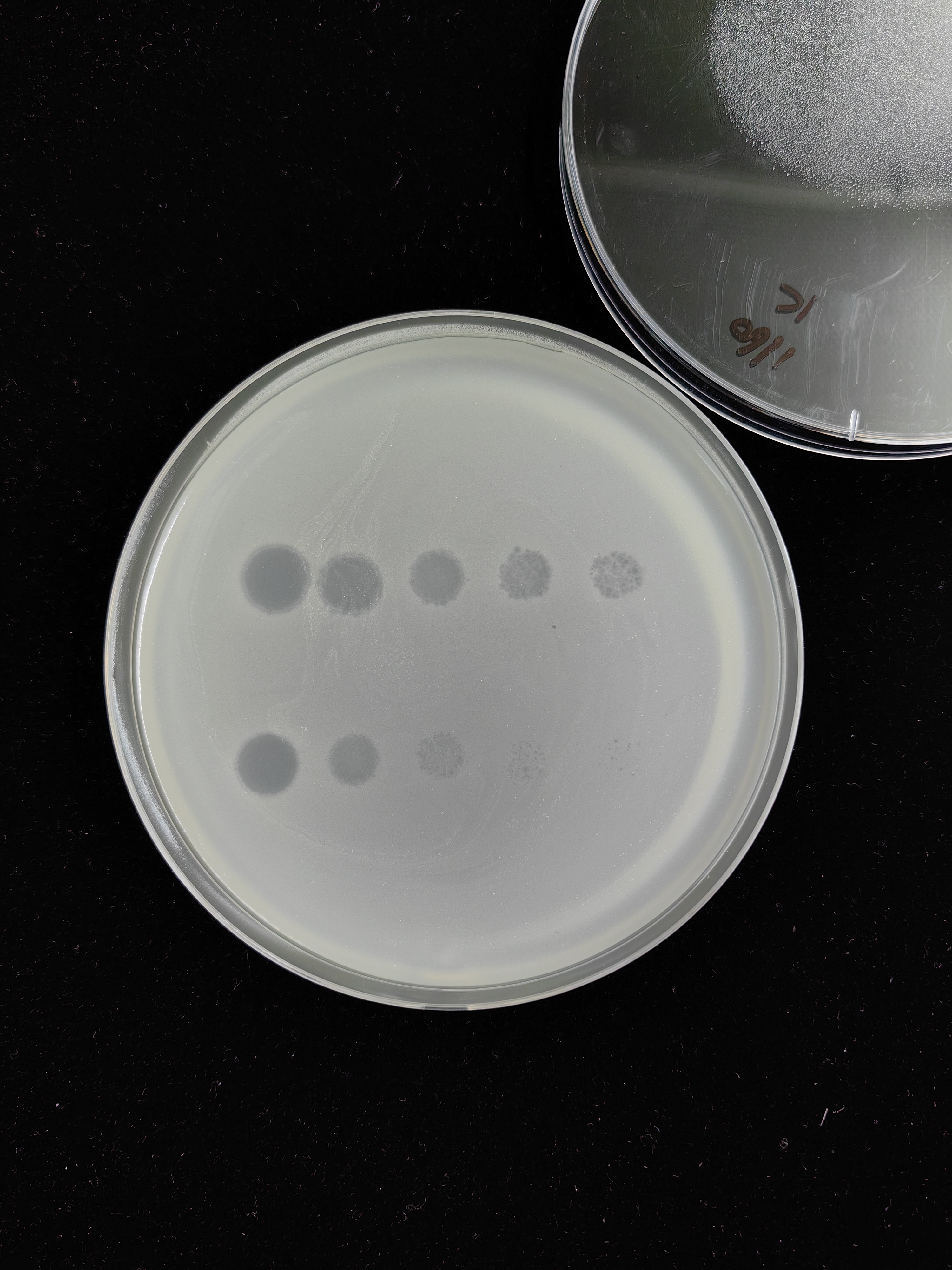

Supplement: Supplementary file 11 — Figure S6 Source Data [file 44319_2025_488_MOESM11_ESM.zip › Appendix Figure S6/S6A/pJR962-Mra_1160 without ATc induction.tiff]

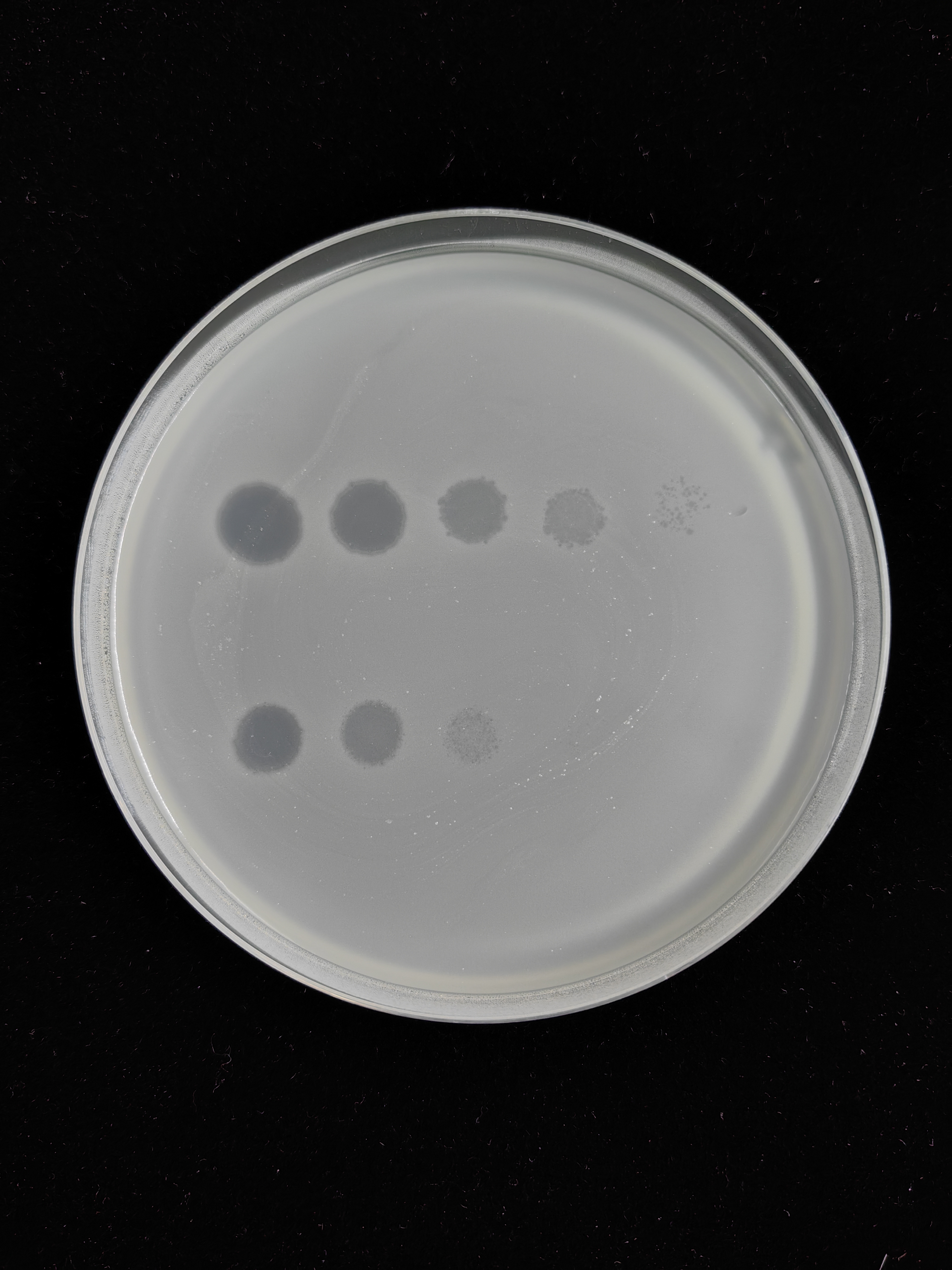

Supplement: Supplementary file 11 — Figure S6 Source Data [file 44319_2025_488_MOESM11_ESM.zip › Appendix Figure S6/S6A/pJR962-Mra_1598A with ATc induction.tiff]

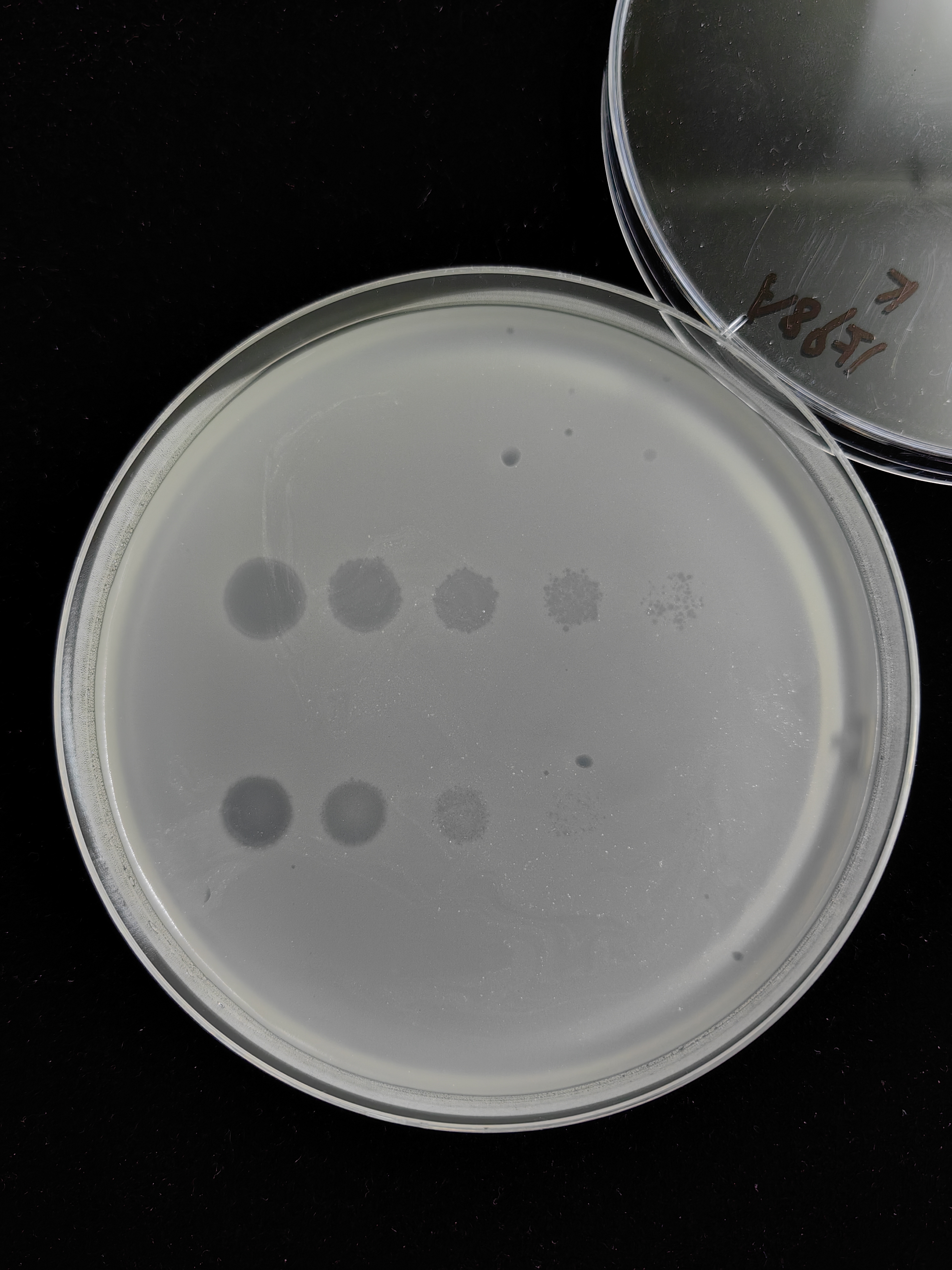

Supplement: Supplementary file 11 — Figure S6 Source Data [file 44319_2025_488_MOESM11_ESM.zip › Appendix Figure S6/S6A/pJR962-Mra_1598A without ATc induction.tiff]

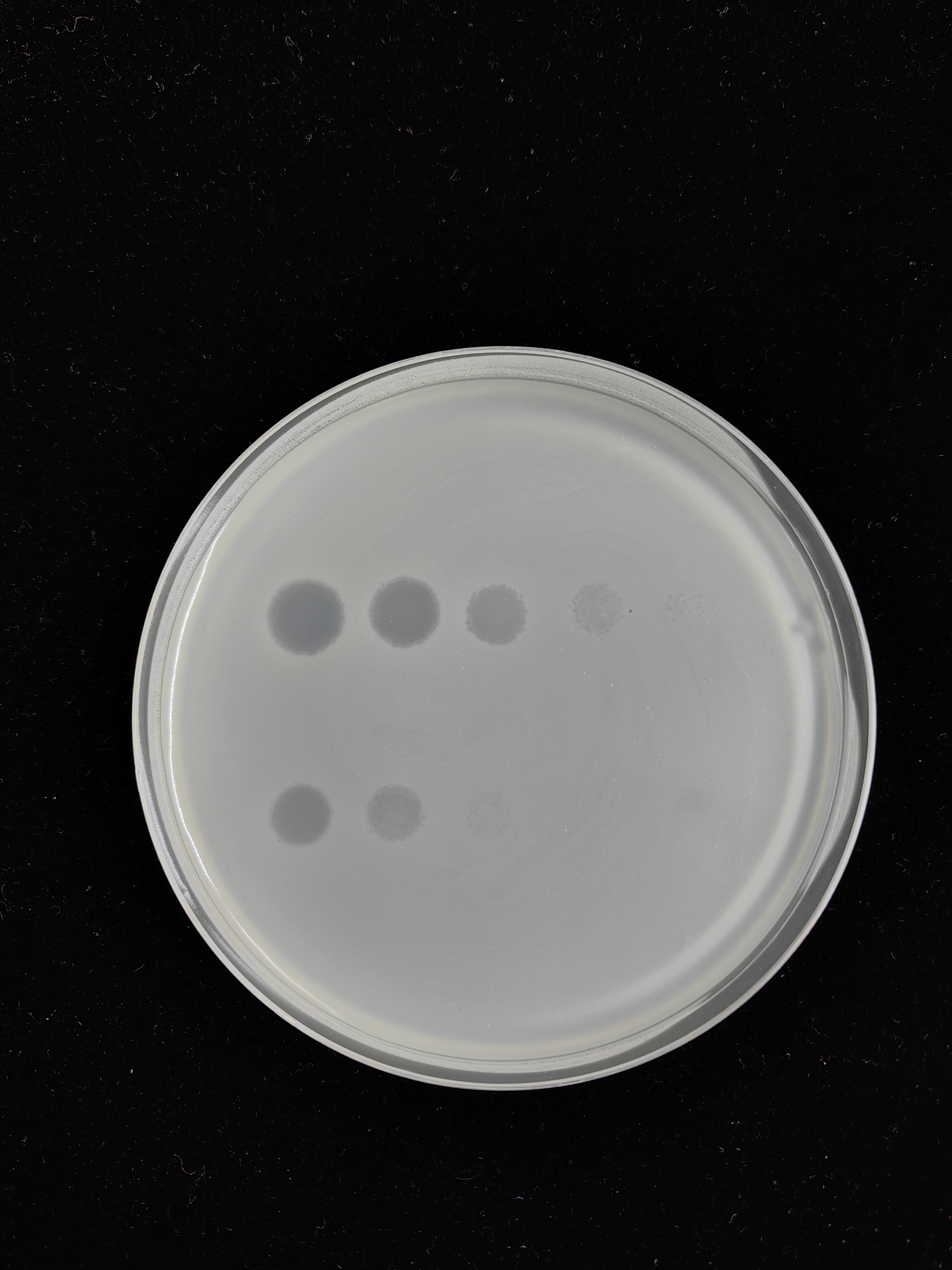

Supplement: Supplementary file 11 — Figure S6 Source Data [file 44319_2025_488_MOESM11_ESM.zip › Appendix Figure S6/S6A/pJR962-Mra_1649 with ATc induction.tiff]

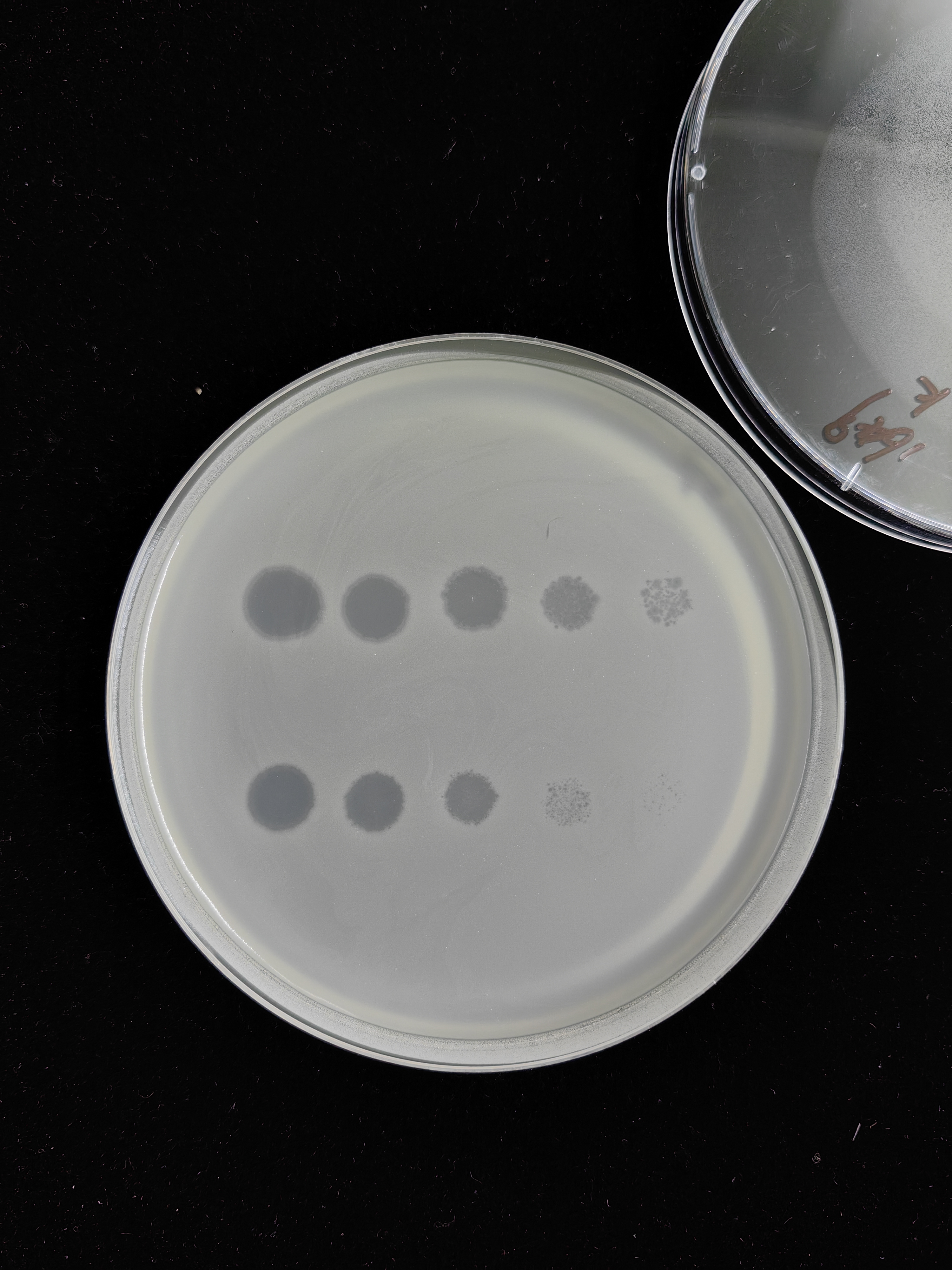

Supplement: Supplementary file 11 — Figure S6 Source Data [file 44319_2025_488_MOESM11_ESM.zip › Appendix Figure S6/S6A/pJR962-Mra_1649 without ATc induction.tiff]

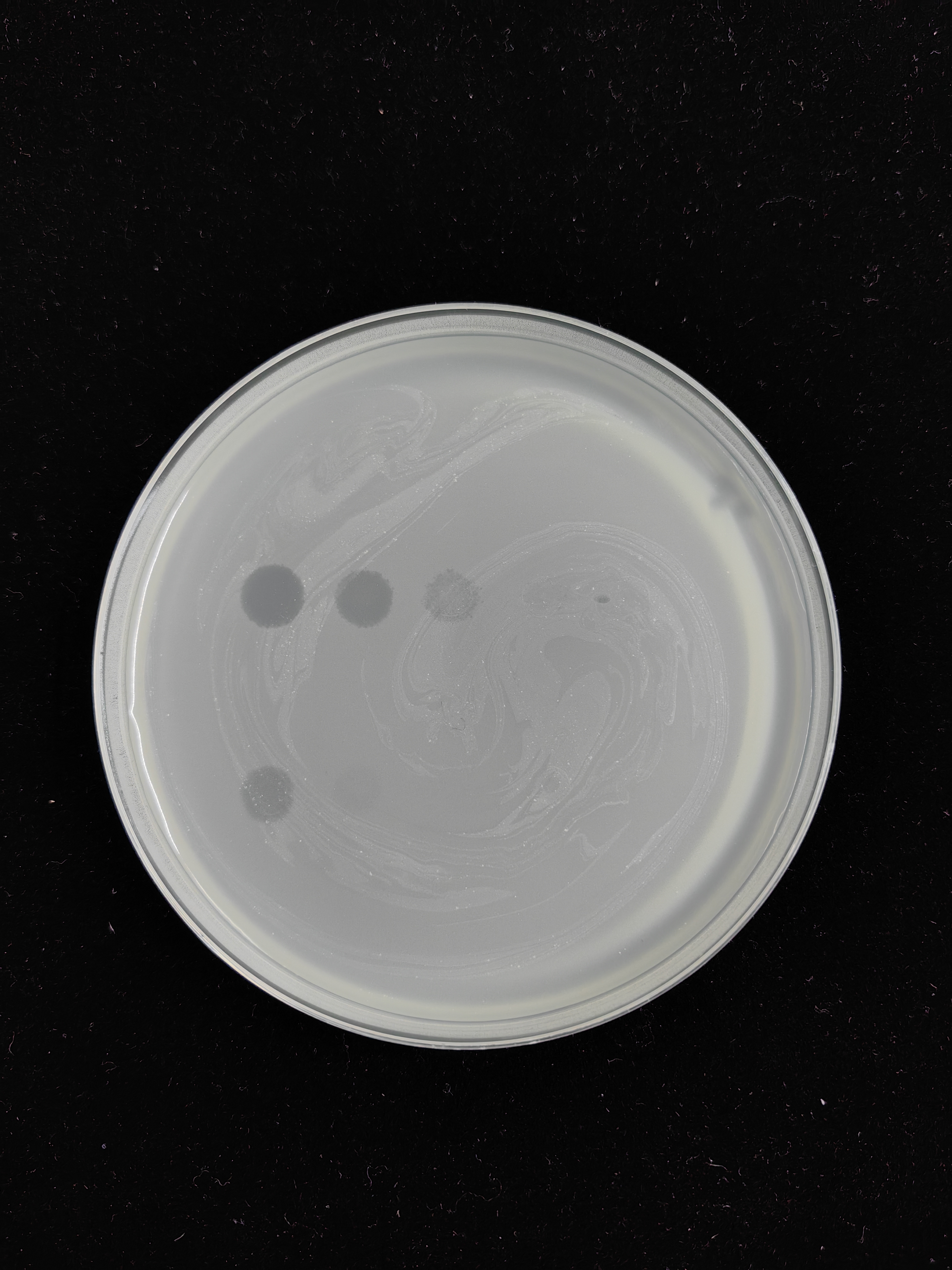

Supplement: Supplementary file 11 — Figure S6 Source Data [file 44319_2025_488_MOESM11_ESM.zip › Appendix Figure S6/S6A/pJR962-Mra_1685 with ATc induction.tiff]

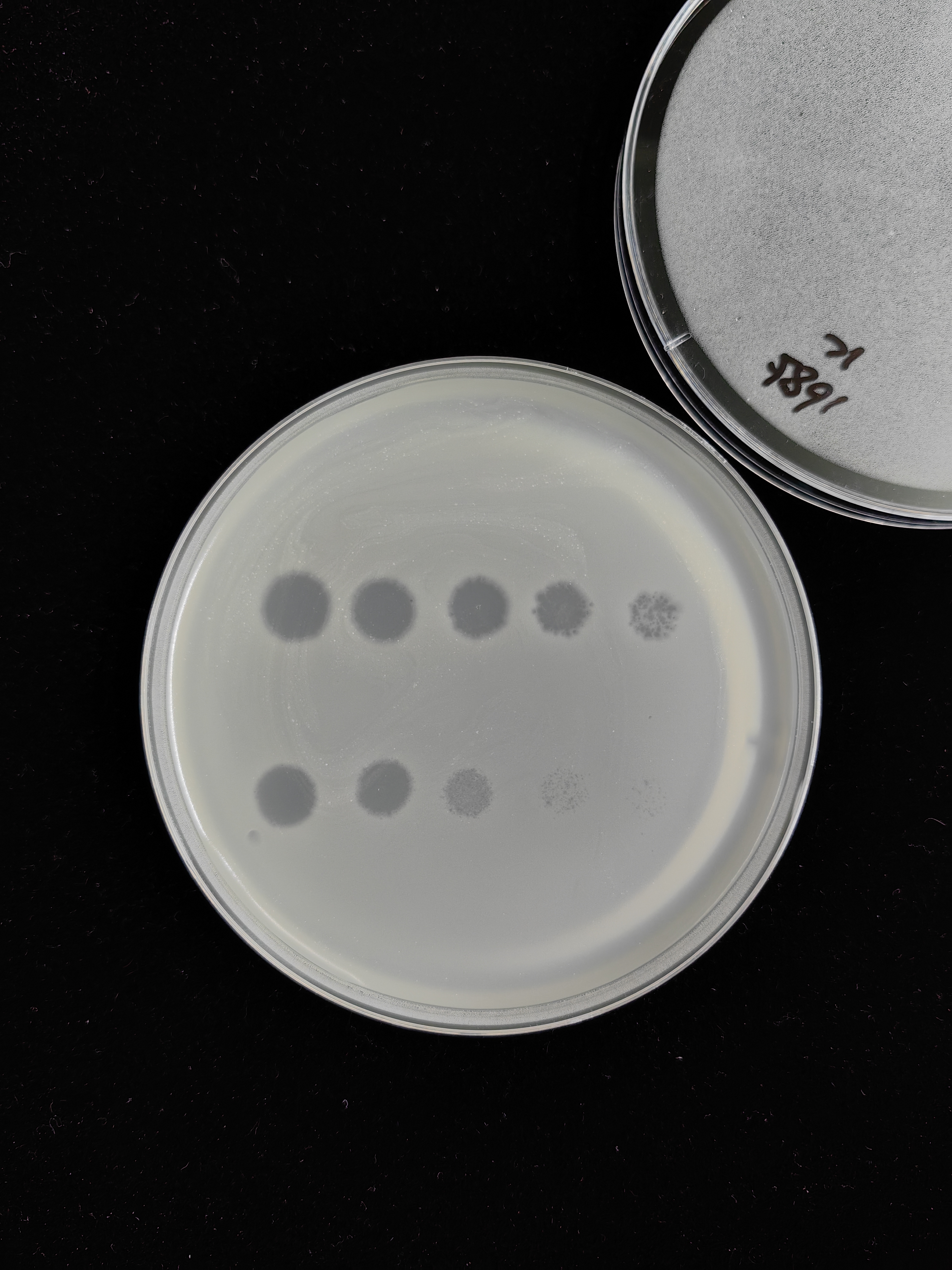

Supplement: Supplementary file 11 — Figure S6 Source Data [file 44319_2025_488_MOESM11_ESM.zip › Appendix Figure S6/S6A/pJR962-Mra_1685 without ATc induction.tiff]

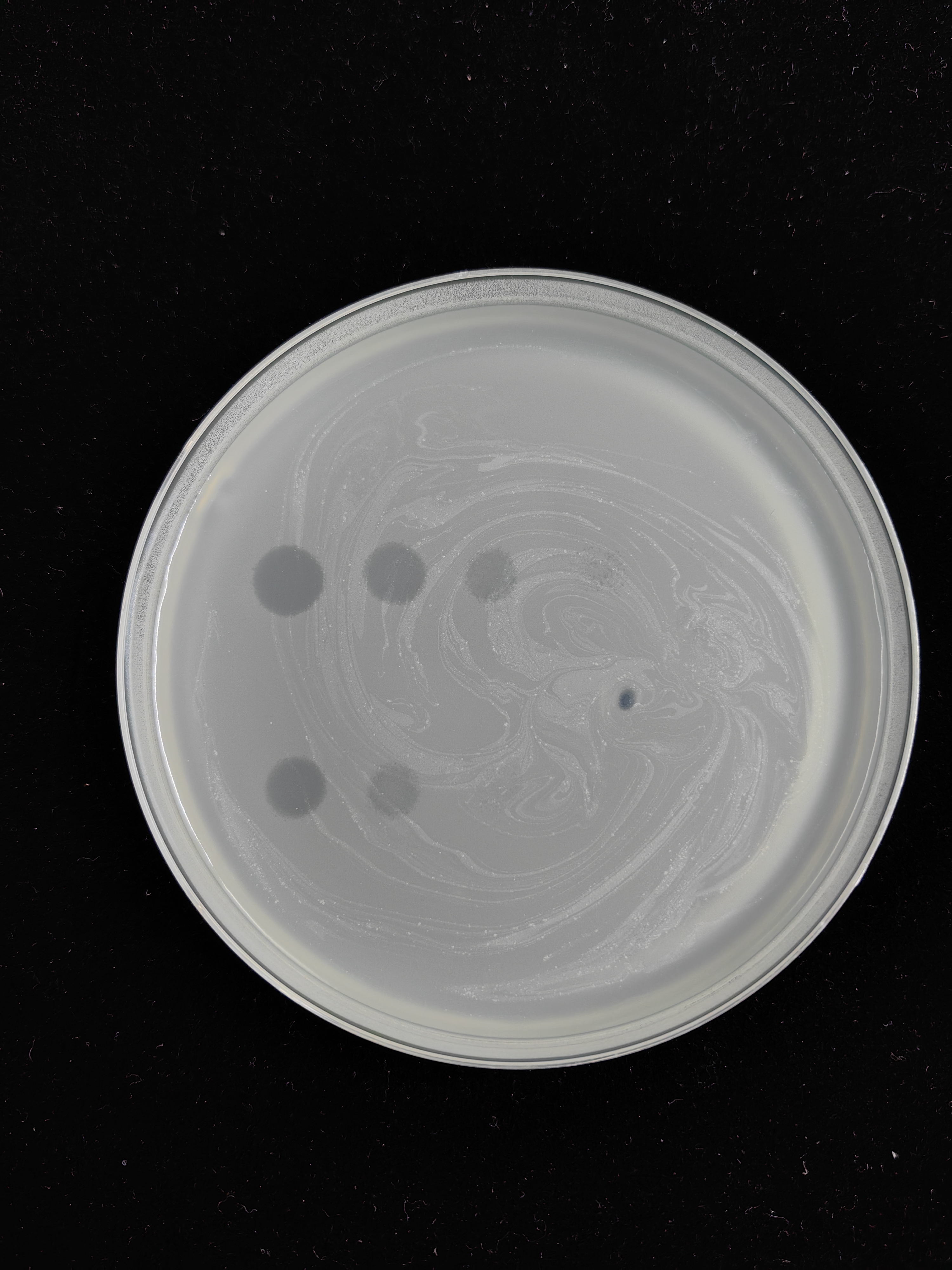

Supplement: Supplementary file 11 — Figure S6 Source Data [file 44319_2025_488_MOESM11_ESM.zip › Appendix Figure S6/S6A/pJR962-Mra_1940A with ATc induction.tiff]

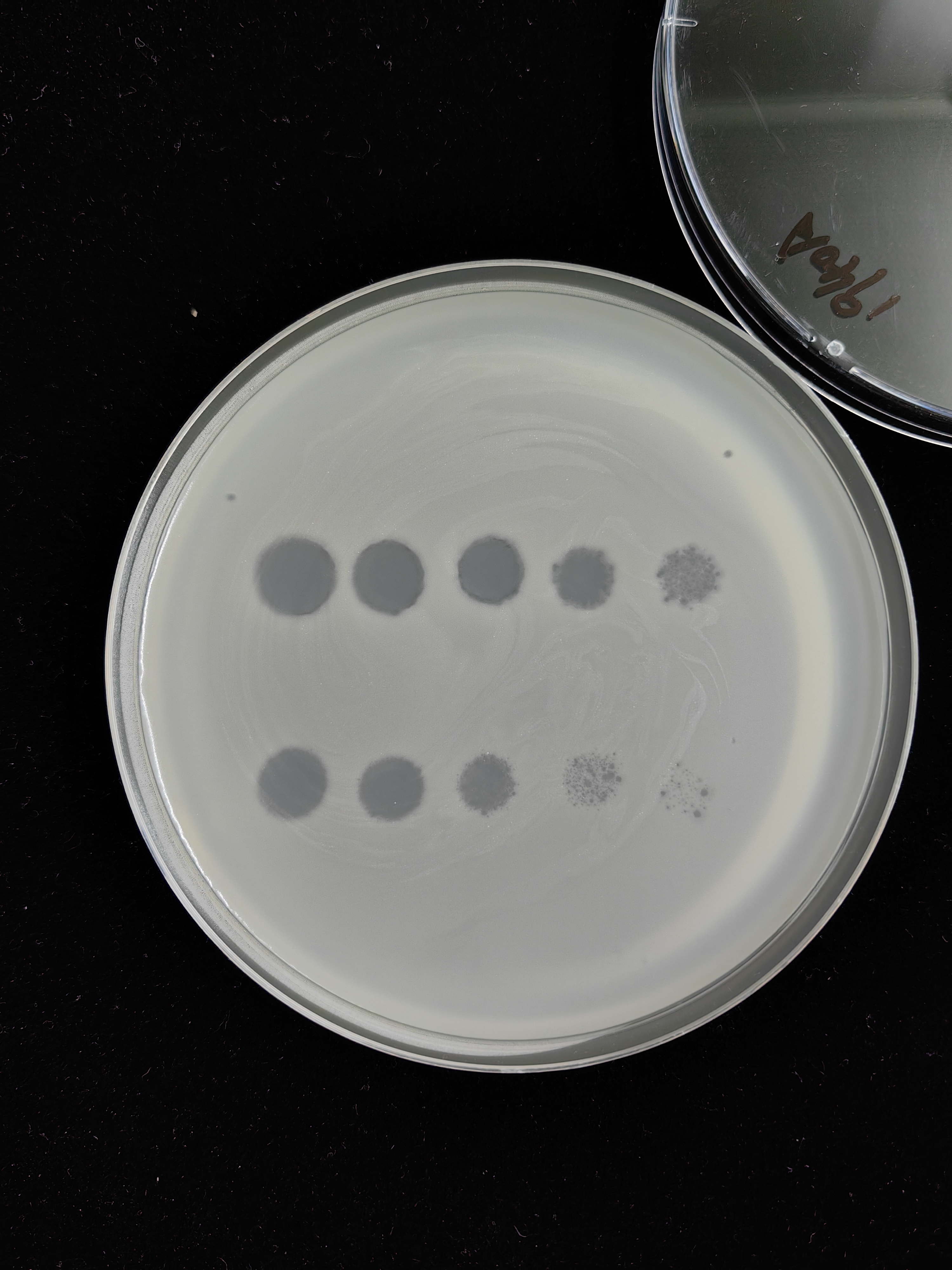

Supplement: Supplementary file 11 — Figure S6 Source Data [file 44319_2025_488_MOESM11_ESM.zip › Appendix Figure S6/S6A/pJR962-Mra_1940A without ATc induction.tiff]

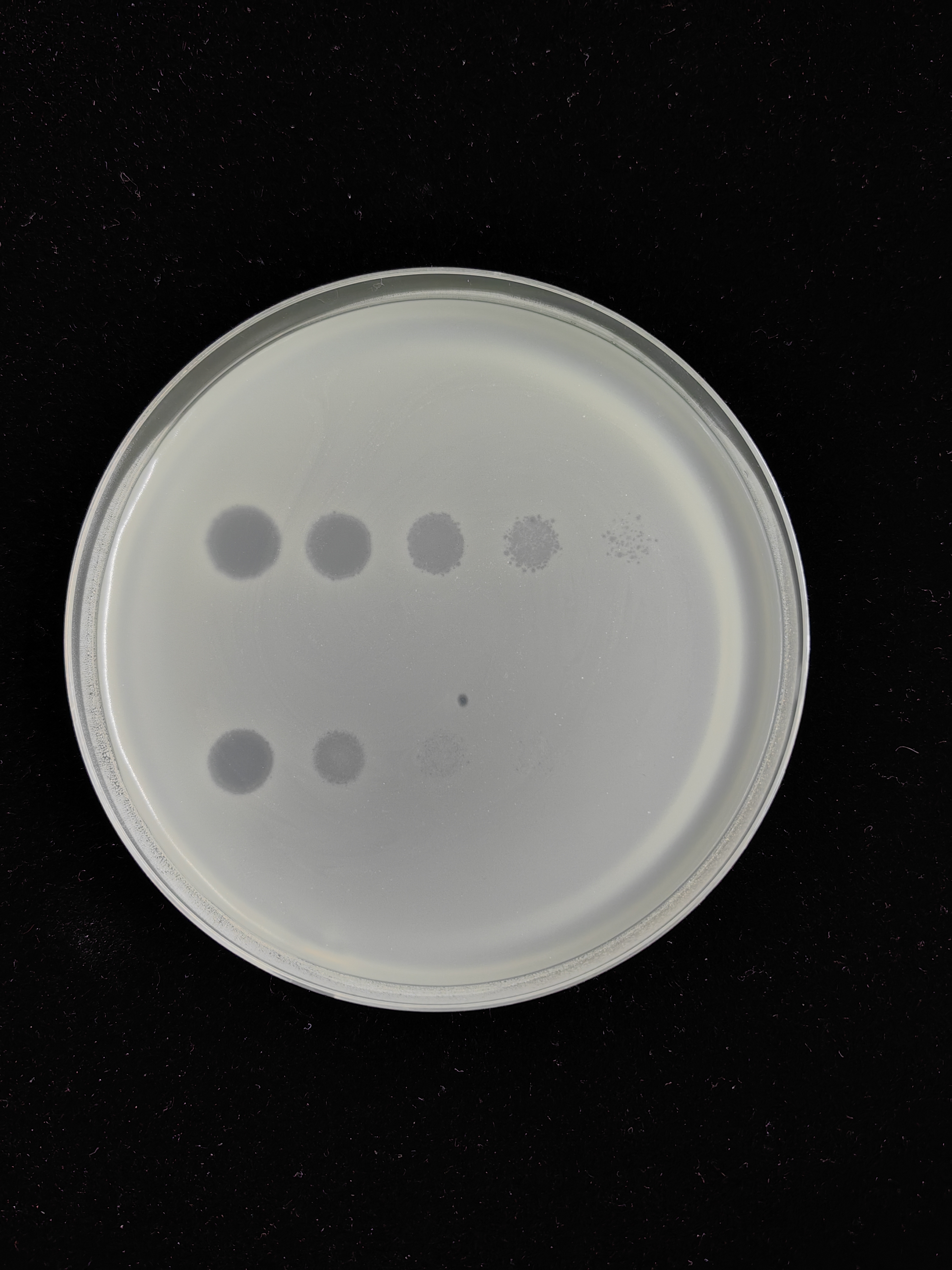

Supplement: Supplementary file 11 — Figure S6 Source Data [file 44319_2025_488_MOESM11_ESM.zip › Appendix Figure S6/S6A/pJR962-Mra_1960 with ATc induction.tiff]

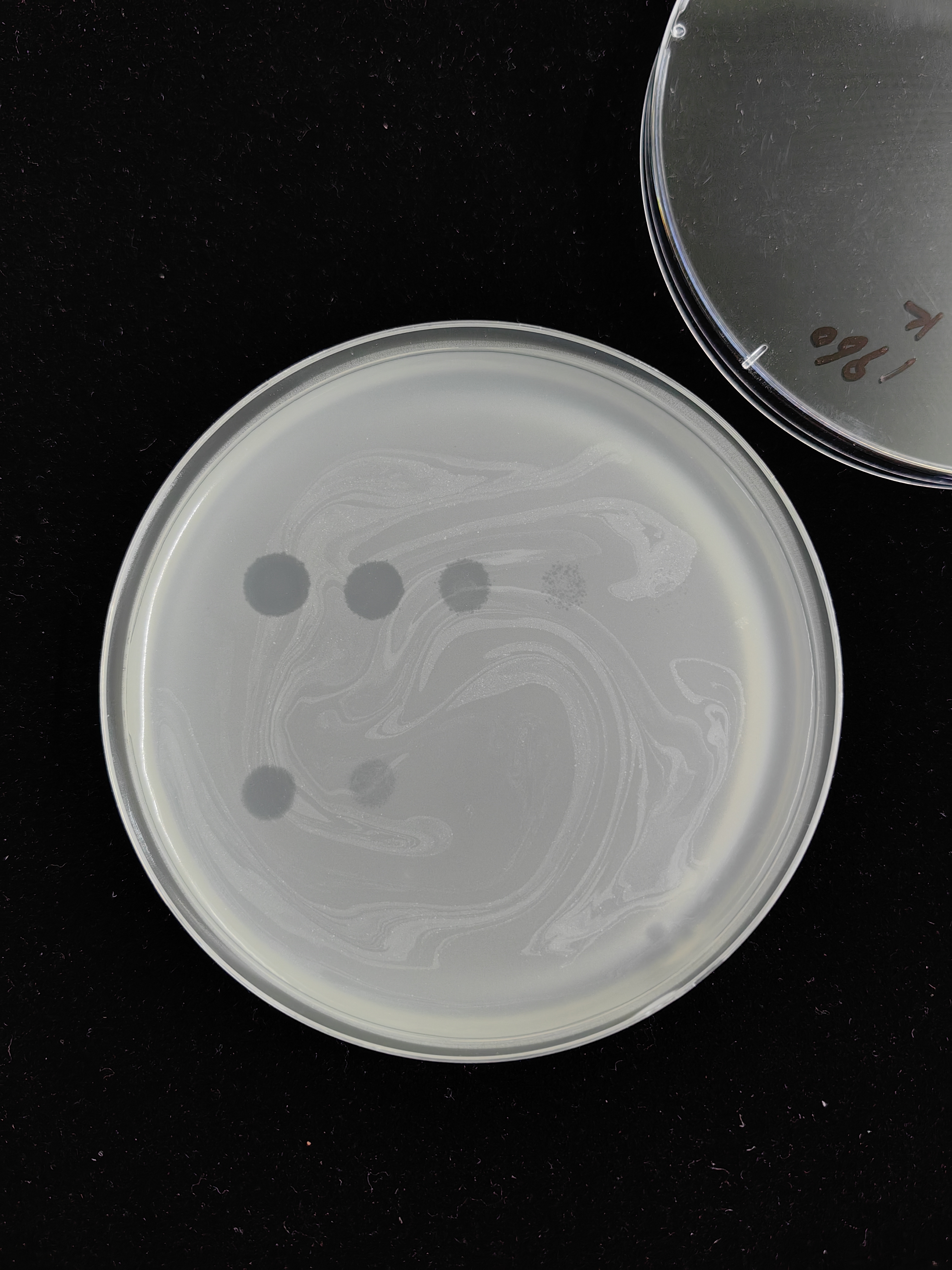

Supplement: Supplementary file 11 — Figure S6 Source Data [file 44319_2025_488_MOESM11_ESM.zip › Appendix Figure S6/S6A/pJR962-Mra_1960 without ATc induction.tiff]

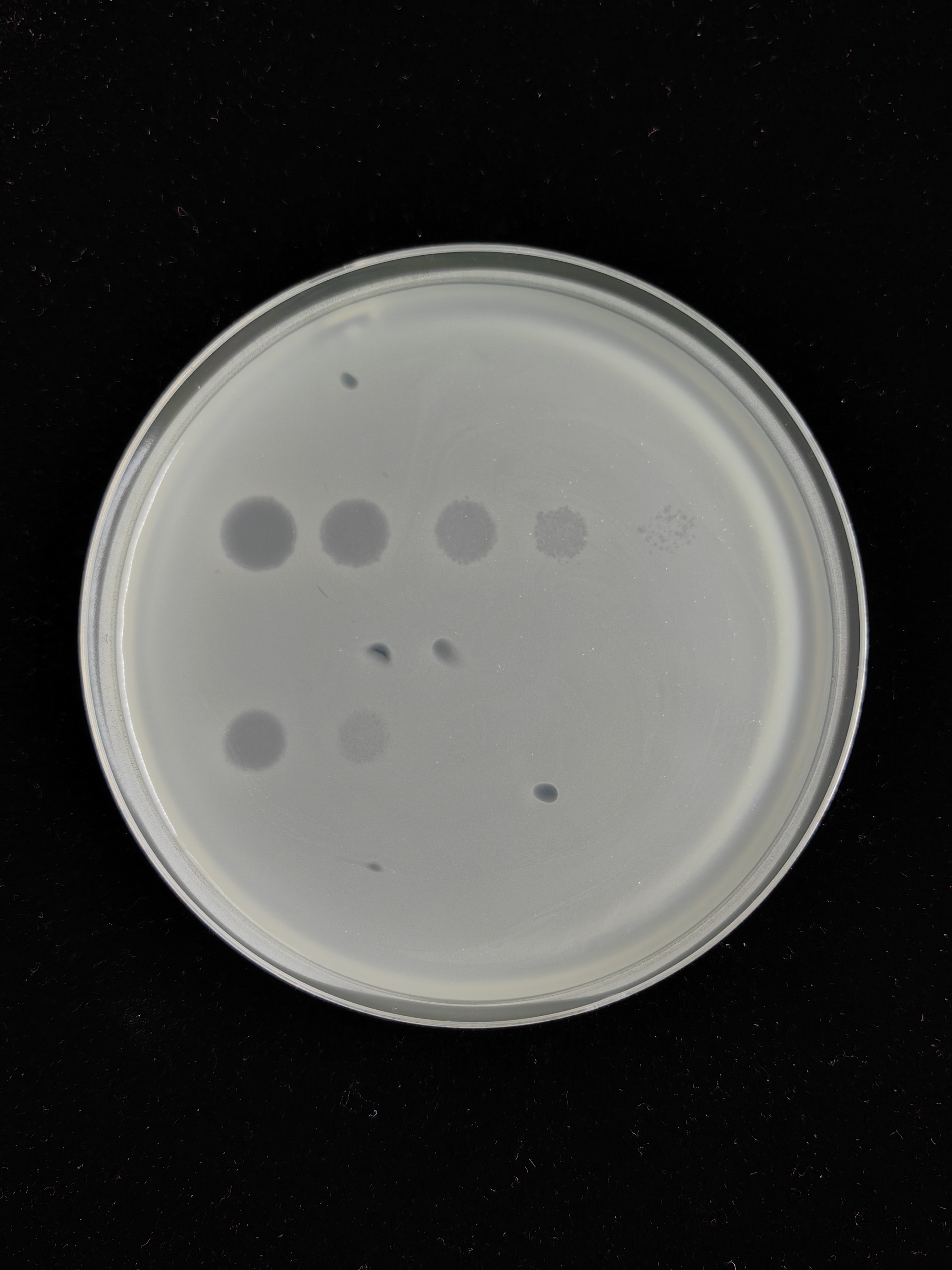

Supplement: Supplementary file 11 — Figure S6 Source Data [file 44319_2025_488_MOESM11_ESM.zip › Appendix Figure S6/S6A/pJR962-Mra_2329A with ATc induction.tiff]

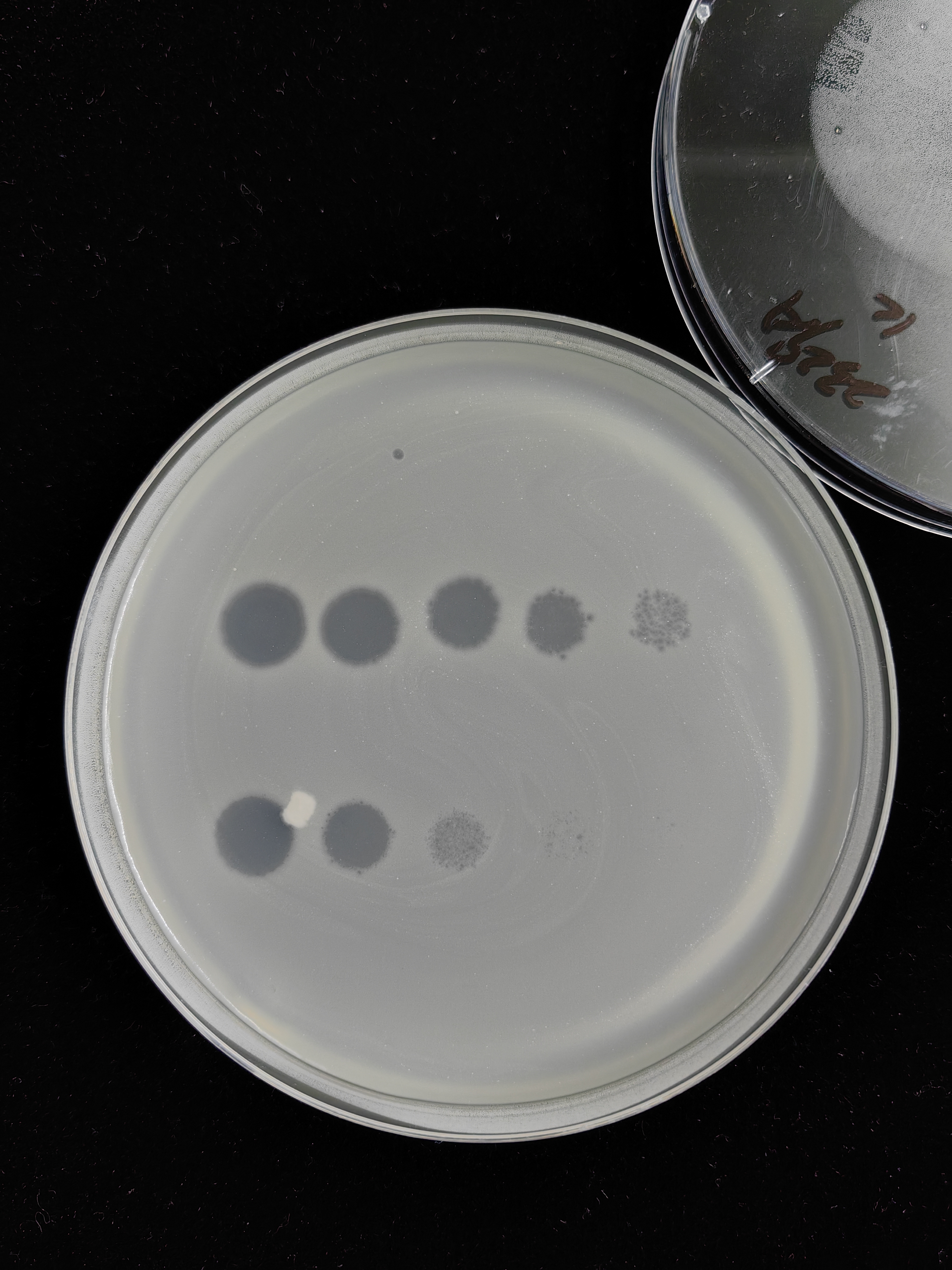

Supplement: Supplementary file 11 — Figure S6 Source Data [file 44319_2025_488_MOESM11_ESM.zip › Appendix Figure S6/S6A/pJR962-Mra_2329A without ATc induction.tiff]

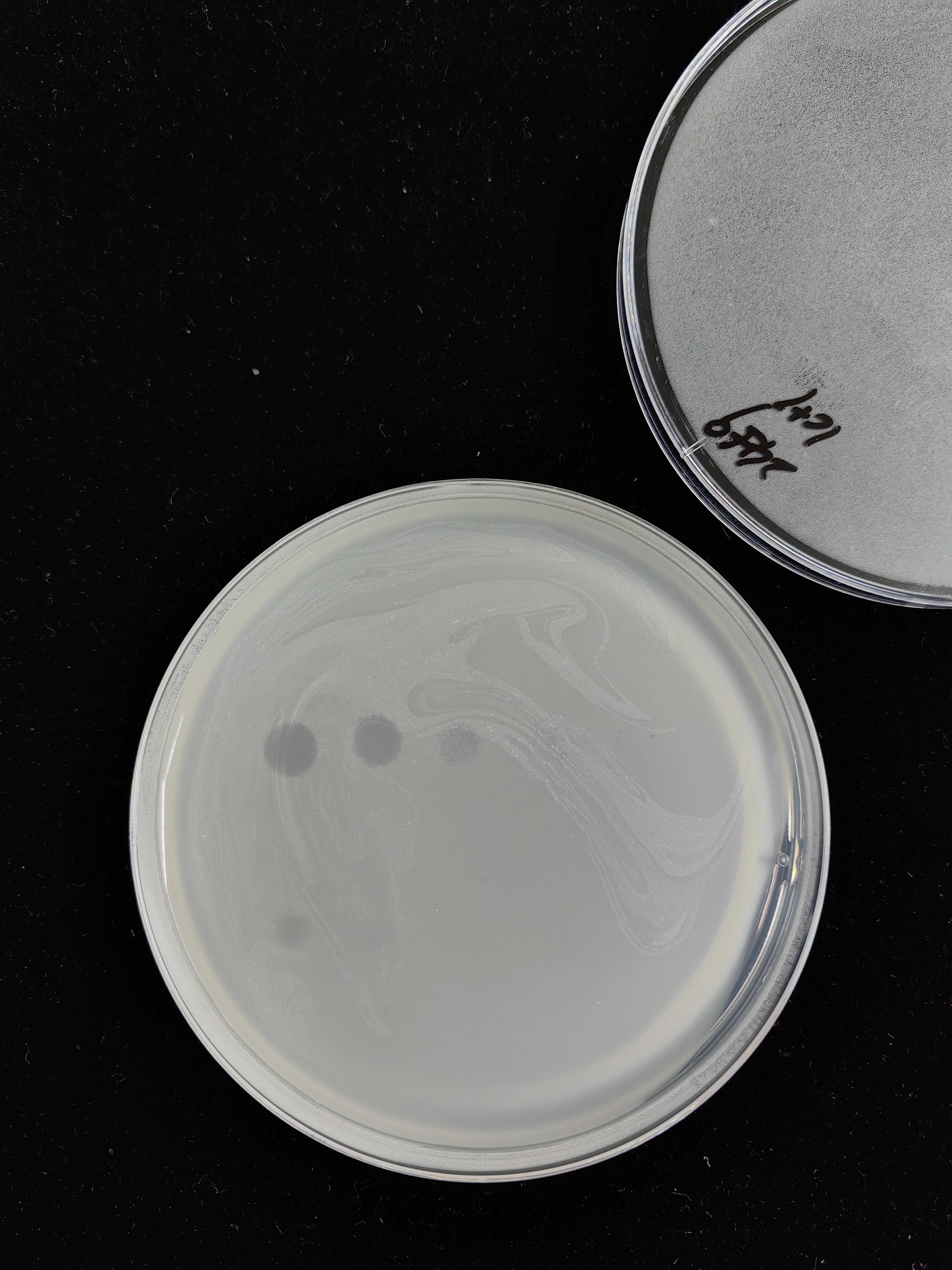

Supplement: Supplementary file 11 — Figure S6 Source Data [file 44319_2025_488_MOESM11_ESM.zip › Appendix Figure S6/S6A/pJR962-Mra_2459 with ATc induction.tiff]

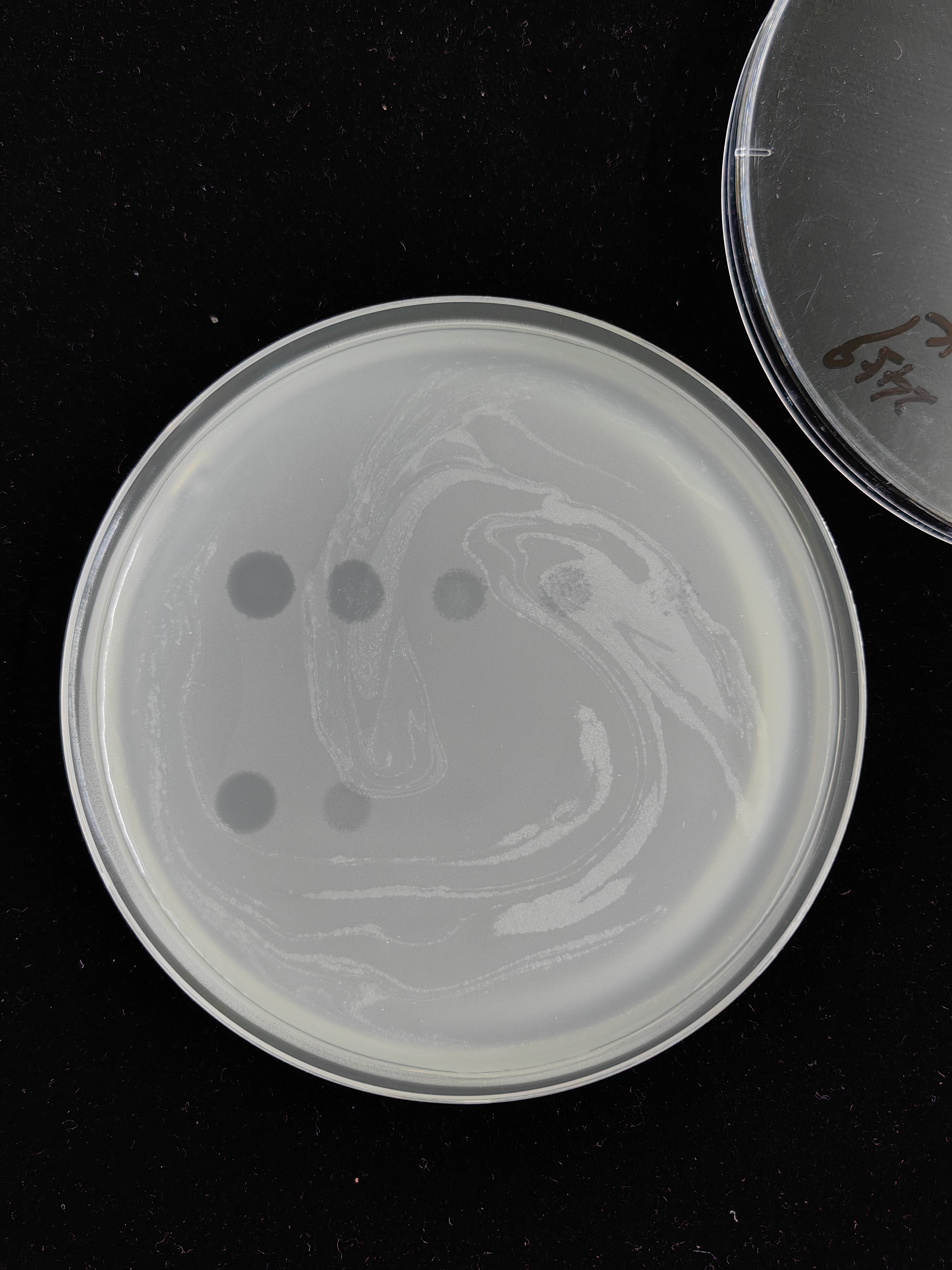

Supplement: Supplementary file 11 — Figure S6 Source Data [file 44319_2025_488_MOESM11_ESM.zip › Appendix Figure S6/S6A/pJR962-Mra_2459 without ATc induction.tiff]

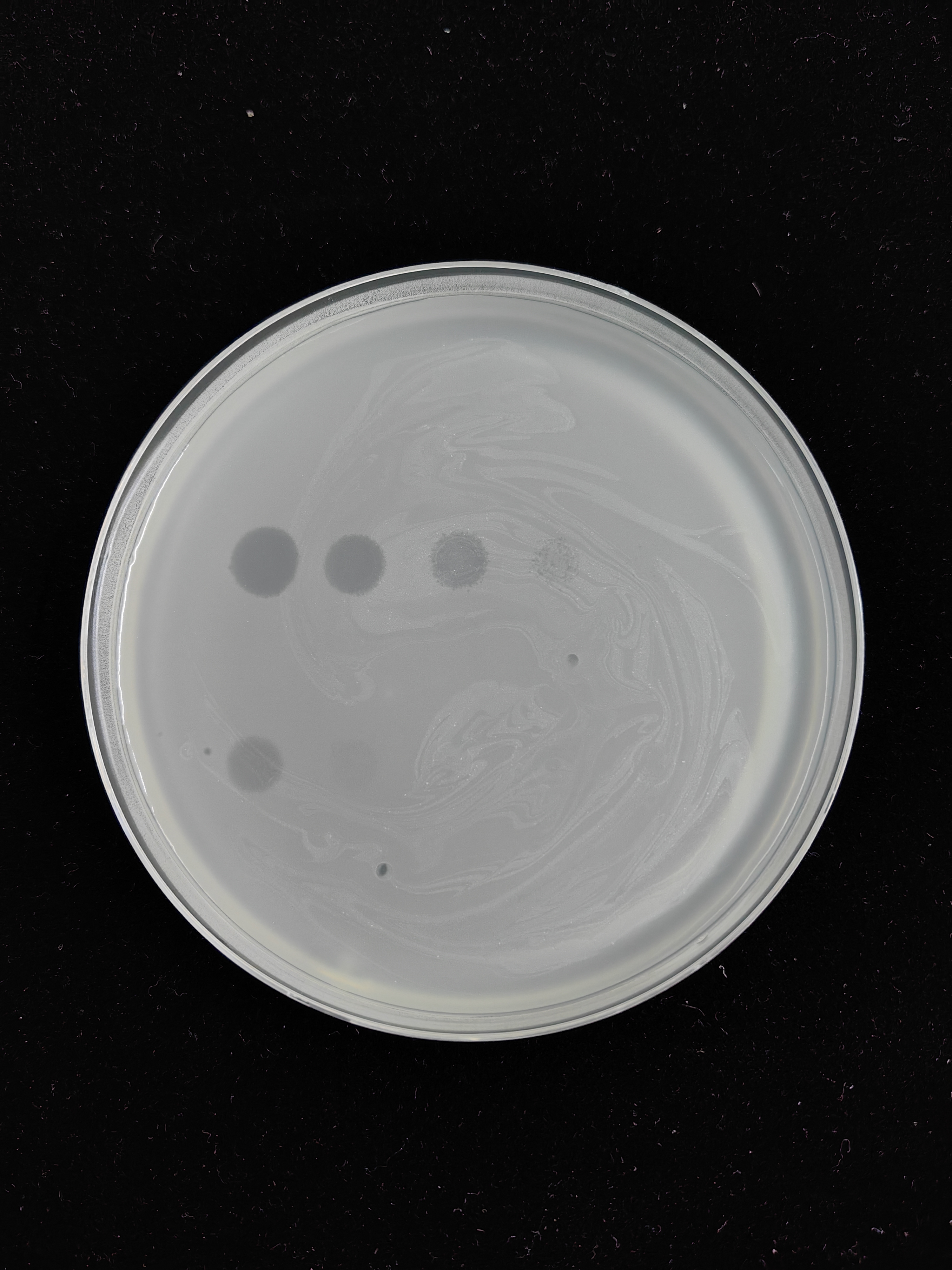

Supplement: Supplementary file 11 — Figure S6 Source Data [file 44319_2025_488_MOESM11_ESM.zip › Appendix Figure S6/S6A/pJR962-Mra_2538 with ATc induction.tiff]

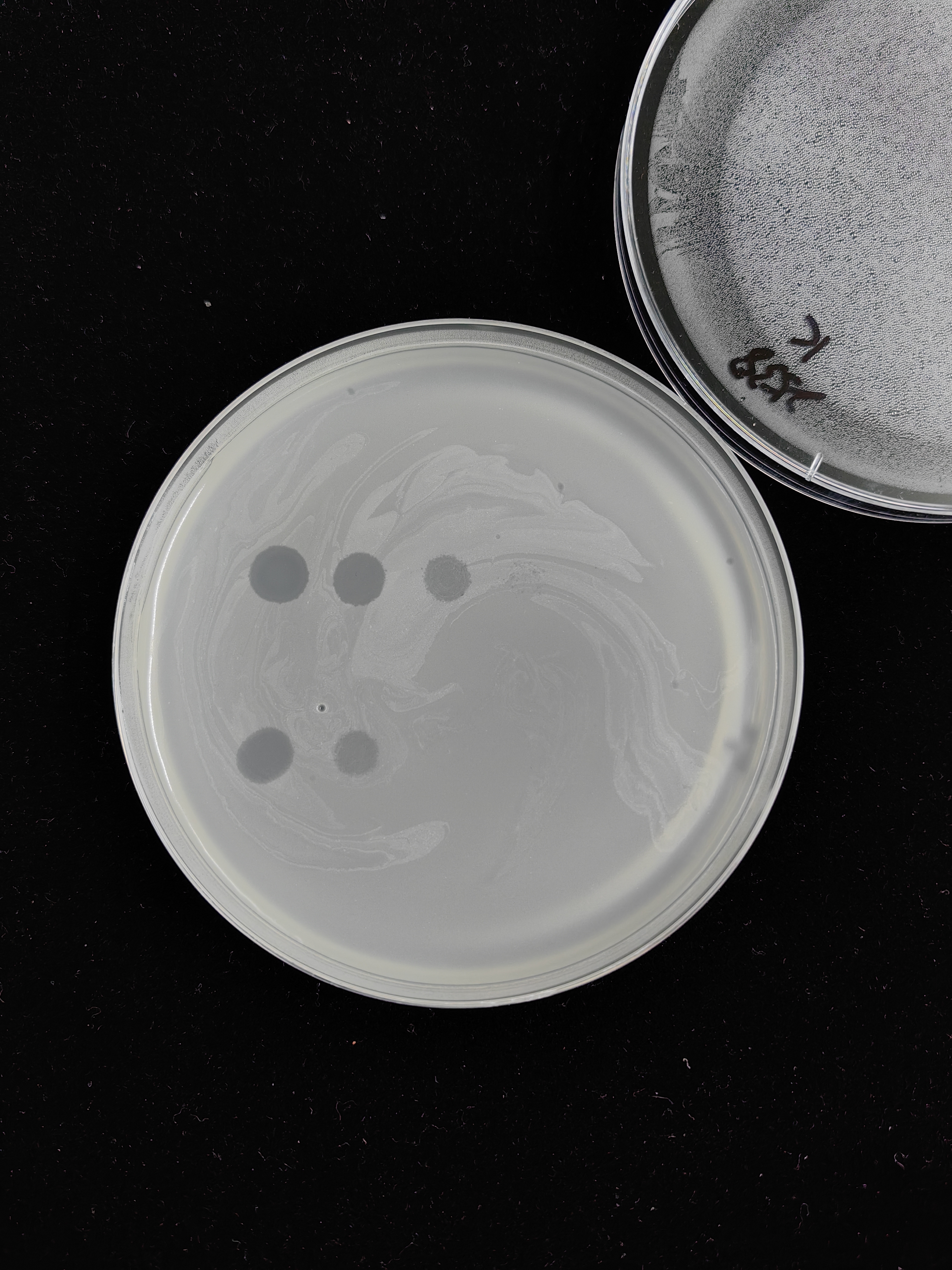

Supplement: Supplementary file 11 — Figure S6 Source Data [file 44319_2025_488_MOESM11_ESM.zip › Appendix Figure S6/S6A/pJR962-Mra_2538 without ATc induction.tiff]

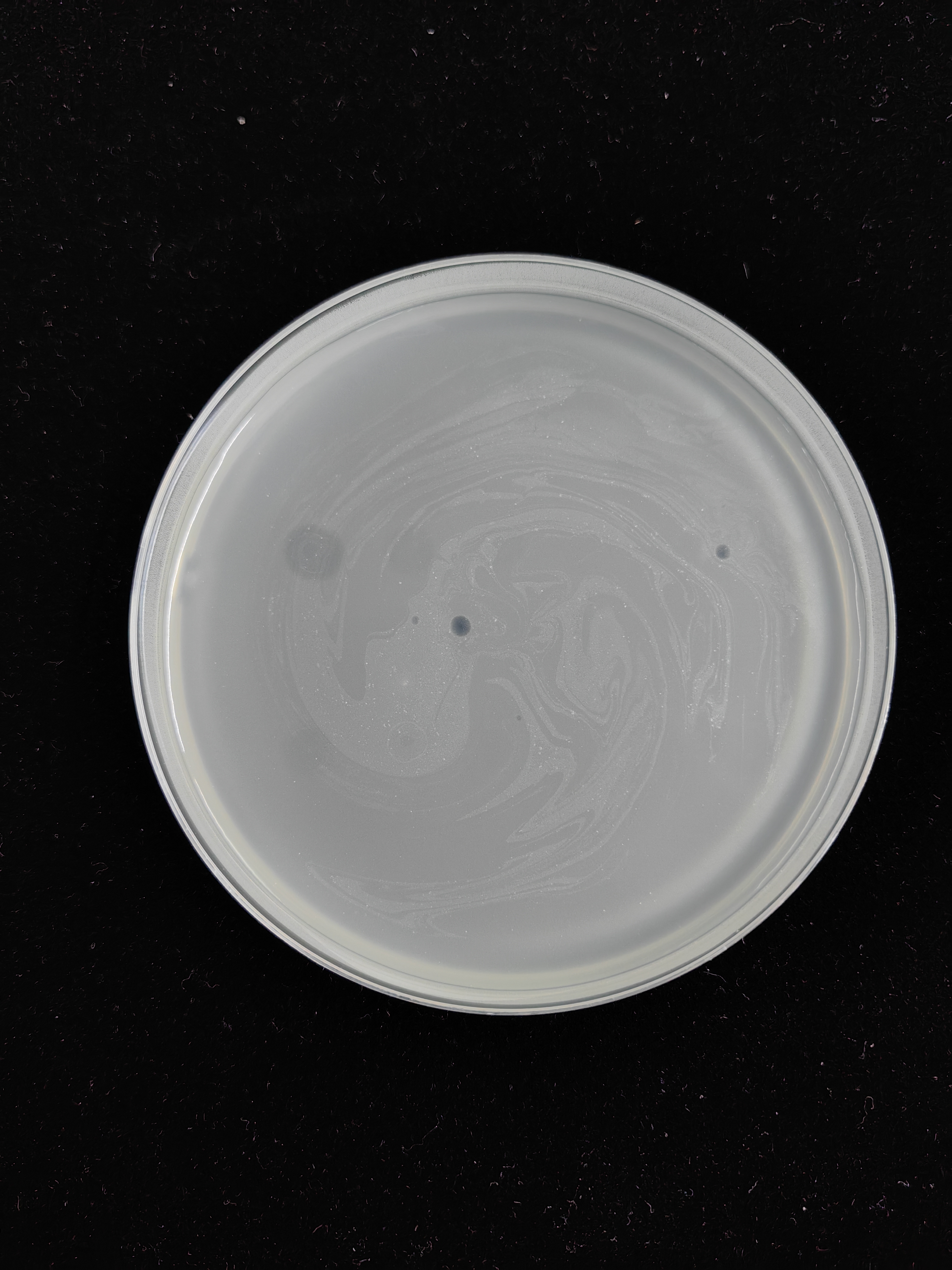

Supplement: Supplementary file 11 — Figure S6 Source Data [file 44319_2025_488_MOESM11_ESM.zip › Appendix Figure S6/S6A/pJR962-Mra_3122 with ATc induction.tiff]

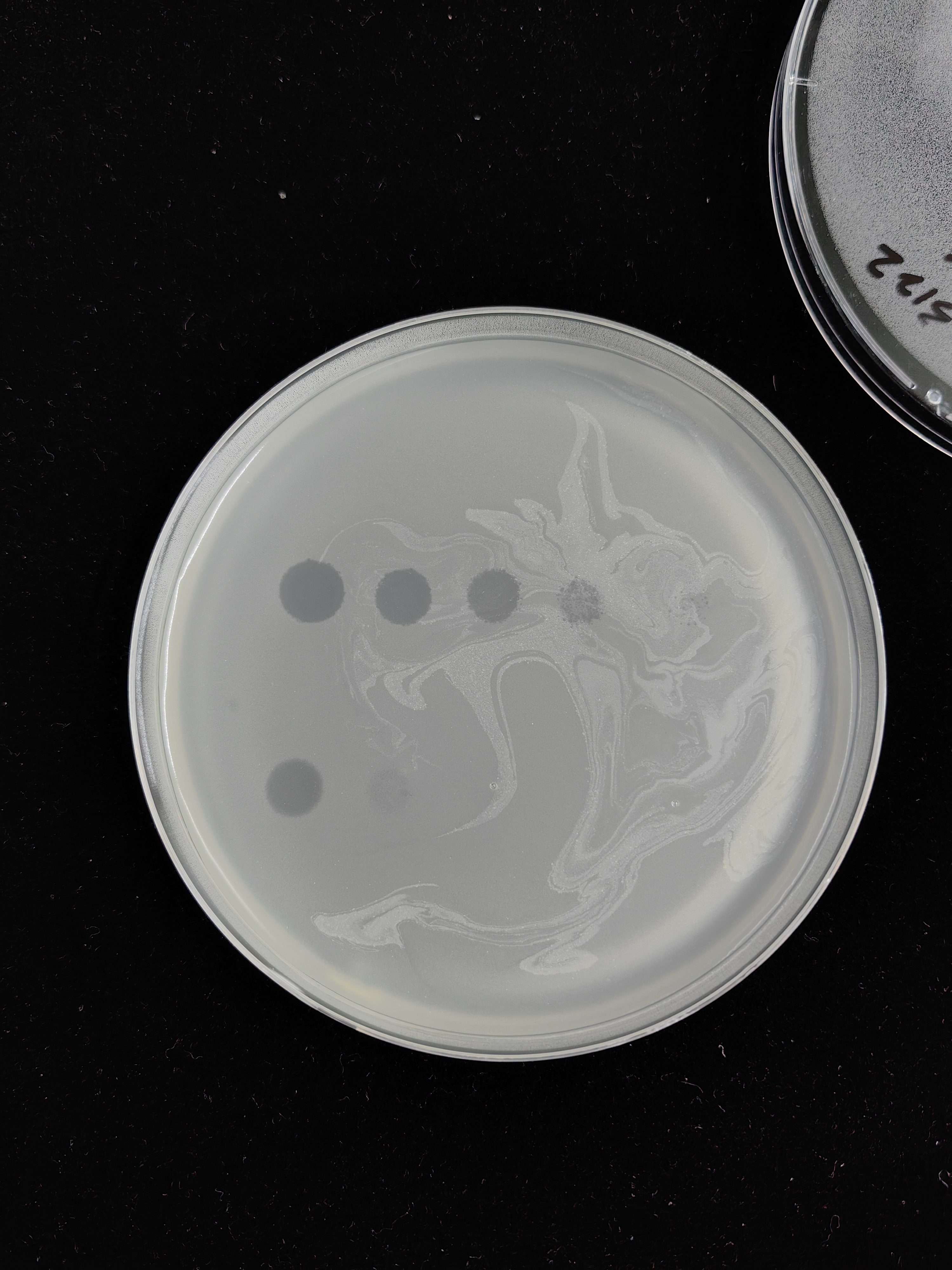

Supplement: Supplementary file 11 — Figure S6 Source Data [file 44319_2025_488_MOESM11_ESM.zip › Appendix Figure S6/S6A/pJR962-Mra_3122 without ATc induction.tiff]

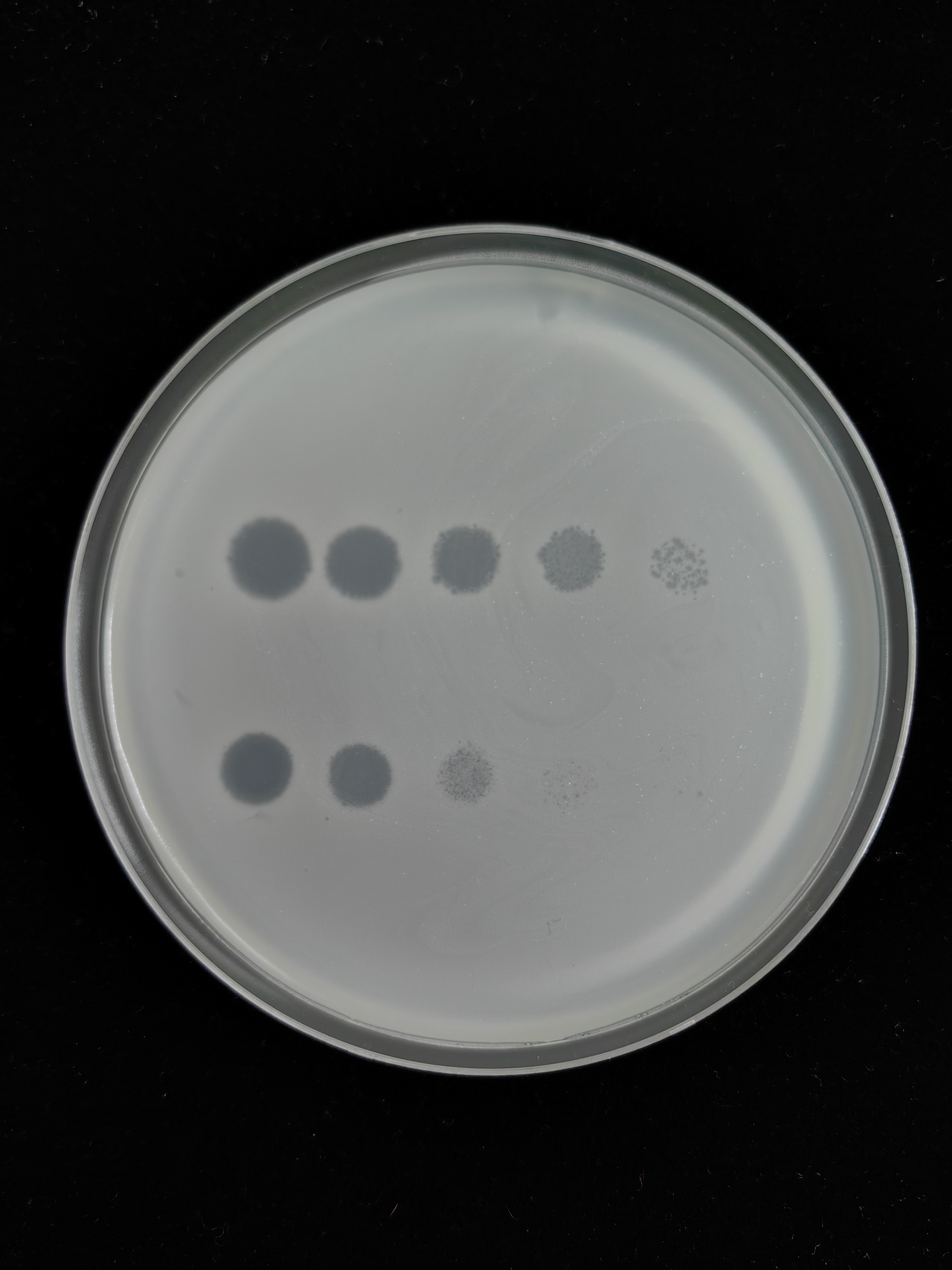

Supplement: Supplementary file 11 — Figure S6 Source Data [file 44319_2025_488_MOESM11_ESM.zip › Appendix Figure S6/S6A/pJR962-Mra_3464 with ATc induction.tiff]

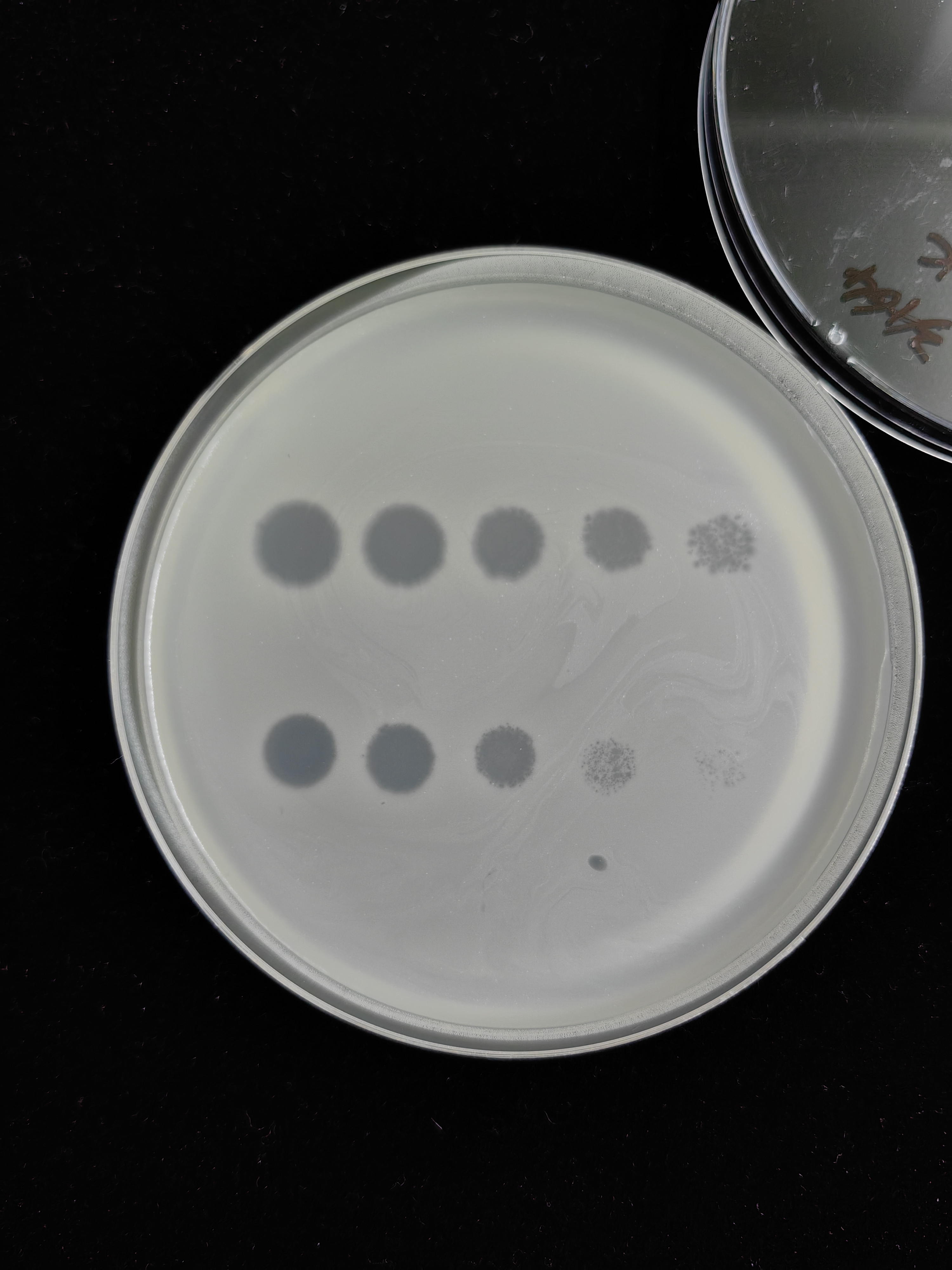

Supplement: Supplementary file 11 — Figure S6 Source Data [file 44319_2025_488_MOESM11_ESM.zip › Appendix Figure S6/S6A/pJR962-Mra_3464 without ATc induction.tiff]

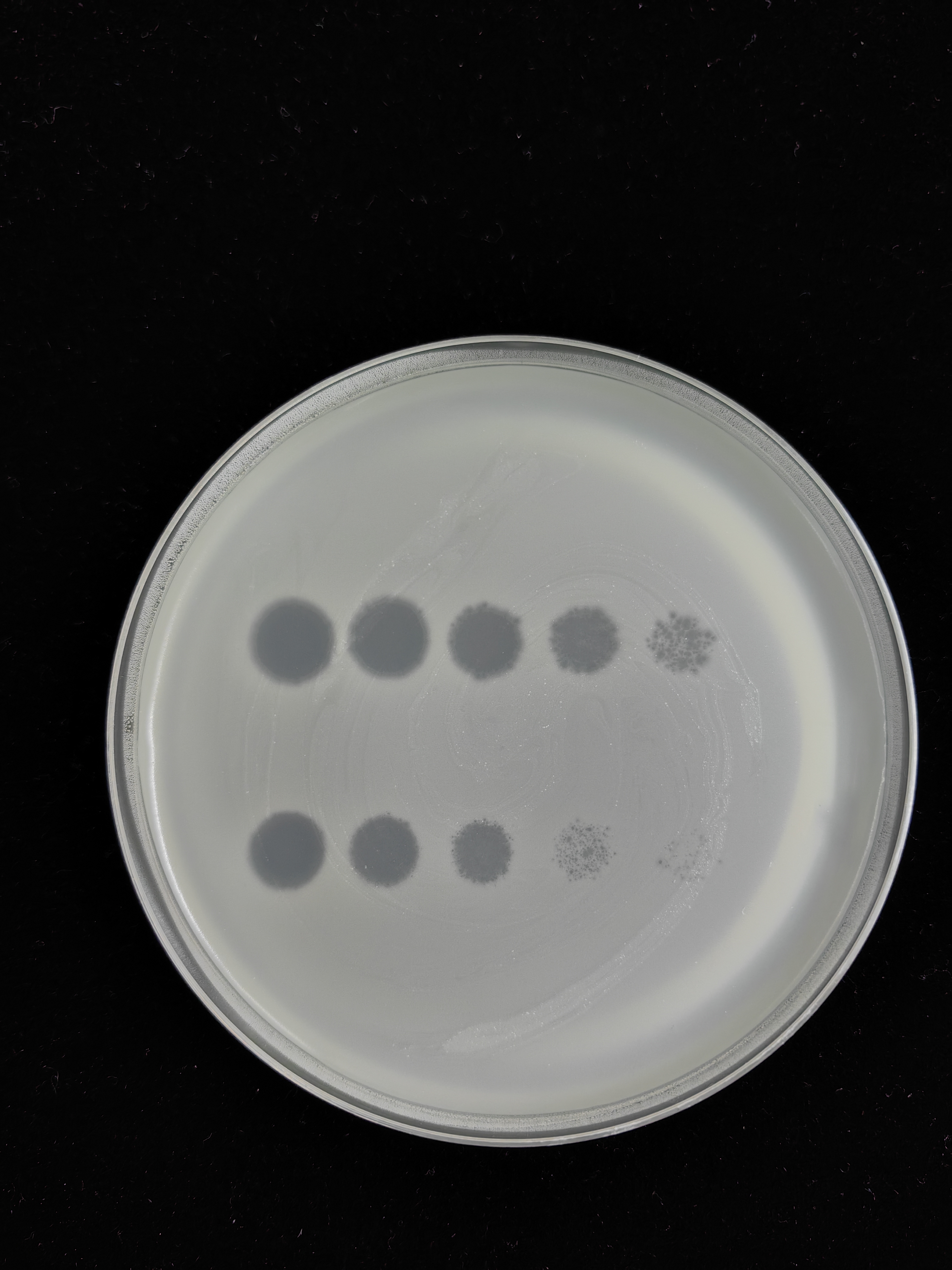

Supplement: Supplementary file 11 — Figure S6 Source Data [file 44319_2025_488_MOESM11_ESM.zip › Appendix Figure S6/S6A/pJR962-Mra_3701 with ATc induction.tiff]

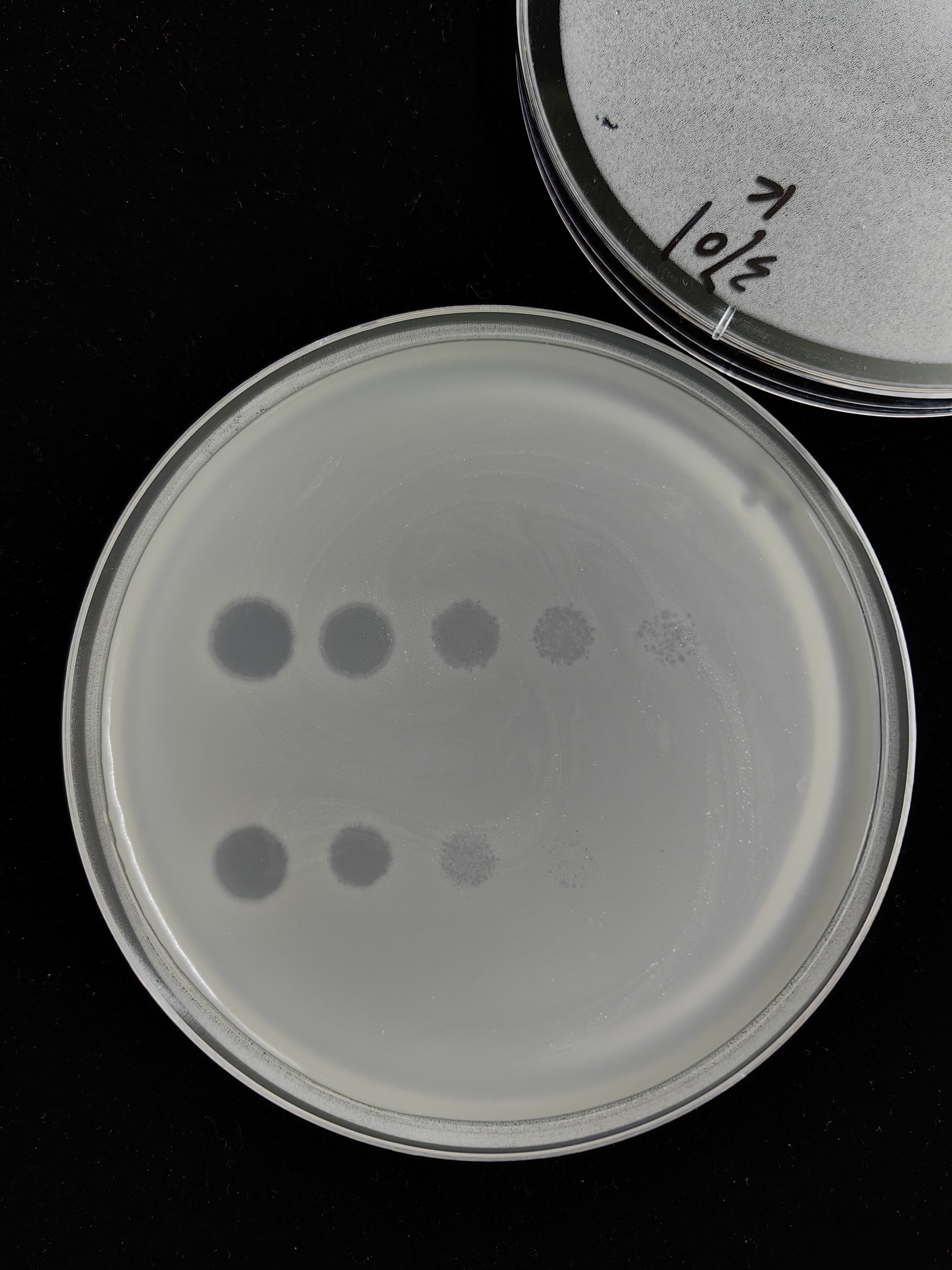

Supplement: Supplementary file 11 — Figure S6 Source Data [file 44319_2025_488_MOESM11_ESM.zip › Appendix Figure S6/S6A/pJR962-Mra_3701 without ATc induction.tiff]

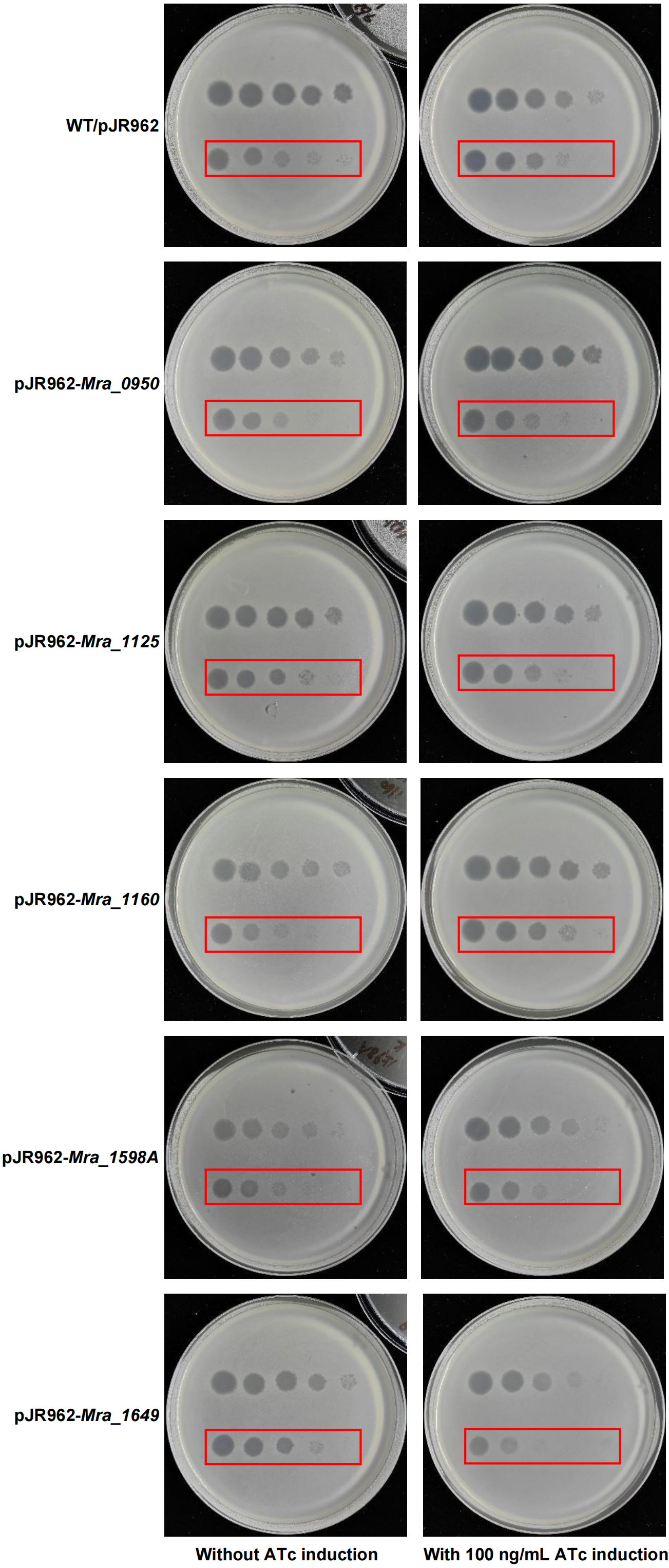

Supplement: Supplementary file 11 — Figure S6 Source Data [file 44319_2025_488_MOESM11_ESM.zip › Appendix Figure S6/S6A/README-1.tif]

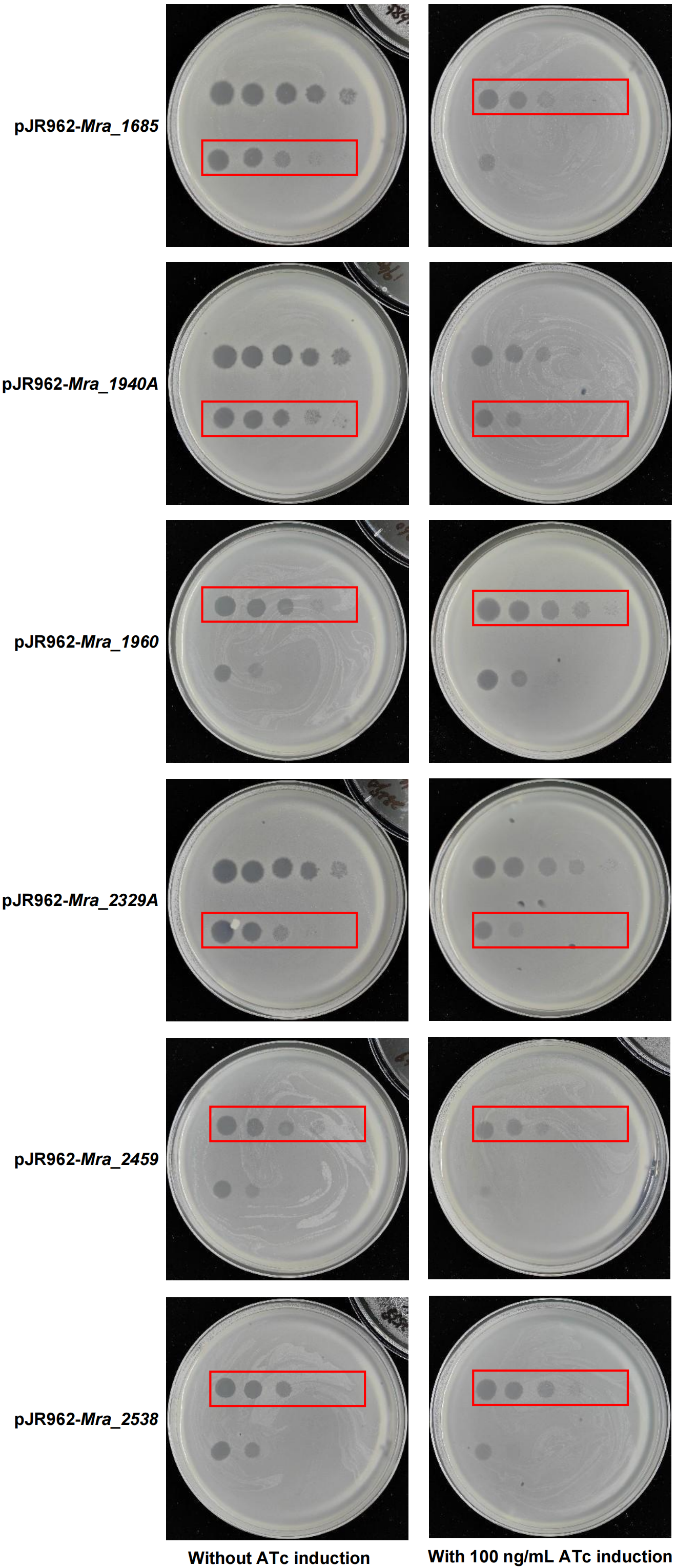

Supplement: Supplementary file 11 — Figure S6 Source Data [file 44319_2025_488_MOESM11_ESM.zip › Appendix Figure S6/S6A/README-2.tif]

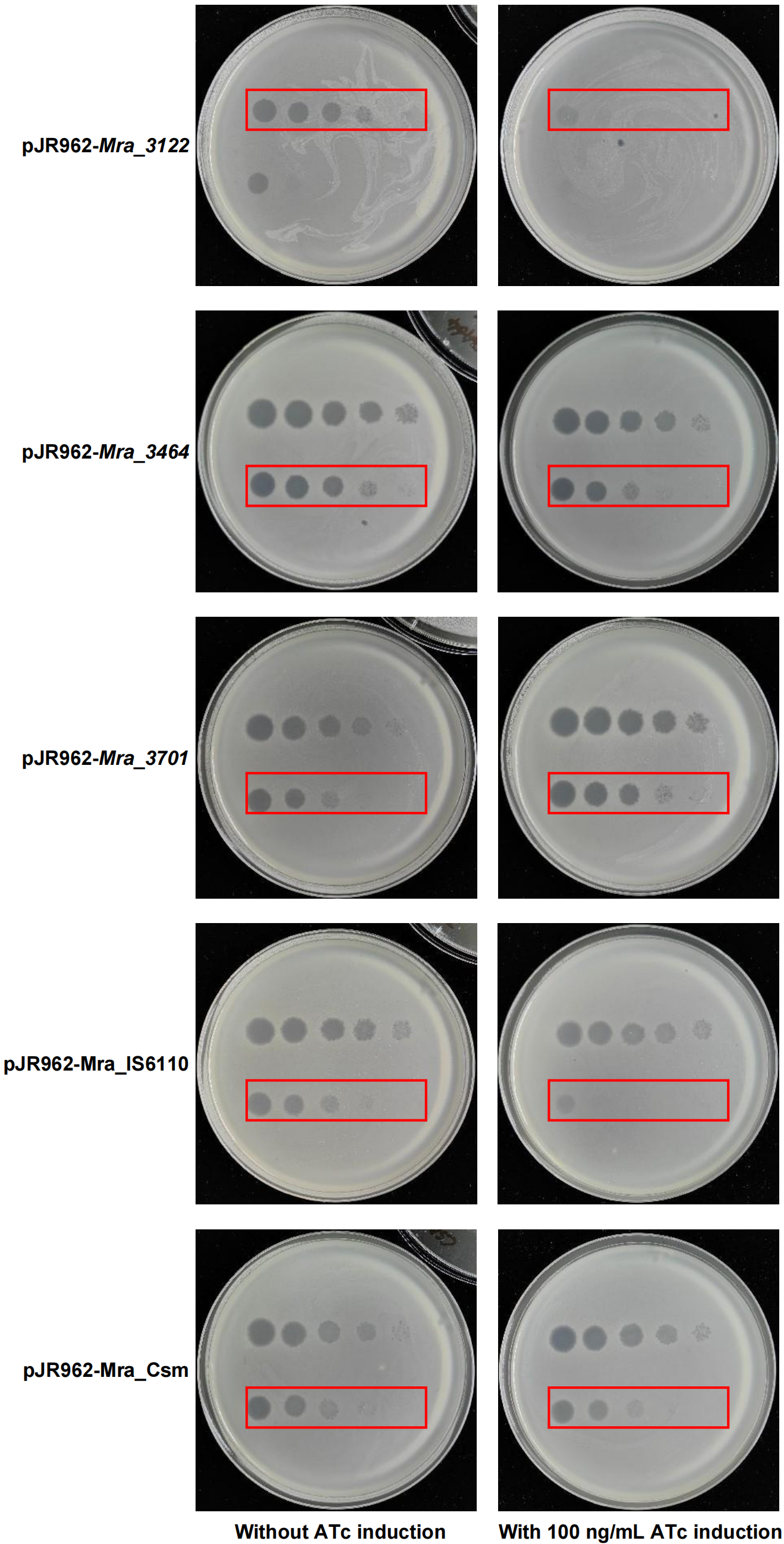

Supplement: Supplementary file 11 — Figure S6 Source Data [file 44319_2025_488_MOESM11_ESM.zip › Appendix Figure S6/S6A/README-3.tif]

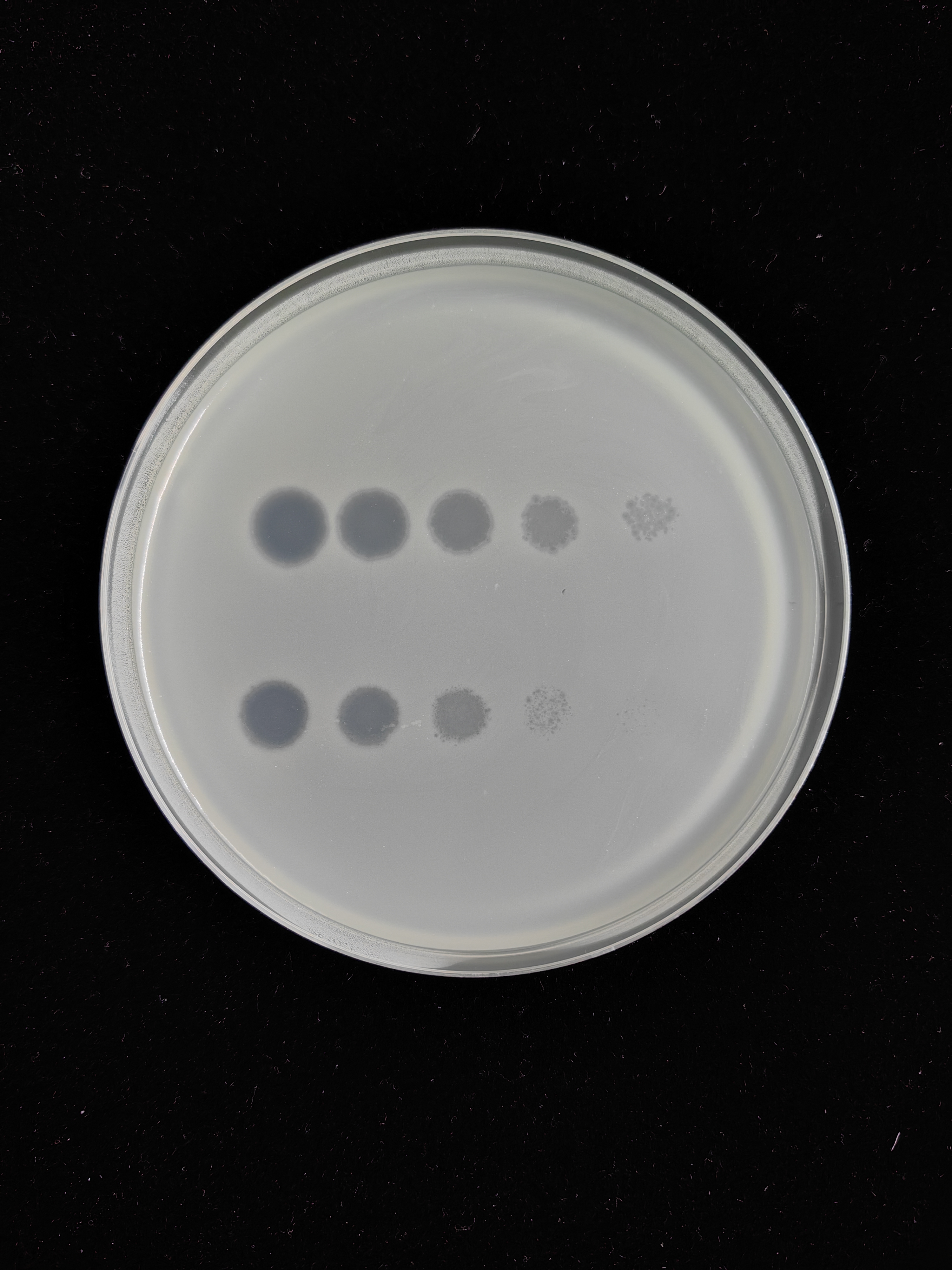

Supplement: Supplementary file 11 — Figure S6 Source Data [file 44319_2025_488_MOESM11_ESM.zip › Appendix Figure S6/S6A/WT-pJR962 with ATc induction.tiff]

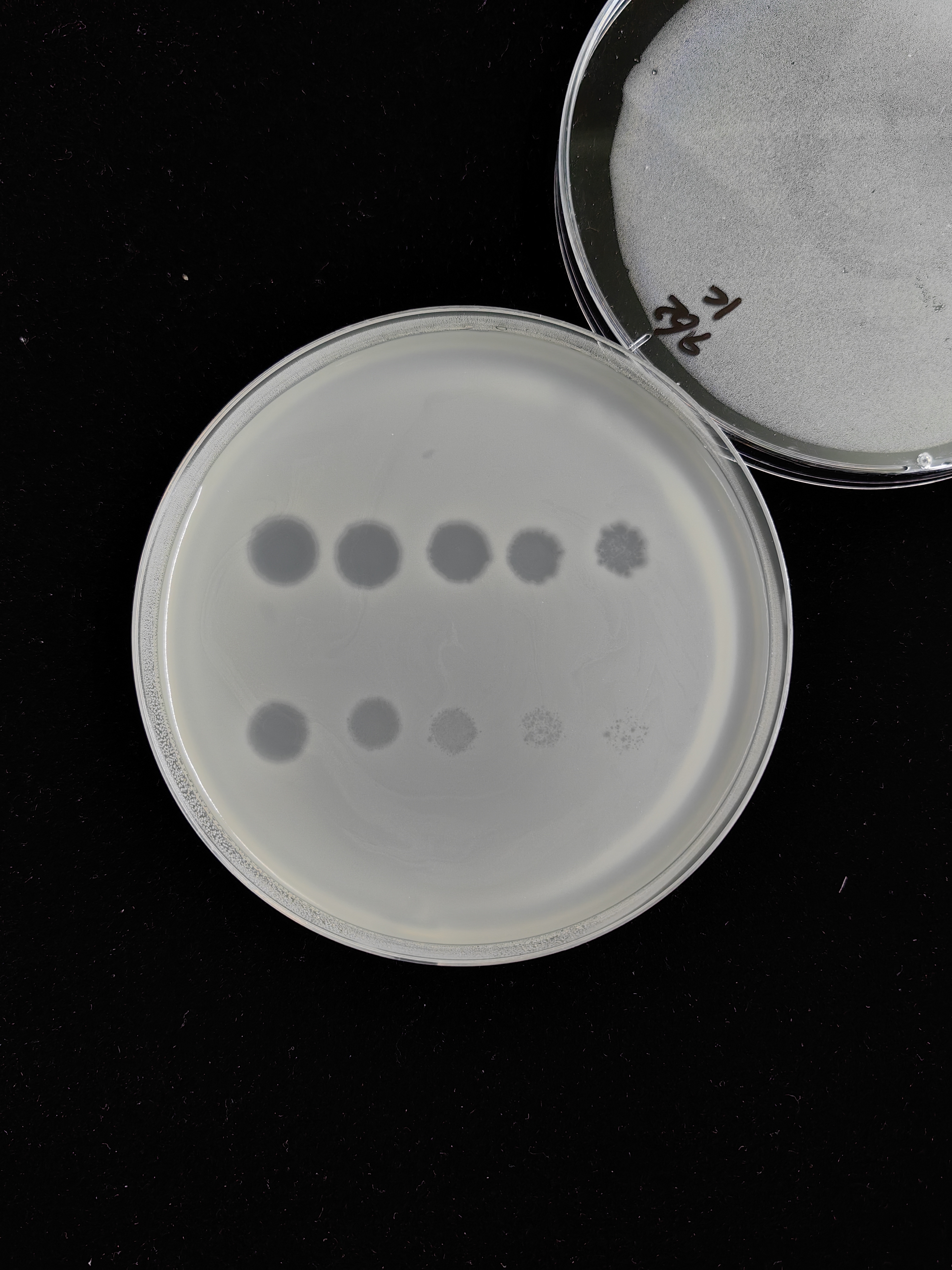

Supplement: Supplementary file 11 — Figure S6 Source Data [file 44319_2025_488_MOESM11_ESM.zip › Appendix Figure S6/S6A/WT-pJR962 without ATc induction.tiff]

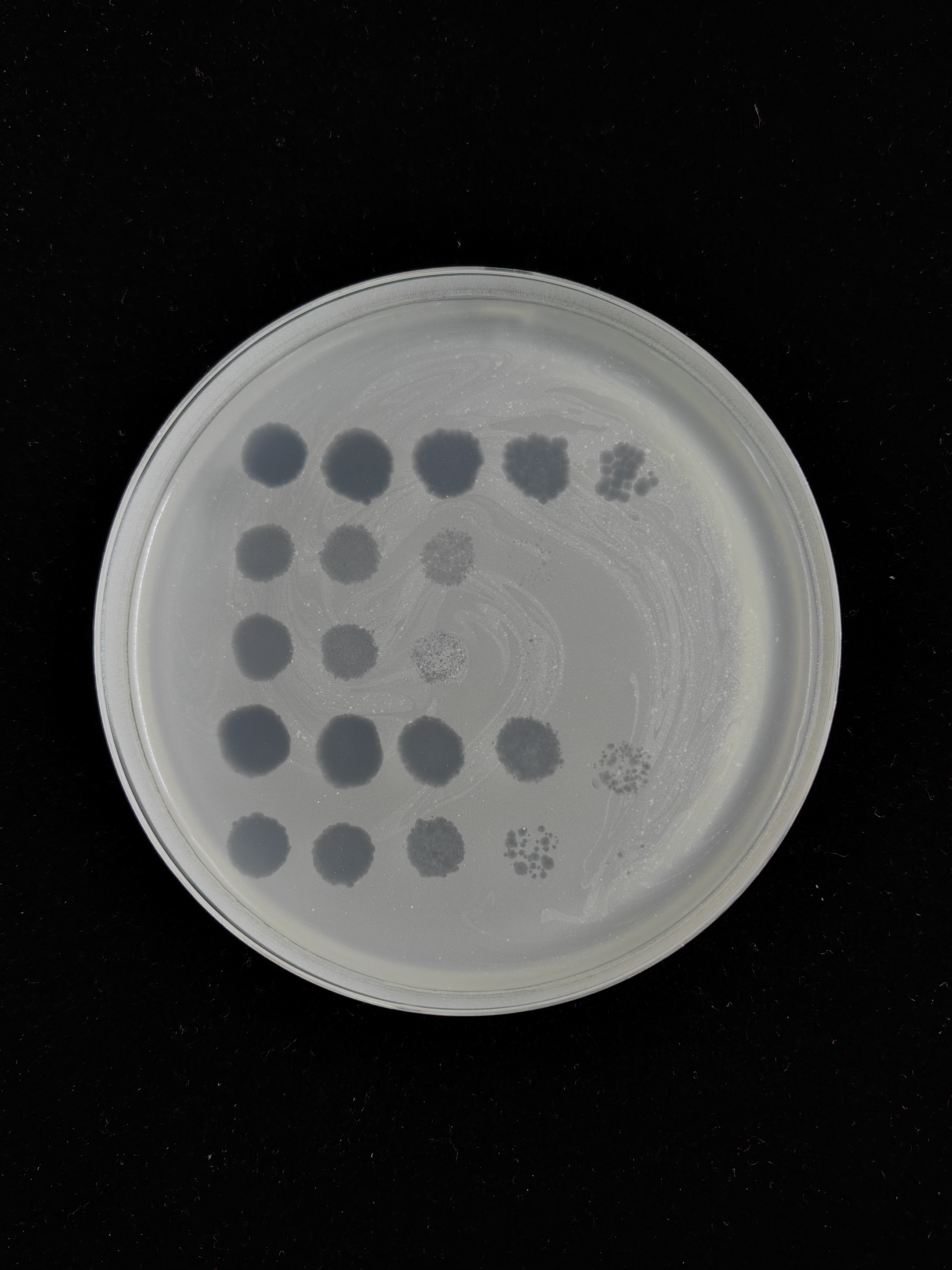

Supplement: Supplementary file 12 — Figure S7 Source Data [file 44319_2025_488_MOESM12_ESM.zip › Appendix Figure S7/S7A/pJR962-1 with ATc induction.tiff]

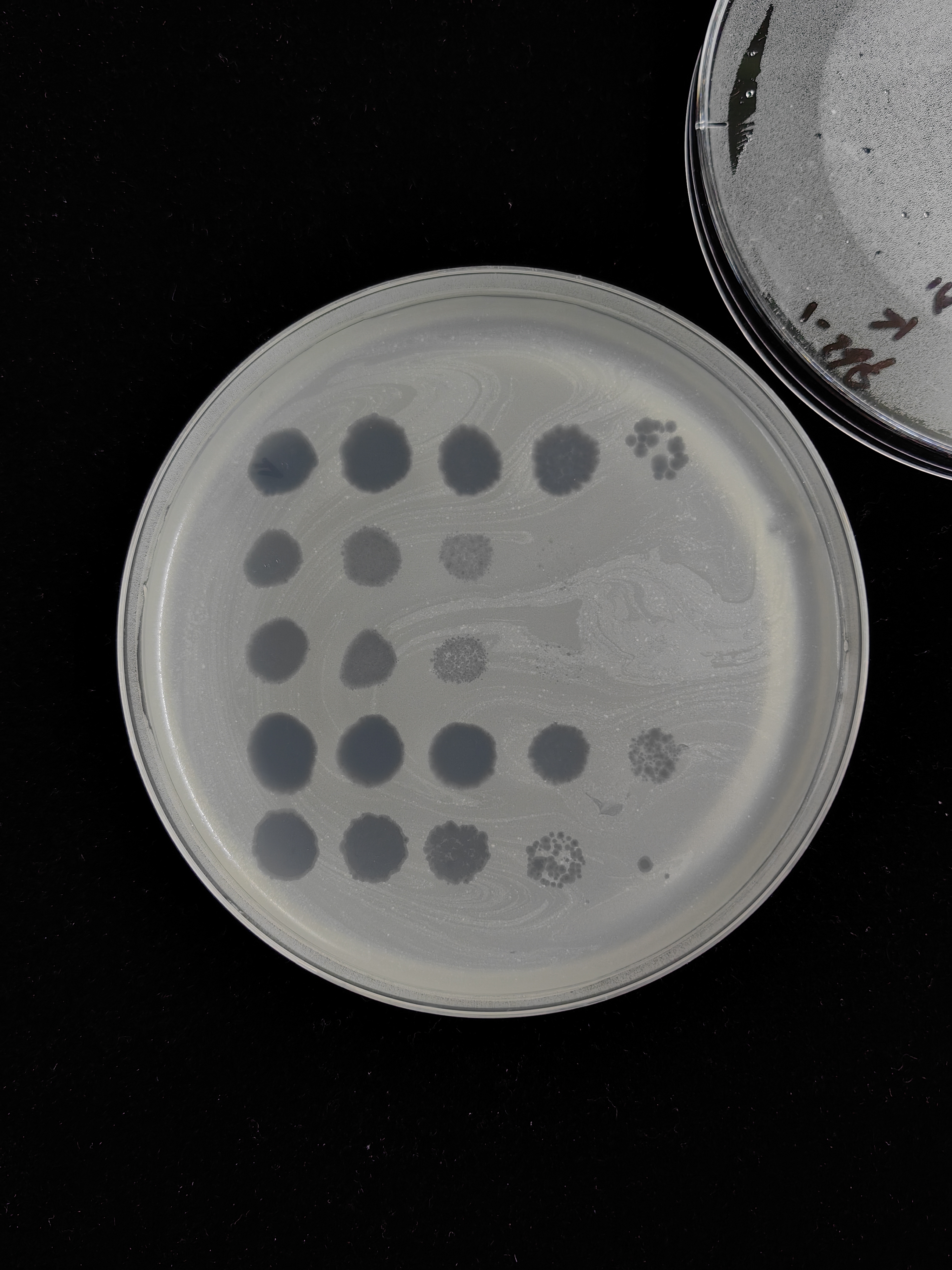

Supplement: Supplementary file 12 — Figure S7 Source Data [file 44319_2025_488_MOESM12_ESM.zip › Appendix Figure S7/S7A/pJR962-1 without ATc induction.tiff]

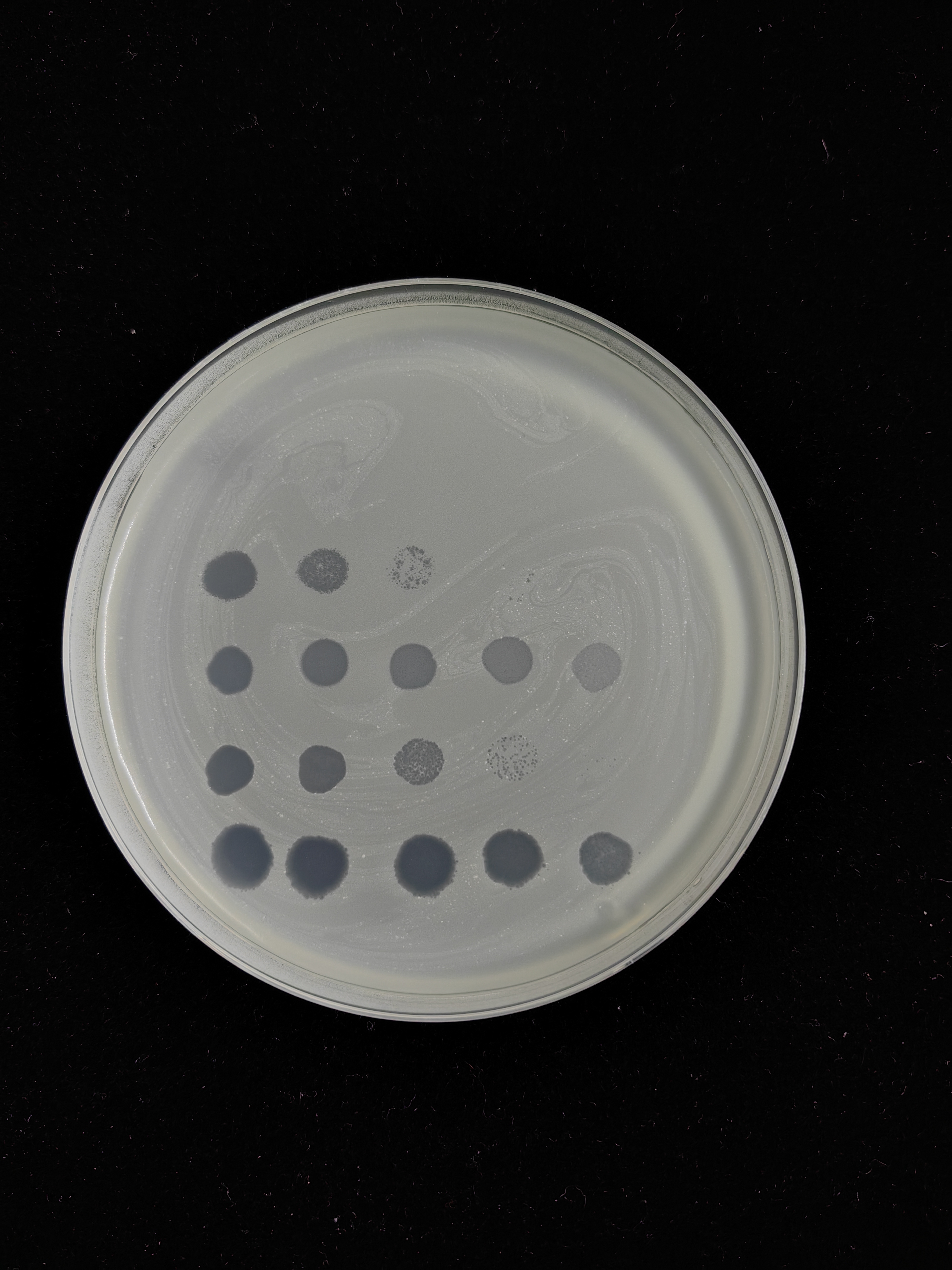

Supplement: Supplementary file 12 — Figure S7 Source Data [file 44319_2025_488_MOESM12_ESM.zip › Appendix Figure S7/S7A/pJR962-2 with ATc induction.tiff]

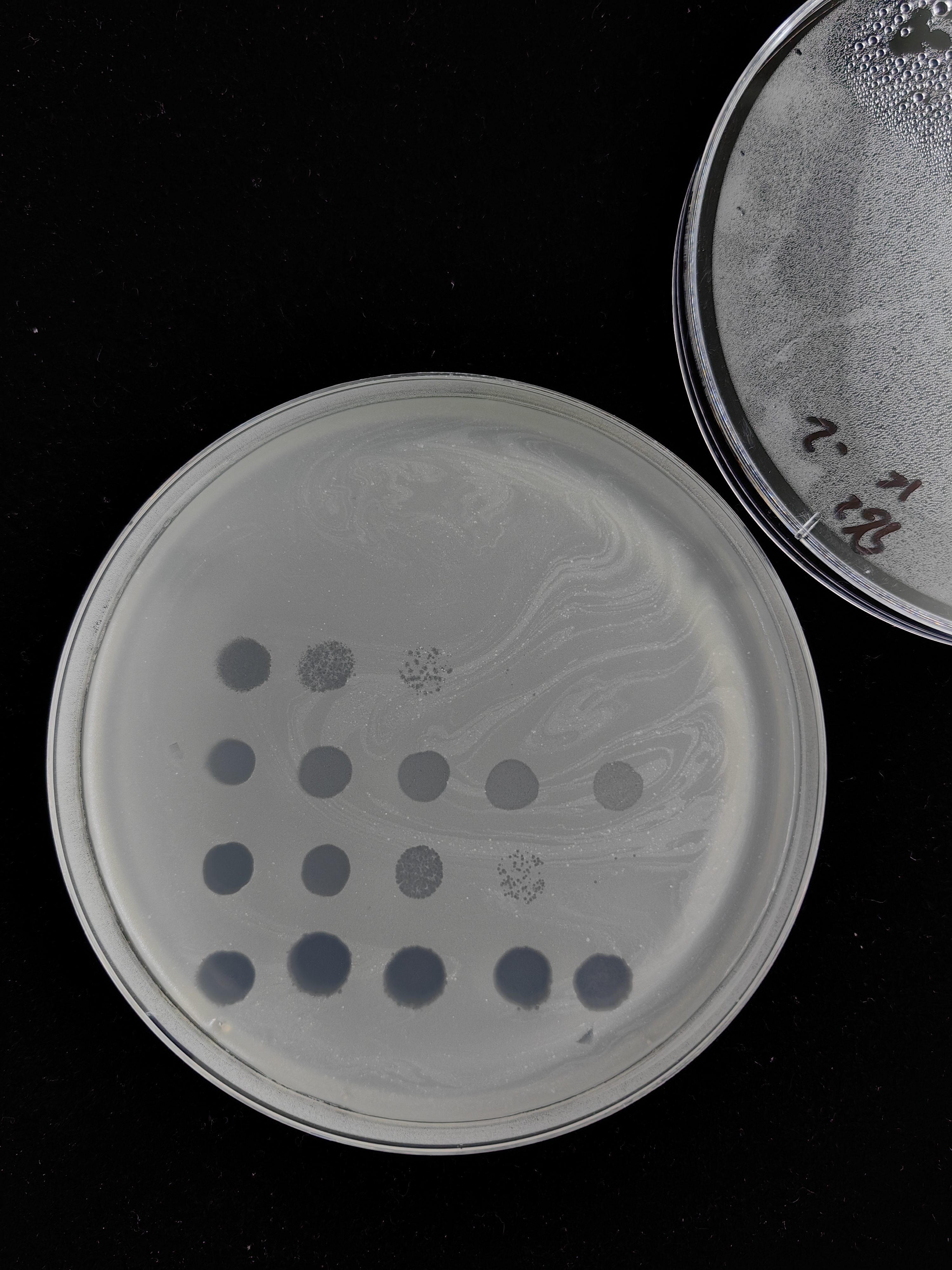

Supplement: Supplementary file 12 — Figure S7 Source Data [file 44319_2025_488_MOESM12_ESM.zip › Appendix Figure S7/S7A/pJR962-2 without ATc induction.tiff]

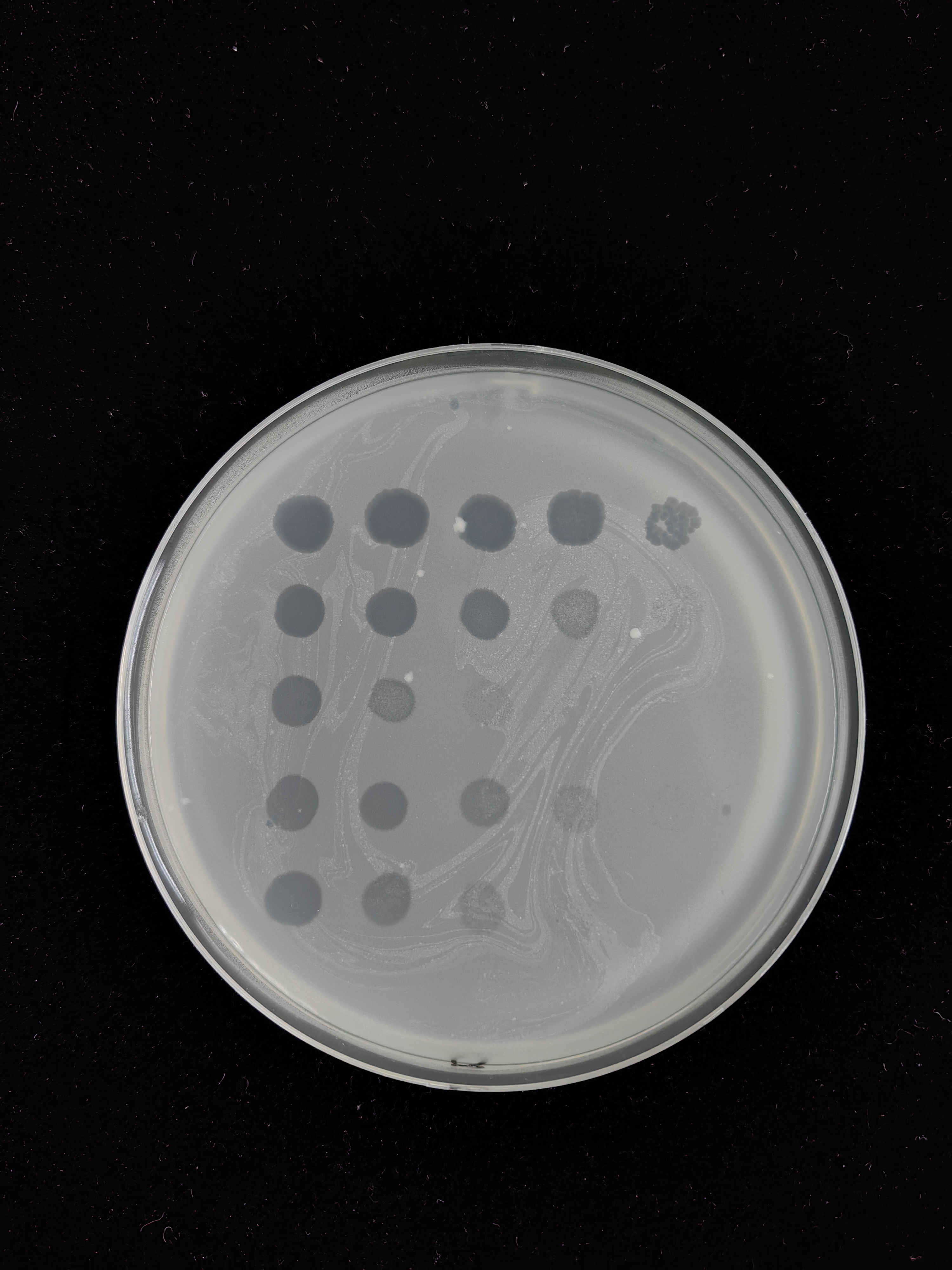

Supplement: Supplementary file 12 — Figure S7 Source Data [file 44319_2025_488_MOESM12_ESM.zip › Appendix Figure S7/S7A/pJR962-3 with ATc induction.tiff]

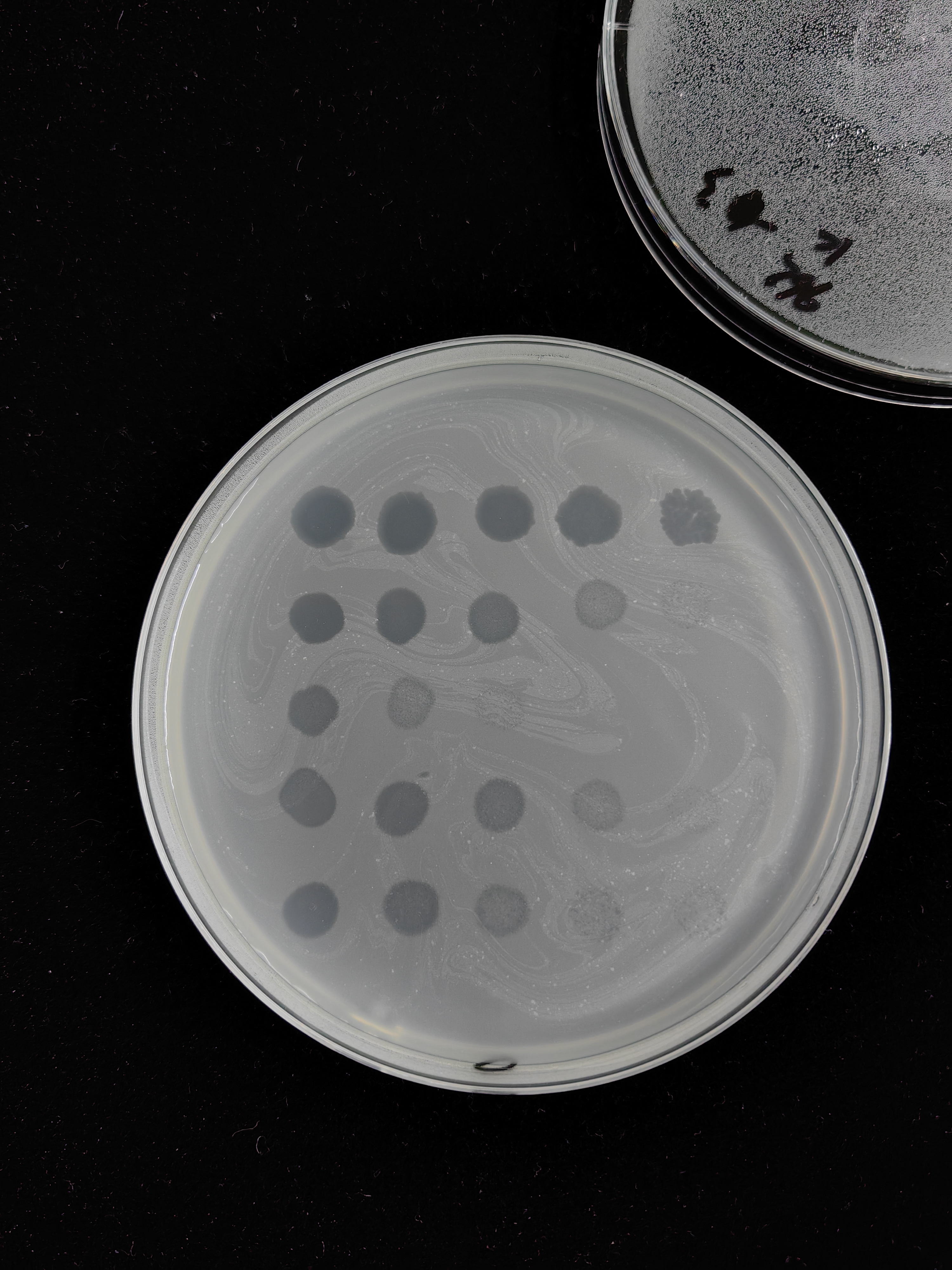

Supplement: Supplementary file 12 — Figure S7 Source Data [file 44319_2025_488_MOESM12_ESM.zip › Appendix Figure S7/S7A/pJR962-3 without ATc induction.tiff]

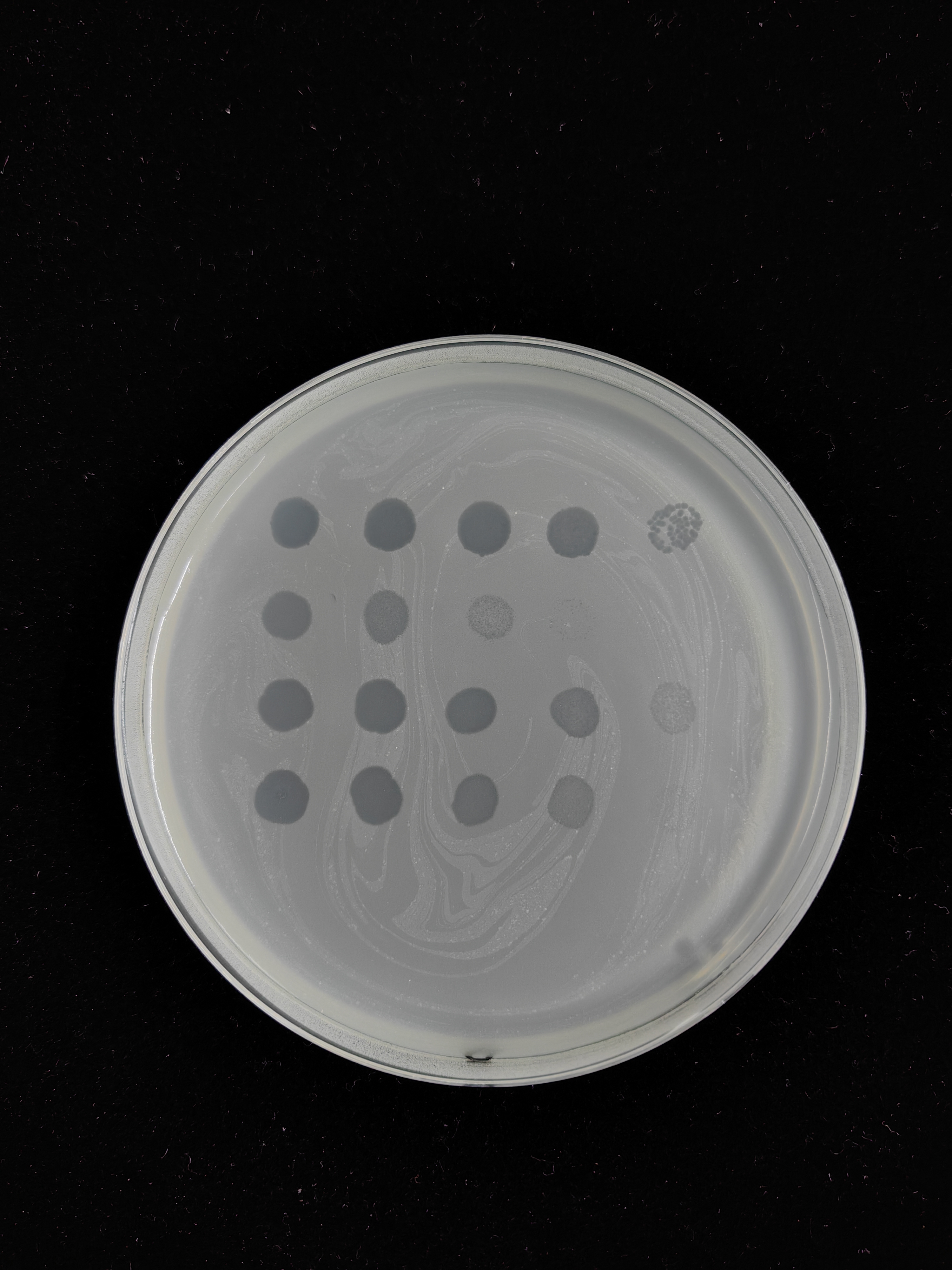

Supplement: Supplementary file 12 — Figure S7 Source Data [file 44319_2025_488_MOESM12_ESM.zip › Appendix Figure S7/S7A/pJR962-4 with ATc induction.tiff]

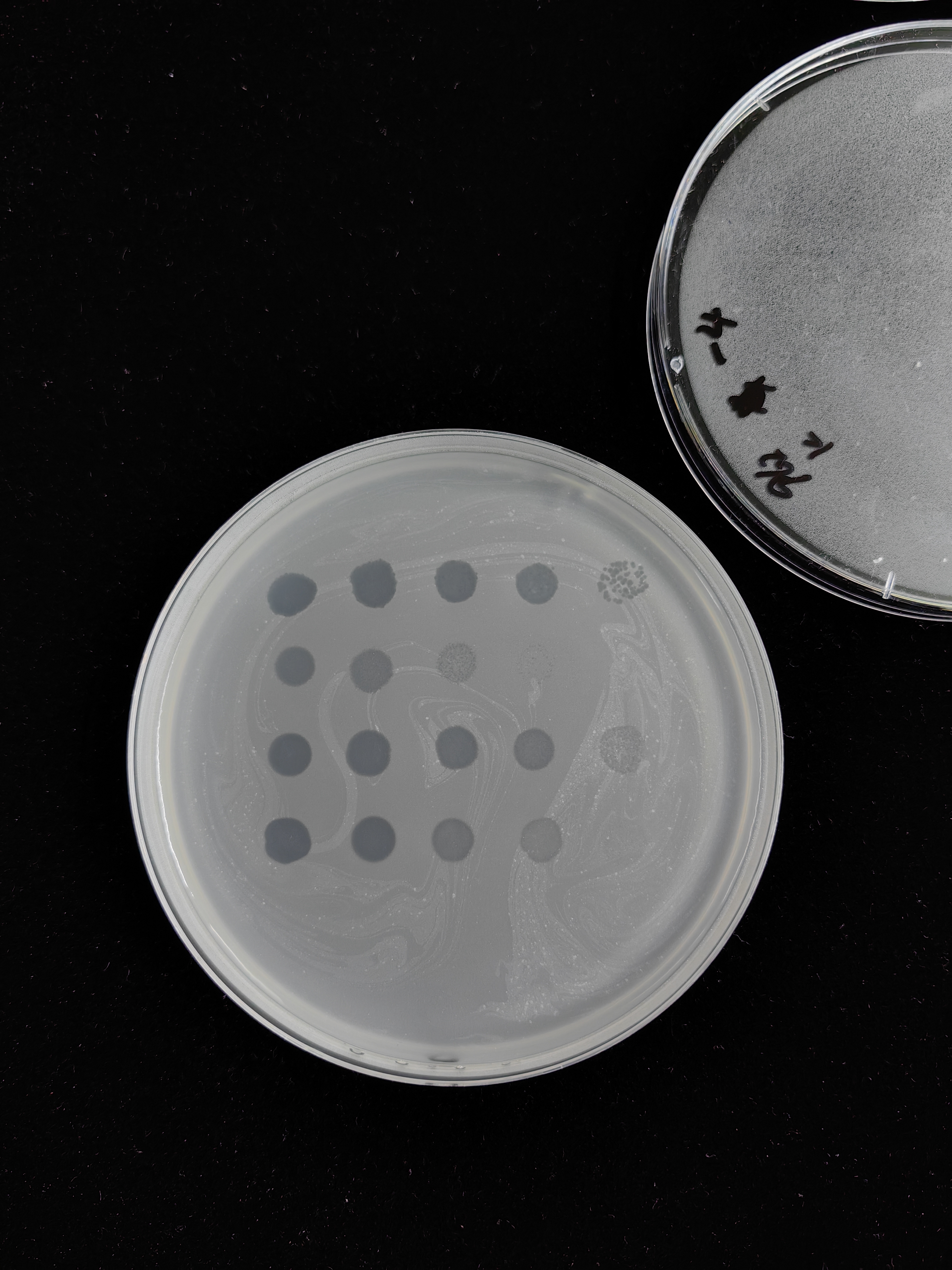

Supplement: Supplementary file 12 — Figure S7 Source Data [file 44319_2025_488_MOESM12_ESM.zip › Appendix Figure S7/S7A/pJR962-4 without ATc induction.tiff]

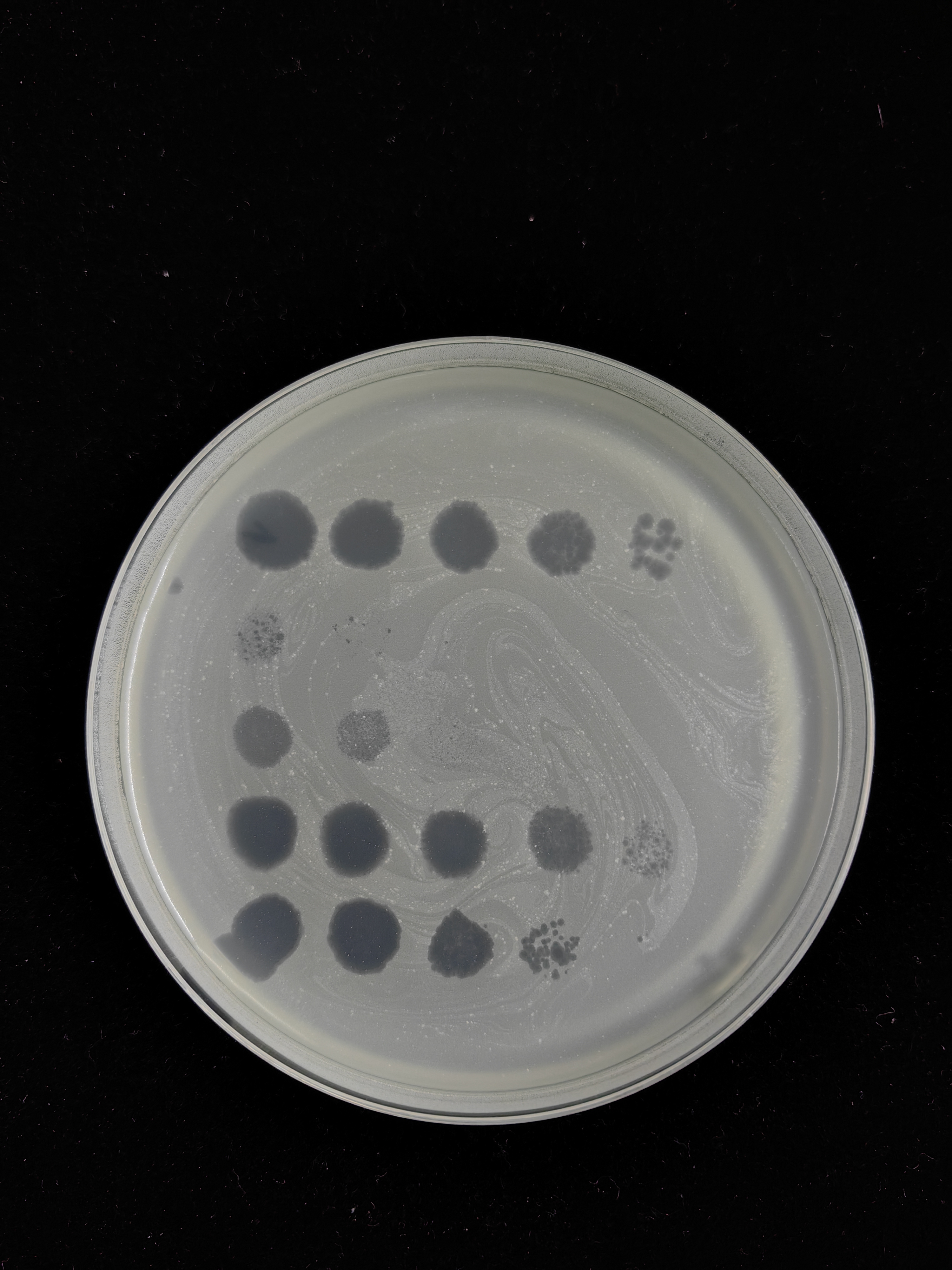

Supplement: Supplementary file 12 — Figure S7 Source Data [file 44319_2025_488_MOESM12_ESM.zip › Appendix Figure S7/S7A/pJR962-Mra IS6110-1 with ATc induction.tiff]

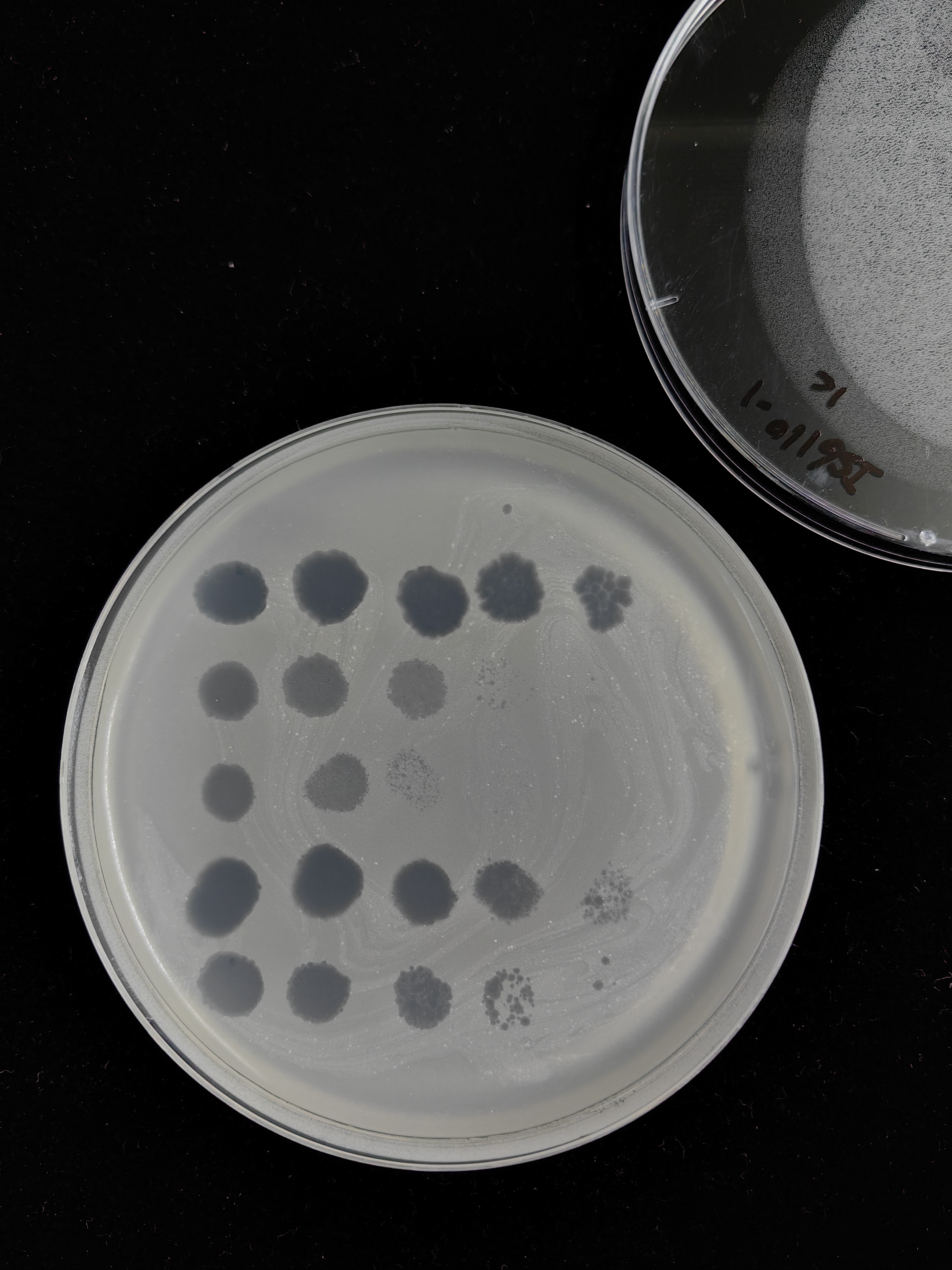

Supplement: Supplementary file 12 — Figure S7 Source Data [file 44319_2025_488_MOESM12_ESM.zip › Appendix Figure S7/S7A/pJR962-Mra IS6110-1 without ATc induction.tiff]

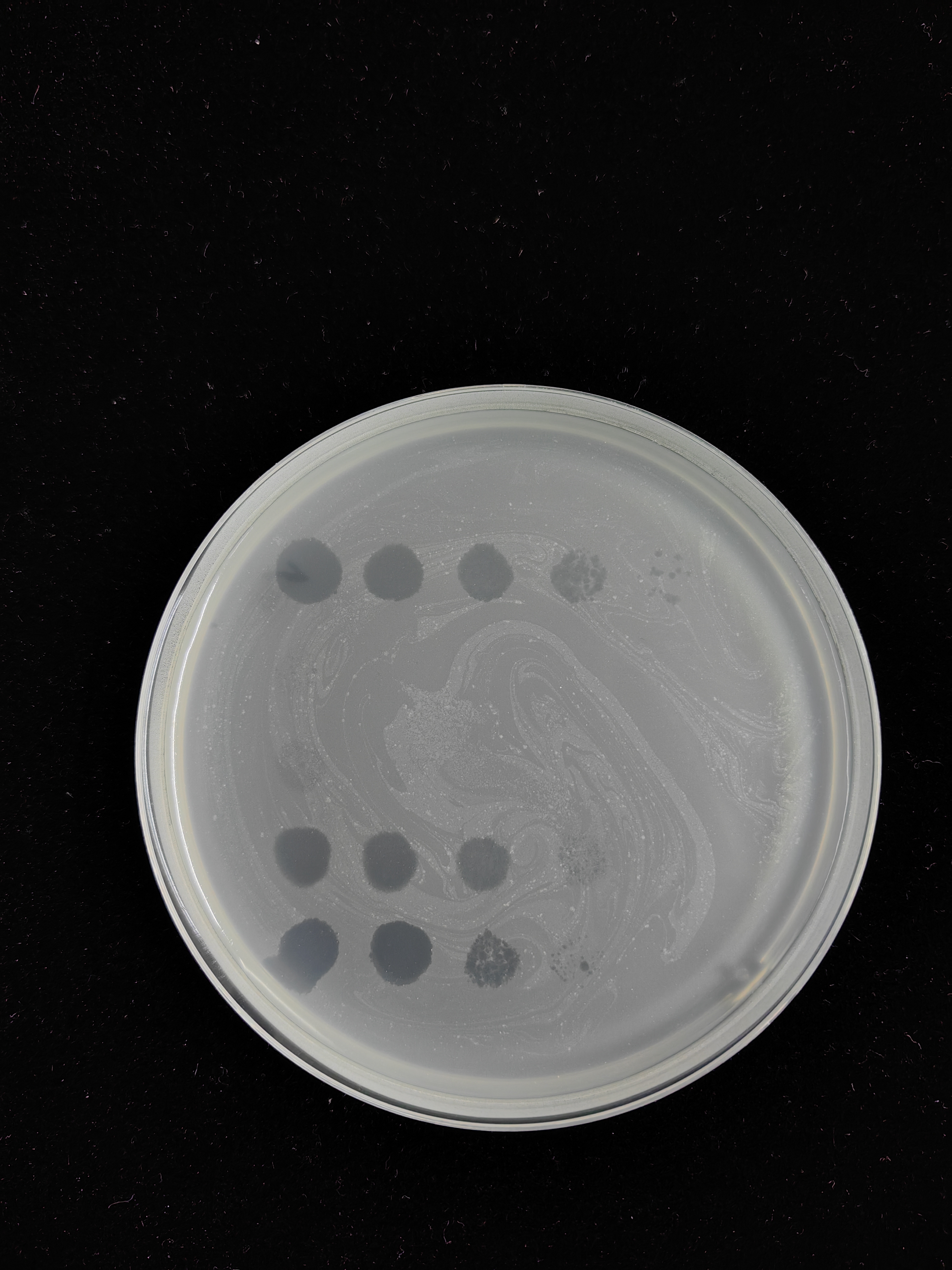

Supplement: Supplementary file 12 — Figure S7 Source Data [file 44319_2025_488_MOESM12_ESM.zip › Appendix Figure S7/S7A/pJR962-Mra IS6110-2 with ATc induction.tiff]

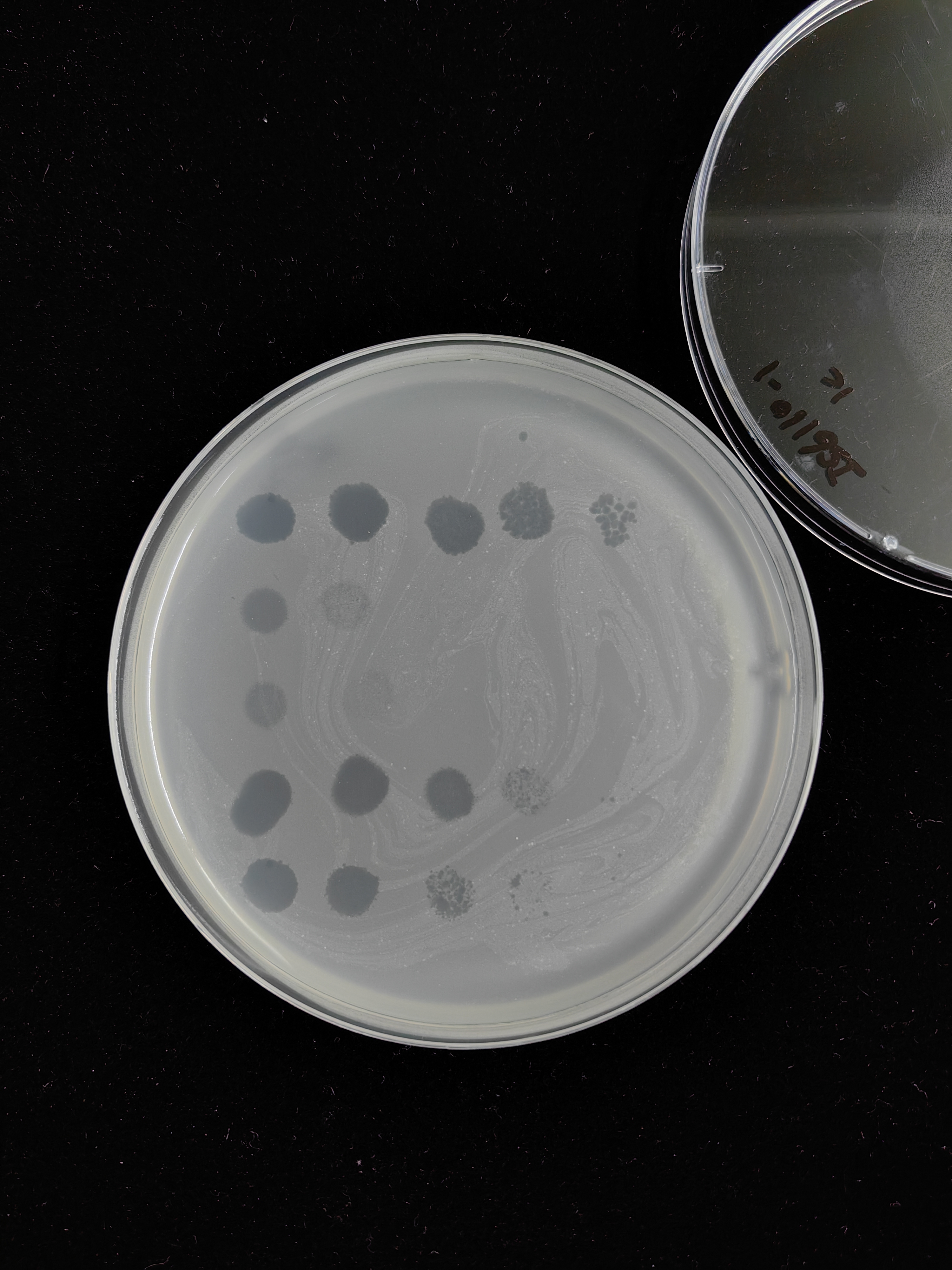

Supplement: Supplementary file 12 — Figure S7 Source Data [file 44319_2025_488_MOESM12_ESM.zip › Appendix Figure S7/S7A/pJR962-Mra IS6110-2 without ATc induction.tiff]

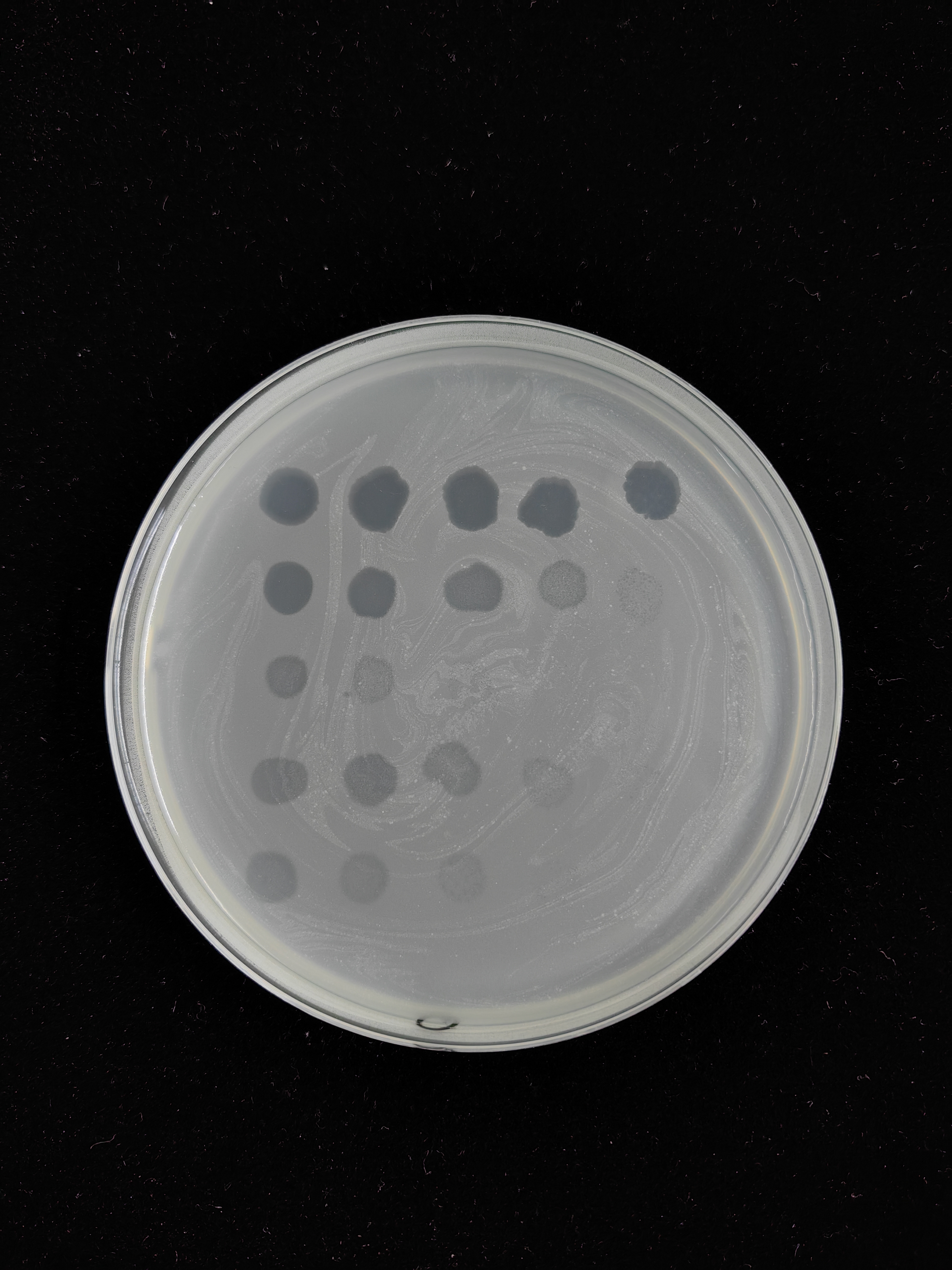

Supplement: Supplementary file 12 — Figure S7 Source Data [file 44319_2025_488_MOESM12_ESM.zip › Appendix Figure S7/S7A/pJR962-Mra IS6110-3 with ATc induction.tiff]

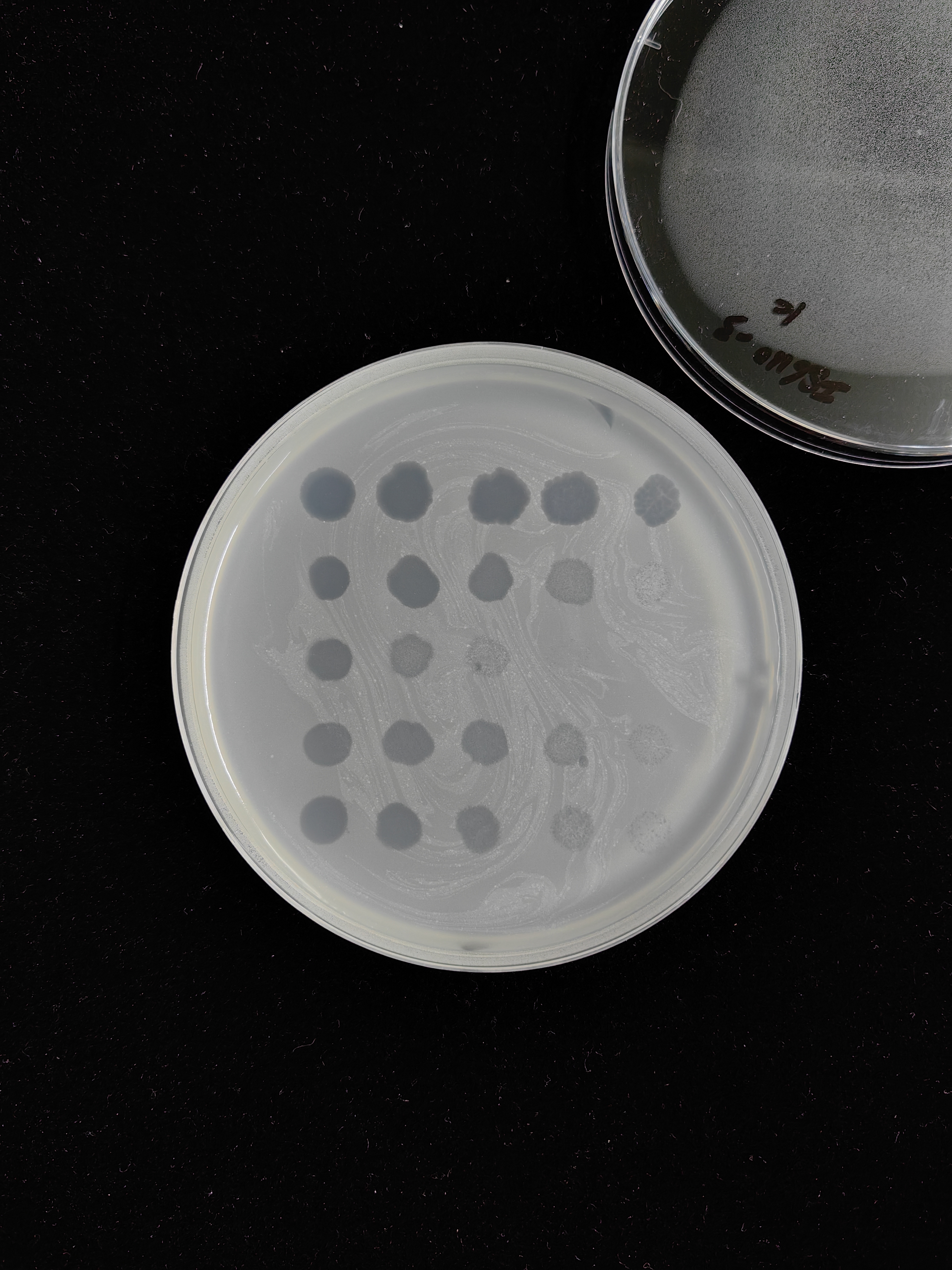

Supplement: Supplementary file 12 — Figure S7 Source Data [file 44319_2025_488_MOESM12_ESM.zip › Appendix Figure S7/S7A/pJR962-Mra IS6110-3 without ATc induction.tiff]

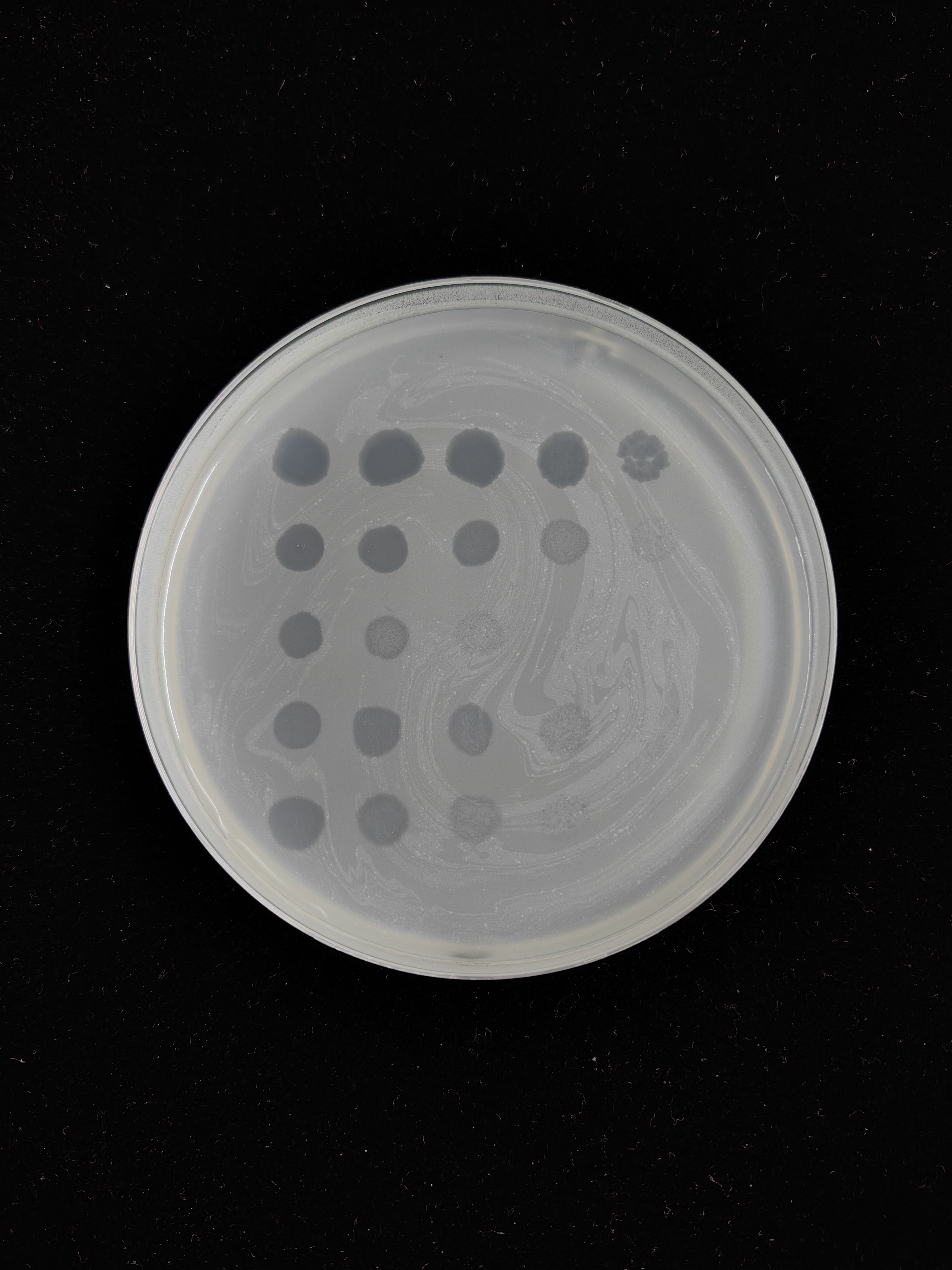

Supplement: Supplementary file 12 — Figure S7 Source Data [file 44319_2025_488_MOESM12_ESM.zip › Appendix Figure S7/S7A/pJR962-Mra_1649-1 with ATc induction.tiff]

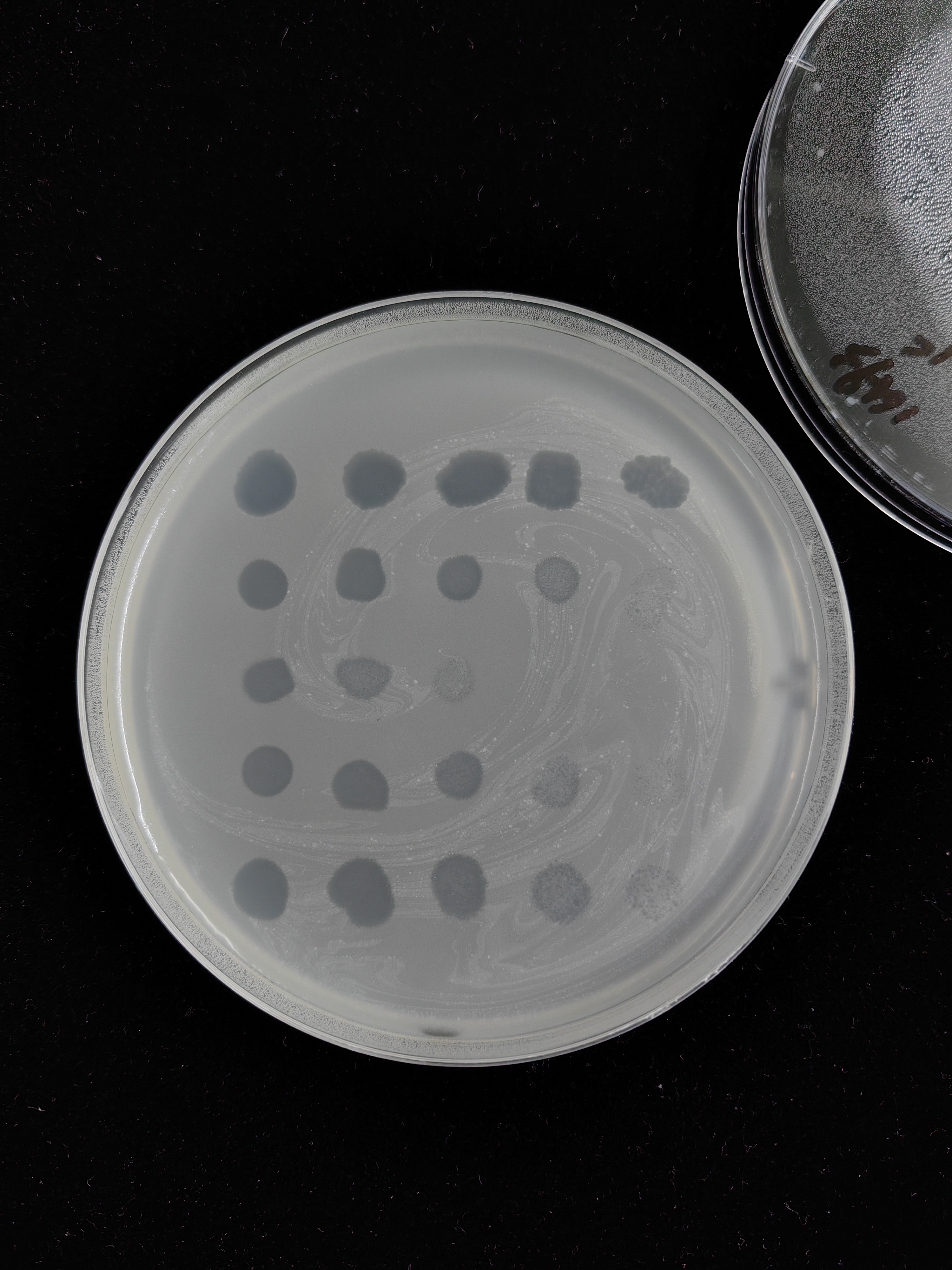

Supplement: Supplementary file 12 — Figure S7 Source Data [file 44319_2025_488_MOESM12_ESM.zip › Appendix Figure S7/S7A/pJR962-Mra_1649-1 without ATc induction.tiff]

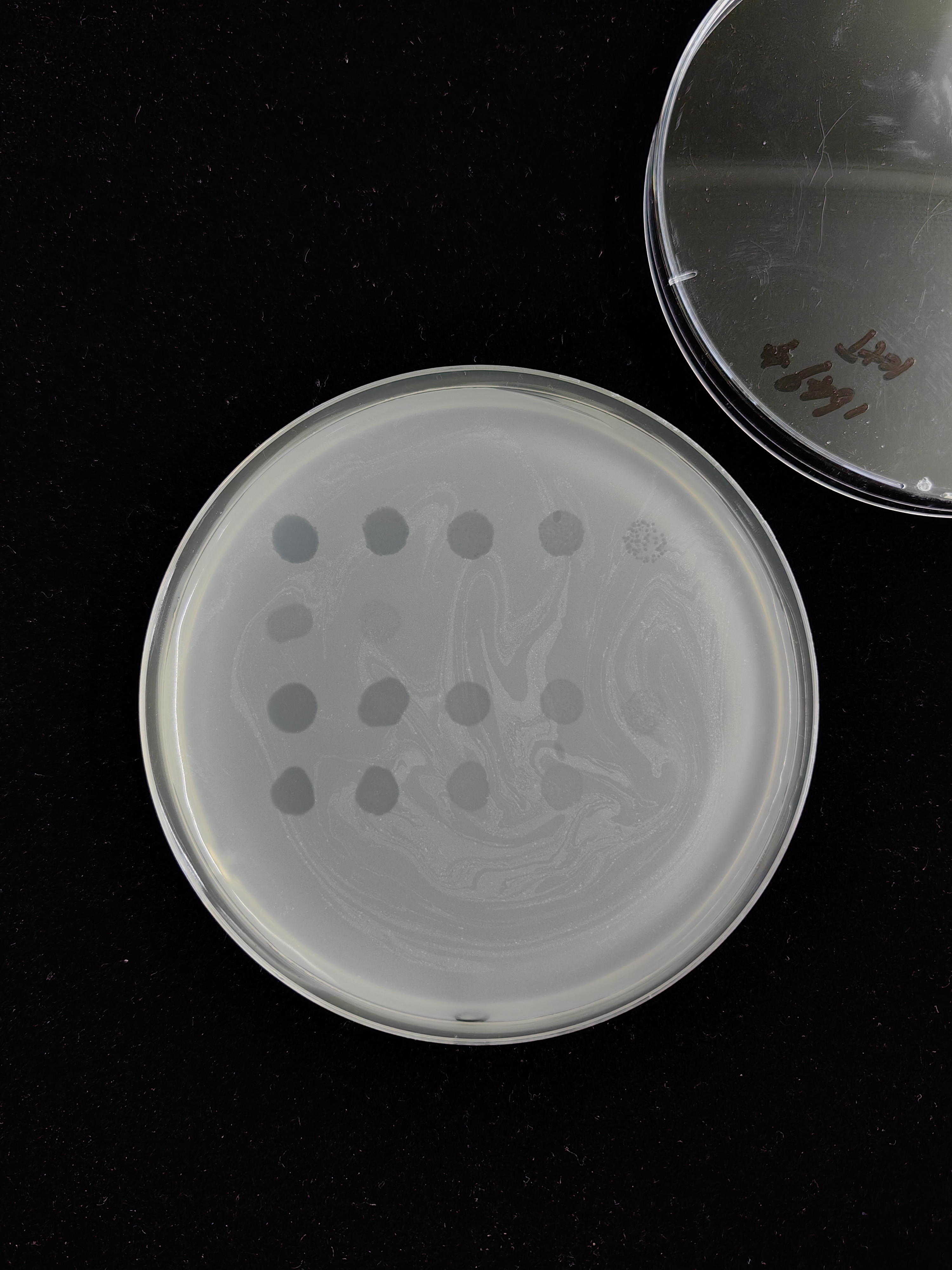

Supplement: Supplementary file 12 — Figure S7 Source Data [file 44319_2025_488_MOESM12_ESM.zip › Appendix Figure S7/S7A/pJR962-Mra_1649-2 with ATc induction.tiff]

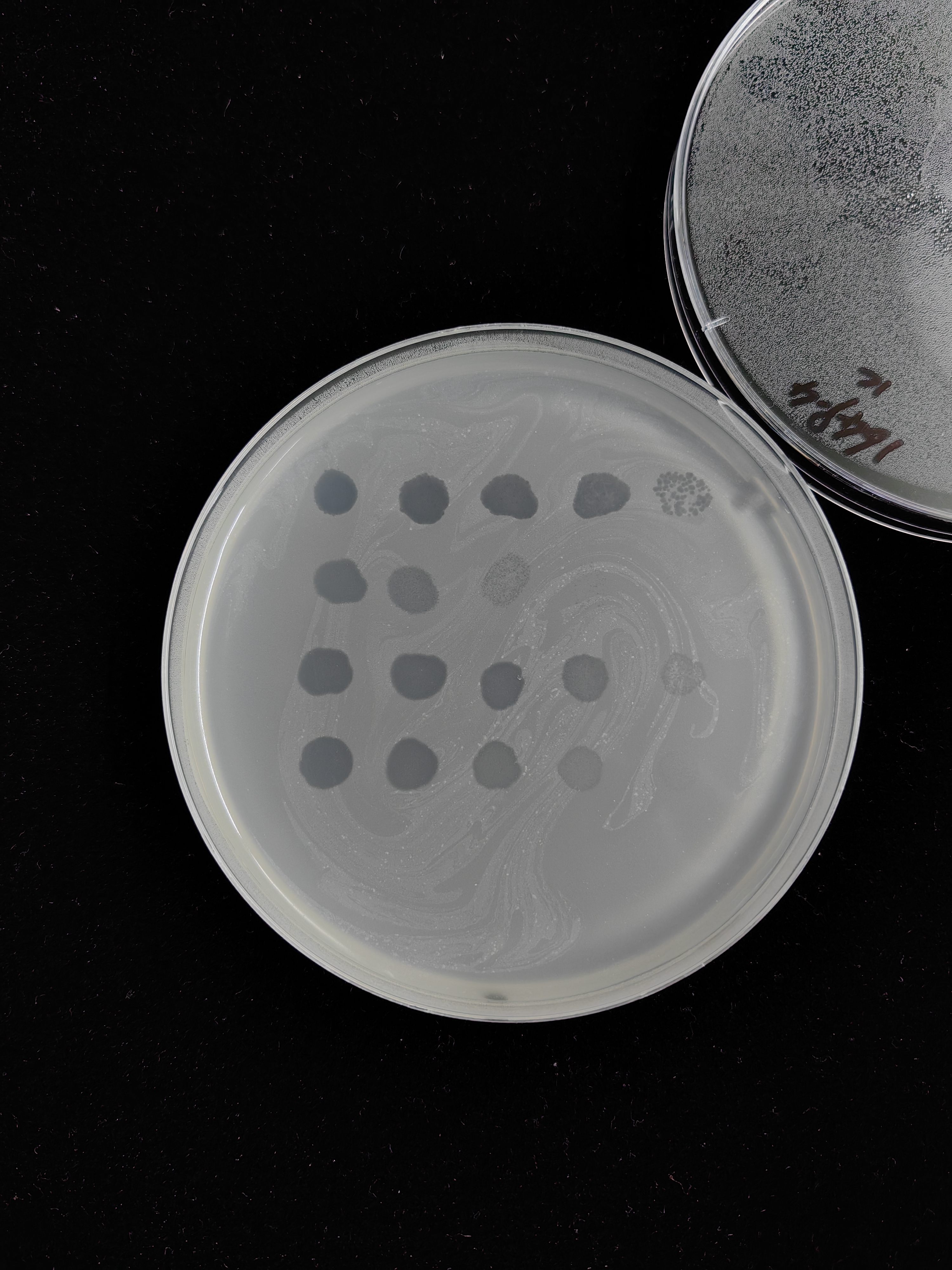

Supplement: Supplementary file 12 — Figure S7 Source Data [file 44319_2025_488_MOESM12_ESM.zip › Appendix Figure S7/S7A/pJR962-Mra_1649-2 without ATc induction.tiff]

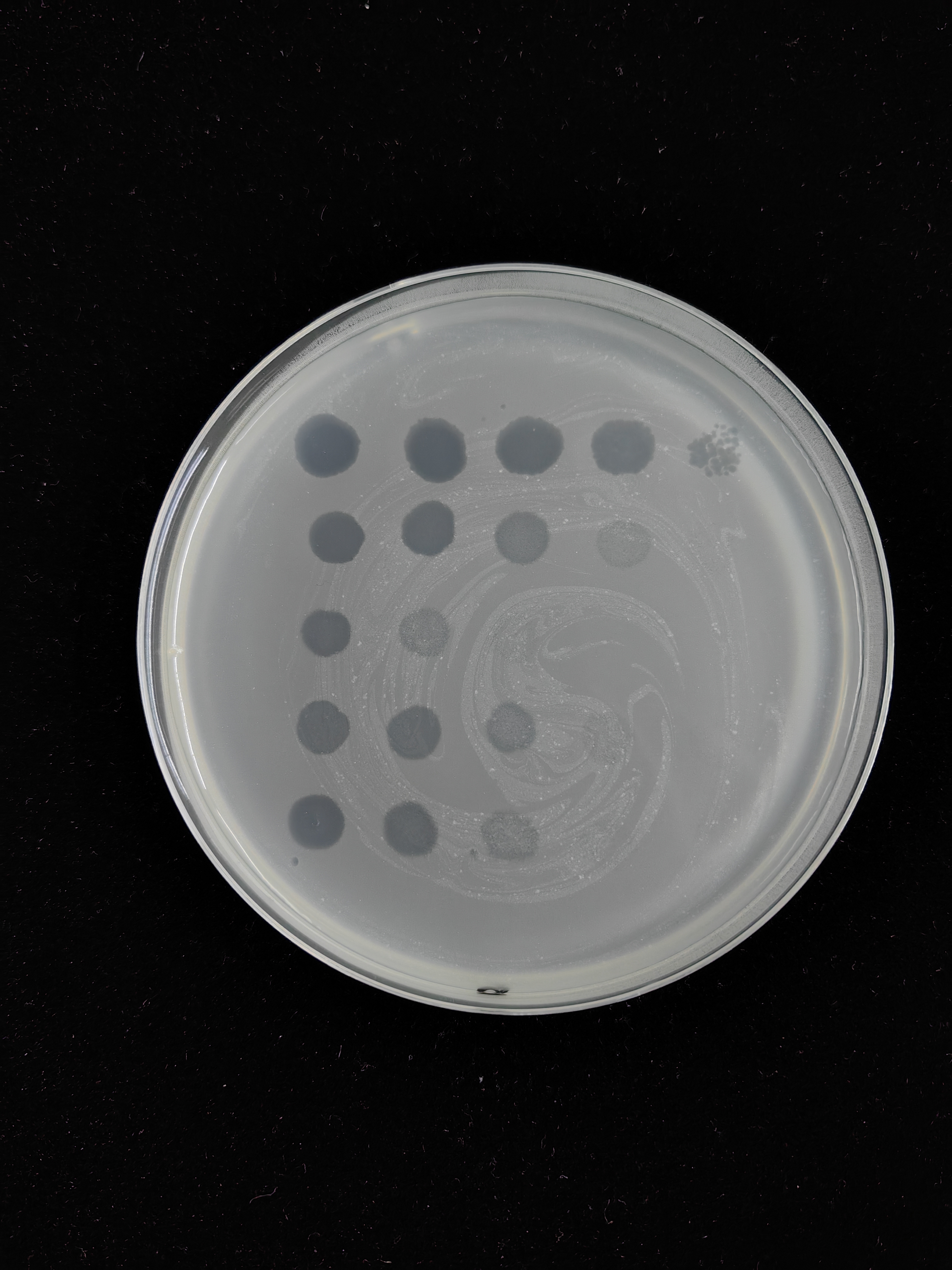

Supplement: Supplementary file 12 — Figure S7 Source Data [file 44319_2025_488_MOESM12_ESM.zip › Appendix Figure S7/S7A/pJR962-Mra_1940-1 with ATc induction.tiff]

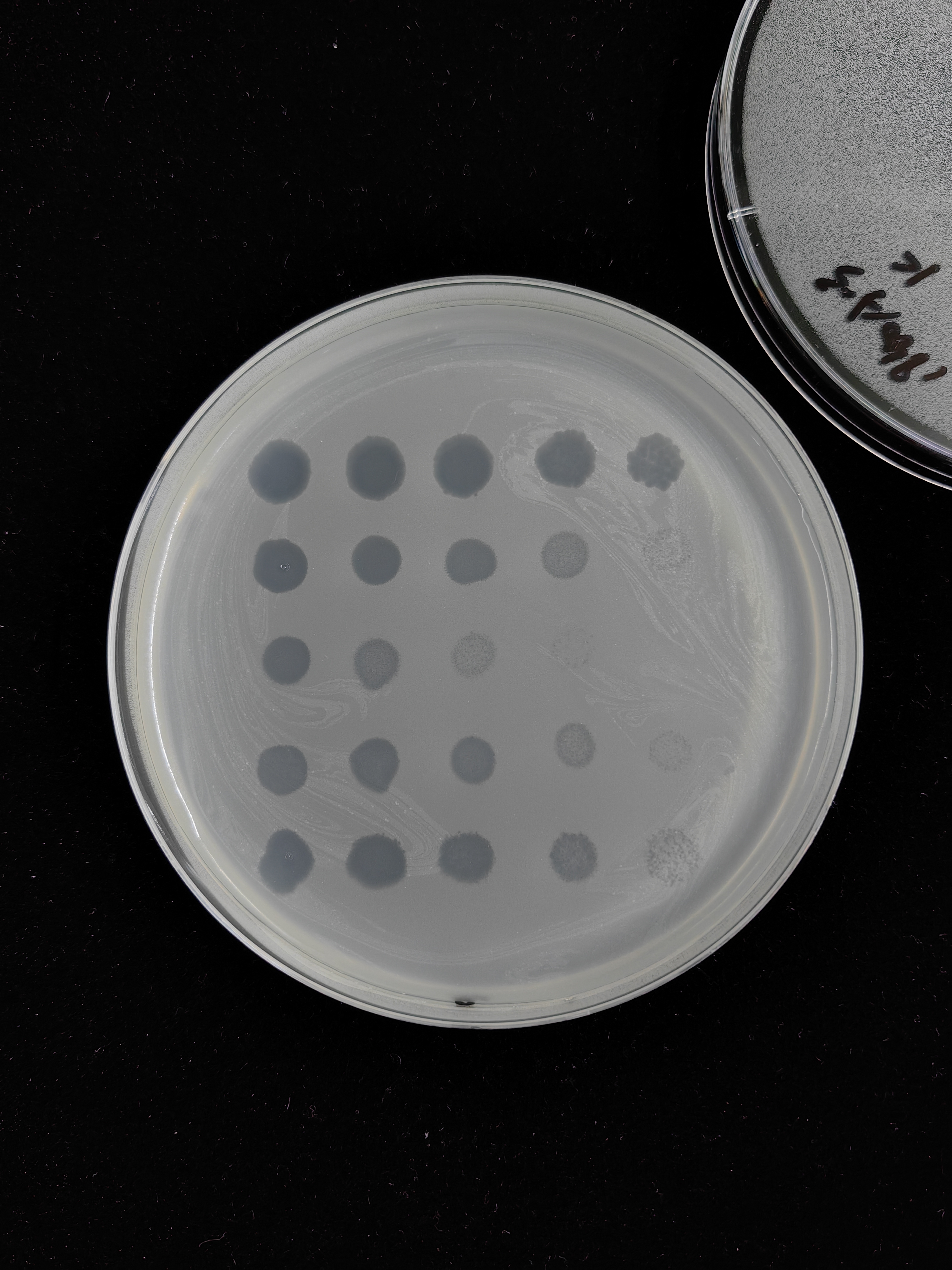

Supplement: Supplementary file 12 — Figure S7 Source Data [file 44319_2025_488_MOESM12_ESM.zip › Appendix Figure S7/S7A/pJR962-Mra_1940A-1 without ATc induction.tiff]

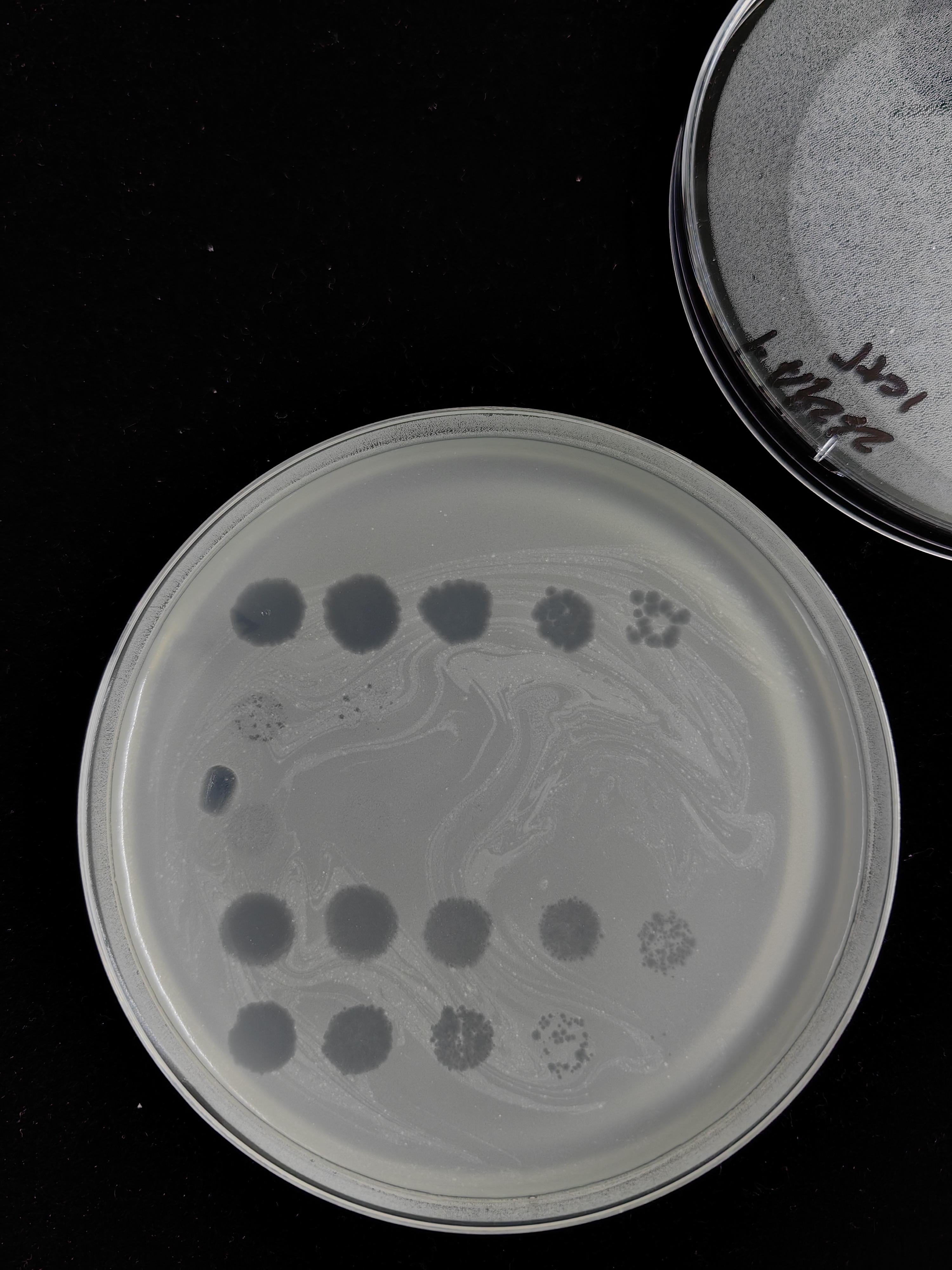

Supplement: Supplementary file 12 — Figure S7 Source Data [file 44319_2025_488_MOESM12_ESM.zip › Appendix Figure S7/S7A/pJR962-Mra_2329A-1 with ATc induction.tiff]

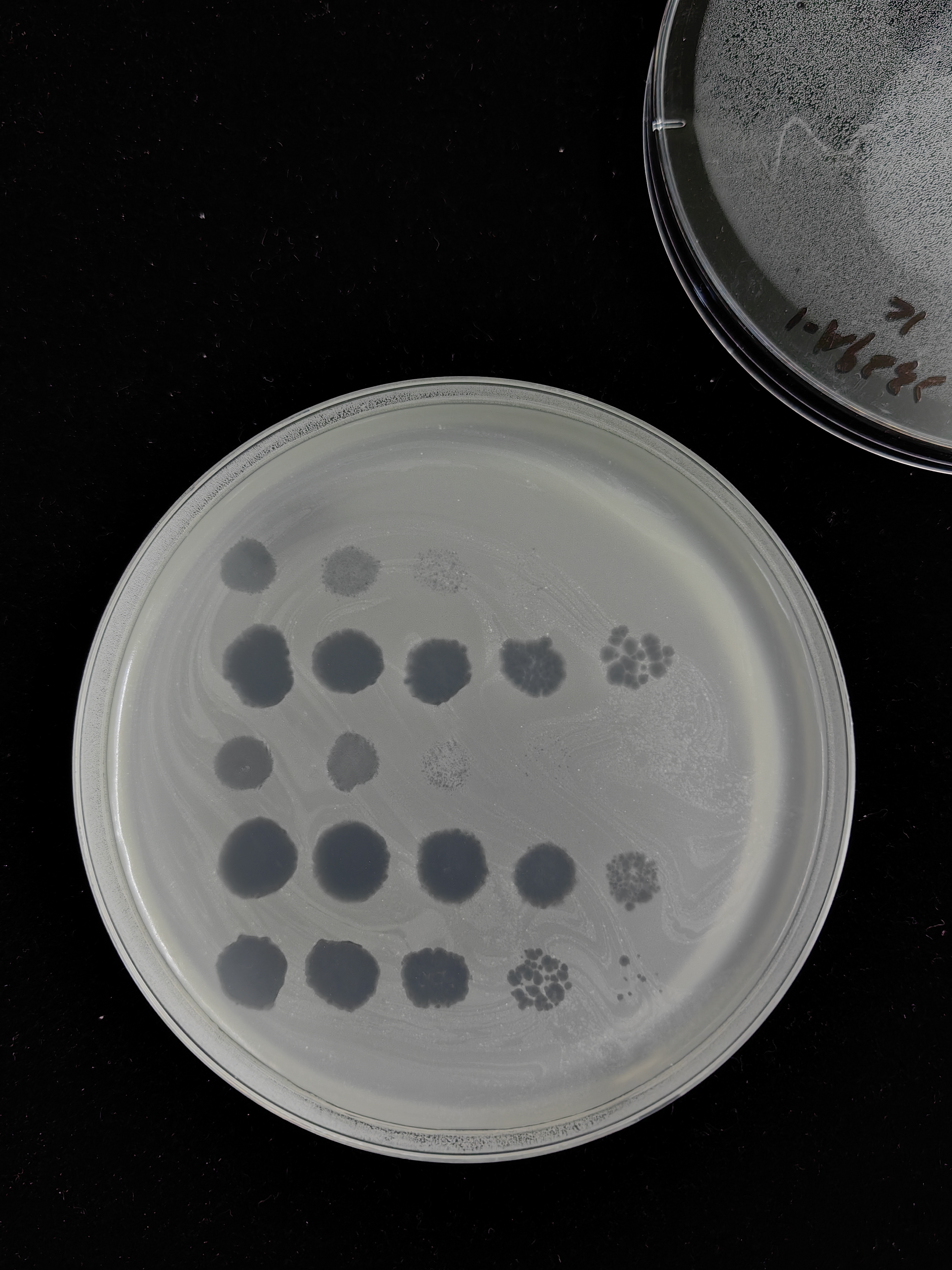

Supplement: Supplementary file 12 — Figure S7 Source Data [file 44319_2025_488_MOESM12_ESM.zip › Appendix Figure S7/S7A/pJR962-Mra_2329A-1 without ATc induction.tiff]

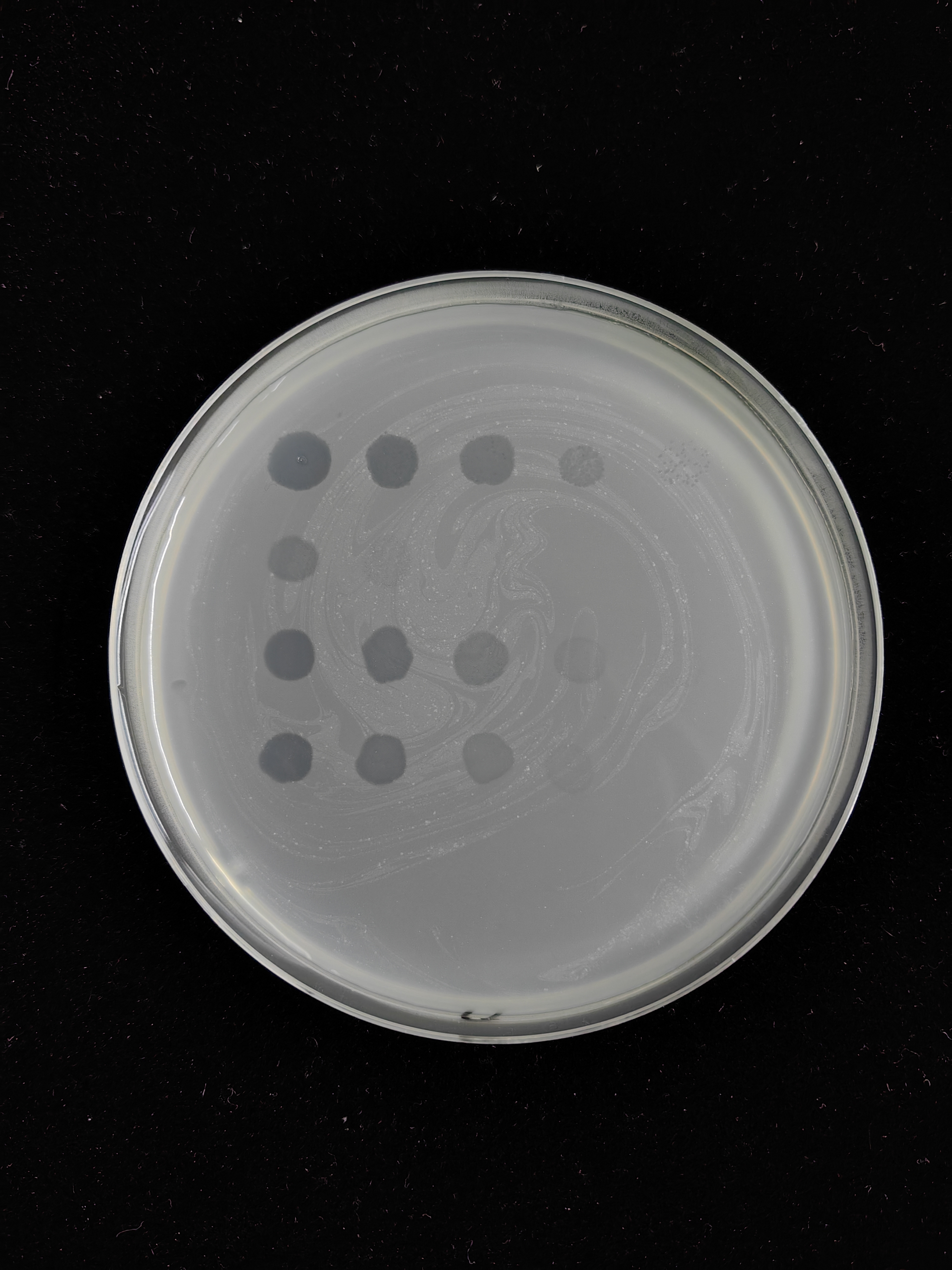

Supplement: Supplementary file 12 — Figure S7 Source Data [file 44319_2025_488_MOESM12_ESM.zip › Appendix Figure S7/S7A/pJR962-Mra_2329A-2 with ATc induction.tiff]

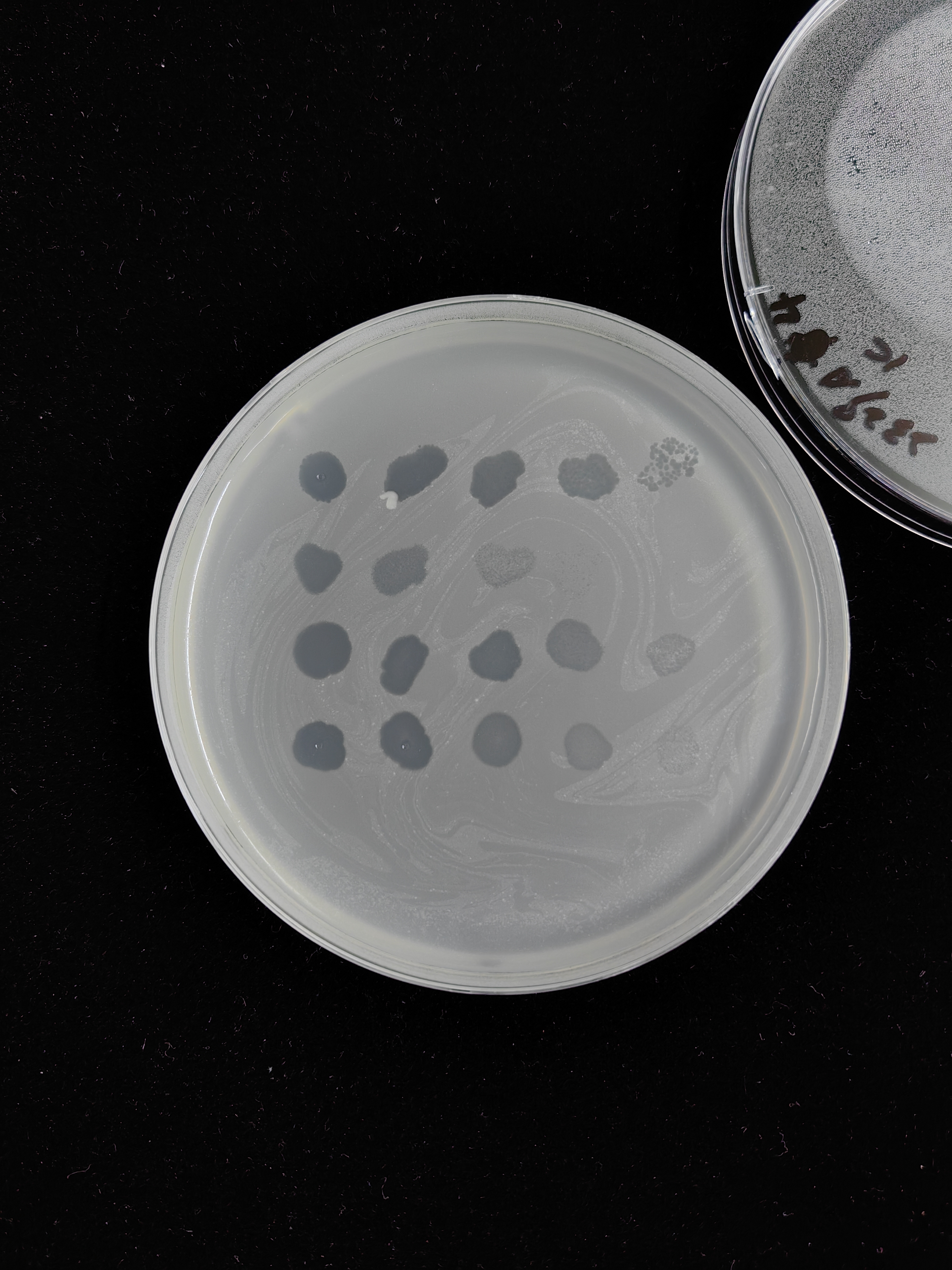

Supplement: Supplementary file 12 — Figure S7 Source Data [file 44319_2025_488_MOESM12_ESM.zip › Appendix Figure S7/S7A/pJR962-Mra_2329A-2 without ATc induction.tiff]

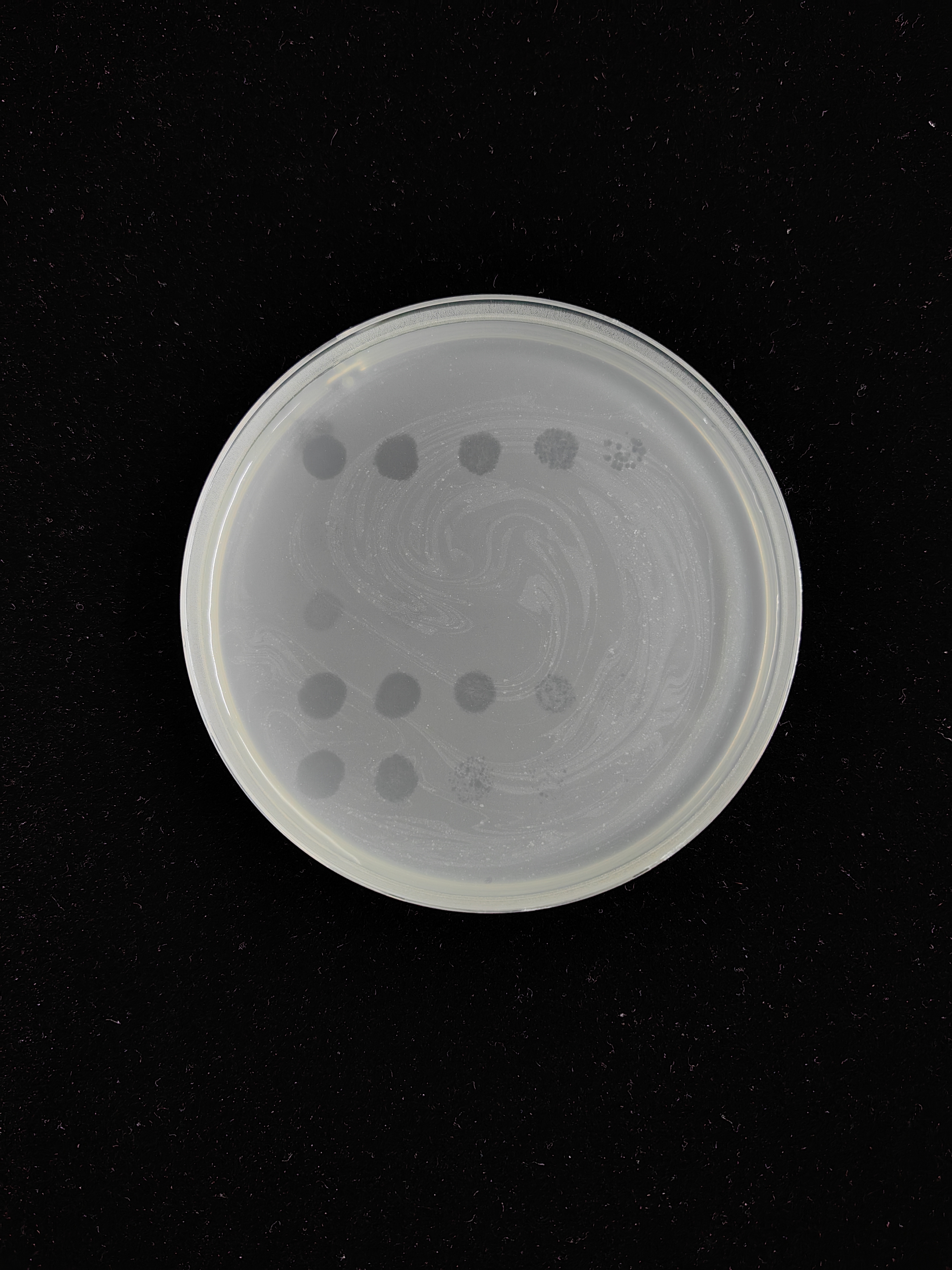

Supplement: Supplementary file 12 — Figure S7 Source Data [file 44319_2025_488_MOESM12_ESM.zip › Appendix Figure S7/S7A/pJR962-Mra_3122-1 with ATc induction.tiff]

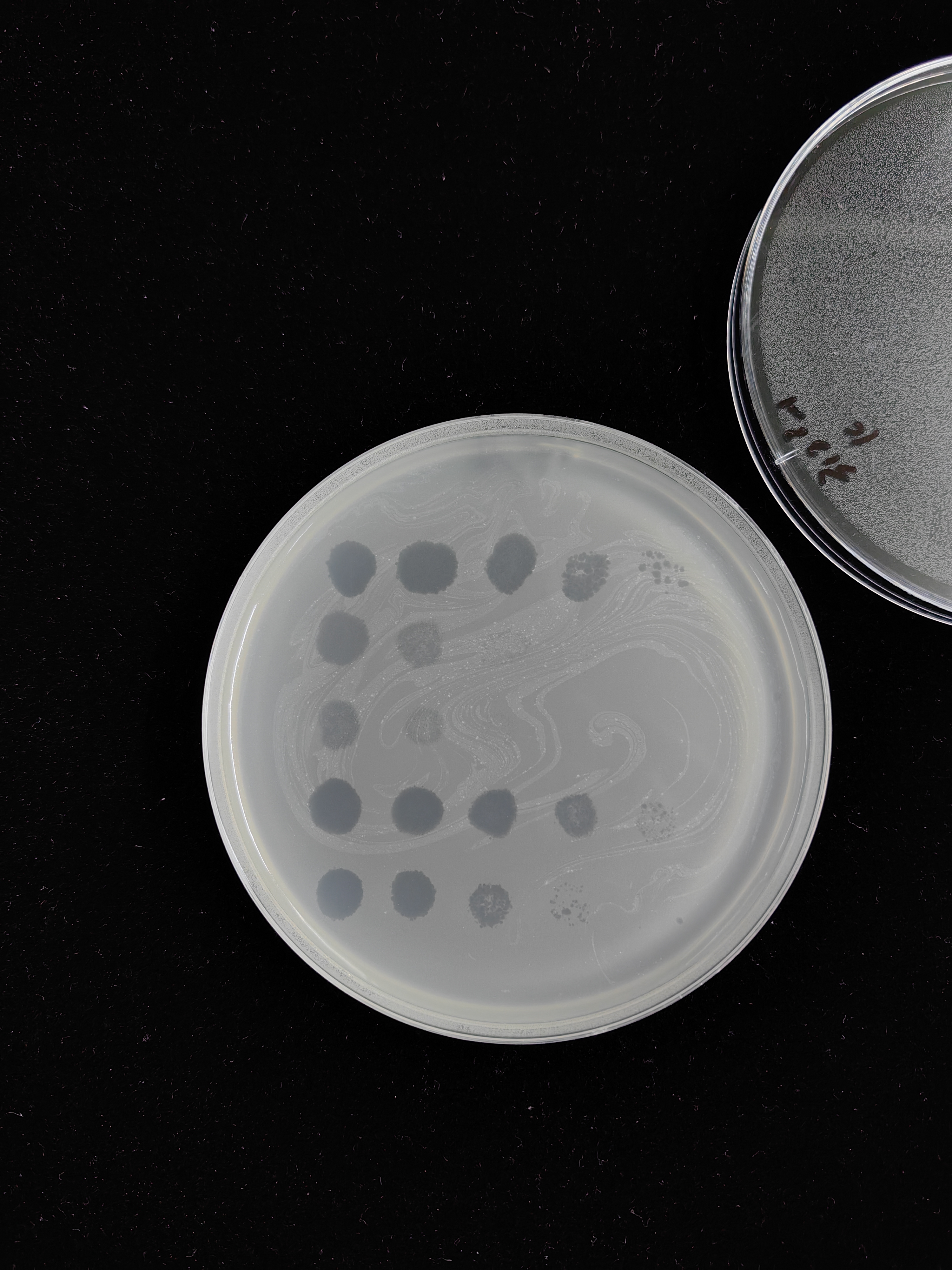

Supplement: Supplementary file 12 — Figure S7 Source Data [file 44319_2025_488_MOESM12_ESM.zip › Appendix Figure S7/S7A/pJR962-Mra_3122-1 without ATc induction.tiff]

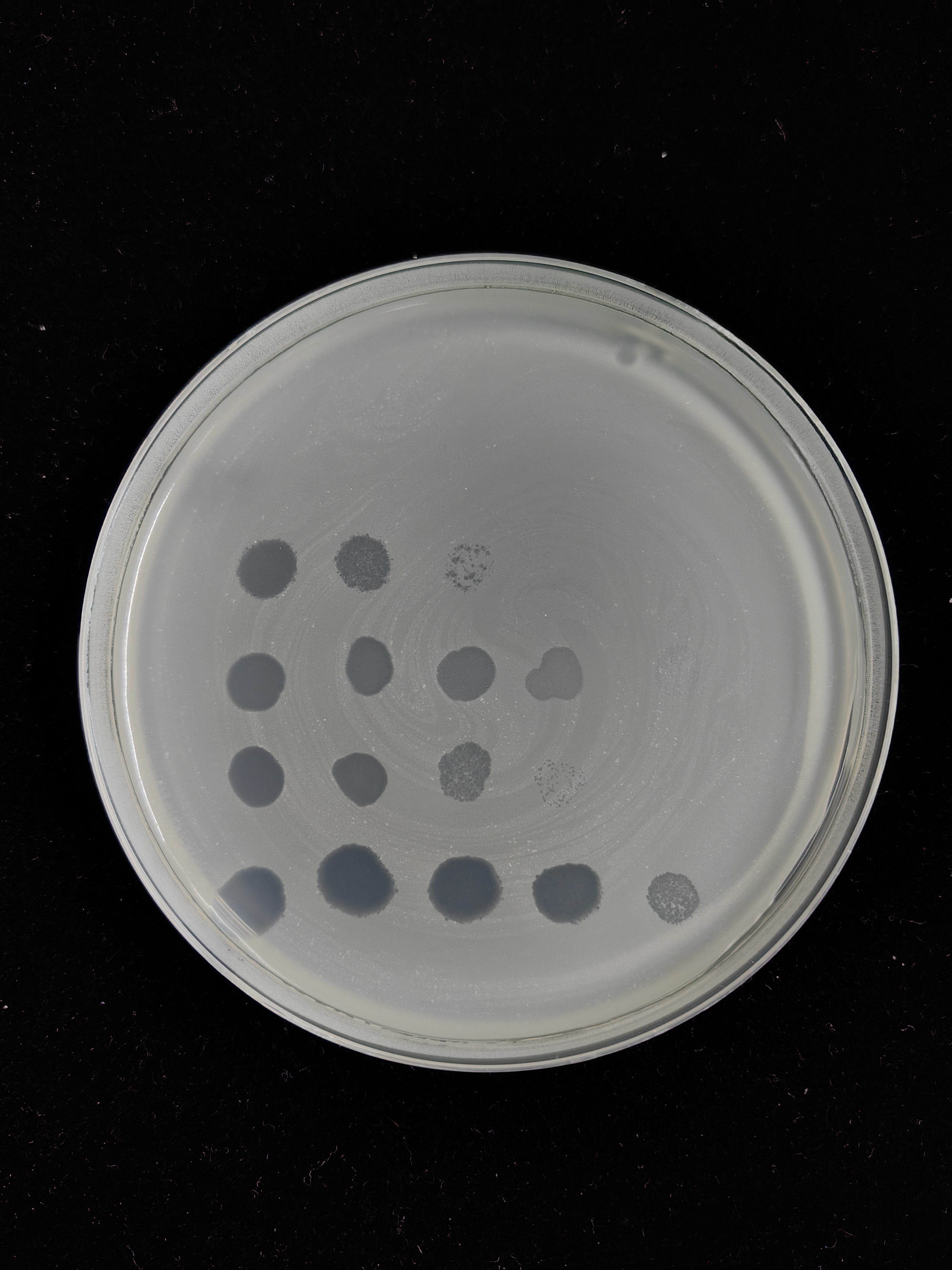

Supplement: Supplementary file 12 — Figure S7 Source Data [file 44319_2025_488_MOESM12_ESM.zip › Appendix Figure S7/S7A/pJR962-Mra_3122-2 with ATc induction.tiff]

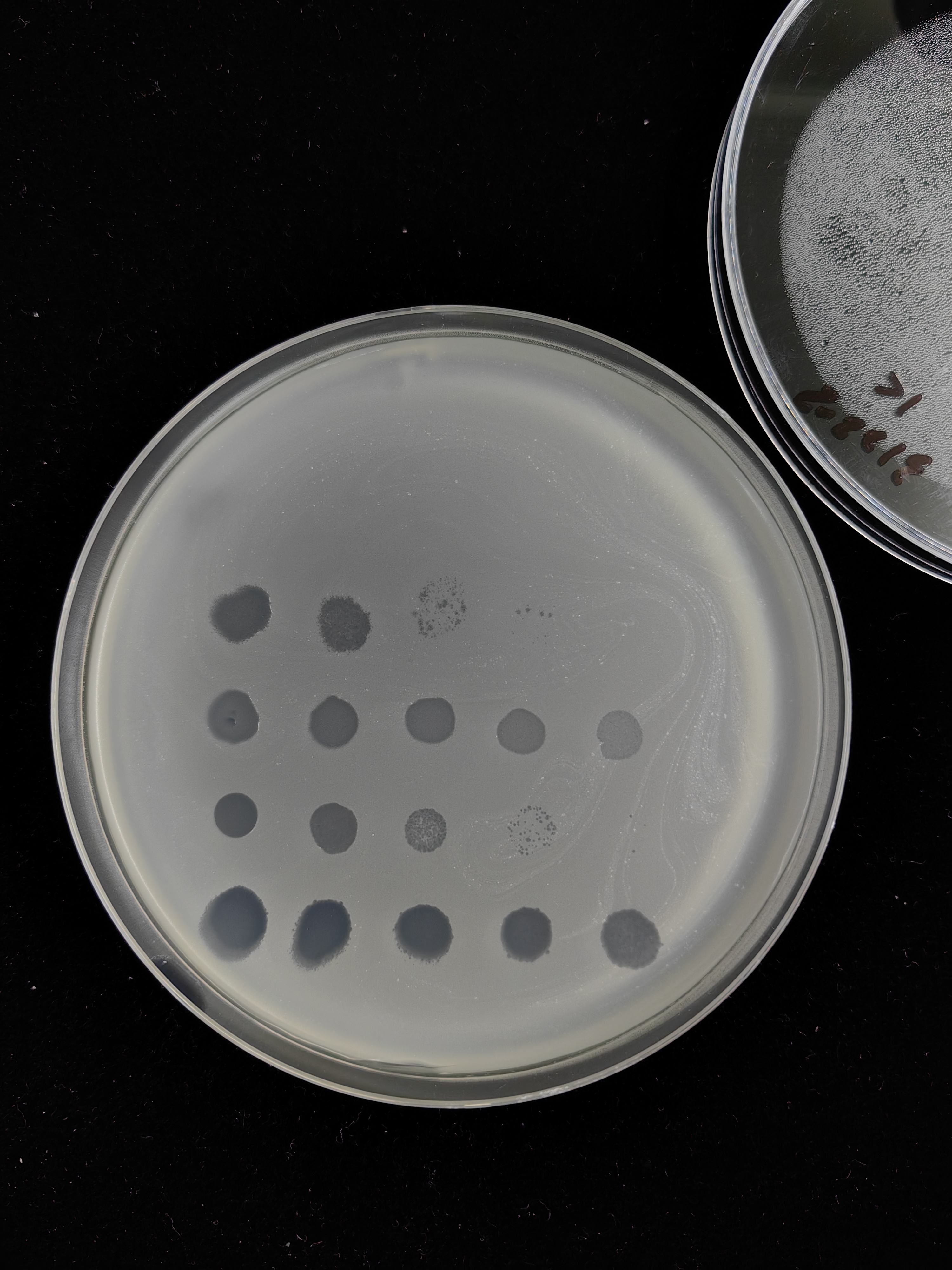

Supplement: Supplementary file 12 — Figure S7 Source Data [file 44319_2025_488_MOESM12_ESM.zip › Appendix Figure S7/S7A/pJR962-Mra_3122-2 without ATc induction.tiff]

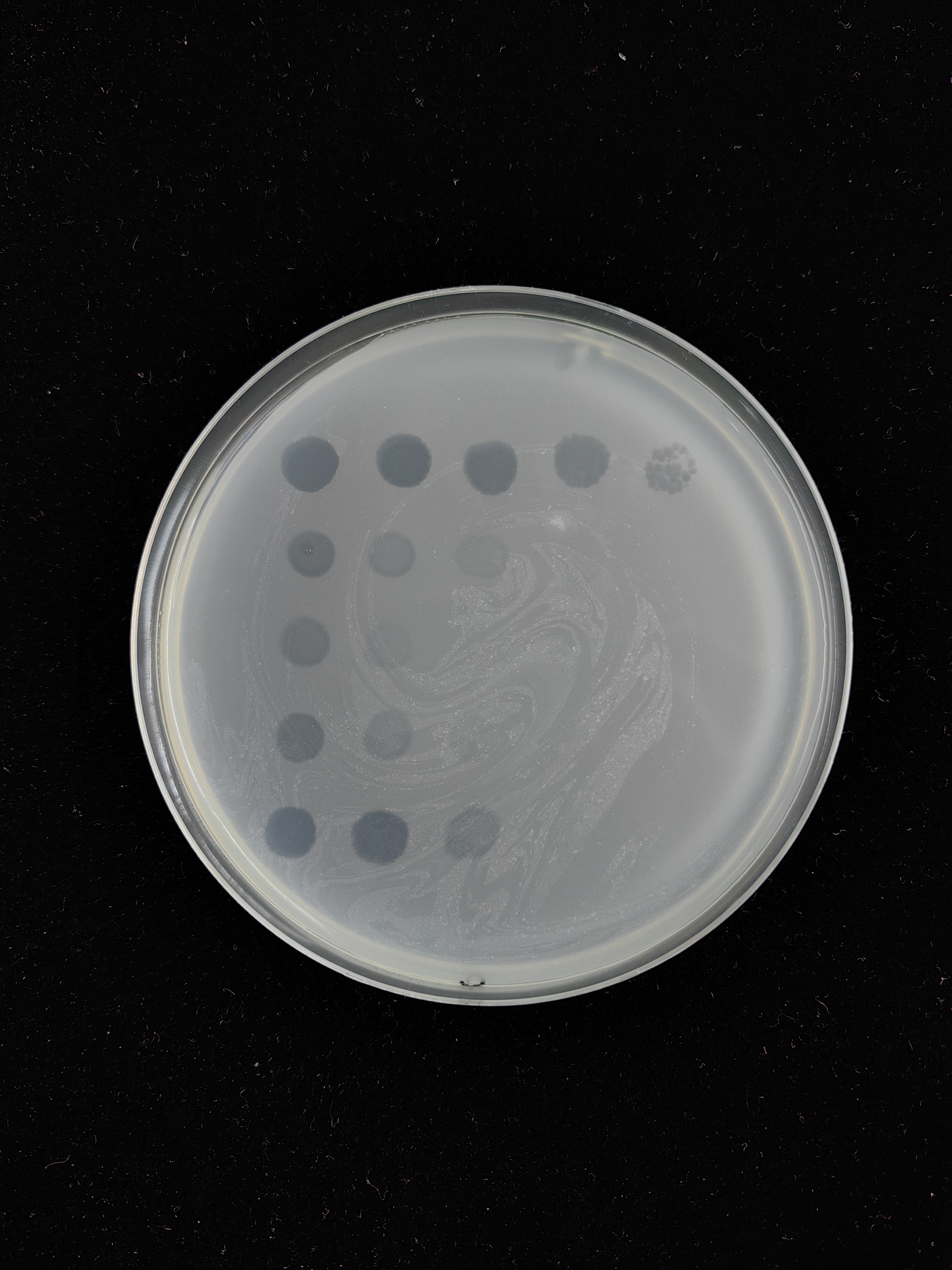

Supplement: Supplementary file 12 — Figure S7 Source Data [file 44319_2025_488_MOESM12_ESM.zip › Appendix Figure S7/S7A/pJR962-Mra_3122-3 with ATc induction.tiff]

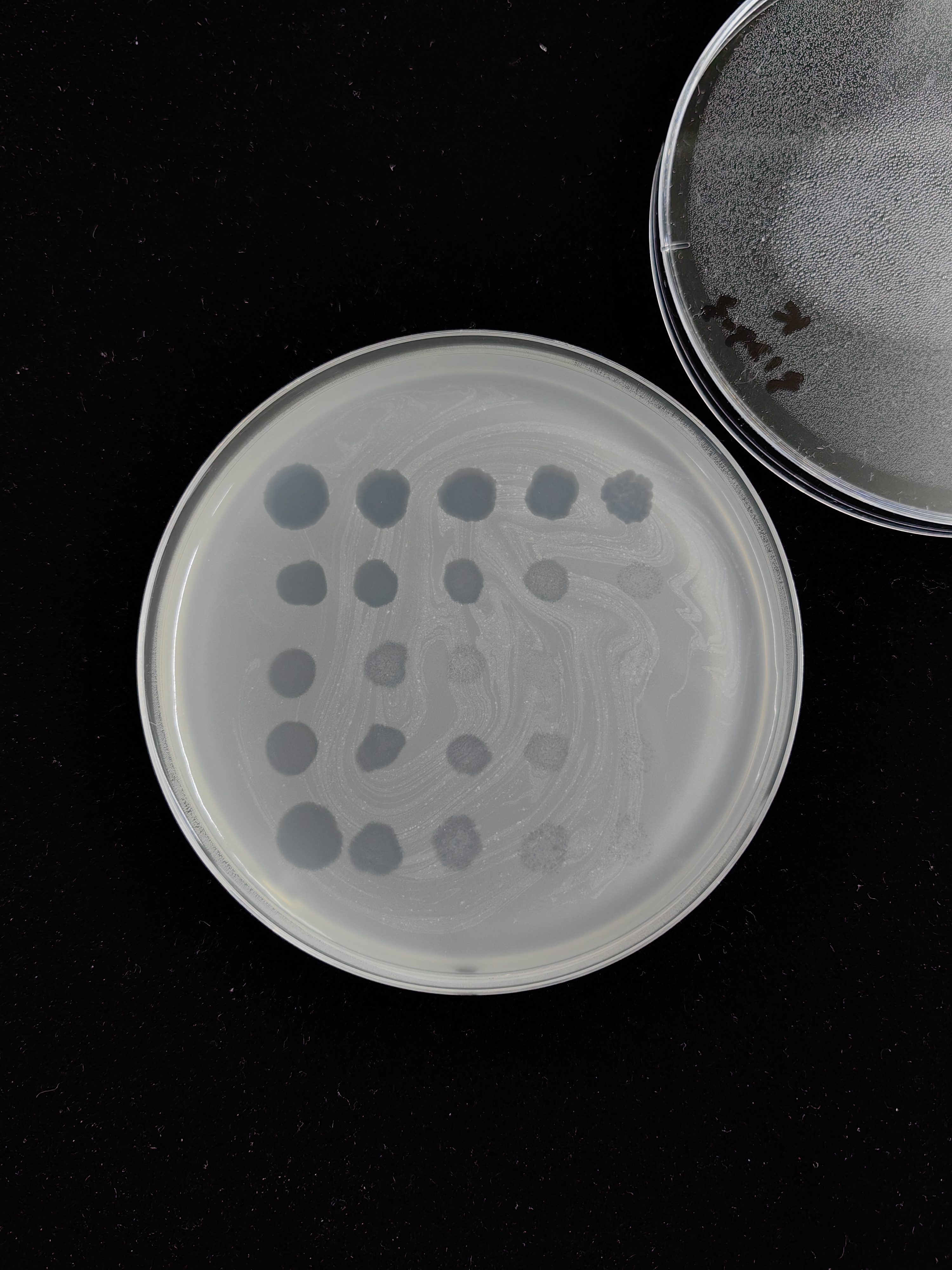

Supplement: Supplementary file 12 — Figure S7 Source Data [file 44319_2025_488_MOESM12_ESM.zip › Appendix Figure S7/S7A/pJR962-Mra_3122-3 without ATc induction.tiff]

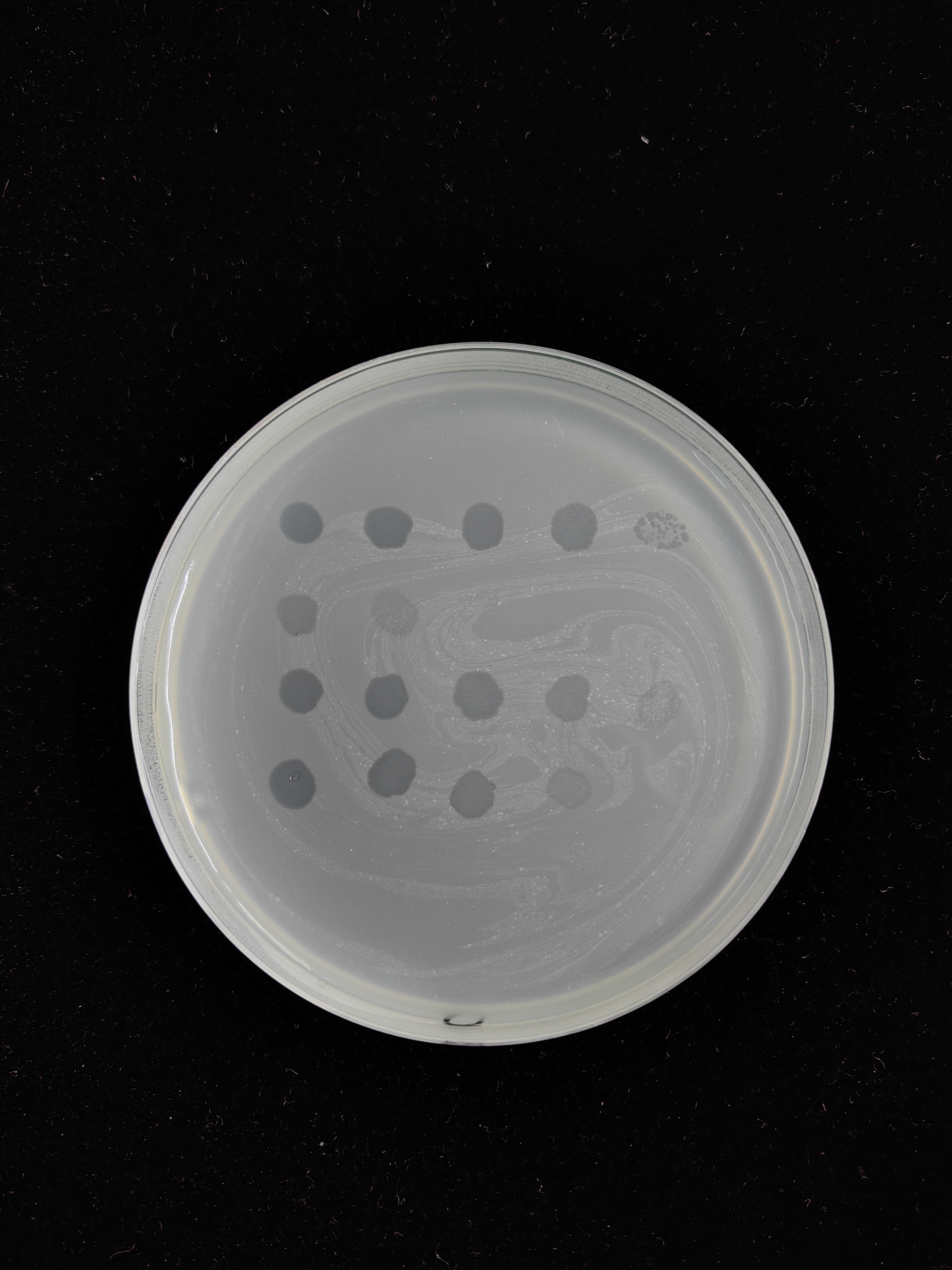

Supplement: Supplementary file 12 — Figure S7 Source Data [file 44319_2025_488_MOESM12_ESM.zip › Appendix Figure S7/S7A/pJR962-Mra_3122-4 with ATc induction.tiff]

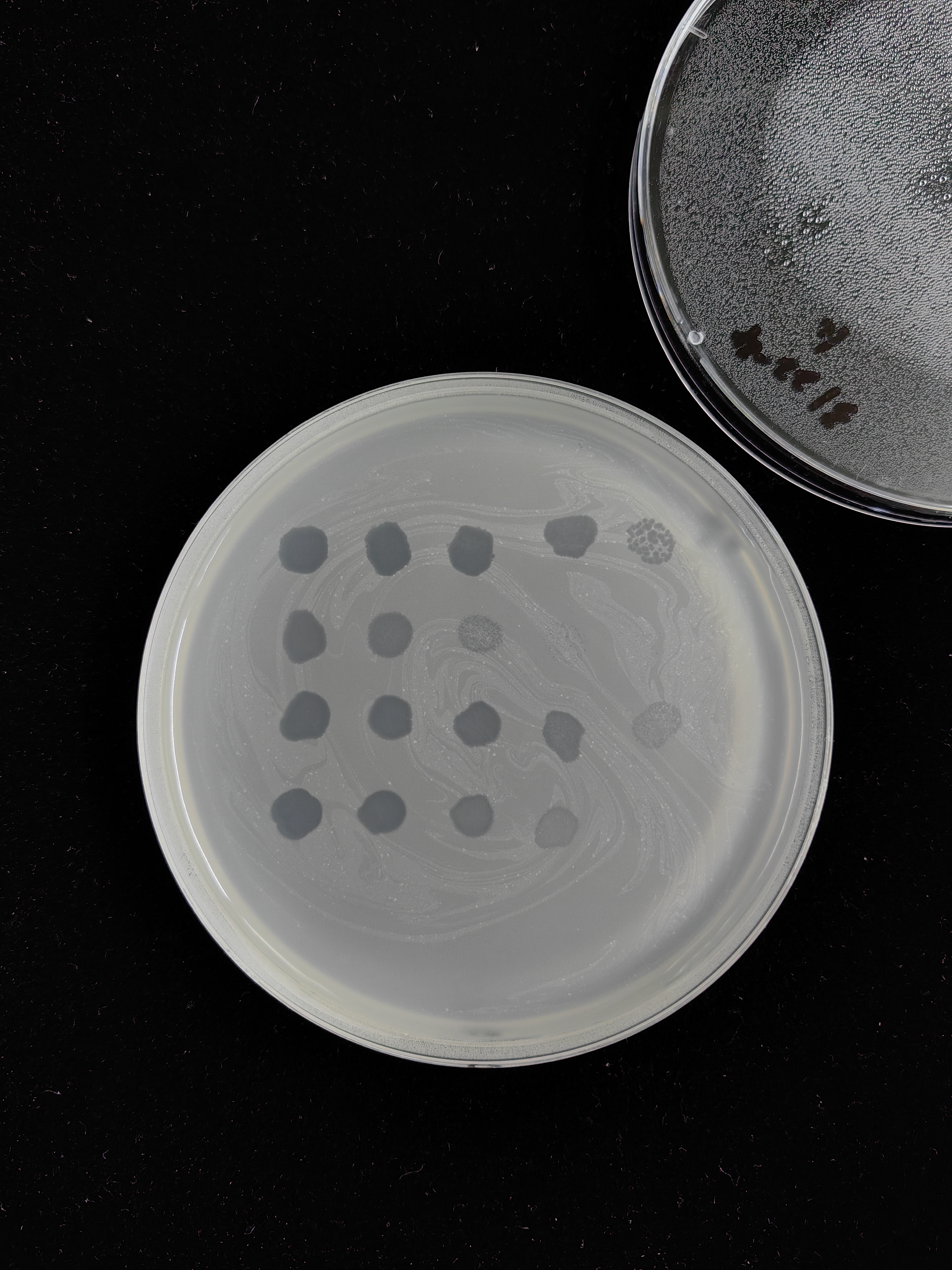

Supplement: Supplementary file 12 — Figure S7 Source Data [file 44319_2025_488_MOESM12_ESM.zip › Appendix Figure S7/S7A/pJR962-Mra_3122-4 without ATc induction.tiff]

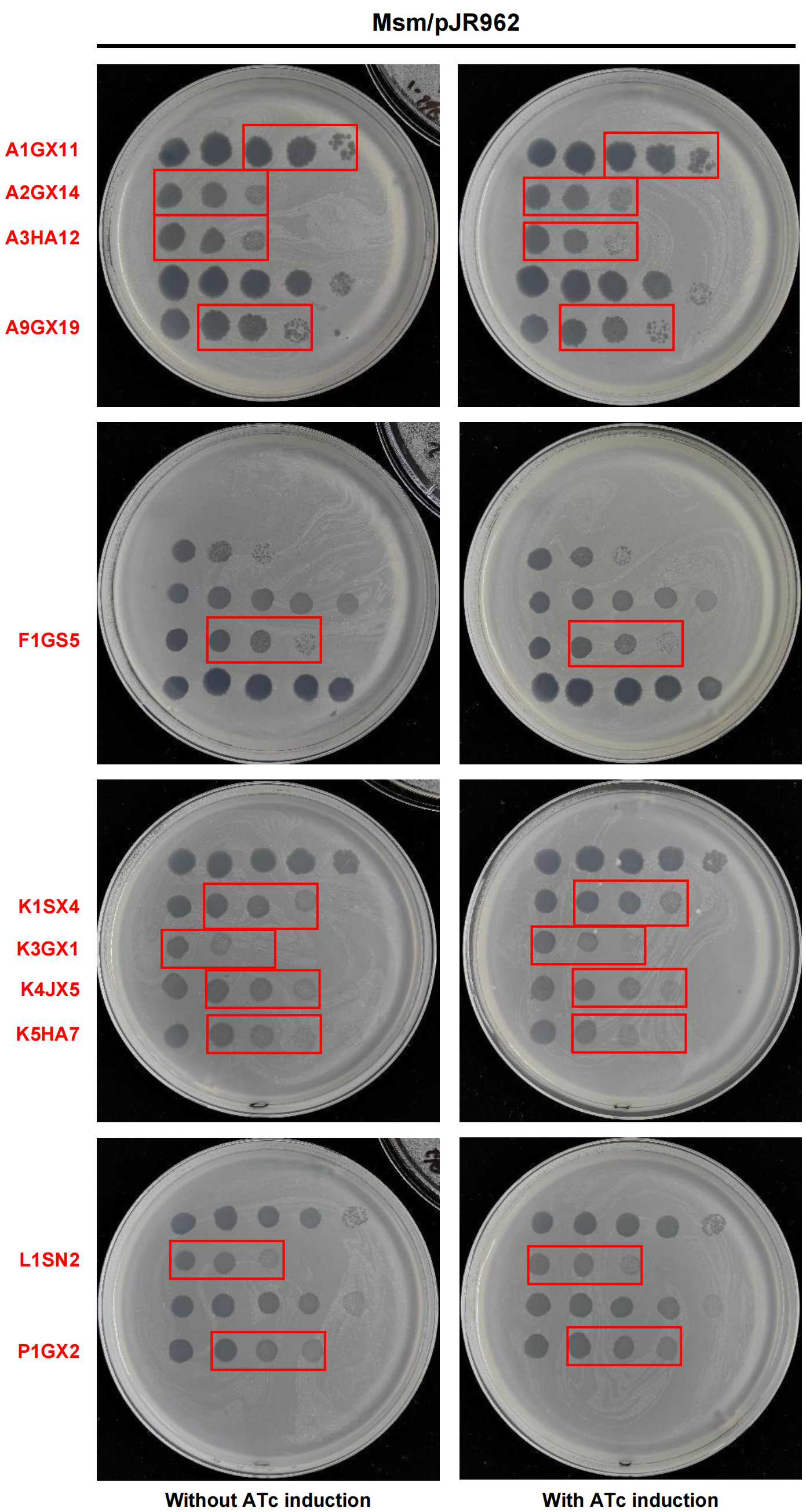

Supplement: Supplementary file 12 — Figure S7 Source Data [file 44319_2025_488_MOESM12_ESM.zip › Appendix Figure S7/S7A/README-1.tif]

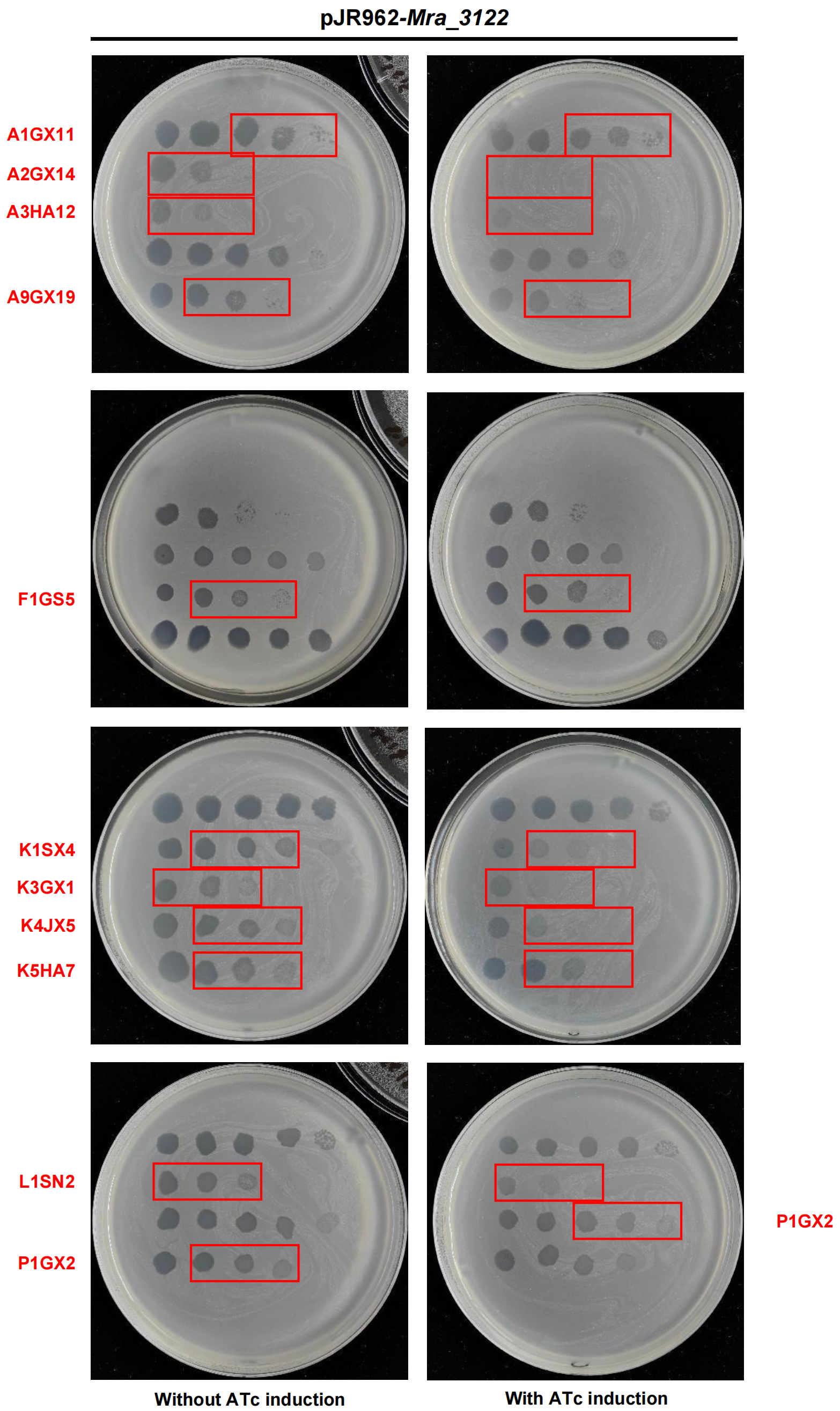

Supplement: Supplementary file 12 — Figure S7 Source Data [file 44319_2025_488_MOESM12_ESM.zip › Appendix Figure S7/S7A/README-2.tif]

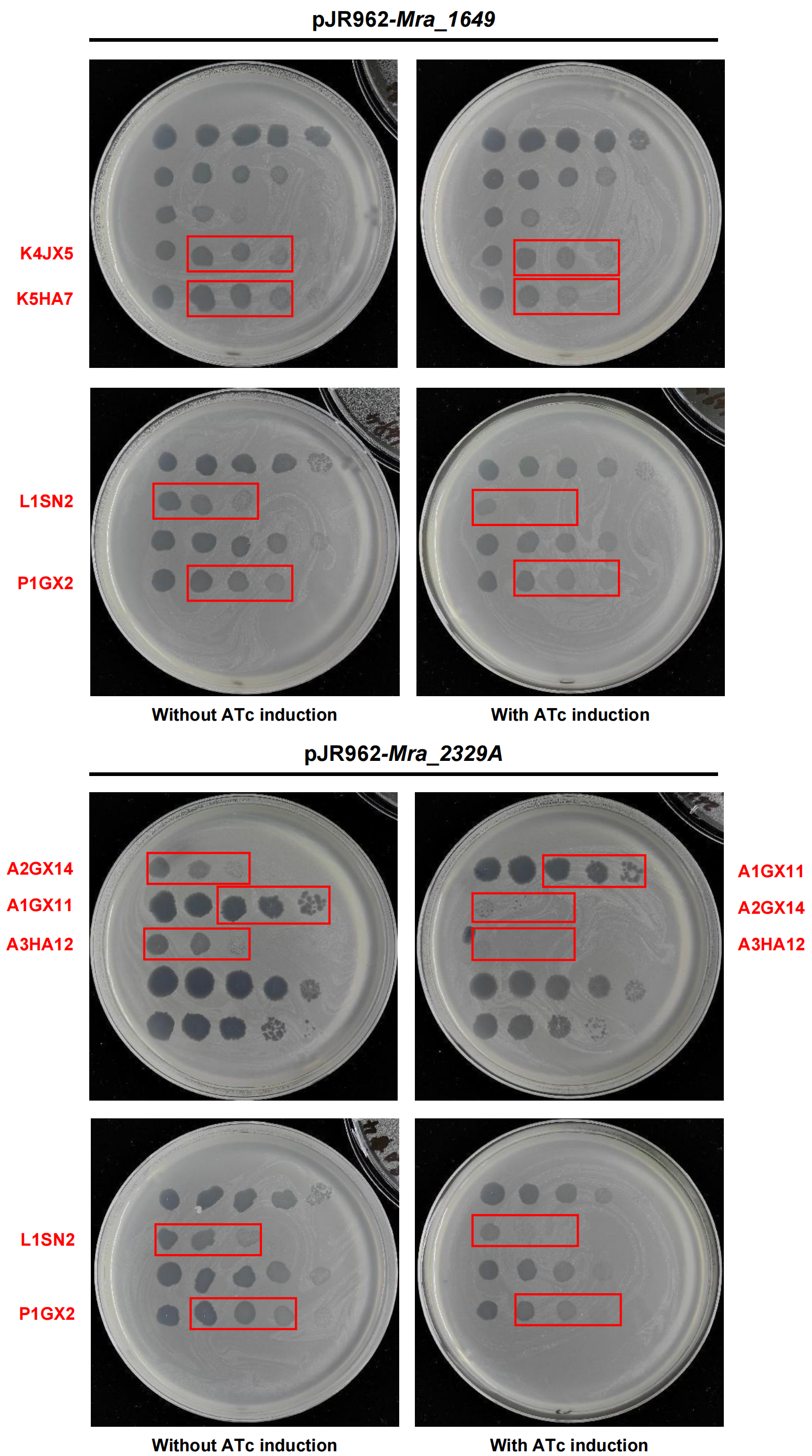

Supplement: Supplementary file 12 — Figure S7 Source Data [file 44319_2025_488_MOESM12_ESM.zip › Appendix Figure S7/S7A/README-3.tif]

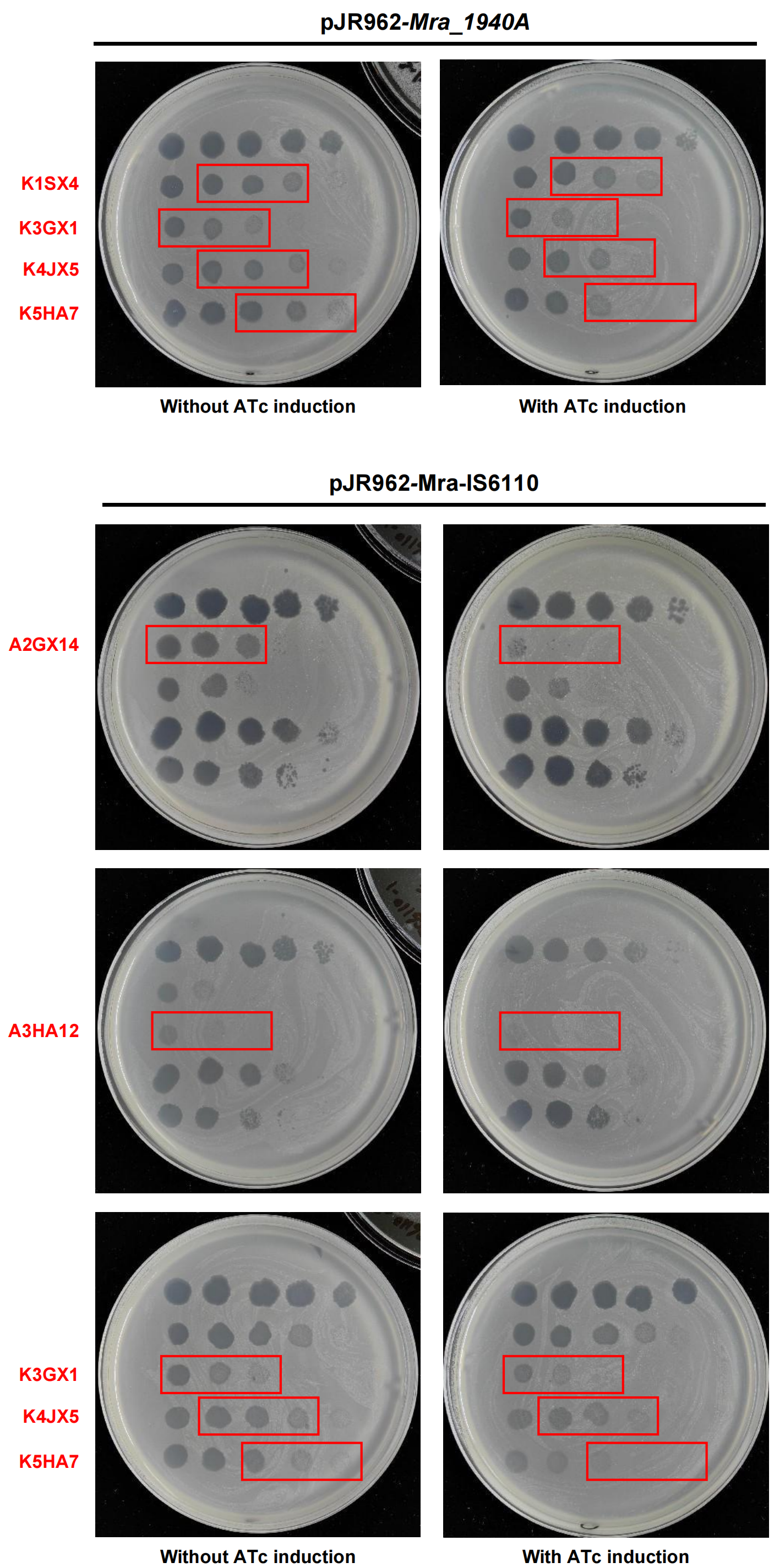

Supplement: Supplementary file 12 — Figure S7 Source Data [file 44319_2025_488_MOESM12_ESM.zip › Appendix Figure S7/S7A/README-4.tif]

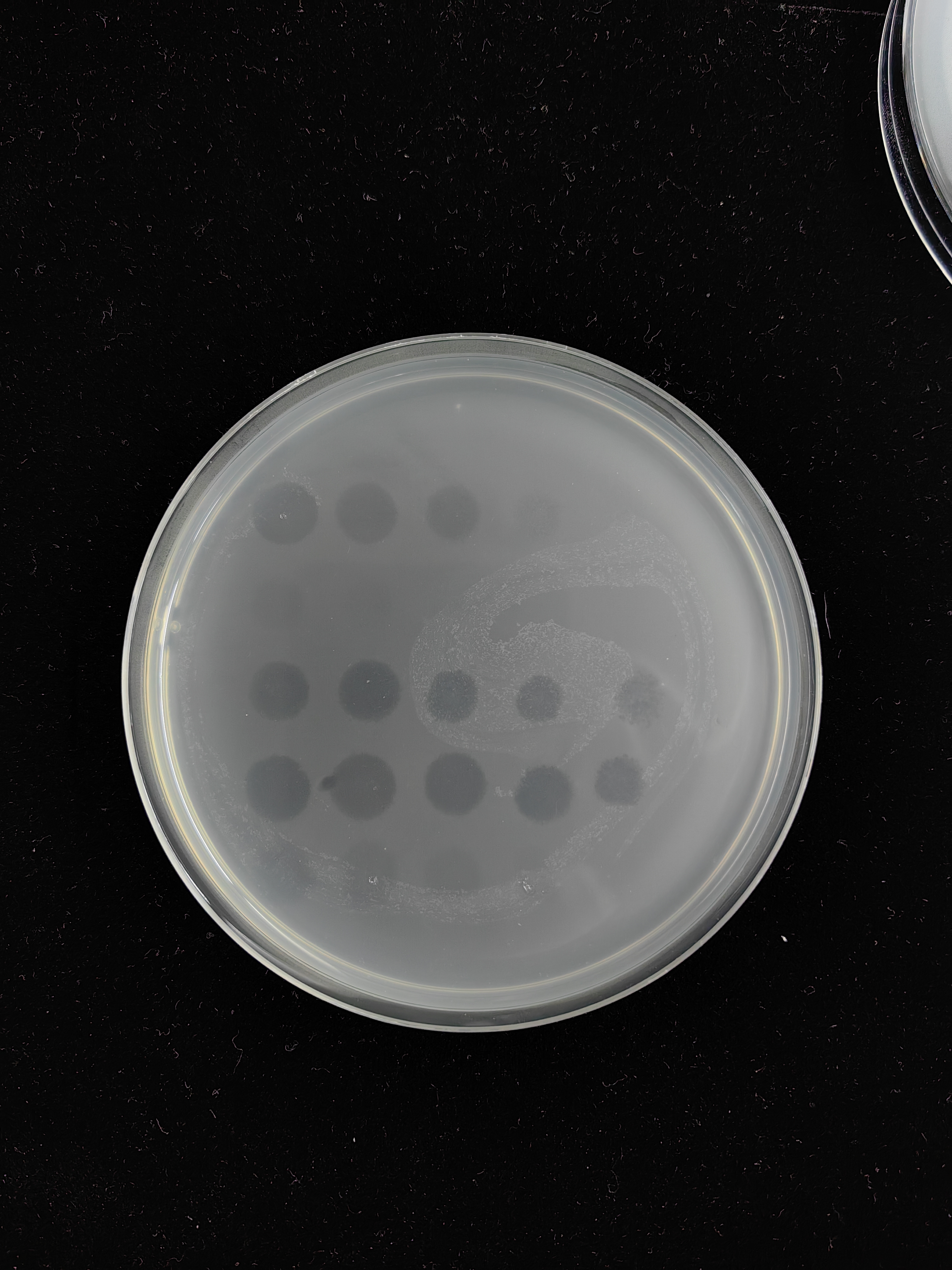

Supplement: Supplementary file 12 — Figure S7 Source Data [file 44319_2025_488_MOESM12_ESM.zip › Appendix Figure S7/S7B/pJR962-1 with ATc induction.tiff]

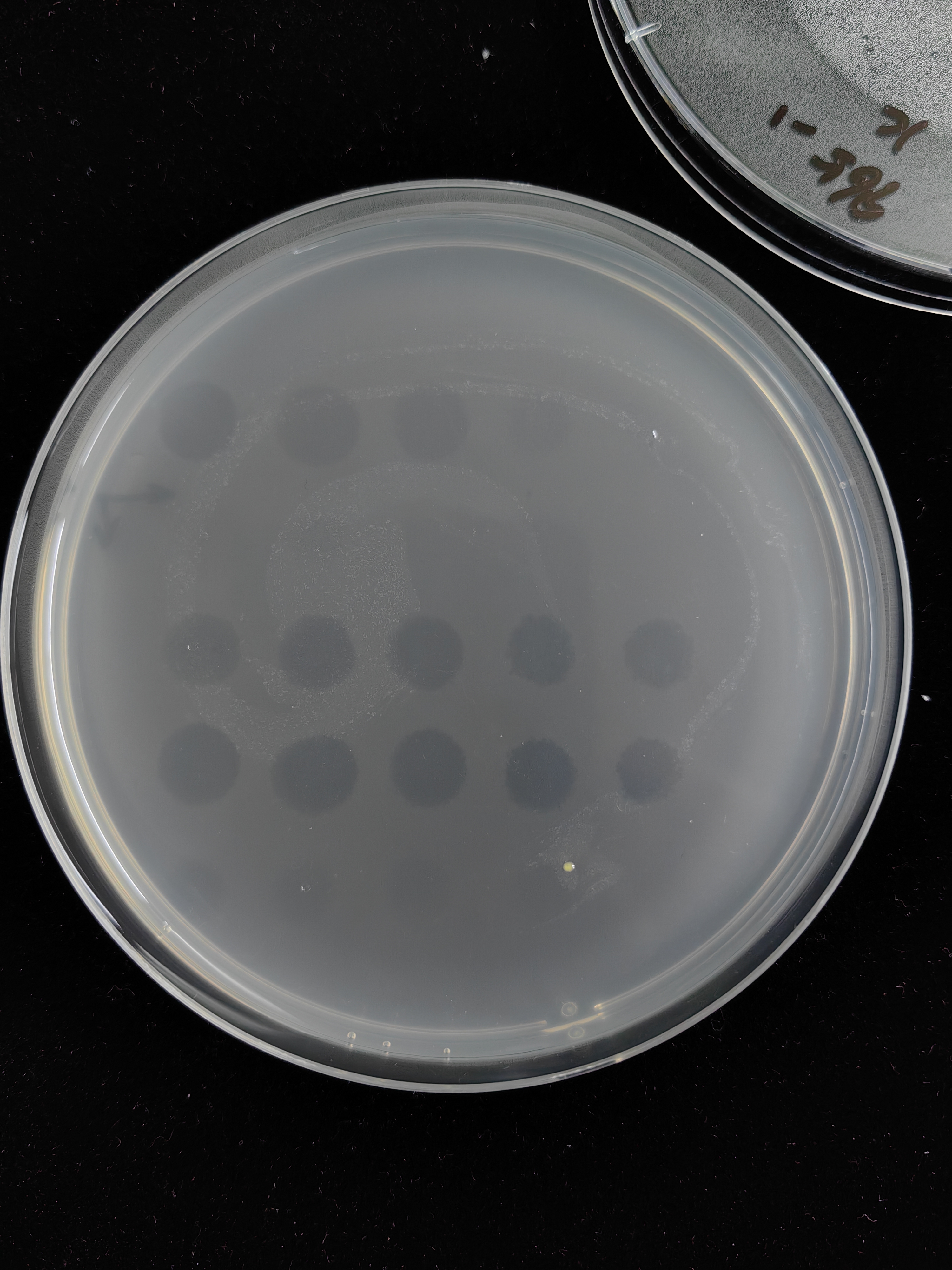

Supplement: Supplementary file 12 — Figure S7 Source Data [file 44319_2025_488_MOESM12_ESM.zip › Appendix Figure S7/S7B/pJR962-1 without ATc induction.tiff]

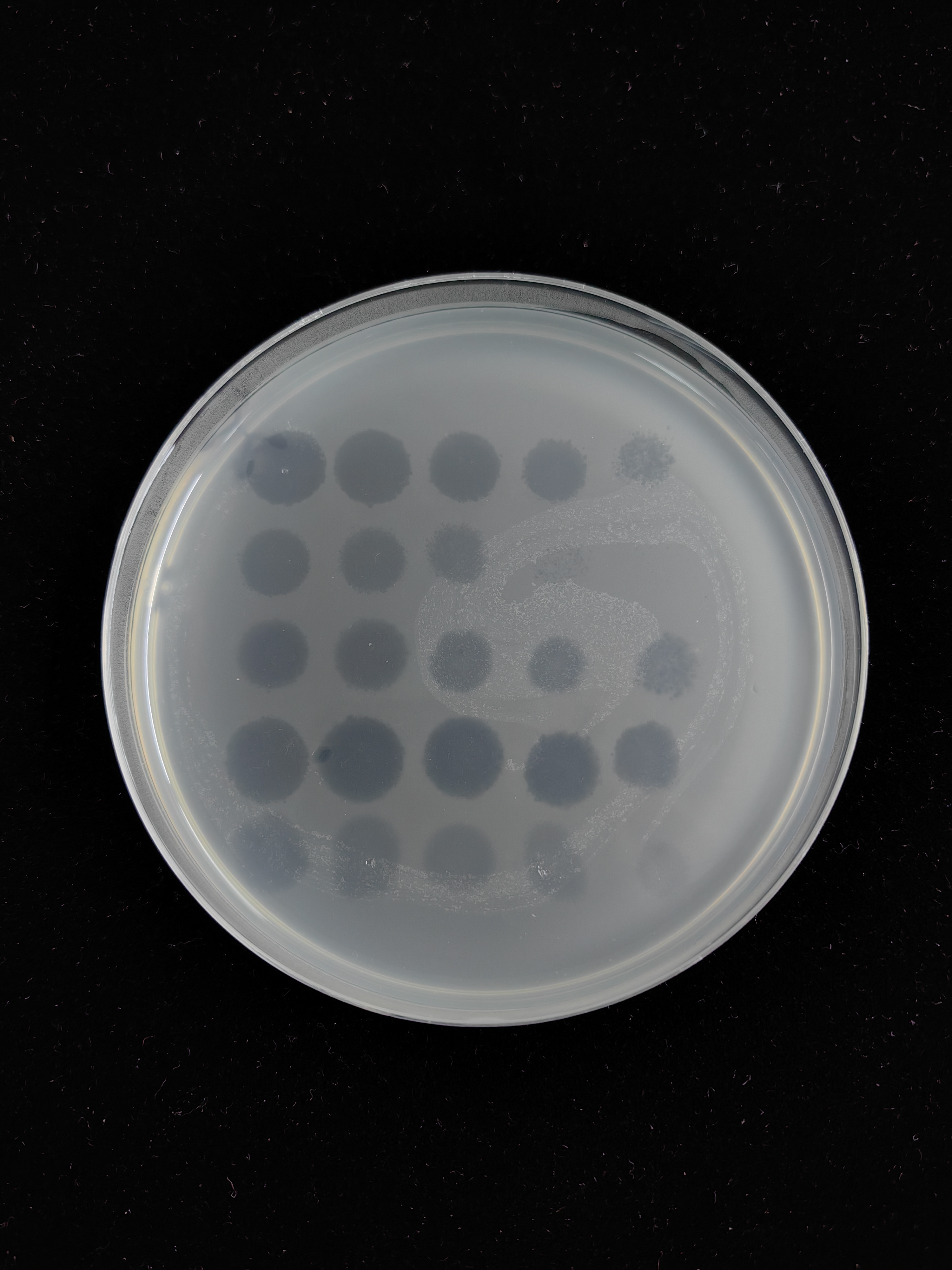

Supplement: Supplementary file 12 — Figure S7 Source Data [file 44319_2025_488_MOESM12_ESM.zip › Appendix Figure S7/S7B/pJR962-2 with ATc induction.tiff]

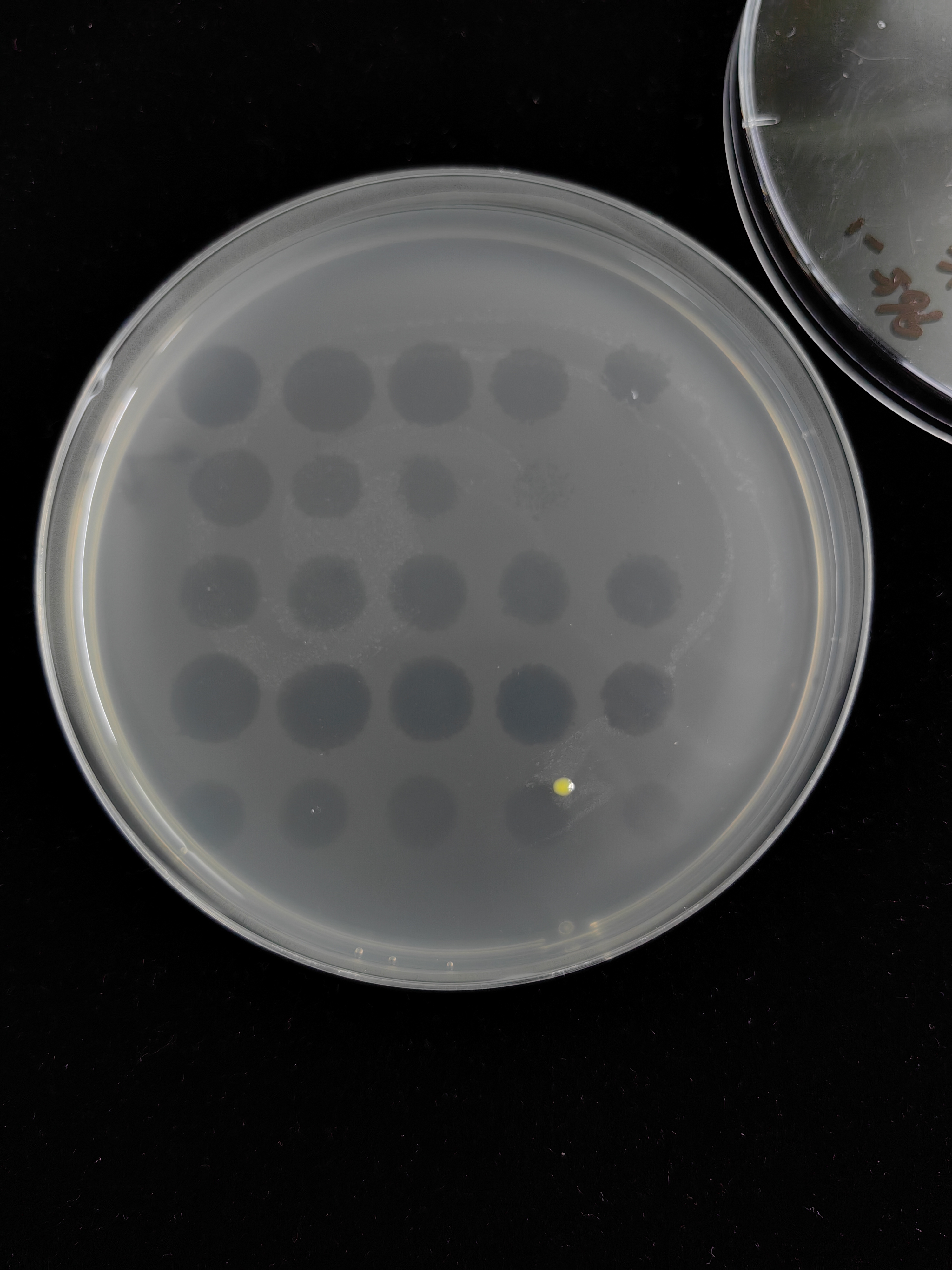

Supplement: Supplementary file 12 — Figure S7 Source Data [file 44319_2025_488_MOESM12_ESM.zip › Appendix Figure S7/S7B/pJR962-2 without ATc induction.tiff]

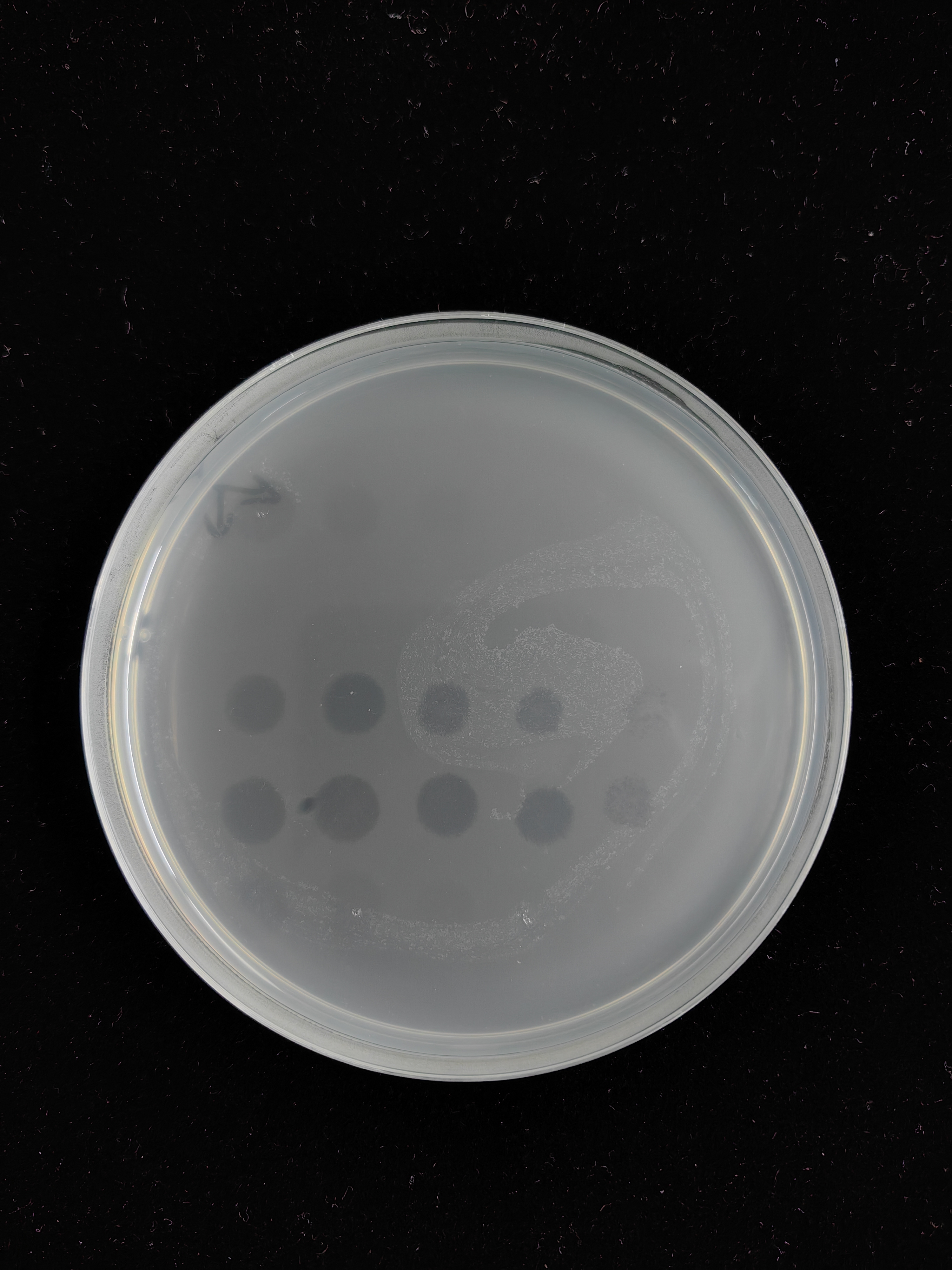

Supplement: Supplementary file 12 — Figure S7 Source Data [file 44319_2025_488_MOESM12_ESM.zip › Appendix Figure S7/S7B/pJR962-3 with ATc induction.tiff]

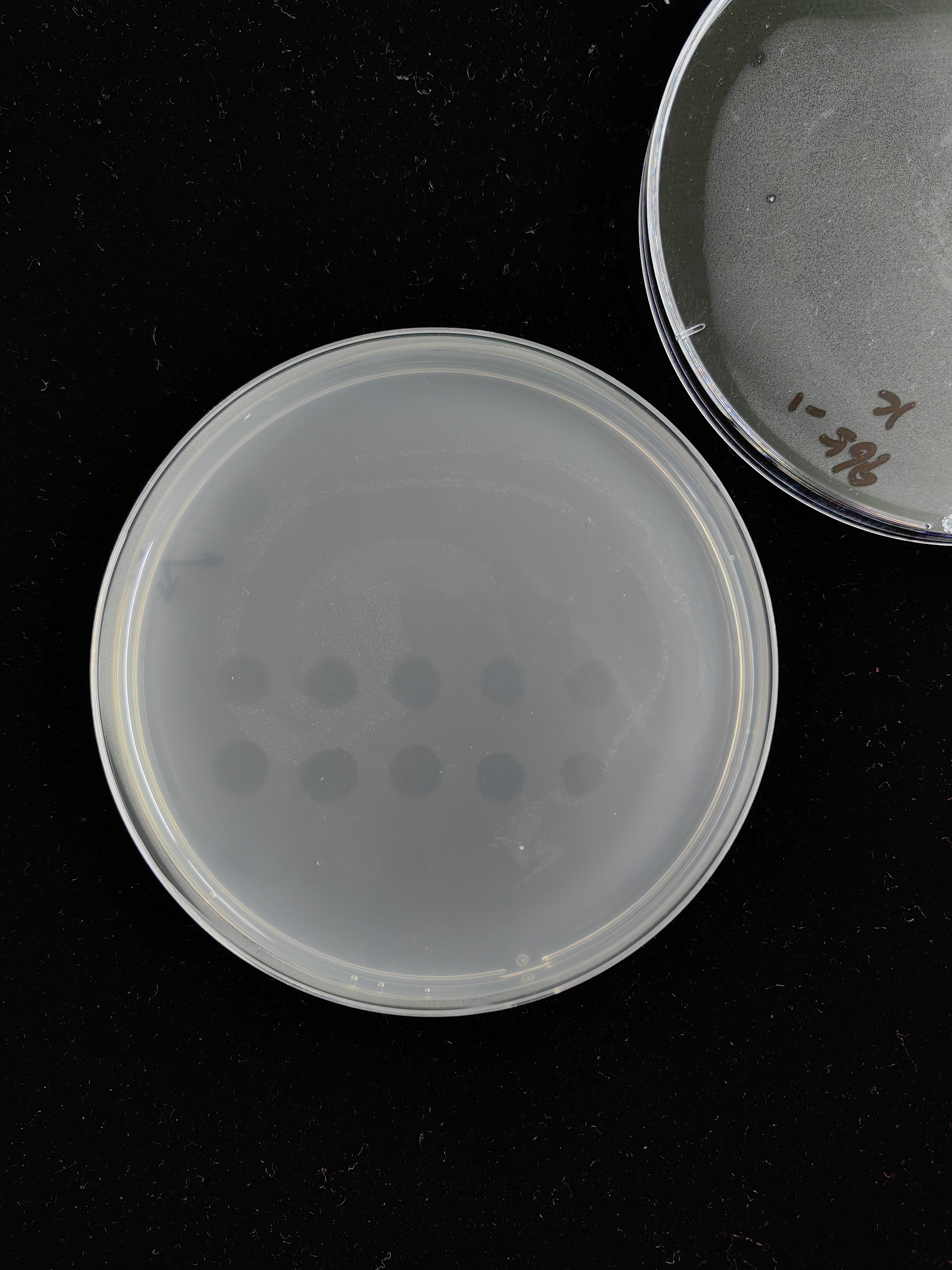

Supplement: Supplementary file 12 — Figure S7 Source Data [file 44319_2025_488_MOESM12_ESM.zip › Appendix Figure S7/S7B/pJR962-3 without ATc induction.tiff]

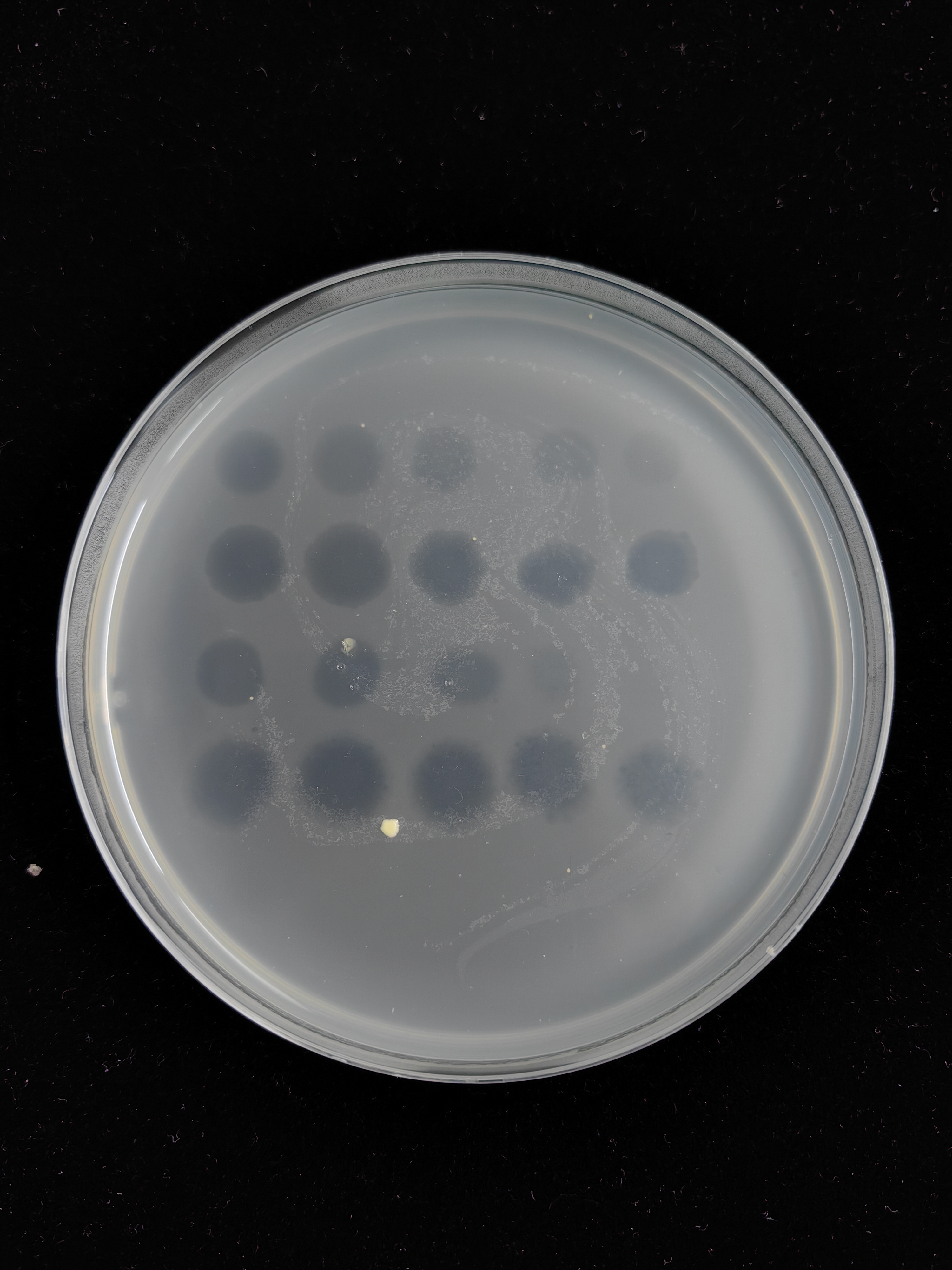

Supplement: Supplementary file 12 — Figure S7 Source Data [file 44319_2025_488_MOESM12_ESM.zip › Appendix Figure S7/S7B/pJR962-4 with ATc induction.tiff]

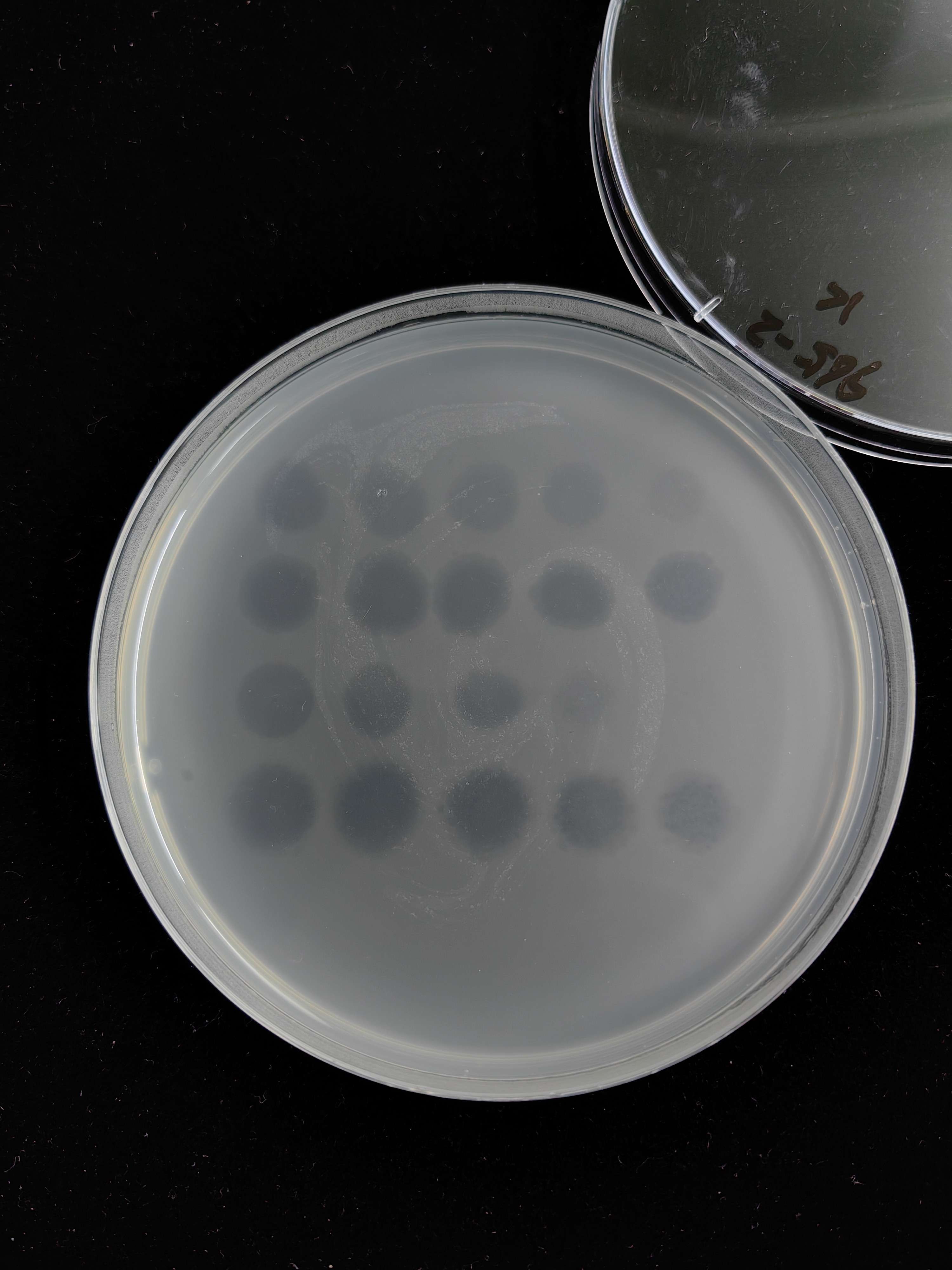

Supplement: Supplementary file 12 — Figure S7 Source Data [file 44319_2025_488_MOESM12_ESM.zip › Appendix Figure S7/S7B/pJR962-4 without ATc induction.tiff]

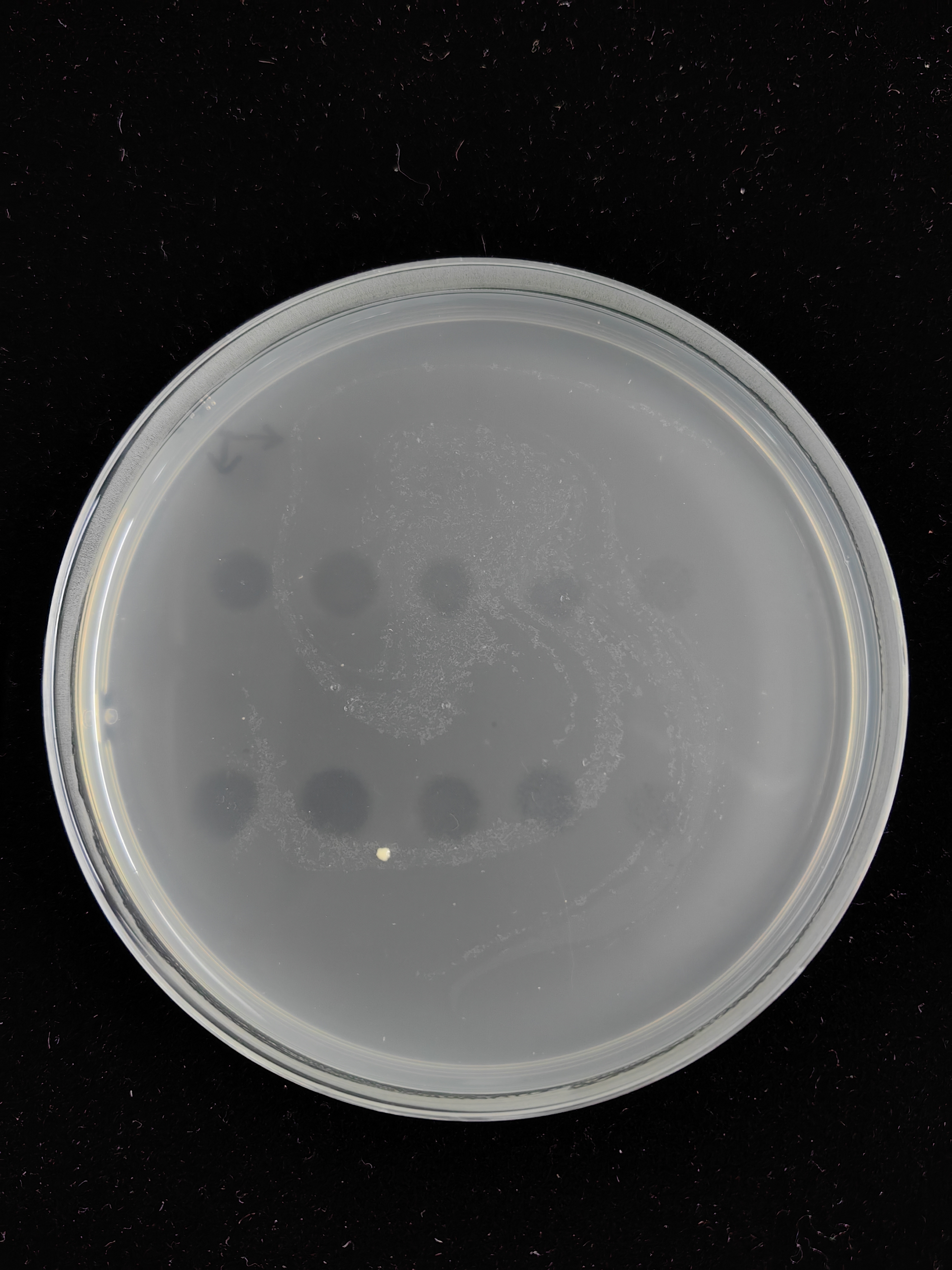

Supplement: Supplementary file 12 — Figure S7 Source Data [file 44319_2025_488_MOESM12_ESM.zip › Appendix Figure S7/S7B/pJR962-5 with ATc induction.tiff]

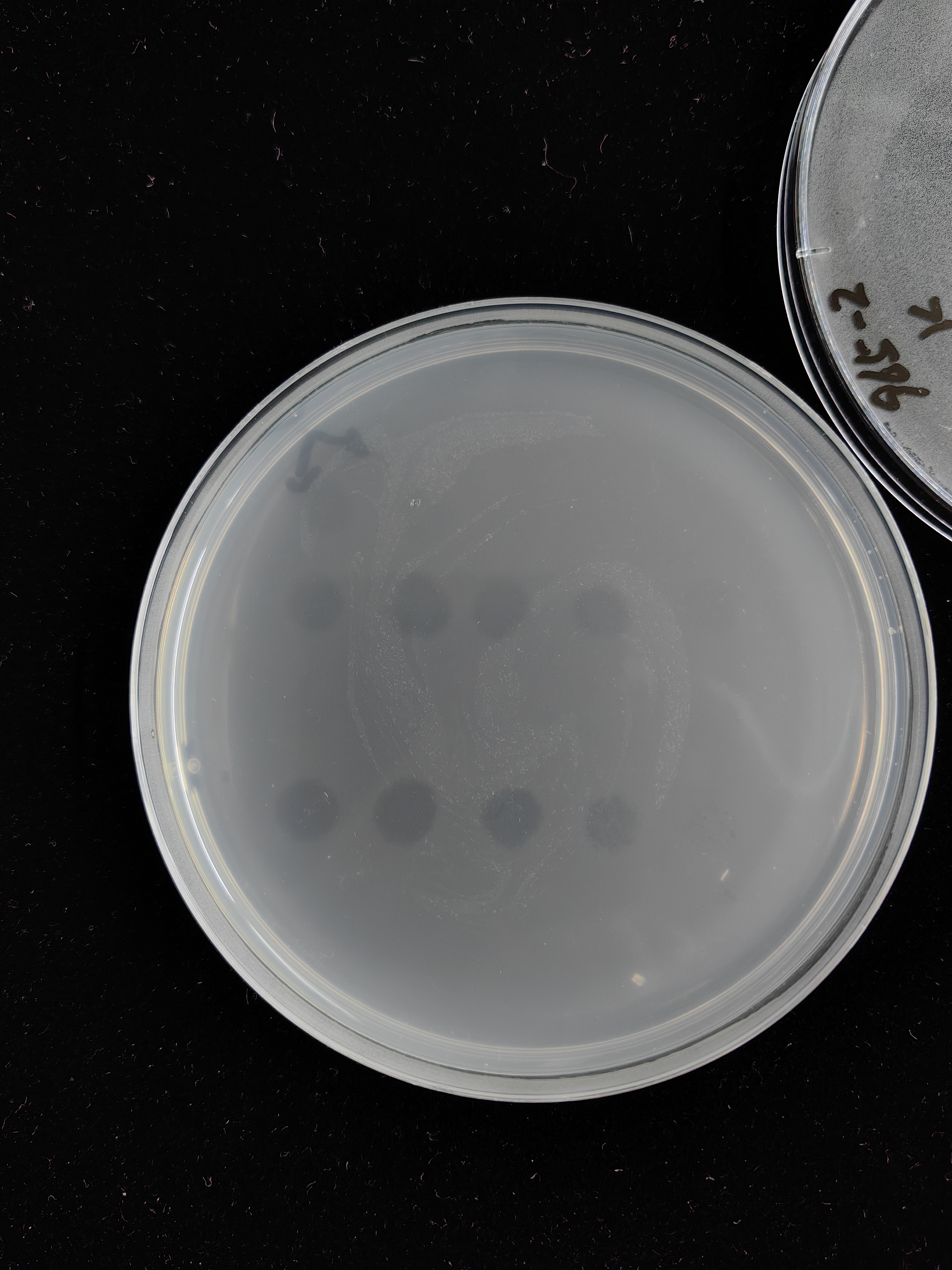

Supplement: Supplementary file 12 — Figure S7 Source Data [file 44319_2025_488_MOESM12_ESM.zip › Appendix Figure S7/S7B/pJR962-5 without ATc induction.tiff]

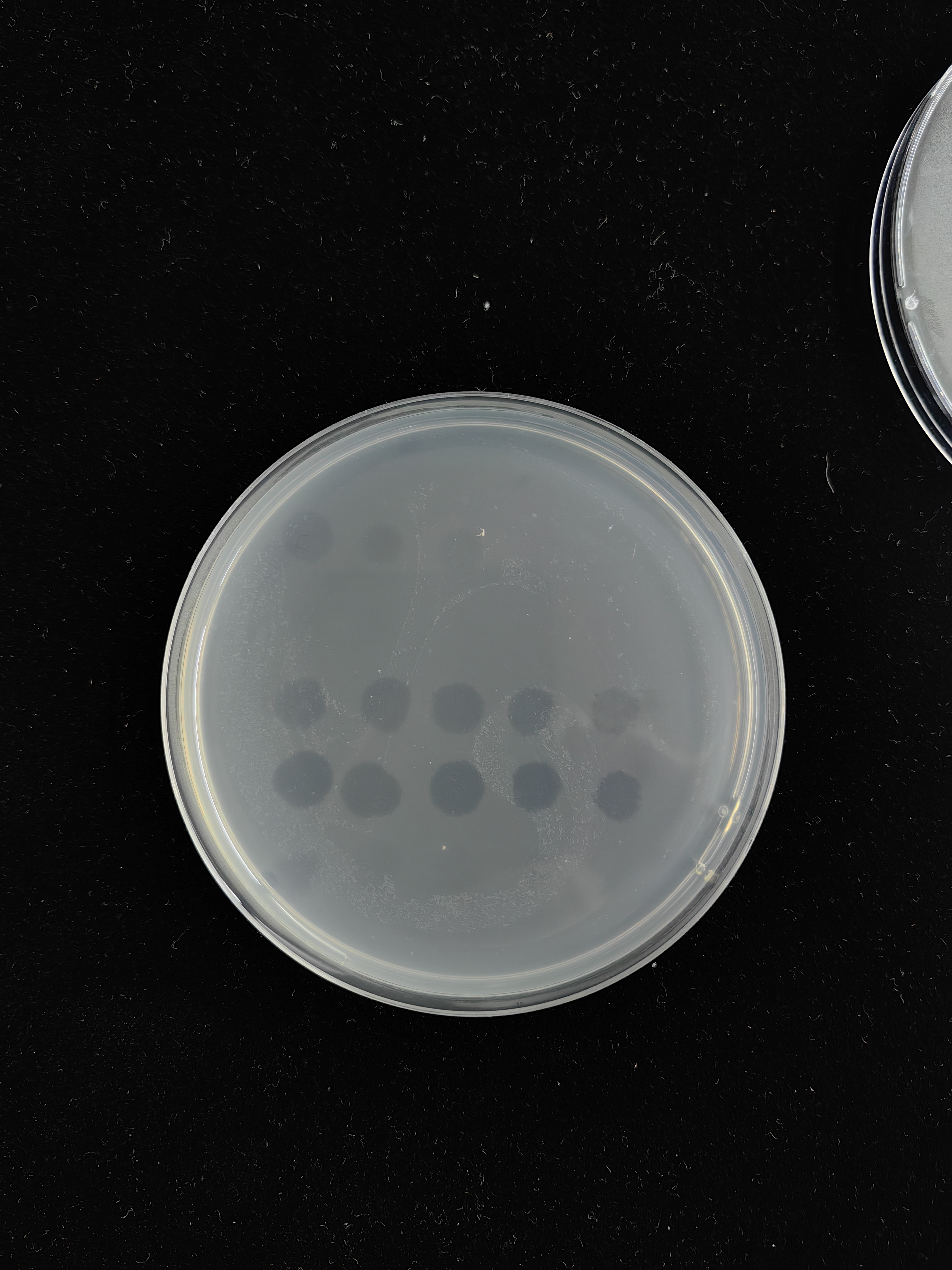

Supplement: Supplementary file 12 — Figure S7 Source Data [file 44319_2025_488_MOESM12_ESM.zip › Appendix Figure S7/S7B/pJR962-Mra_1940A-1 with ATc induction.tiff]

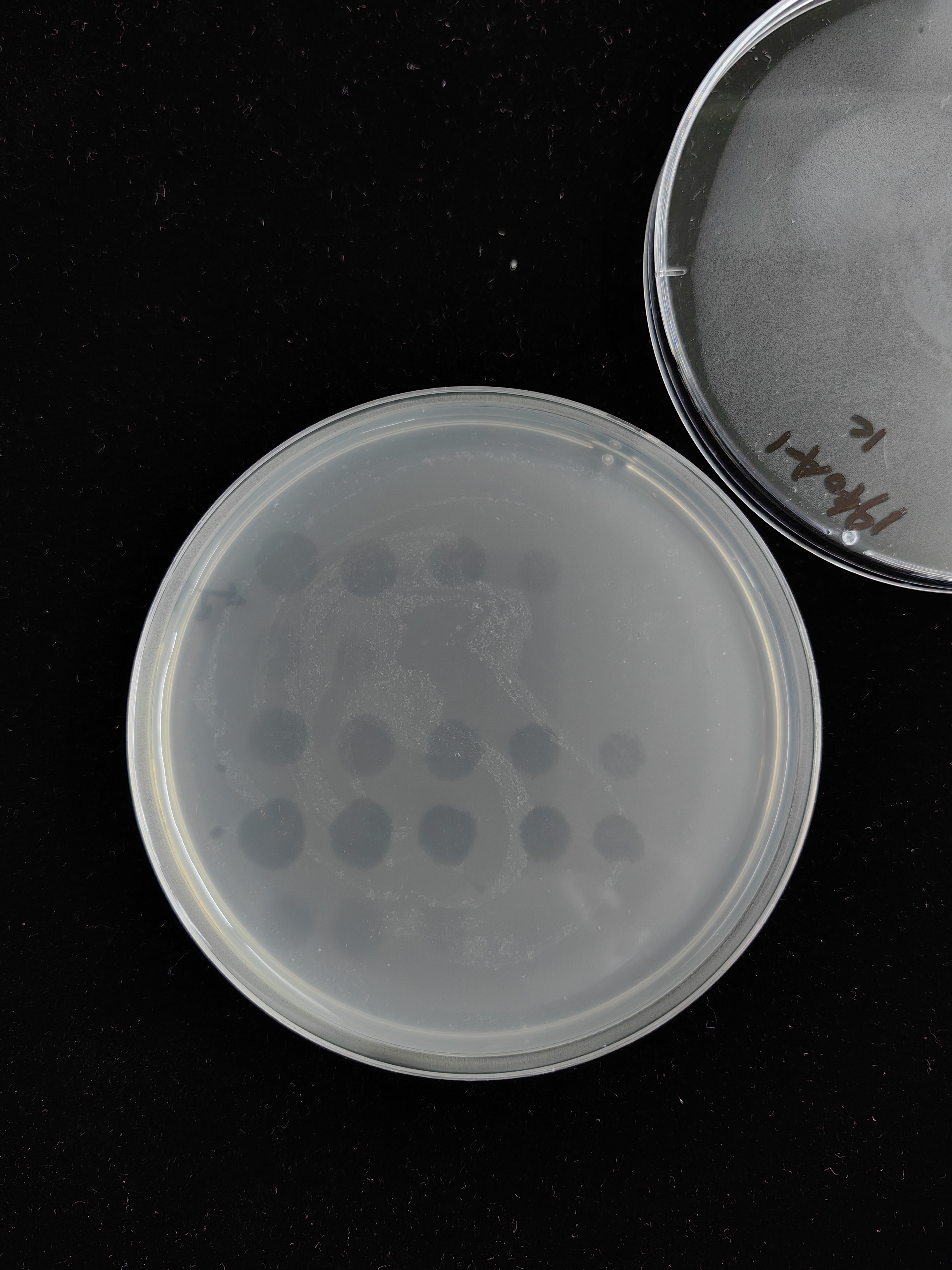

Supplement: Supplementary file 12 — Figure S7 Source Data [file 44319_2025_488_MOESM12_ESM.zip › Appendix Figure S7/S7B/pJR962-Mra_1940A-1 without ATc induction.tiff]

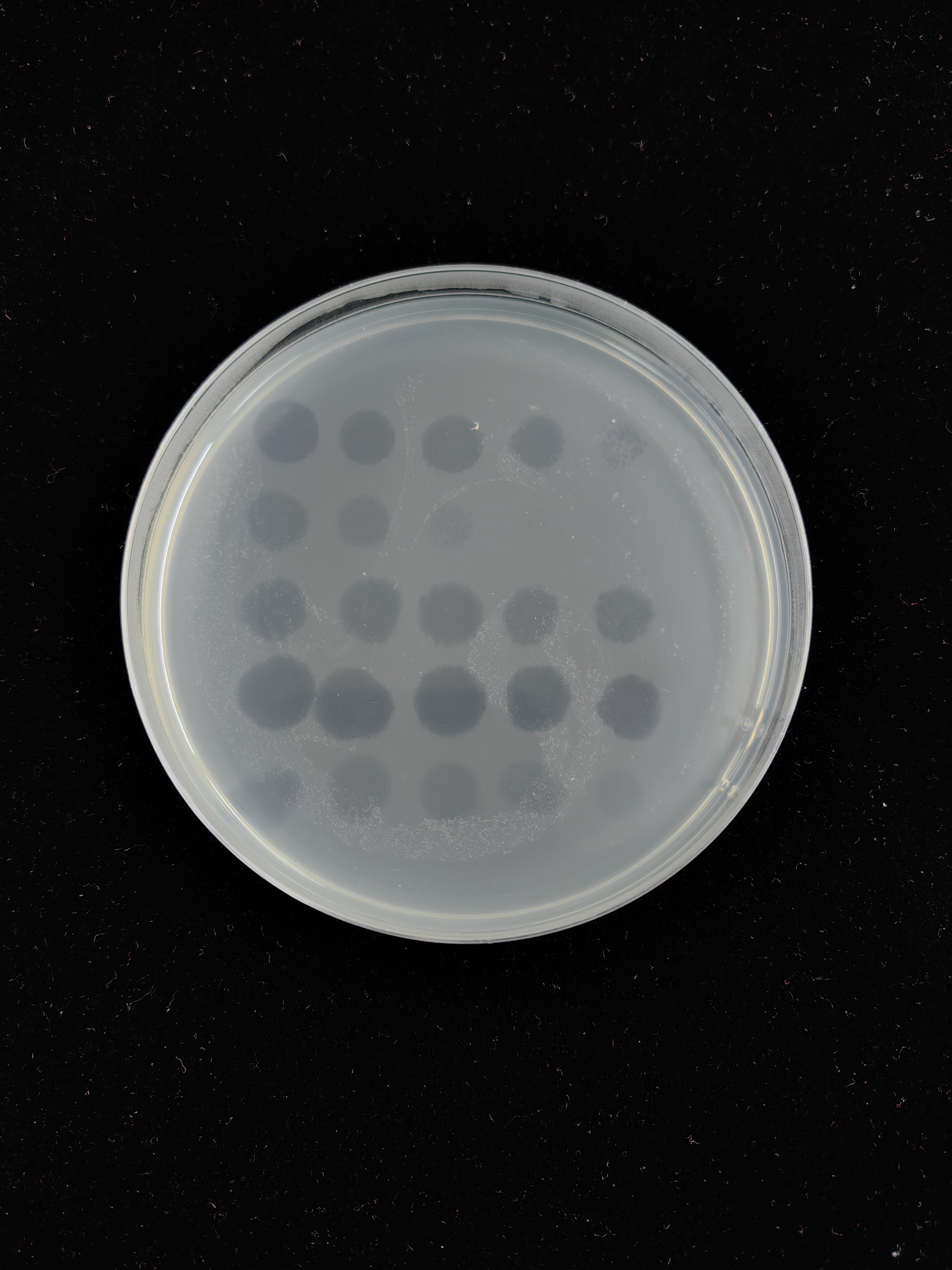

Supplement: Supplementary file 12 — Figure S7 Source Data [file 44319_2025_488_MOESM12_ESM.zip › Appendix Figure S7/S7B/pJR962-Mra_1940A-2 with ATc induction.tiff]

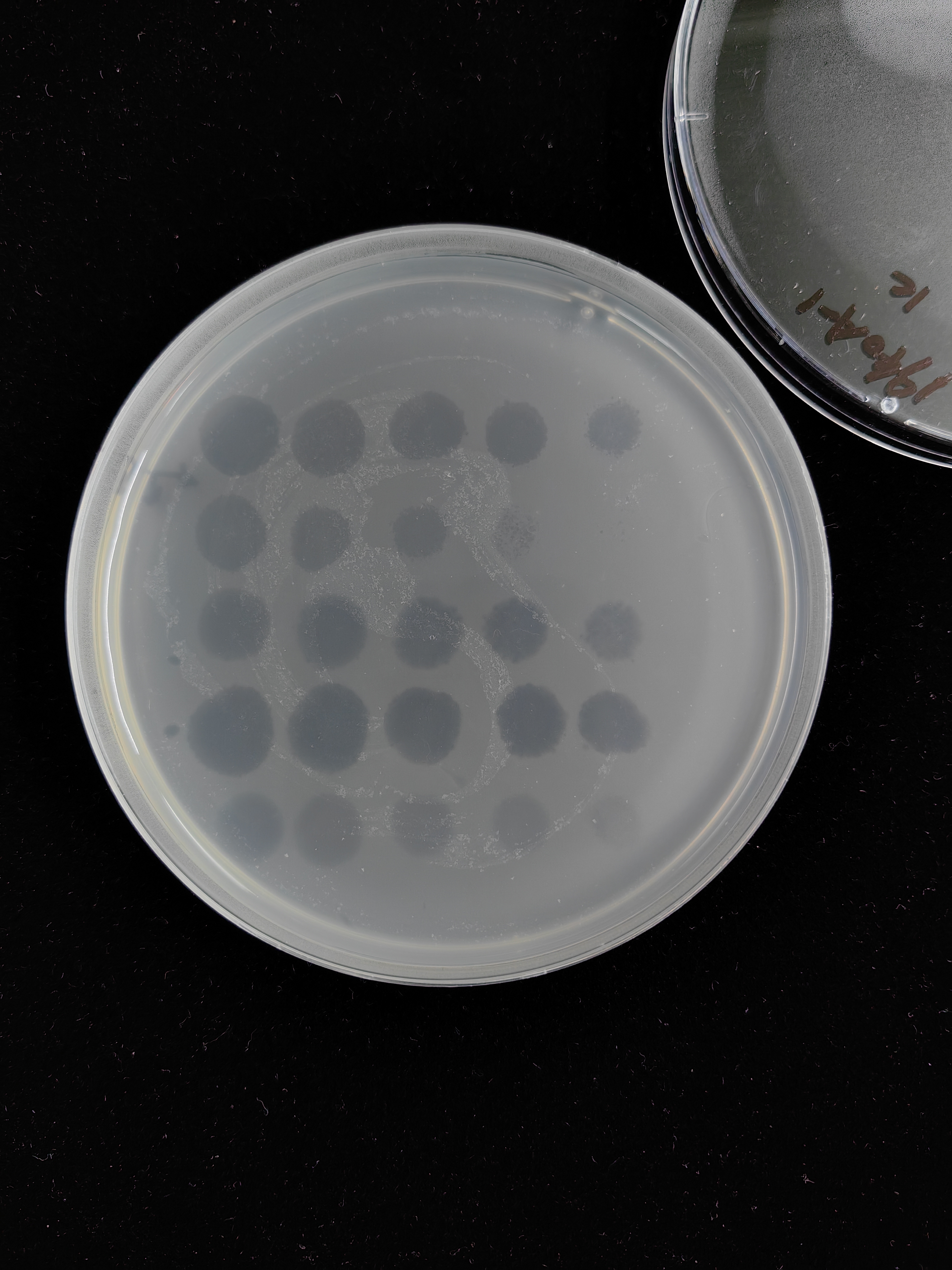

Supplement: Supplementary file 12 — Figure S7 Source Data [file 44319_2025_488_MOESM12_ESM.zip › Appendix Figure S7/S7B/pJR962-Mra_1940A-2 without ATc induction.tiff]

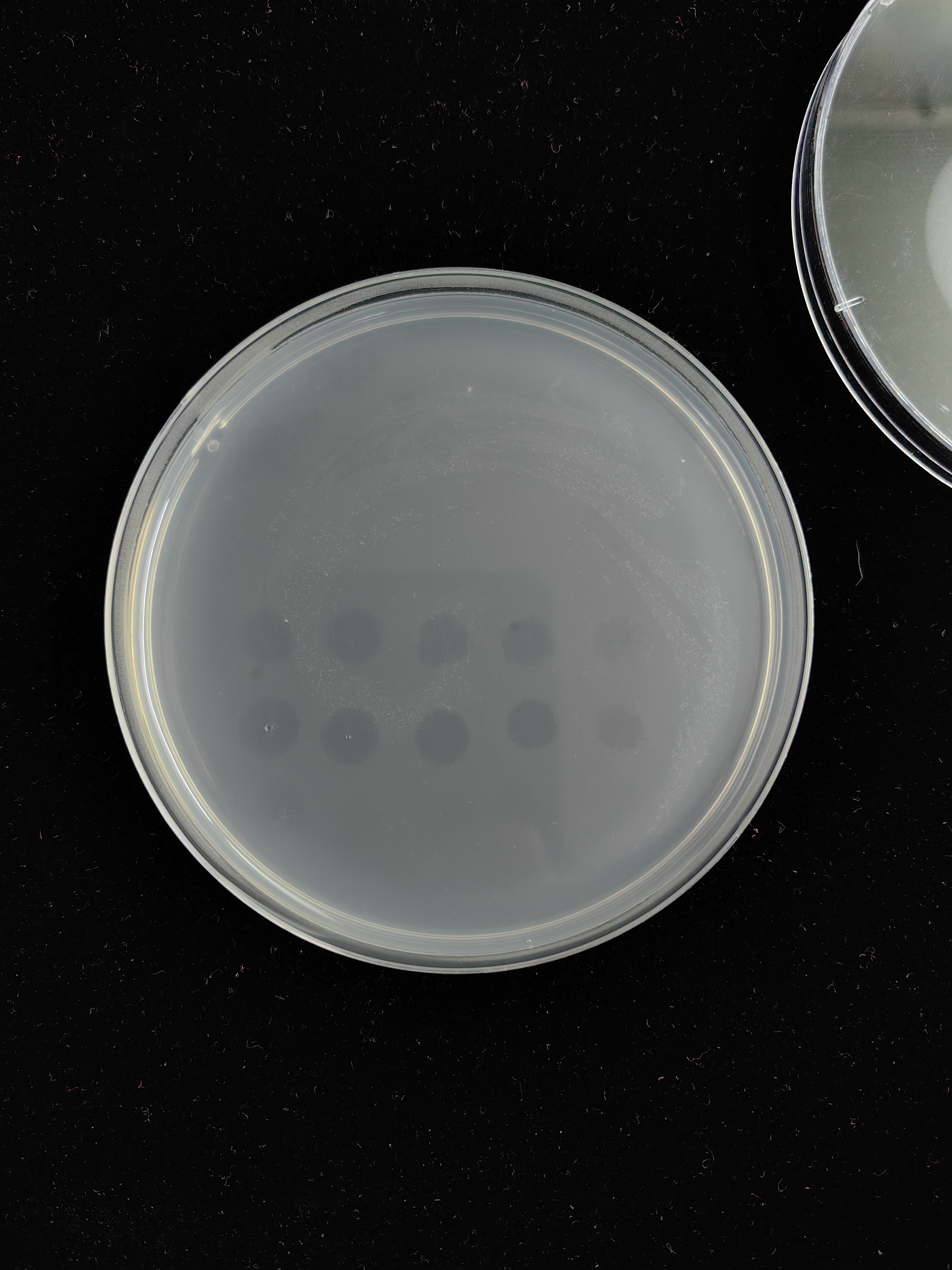

Supplement: Supplementary file 12 — Figure S7 Source Data [file 44319_2025_488_MOESM12_ESM.zip › Appendix Figure S7/S7B/pJR962-Mra_3122-1 with ATc induction.tiff]

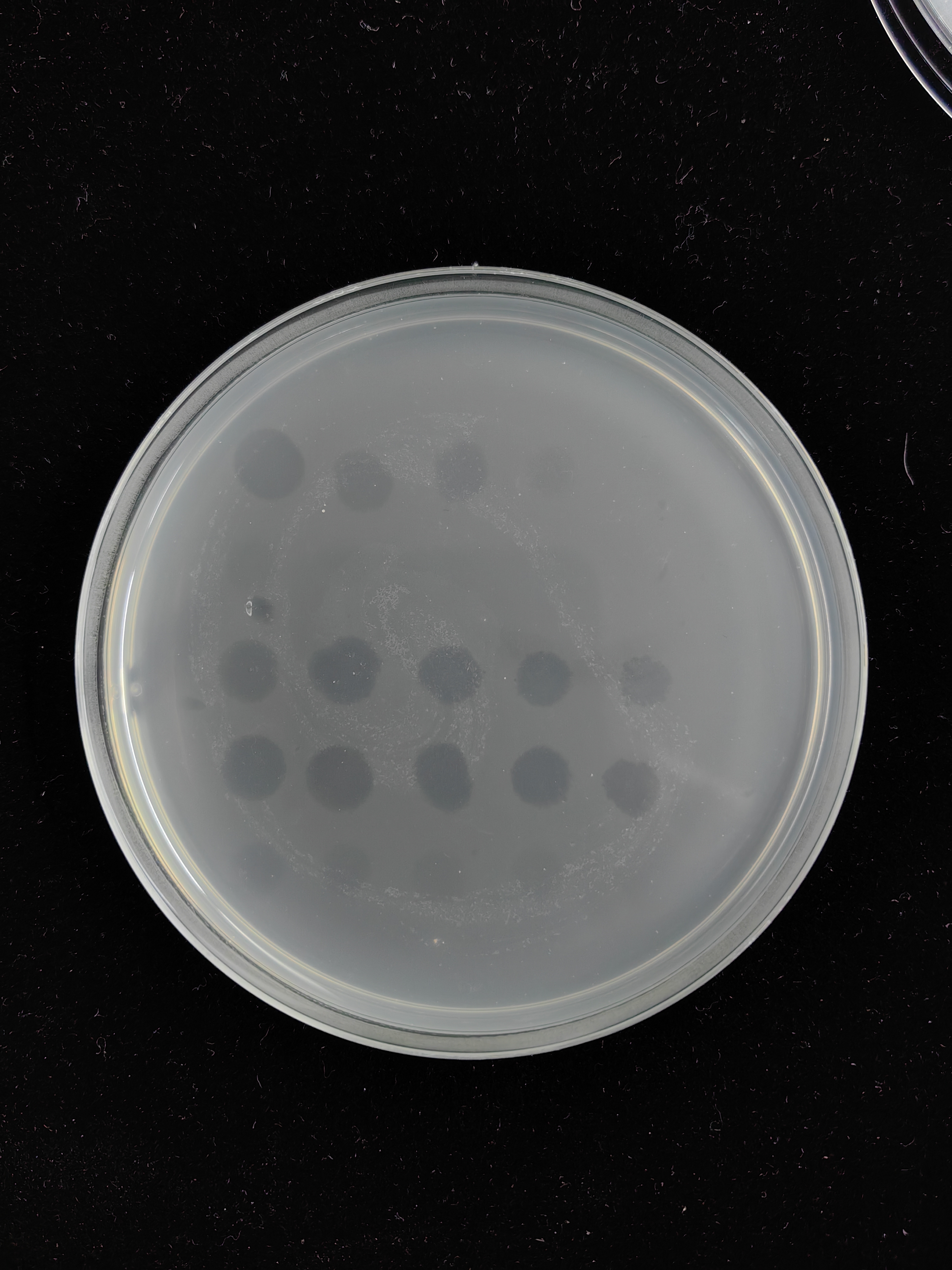

Supplement: Supplementary file 12 — Figure S7 Source Data [file 44319_2025_488_MOESM12_ESM.zip › Appendix Figure S7/S7B/pJR962-Mra_3122-1 without ATc induction.tiff]

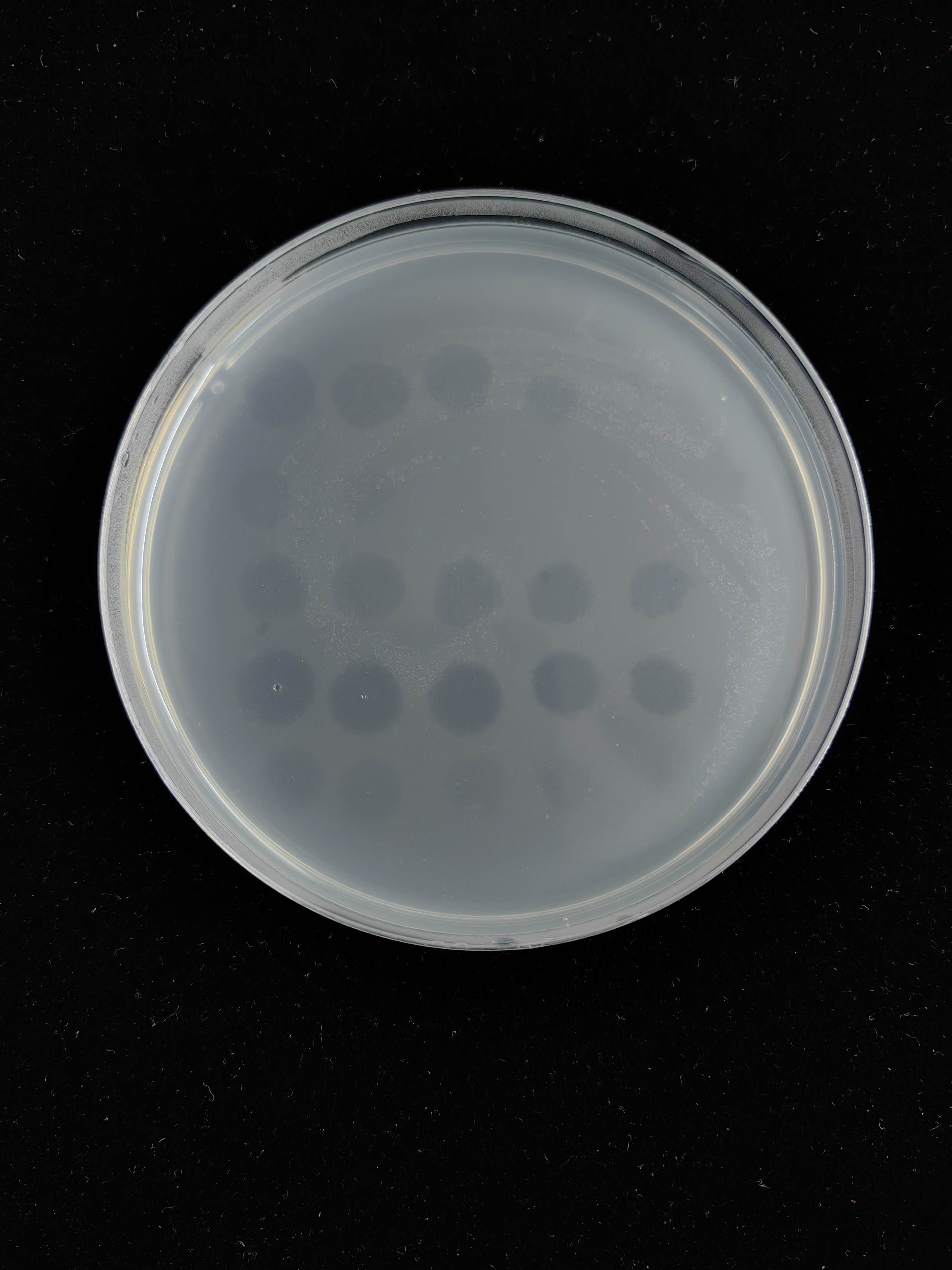

Supplement: Supplementary file 12 — Figure S7 Source Data [file 44319_2025_488_MOESM12_ESM.zip › Appendix Figure S7/S7B/pJR962-Mra_3122-2 with ATc induction.tiff]

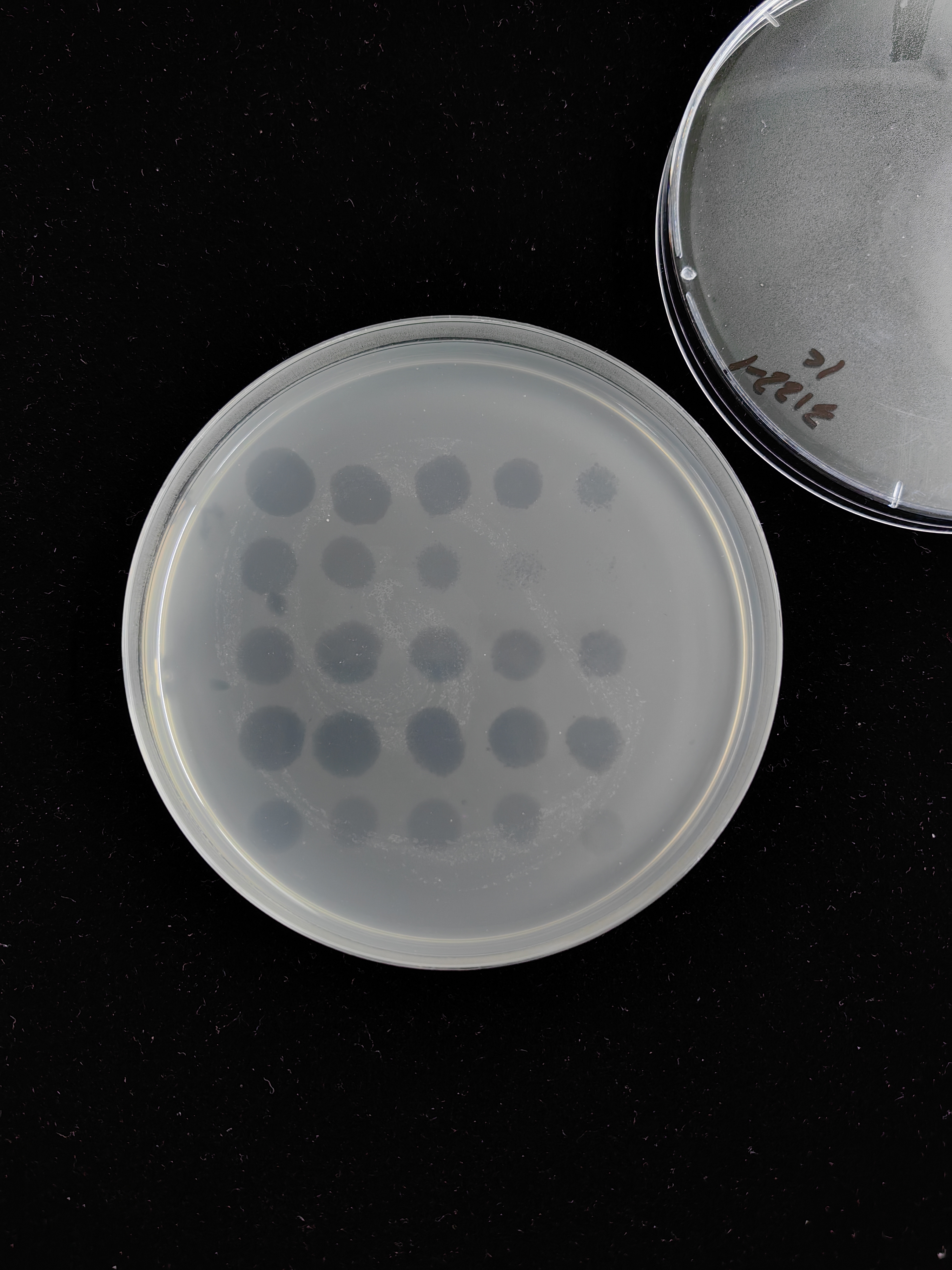

Supplement: Supplementary file 12 — Figure S7 Source Data [file 44319_2025_488_MOESM12_ESM.zip › Appendix Figure S7/S7B/pJR962-Mra_3122-2 without ATc induction.tiff]

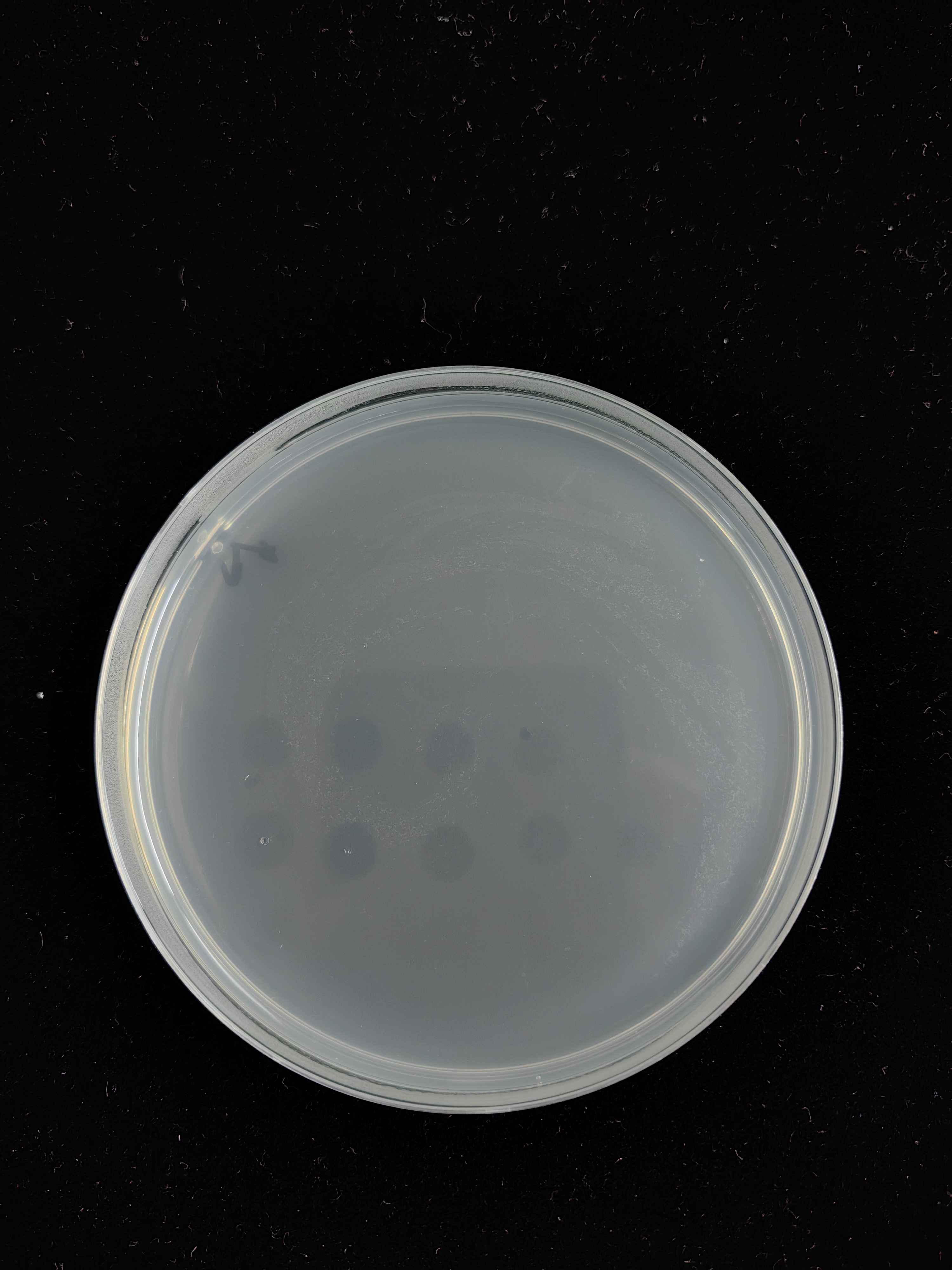

Supplement: Supplementary file 12 — Figure S7 Source Data [file 44319_2025_488_MOESM12_ESM.zip › Appendix Figure S7/S7B/pJR962-Mra_3122-3 with ATc induction.tiff]

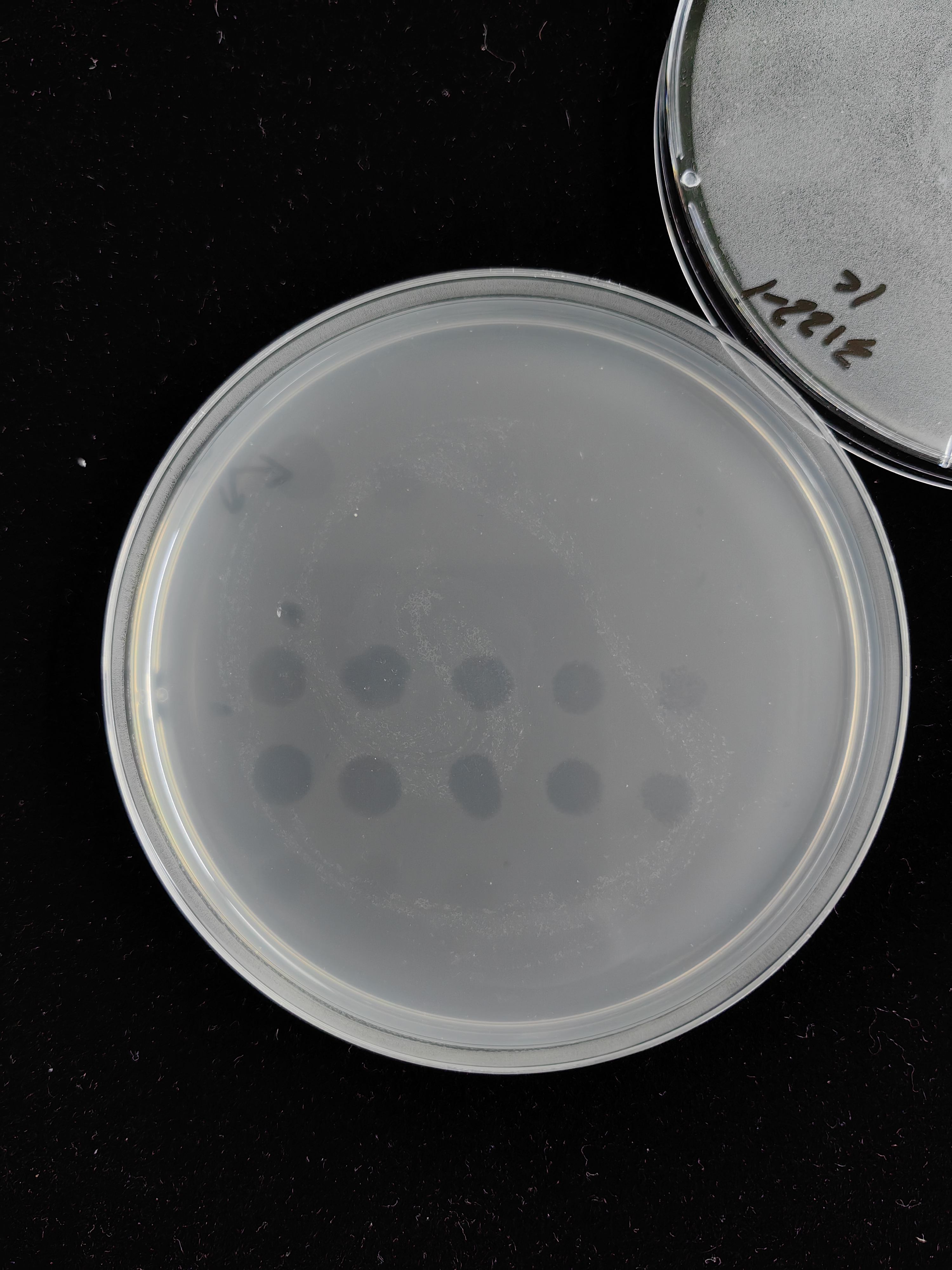

Supplement: Supplementary file 12 — Figure S7 Source Data [file 44319_2025_488_MOESM12_ESM.zip › Appendix Figure S7/S7B/pJR962-Mra_3122-3 without ATc induction.tiff]

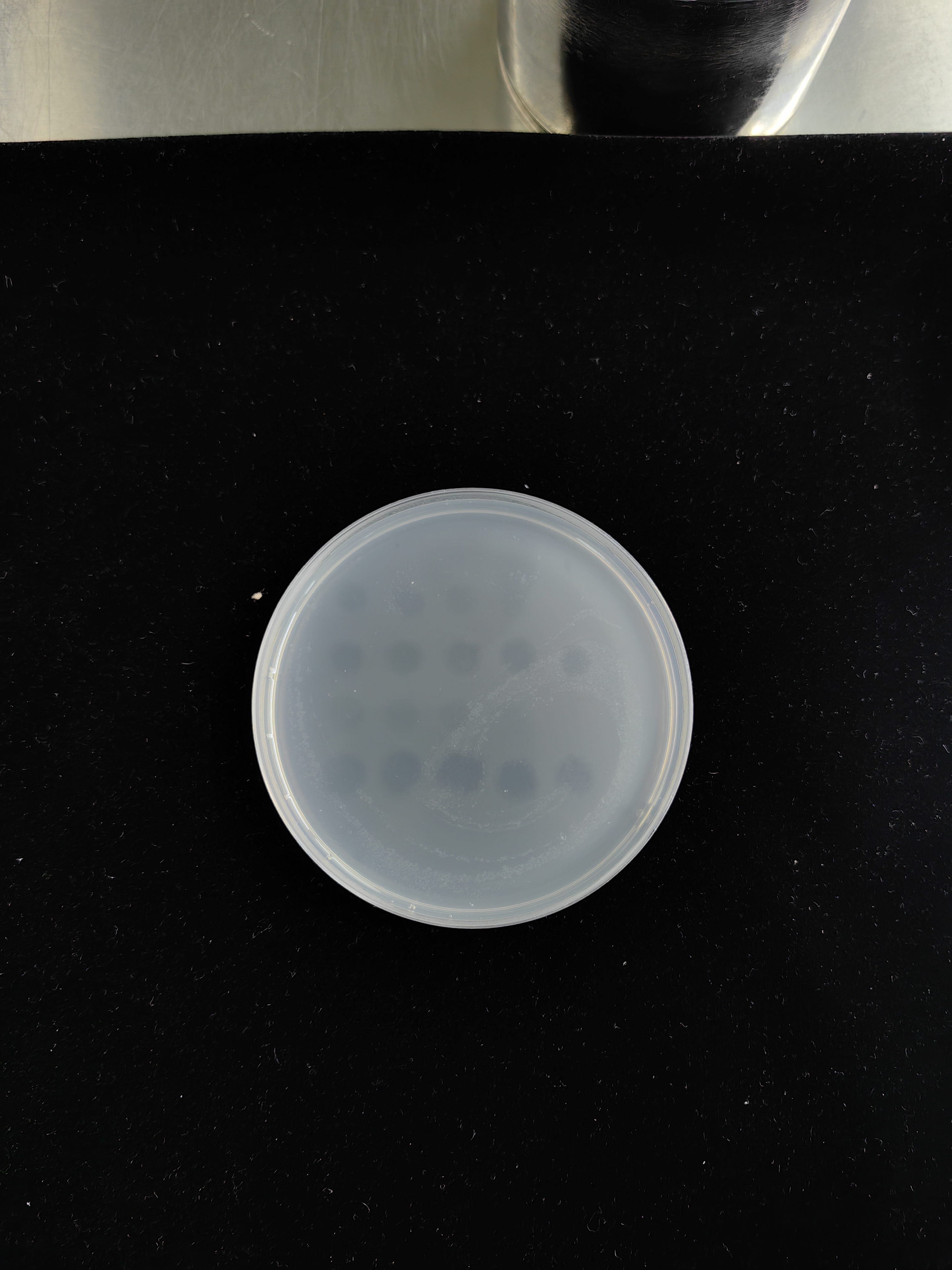

Supplement: Supplementary file 12 — Figure S7 Source Data [file 44319_2025_488_MOESM12_ESM.zip › Appendix Figure S7/S7B/pJR962-Mra_3122-4 with ATc induction.tiff]

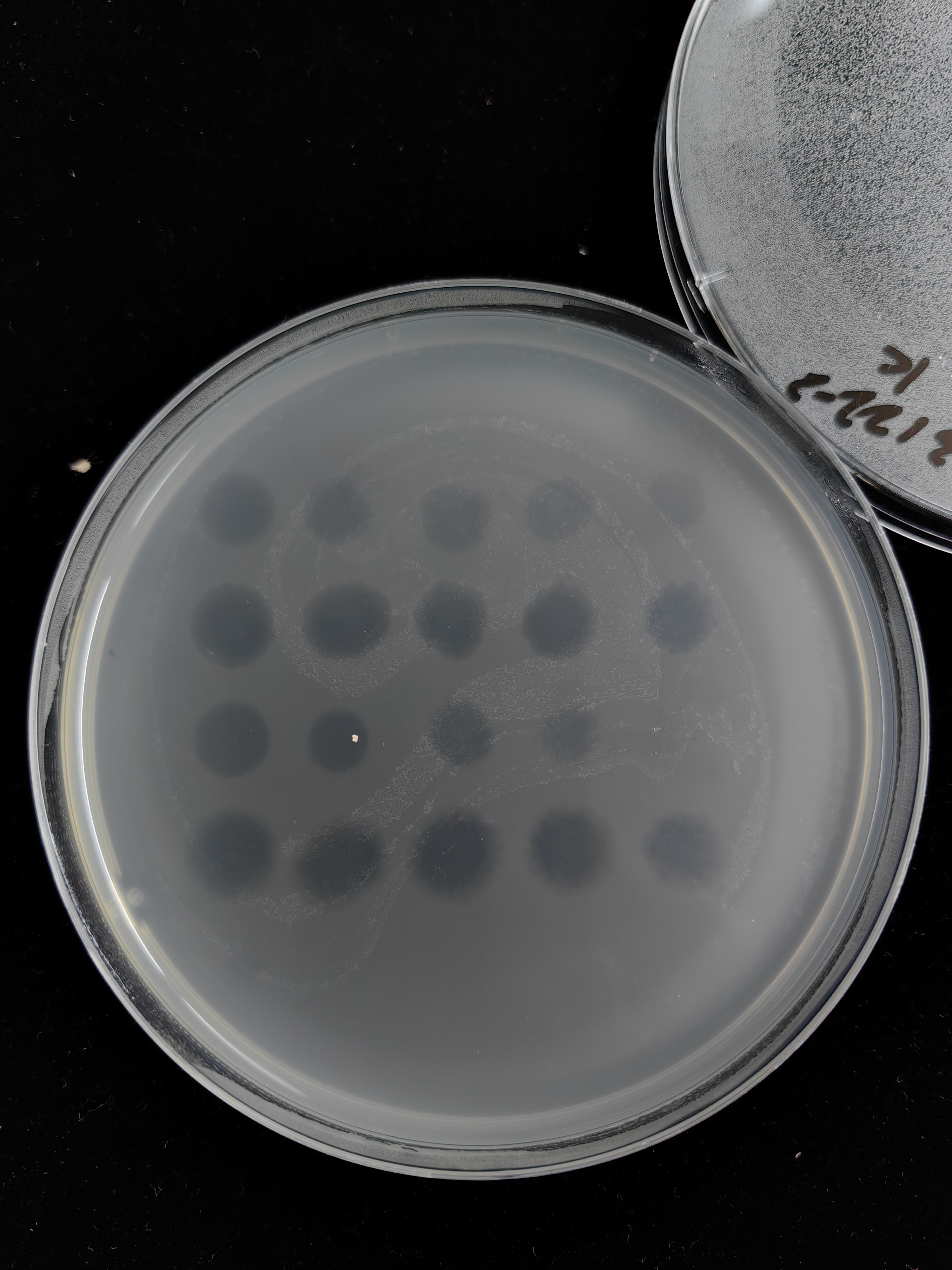

Supplement: Supplementary file 12 — Figure S7 Source Data [file 44319_2025_488_MOESM12_ESM.zip › Appendix Figure S7/S7B/pJR962-Mra_3122-4 without ATc induction.tiff]

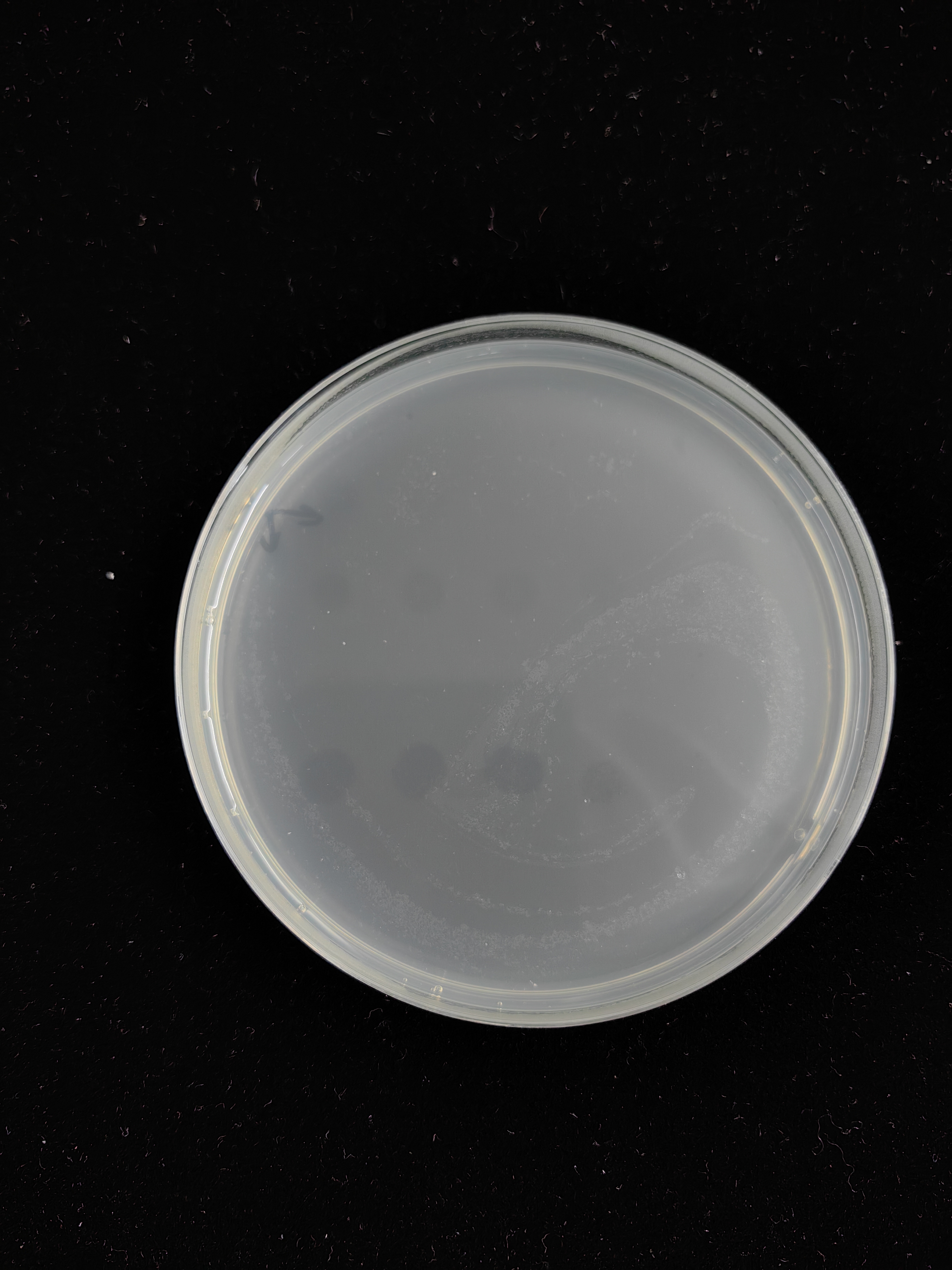

Supplement: Supplementary file 12 — Figure S7 Source Data [file 44319_2025_488_MOESM12_ESM.zip › Appendix Figure S7/S7B/pJR962-Mra_3122-5 with ATc induction.tiff]

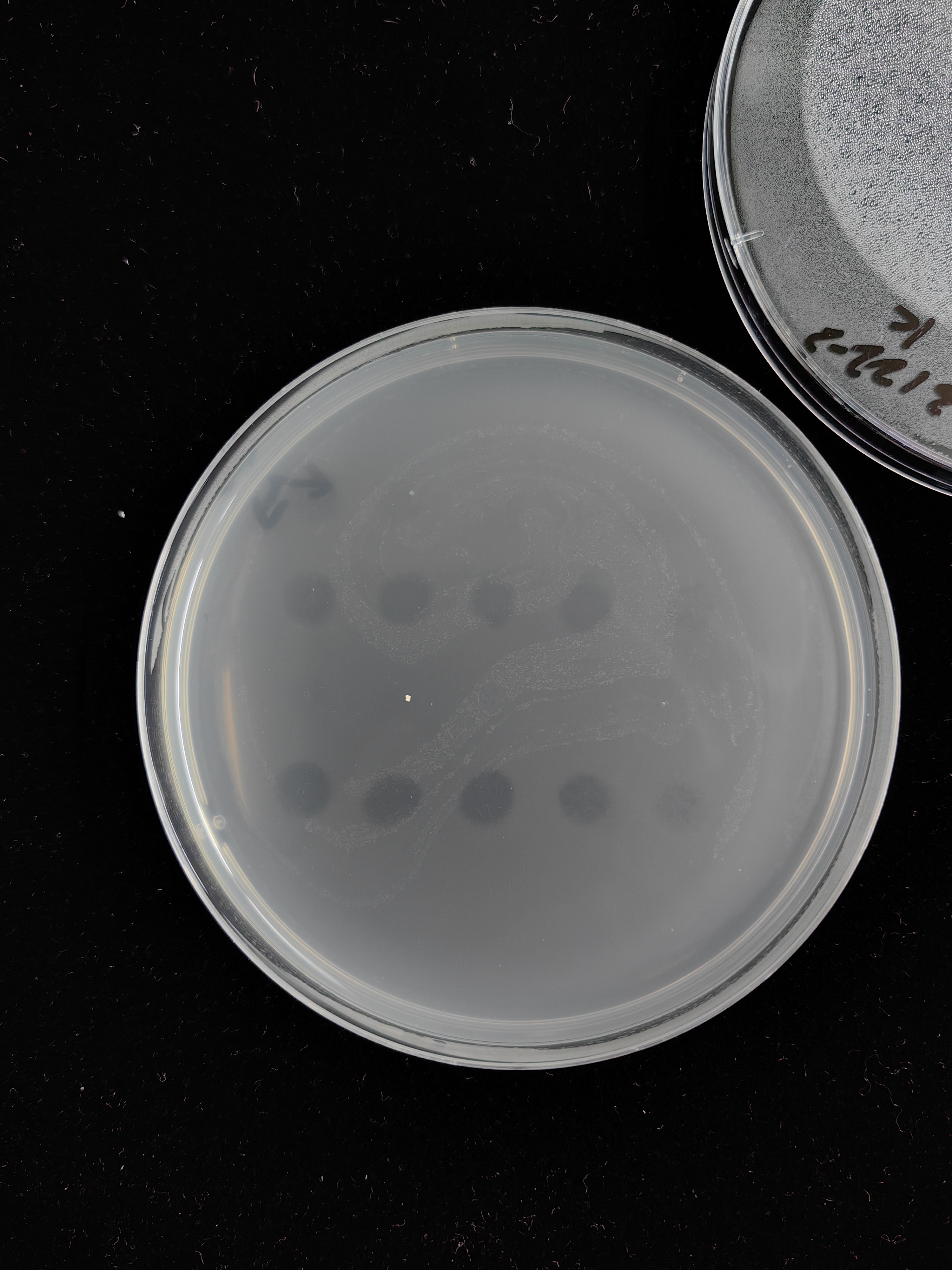

Supplement: Supplementary file 12 — Figure S7 Source Data [file 44319_2025_488_MOESM12_ESM.zip › Appendix Figure S7/S7B/pJR962-Mra_3122-5 without ATc induction.tiff]

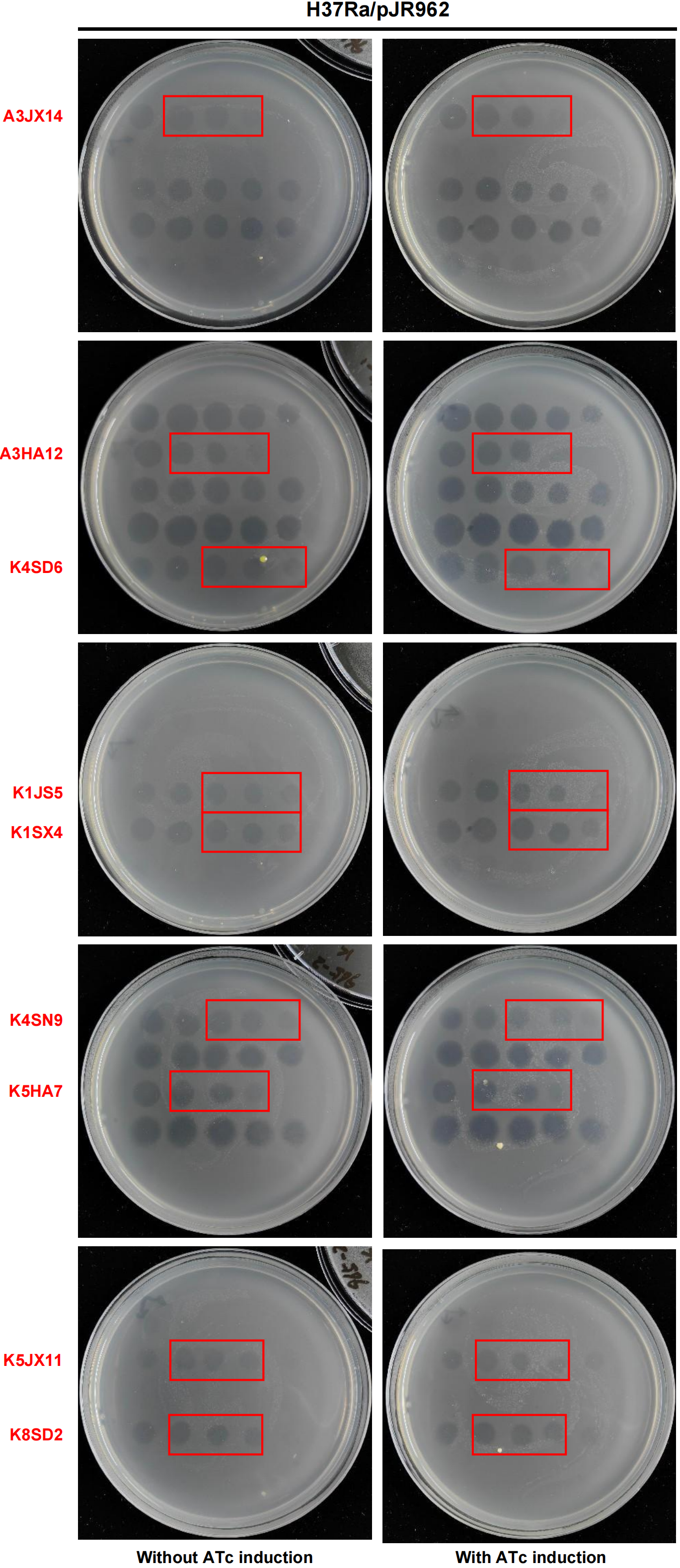

Supplement: Supplementary file 12 — Figure S7 Source Data [file 44319_2025_488_MOESM12_ESM.zip › Appendix Figure S7/S7B/README-1.tif]

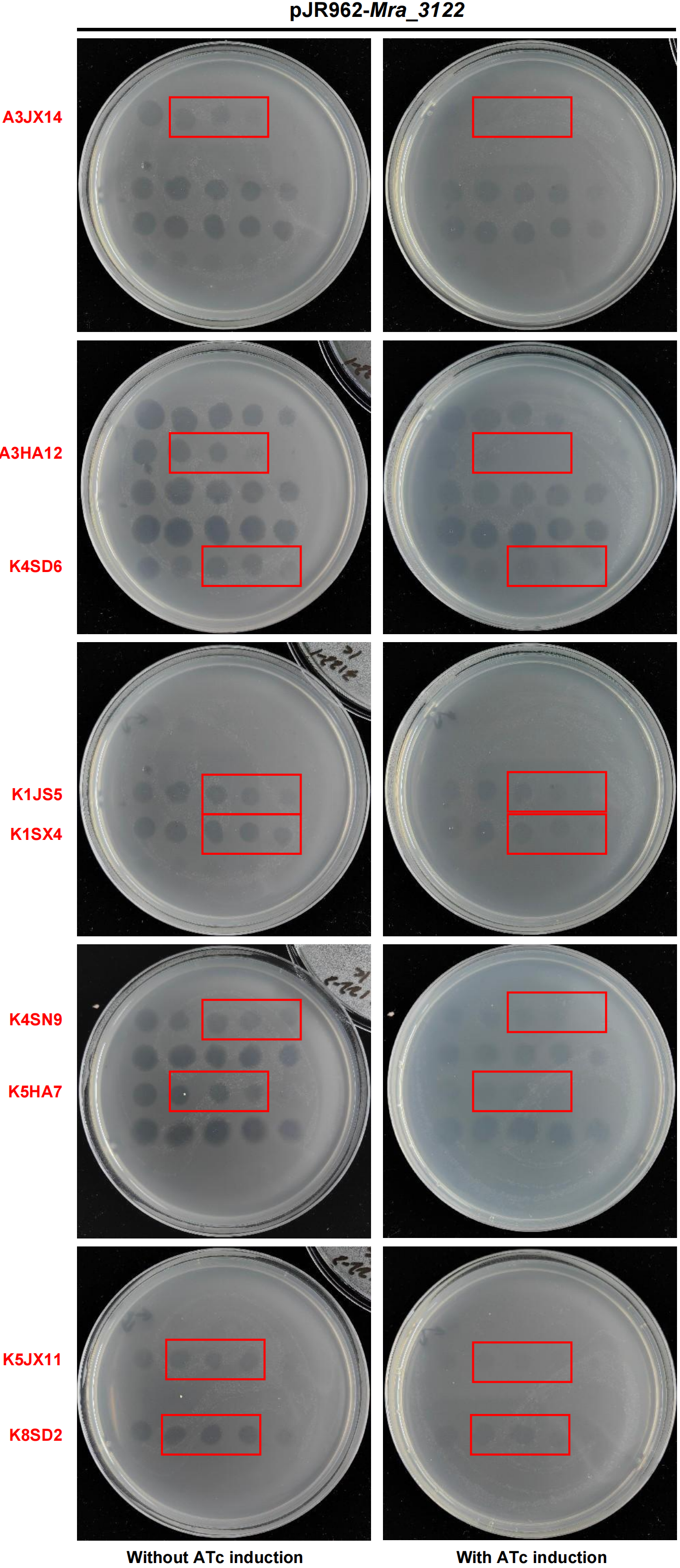

Supplement: Supplementary file 12 — Figure S7 Source Data [file 44319_2025_488_MOESM12_ESM.zip › Appendix Figure S7/S7B/README-2.tif]

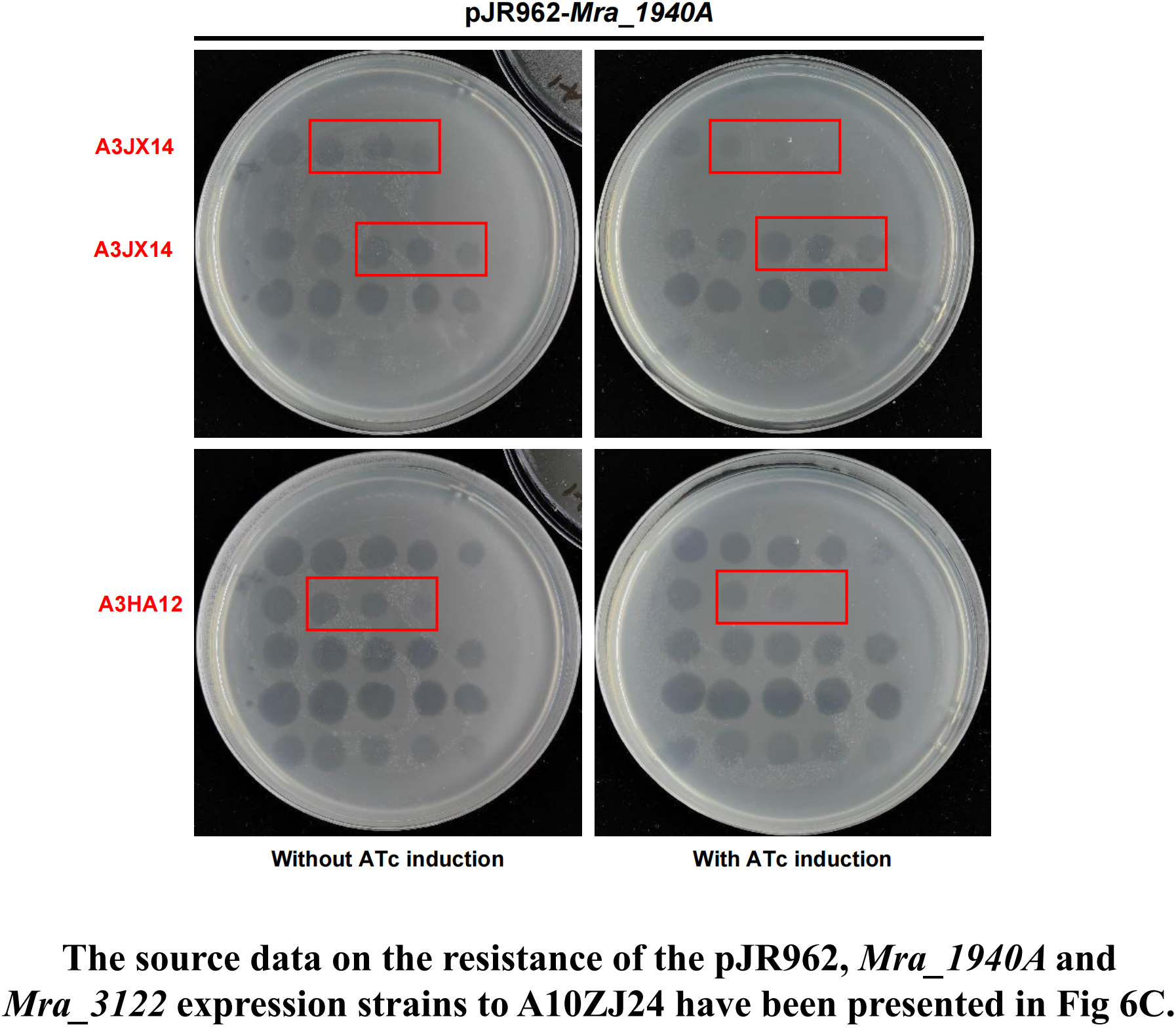

Supplement: Supplementary file 12 — Figure S7 Source Data [file 44319_2025_488_MOESM12_ESM.zip › Appendix Figure S7/S7B/README_3.tif]
